# Supplementary material for: Global and regional perspectives on optimizing thermo-responsive dynamic windows for energy-efficient buildings
Source: Nat Commun. 2025 Jan 2;16:199. doi: 10.1038/s41467-024-54967-8 (PMC11695856; doi:10.1038/s41467-024-54967-8)
Supplement: Supplementary file 1 — Supplementary Information [file 41467_2024_54967_MOESM1_ESM.pdf]

Supplementary Information

Global and regional perspectives on optimizing thermo-responsive dynamic windows for energy-efficient buildings

Yuan Gao[1]*, Jacob C. Jonsson[1], D. Charlie Curcija[1], Simon Vidanovic[1], Tianzhen Hong[1]

[1]Building Technology & Urban Systems Division, Lawrence Berkeley National Laboratory, Berkeley, CA, USA

*Email: y.gao@lbl.gov

Table of contents

Supplementary Figures 1-125

Supplementary Tables 1-3

Supplementary Note 1 (including Supplementary Figures 126-127 and Tables 4-6)

Supplementary References 1-86

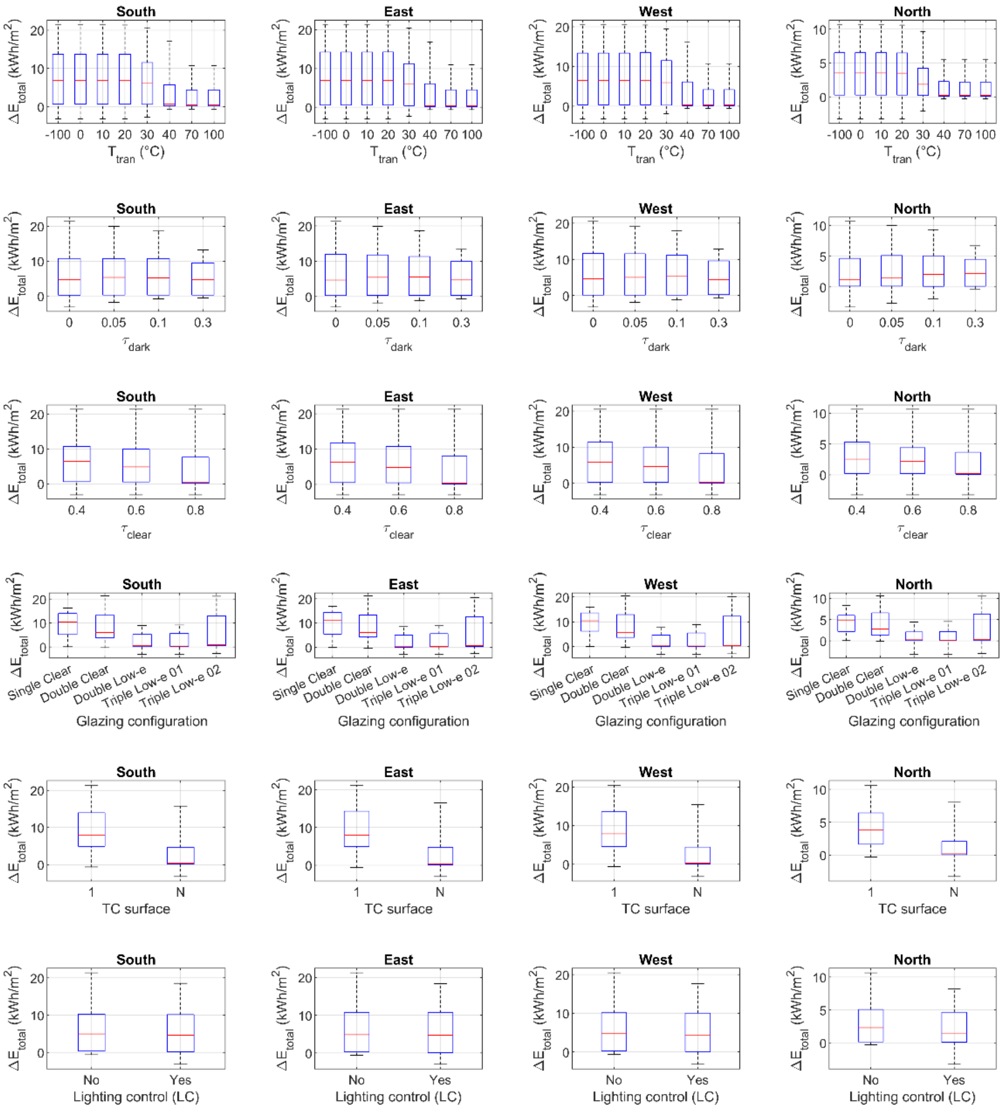

Supplementary Figure 1| Statistical analysis of the total site energy saving per conditioned floor area (ΔE_{total}) by TR windows with six variables (T_{tran}, τ_{dark}, τ_{clear}, glazing configuration, TR-applied surface, and lighting control) in four window orientations in Miami, Florida (climate classification: 1A). In each box plot, the red central mark on each box indicates the median, and the bottom and top edges of the box indicate the 25th and 75th percentiles, respectively. The short black marks above and below each box indicate the maximum and minimum, respectively.

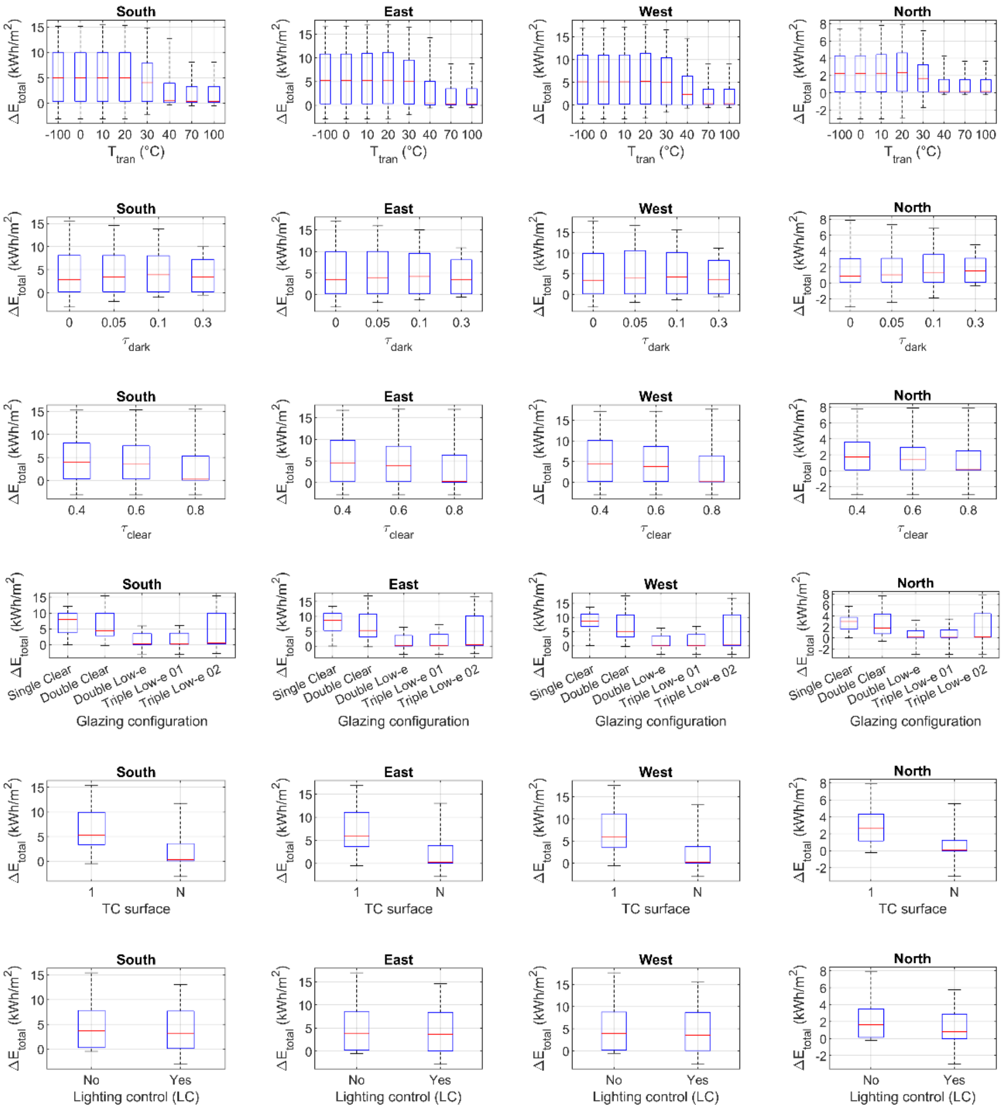

Supplementary Figure 2| Statistical analysis of the total site energy saving per conditioned floor area (ΔE_{total}) by TR windows with six variables (T_{tran}, τ_{dark}, τ_{clear}, glazing configuration, TR-applied surface, and lighting control) in four window orientations in Houston, Texas (climate classification: 2A). In each box plot, the red central mark on each box indicates the median, and the bottom and top edges of the box indicate the 25th and 75th percentiles, respectively. The short black marks above and below each box indicate the maximum and minimum, respectively.

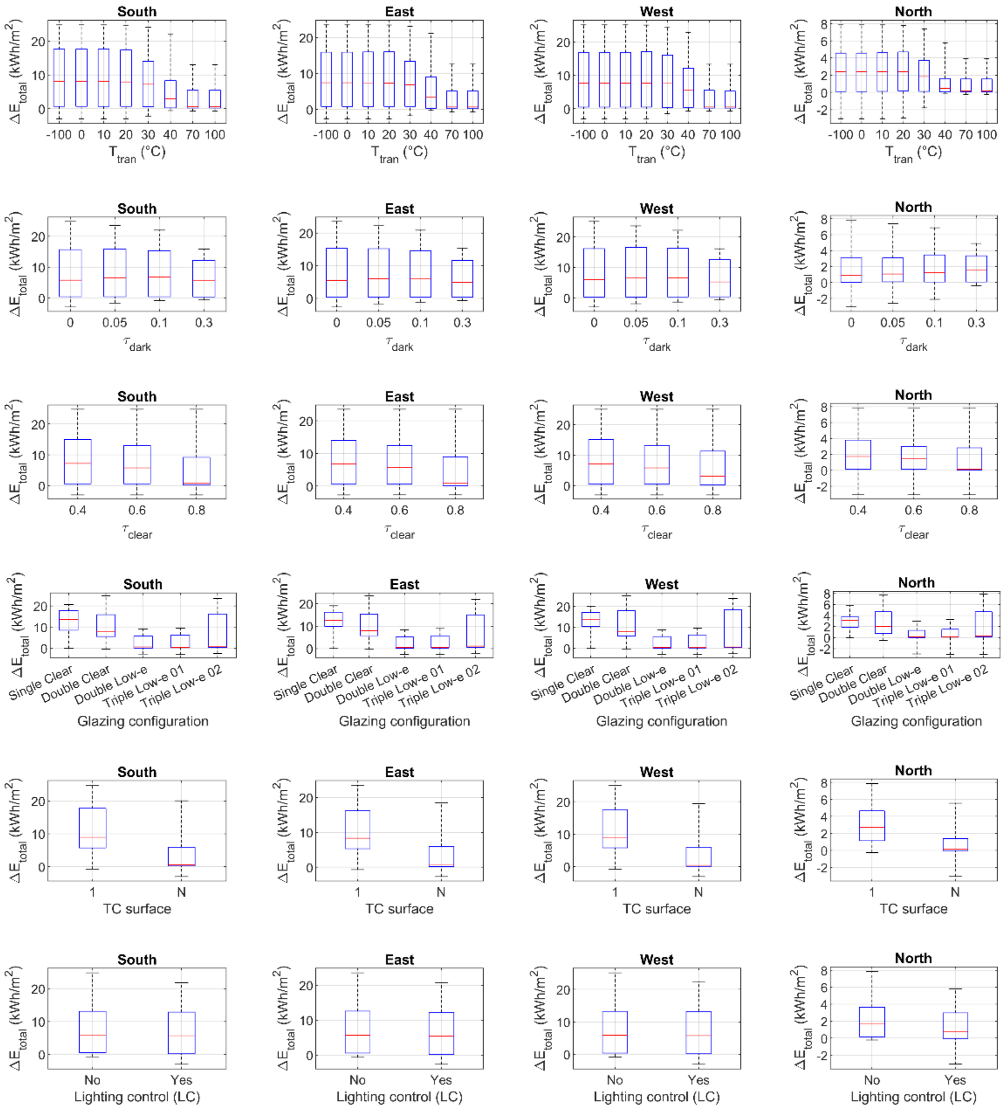

Supplementary Figure 3| Statistical analysis of the total site energy saving per conditioned floor area (ΔE_{total}) by TR windows with six variables (T_{tran}, τ_{dark}, τ_{clear}, glazing configuration, TR-applied surface, and lighting control) in four window orientations in Phoenix, Arizona (climate classification: 2B). In each box plot, the red central mark on each box indicates the median, and the bottom and top edges of the box indicate the 25th and 75th percentiles, respectively. The short black marks above and below each box indicate the maximum and minimum, respectively.

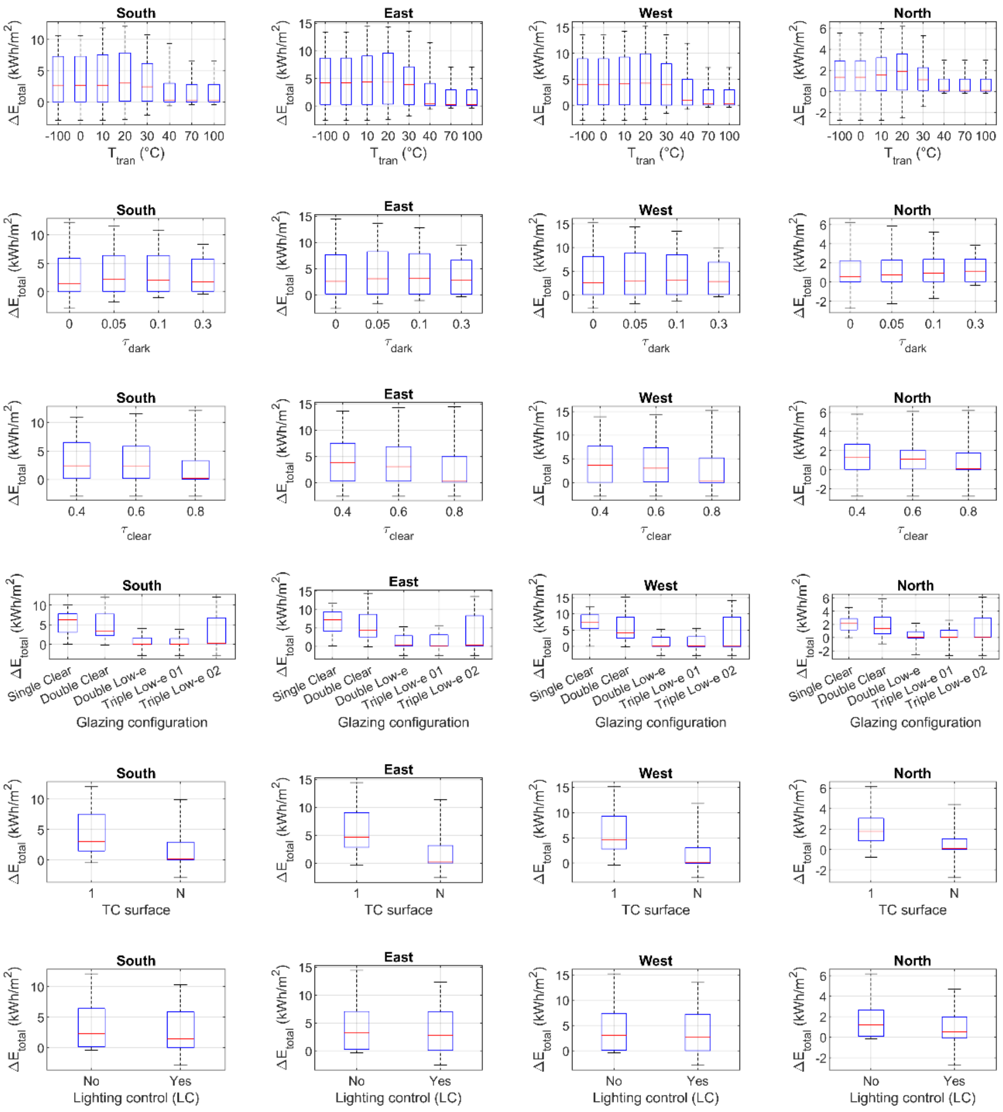

Supplementary Figure 4| Statistical analysis of the total site energy saving per conditioned floor area (ΔE_{total}) by TR windows with six variables (T_{tran}, τ_{dark}, τ_{clear}, glazing configuration, TR-applied surface, and lighting control) in four window orientations in Atlanta, Georgia (climate classification: 3A). In each box plot, the red central mark on each box indicates the median, and the bottom and top edges of the box indicate the 25th and 75th percentiles, respectively. The short black marks above and below each box indicate the maximum and minimum, respectively.

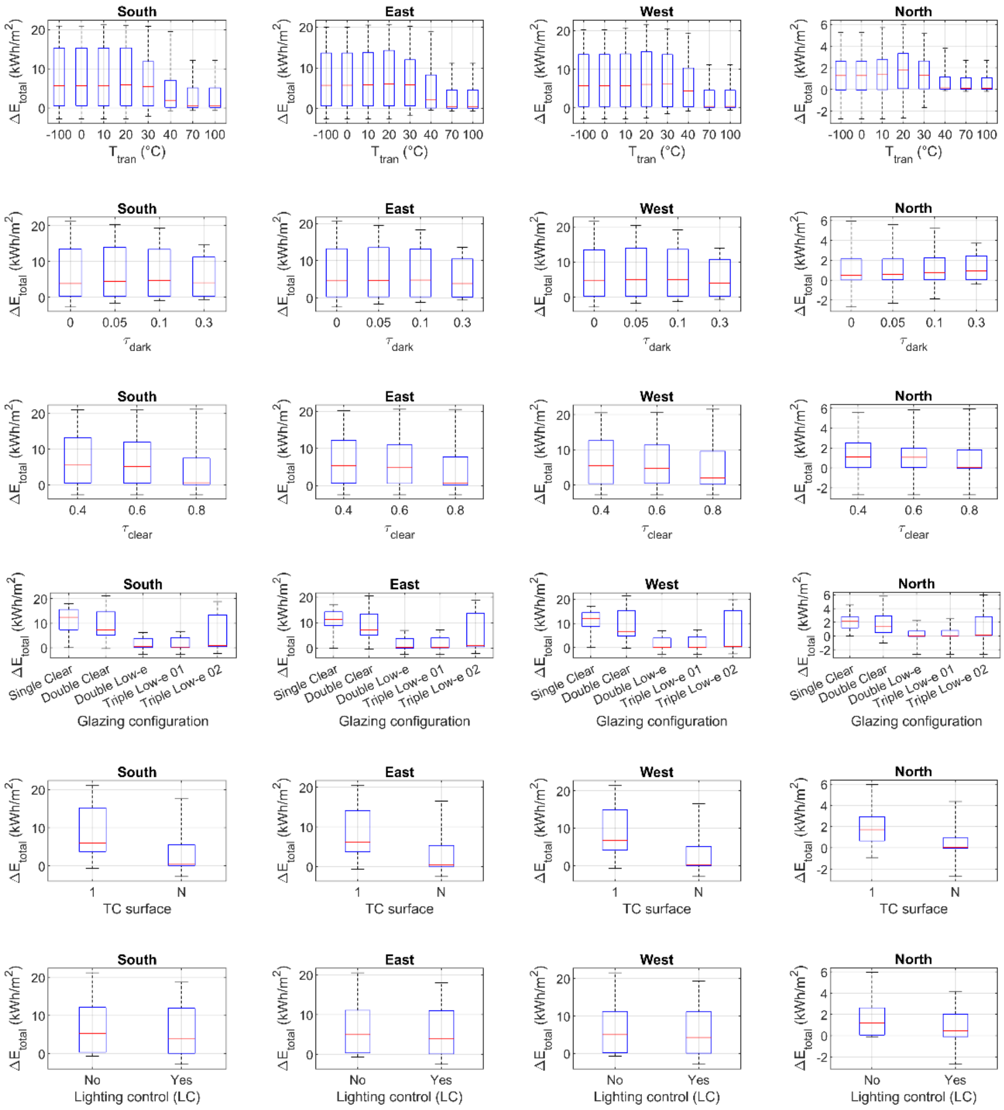

Supplementary Figure 5| Statistical analysis of the total site energy saving per conditioned floor area (ΔE_{total}) by TR windows with six variables (T_{tran}, τ_{dark}, τ_{clear}, glazing configuration, TR-applied surface, and lighting control) in four window orientations in Las Vegas, Nevada (climate classification: 3B). In each box plot, the red central mark on each box indicates the median, and the bottom and top edges of the box indicate the 25th and 75th percentiles, respectively. The short black marks above and below each box indicate the maximum and minimum, respectively.

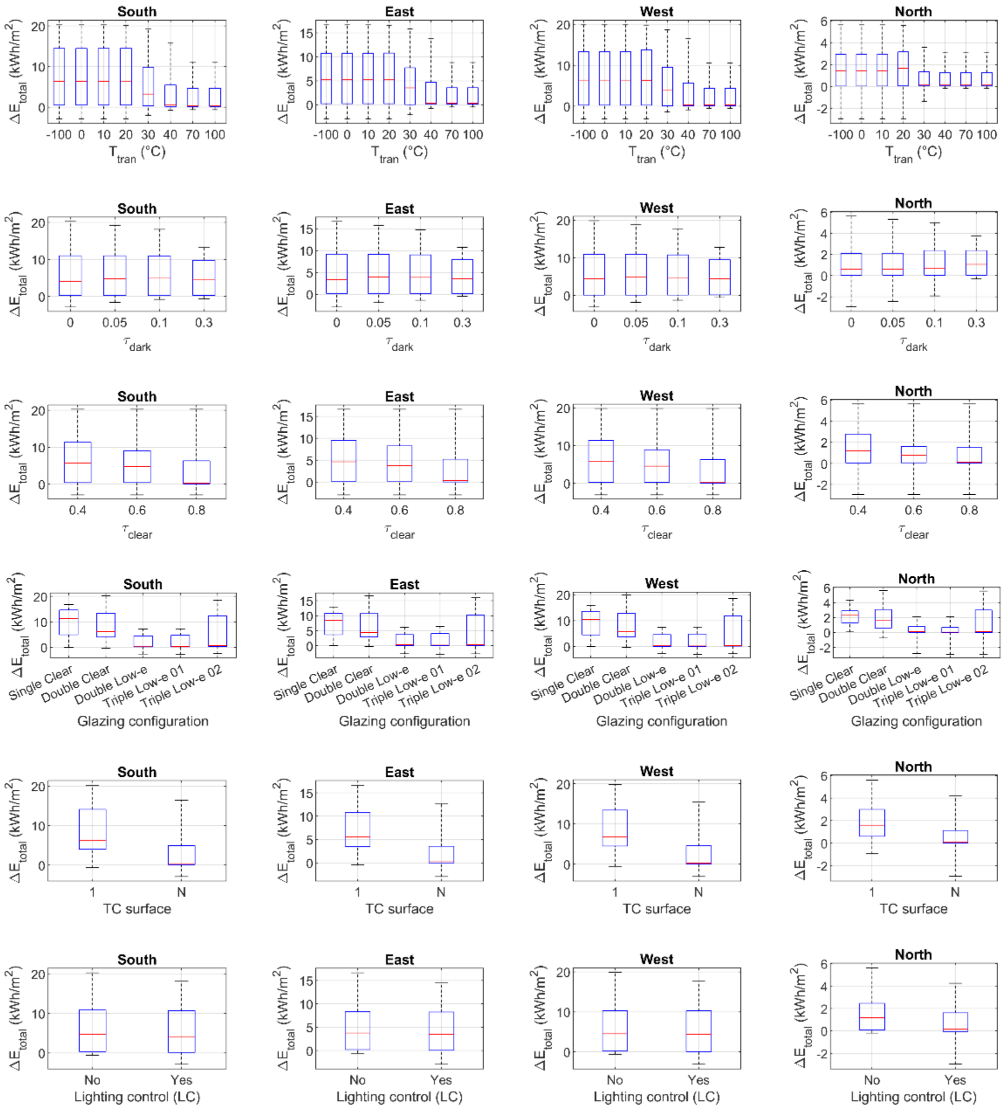

Supplementary Figure 6| Statistical analysis of the total site energy saving per conditioned floor area (ΔE_{total}) by TR windows with six variables (T_{tran}, τ_{dark}, τ_{clear}, glazing configuration, TR-applied surface, and lighting control) in four window orientations in Los Angeles, California (climate classification: 3B). In each box plot, the red central mark on each box indicates the median, and the bottom and top edges of the box indicate the 25th and 75th percentiles, respectively. The short black marks above and below each box indicate the maximum and minimum, respectively.

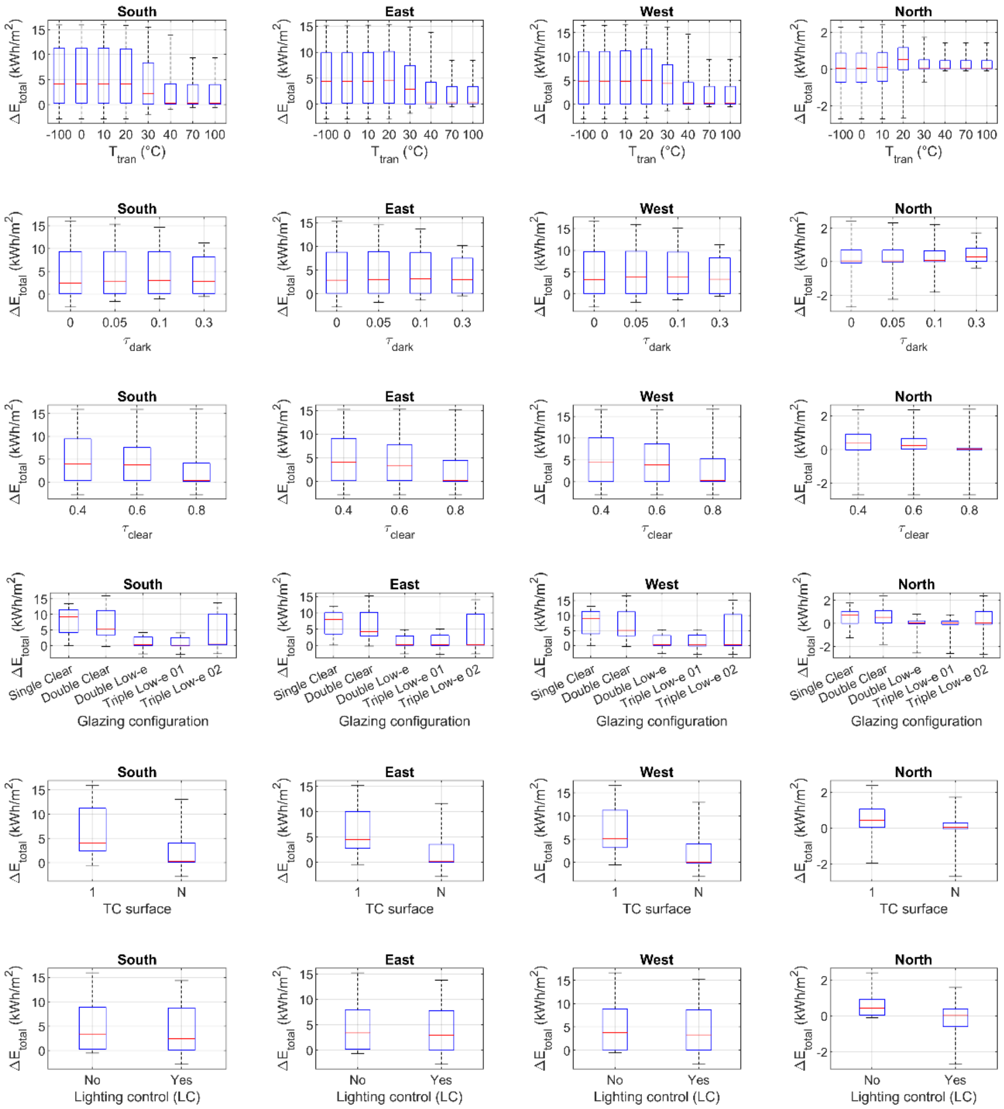

Supplementary Figure 7| Statistical analysis of the total site energy saving per conditioned floor area (ΔE_{total}) by TR windows with six variables (T_{tran}, τ_{dark}, τ_{clear}, glazing configuration, TR-applied surface, and lighting control) in four window orientations in San Francisco, California (climate classification: 3C). In each box plot, the red central mark on each box indicates the median, and the bottom and top edges of the box indicate the 25th and 75th percentiles, respectively. The short black marks above and below each box indicate the maximum and minimum, respectively.

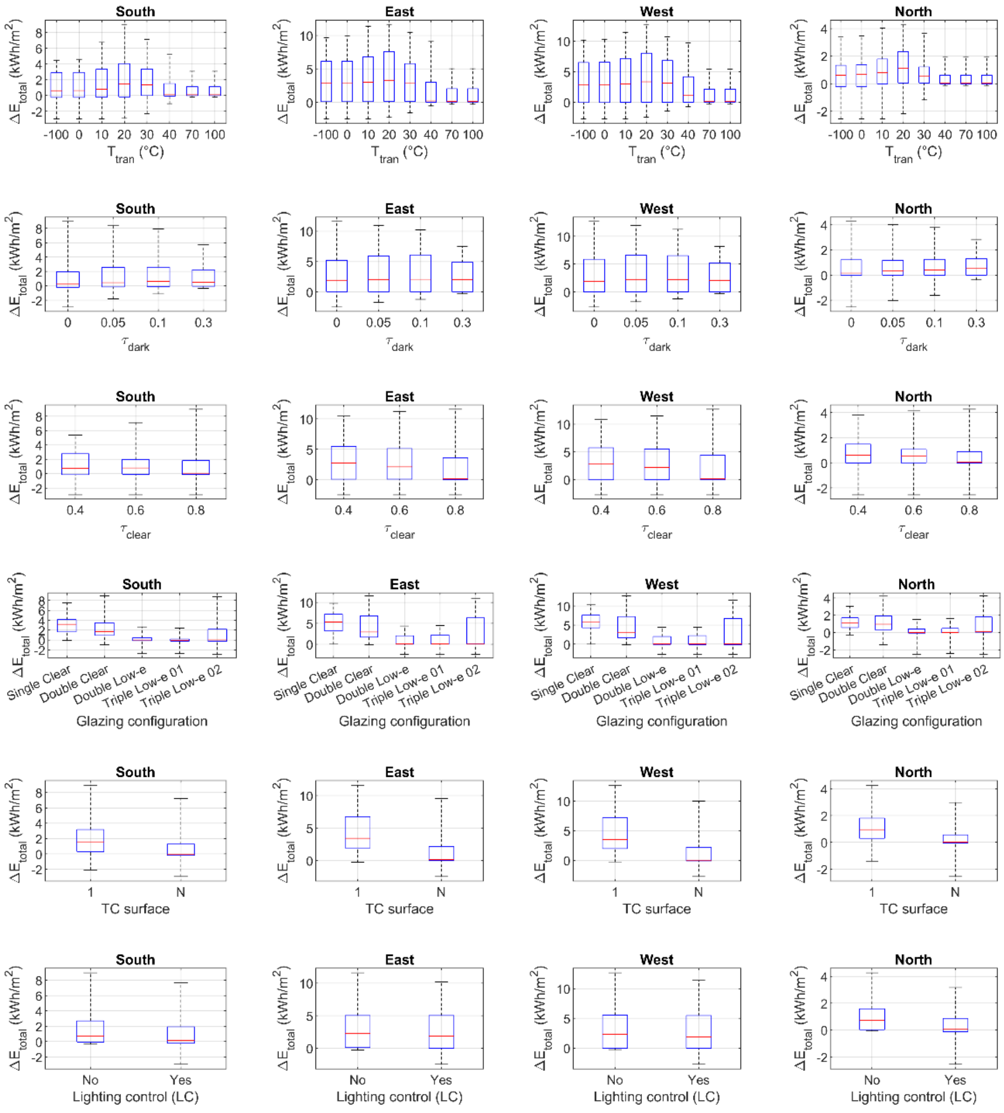

Supplementary Figure 8| Statistical analysis of the total site energy saving per conditioned floor area (ΔE_{total}) by TR windows with six variables (T_{tran}, τ_{dark}, τ_{clear}, glazing configuration, TR-applied surface, and lighting control) in four window orientations in Baltimore, Maryland (climate classification: 4A). In each box plot, the red central mark on each box indicates the median, and the bottom and top edges of the box indicate the 25th and 75th percentiles, respectively. The short black marks above and below each box indicate the maximum and minimum, respectively.

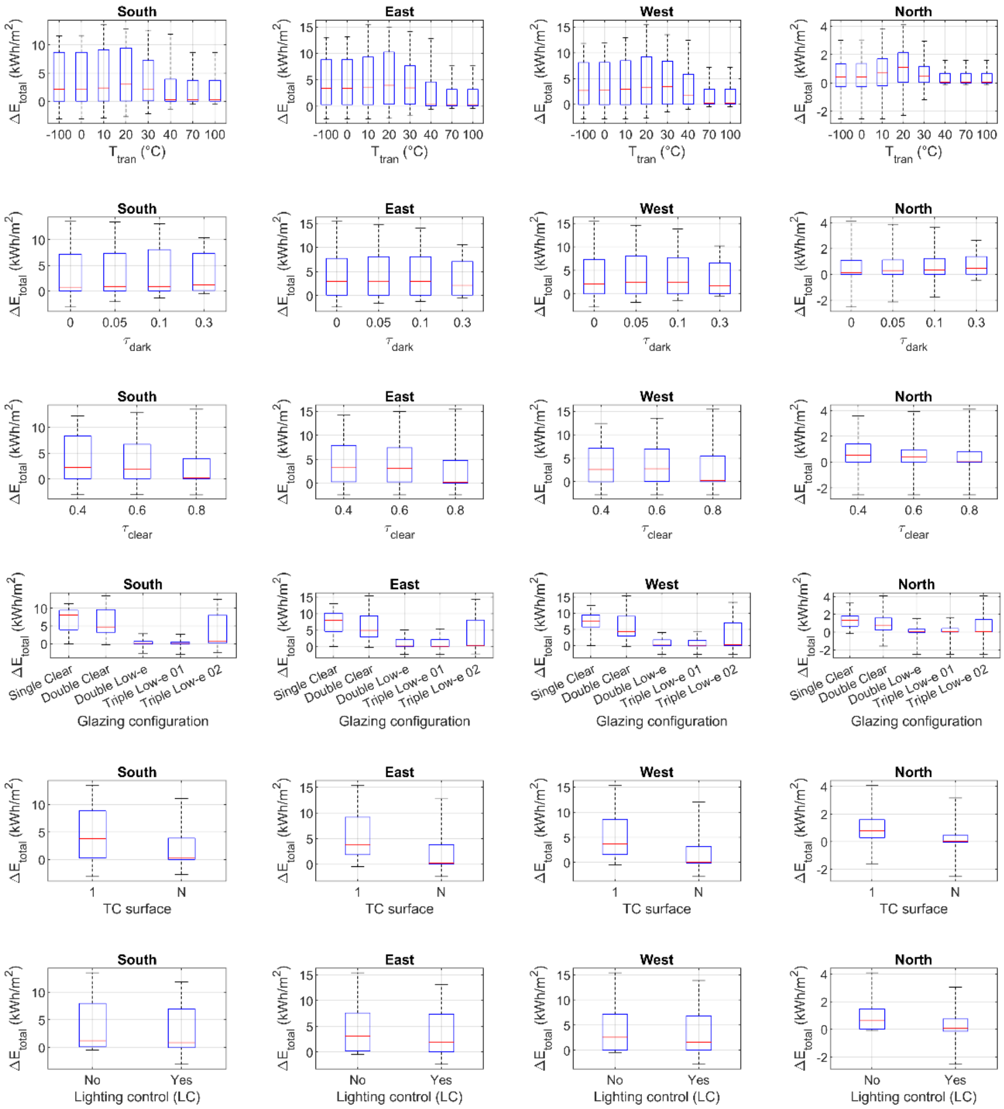

Supplementary Figure 9| Statistical analysis of the total site energy saving per conditioned floor area (ΔE_{total}) by TR windows with six variables (T_{tran}, τ_{dark}, τ_{clear}, glazing configuration, TR-applied surface, and lighting control) in four window orientations in Albuquerque, New Mexico (climate classification: 4B). In each box plot, the red central mark on each box indicates the median, and the bottom and top edges of the box indicate the 25th and 75th percentiles, respectively. The short black marks above and below each box indicate the maximum and minimum, respectively.

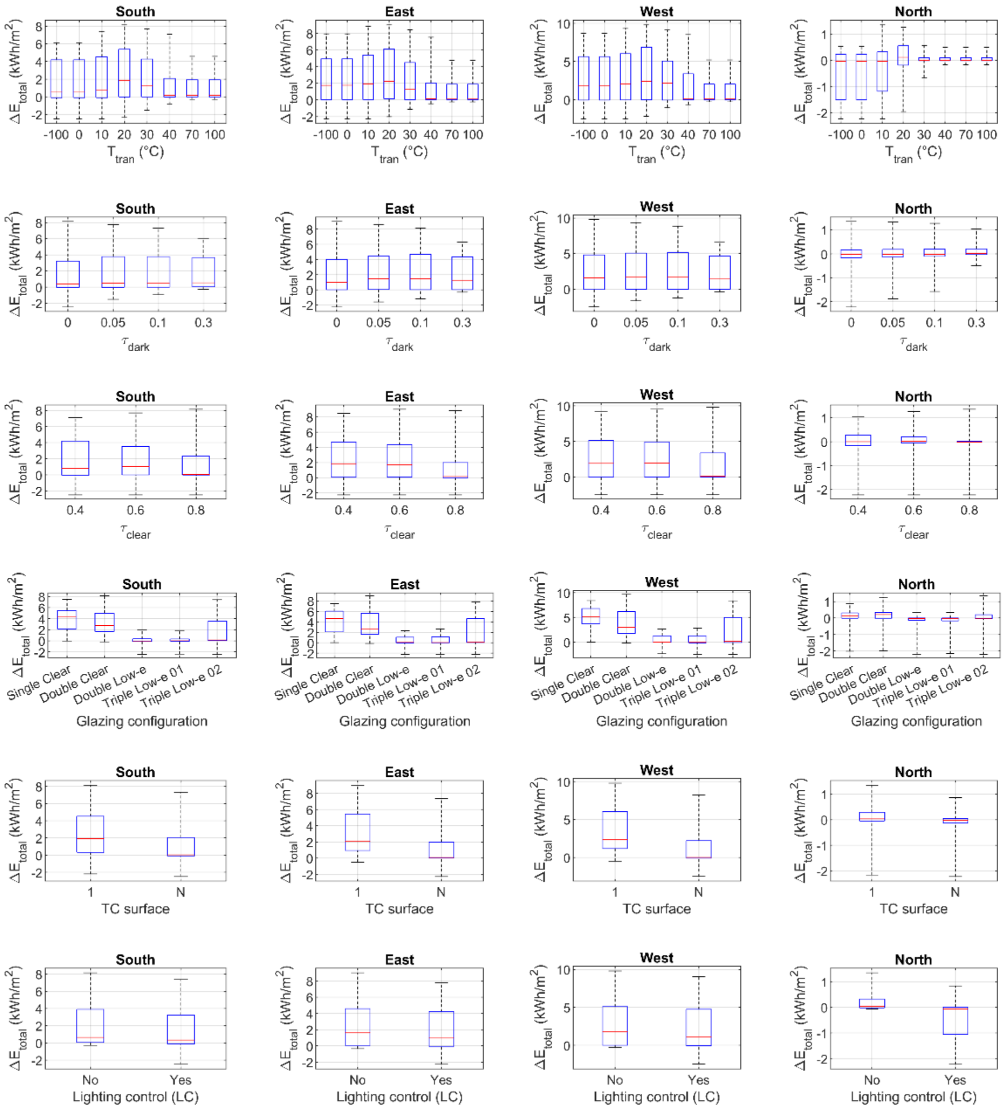

Supplementary Figure 10| Statistical analysis of the total site energy saving per conditioned floor area (ΔE_{total}) by TR windows with six variables (T_{tran}, τ_{dark}, τ_{clear}, glazing configuration, TR-applied surface, and lighting control) in four window orientations in Seattle, Washington (climate classification: 4C). In each box plot, the red central mark on each box indicates the median, and the bottom and top edges of the box indicate the 25th and 75th percentiles, respectively. The short black marks above and below each box indicate the maximum and minimum, respectively.

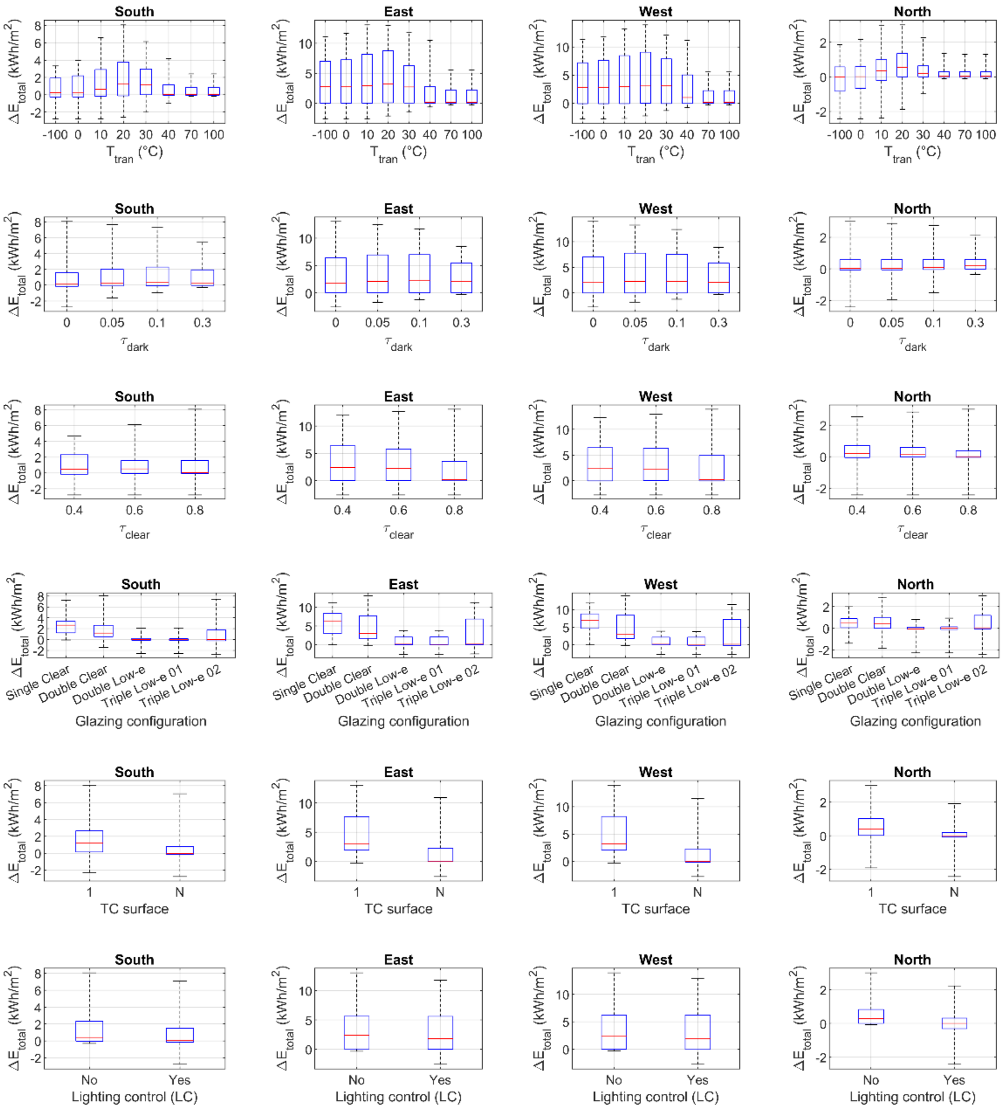

Supplementary Figure 11| Statistical analysis of the total site energy saving per conditioned floor area (ΔE_{total}) by TR windows with six variables (T_{tran}, τ_{dark}, τ_{clear}, glazing configuration, TR-applied surface, and lighting control) in four window orientations in Chicago, Illinois (climate classification: 5A). In each box plot, the red central mark on each box indicates the median, and the bottom and top edges of the box indicate the 25th and 75th percentiles, respectively. The short black marks above and below each box indicate the maximum and minimum, respectively.

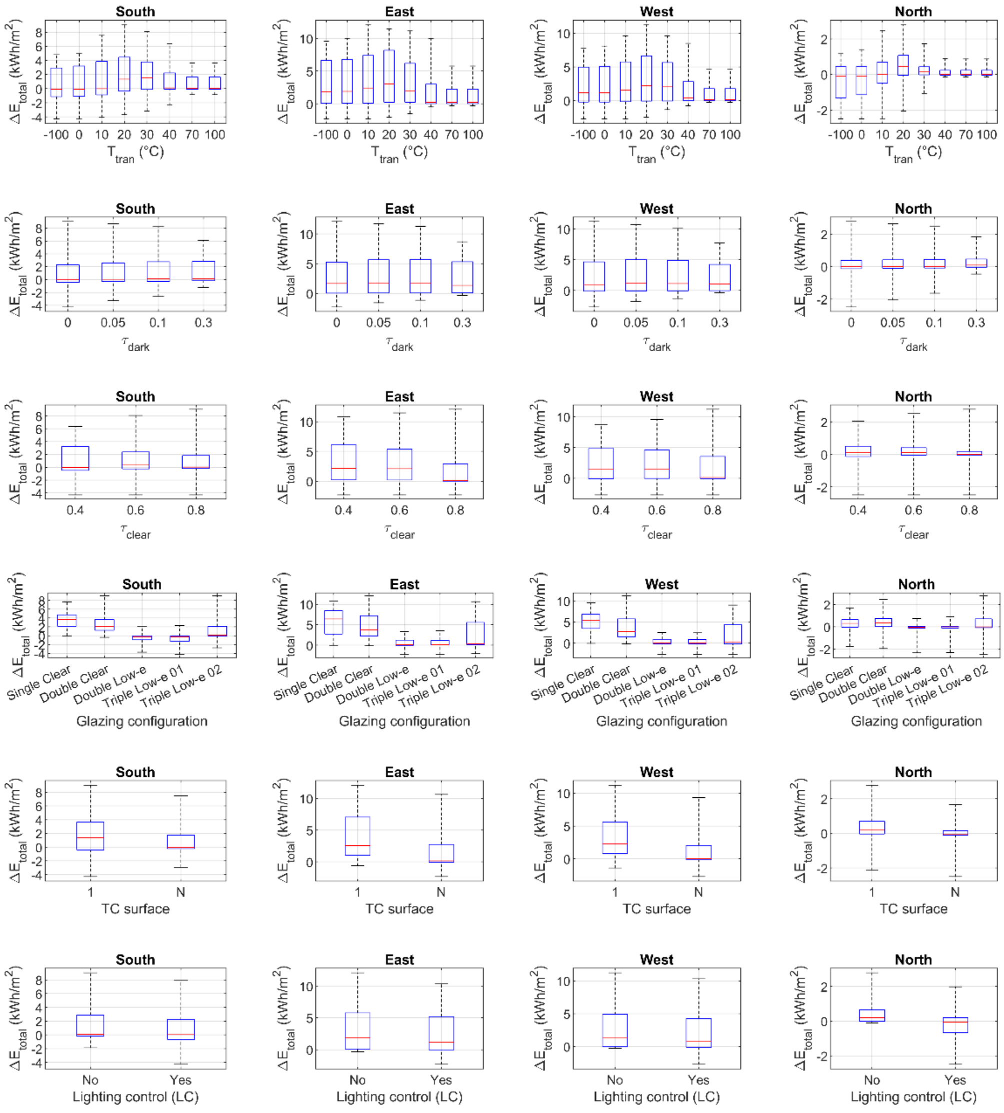

Supplementary Figure 12| Statistical analysis of the total site energy saving per conditioned floor area (ΔE_{total}) by TR windows with six variables (T_{tran}, τ_{dark}, τ_{clear}, glazing configuration, TR-applied surface, and lighting control) in four window orientations in Boulder, Colorado (climate classification: 5B). In each box plot, the red central mark on each box indicates the median, and the bottom and top edges of the box indicate the 25th and 75th percentiles, respectively. The short black marks above and below each box indicate the maximum and minimum, respectively.

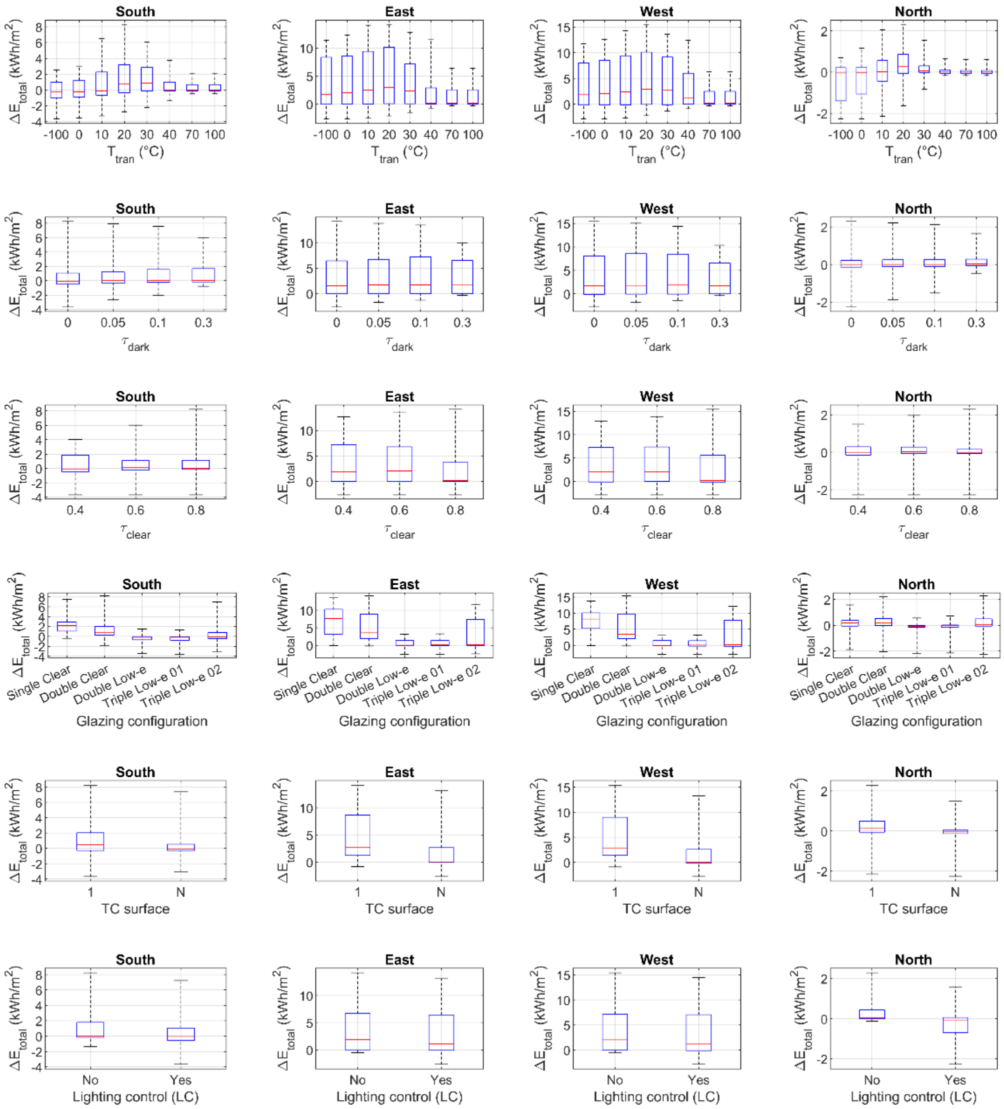

Supplementary Figure 13| Statistical analysis of the total site energy saving per conditioned floor area (ΔE_{total}) by TR windows with six variables (T_{tran}, τ_{dark}, τ_{clear}, glazing configuration, TR-applied surface, and lighting control) in four window orientations in Minneapolis, Minnesota (climate classification: 6A). In each box plot, the red central mark on each box indicates the median, and the bottom and top edges of the box indicate the 25th and 75th percentiles, respectively. The short black marks above and below each box indicate the maximum and minimum, respectively.

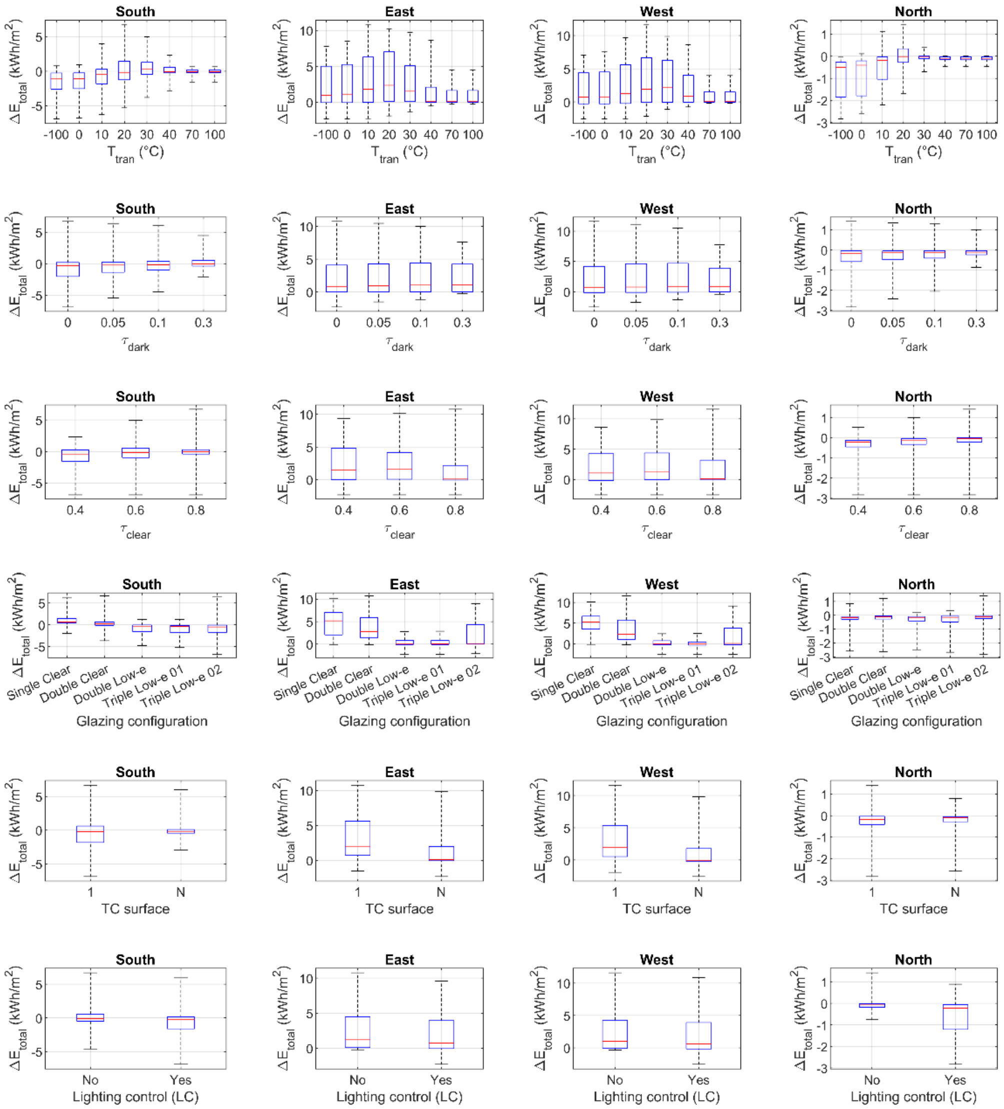

Supplementary Figure 14| Statistical analysis of the total site energy saving per conditioned floor area (ΔE_{total}) by TR windows with six variables (T_{tran}, τ_{dark}, τ_{clear}, glazing configuration, TR-applied surface, and lighting control) in four window orientations in Helena, Montana (climate classification: 6B). In each box plot, the red central mark on each box indicates the median, and the bottom and top edges of the box indicate the 25th and 75th percentiles, respectively. The short black marks above and below each box indicate the maximum and minimum, respectively.

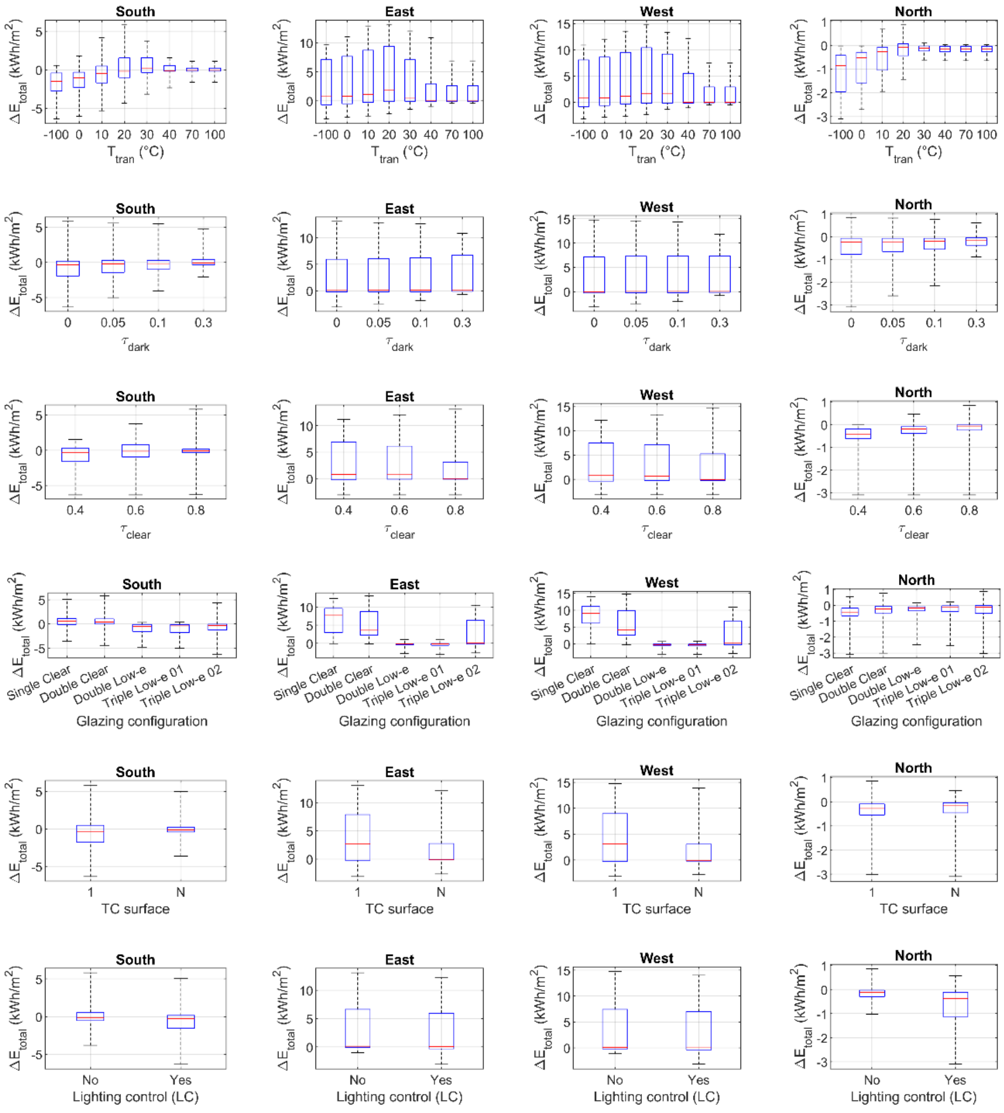

Supplementary Figure 15| Statistical analysis of the total site energy saving per conditioned floor area (ΔE_{total}) by TR windows with six variables (T_{tran}, τ_{dark}, τ_{clear}, glazing configuration, TR-applied surface, and lighting control) in four window orientations in Duluth, Minnesota (climate classification: 7). In each box plot, the red central mark on each box indicates the median, and the bottom and top edges of the box indicate the 25th and 75th percentiles, respectively. The short black marks above and below each box indicate the maximum and minimum, respectively.

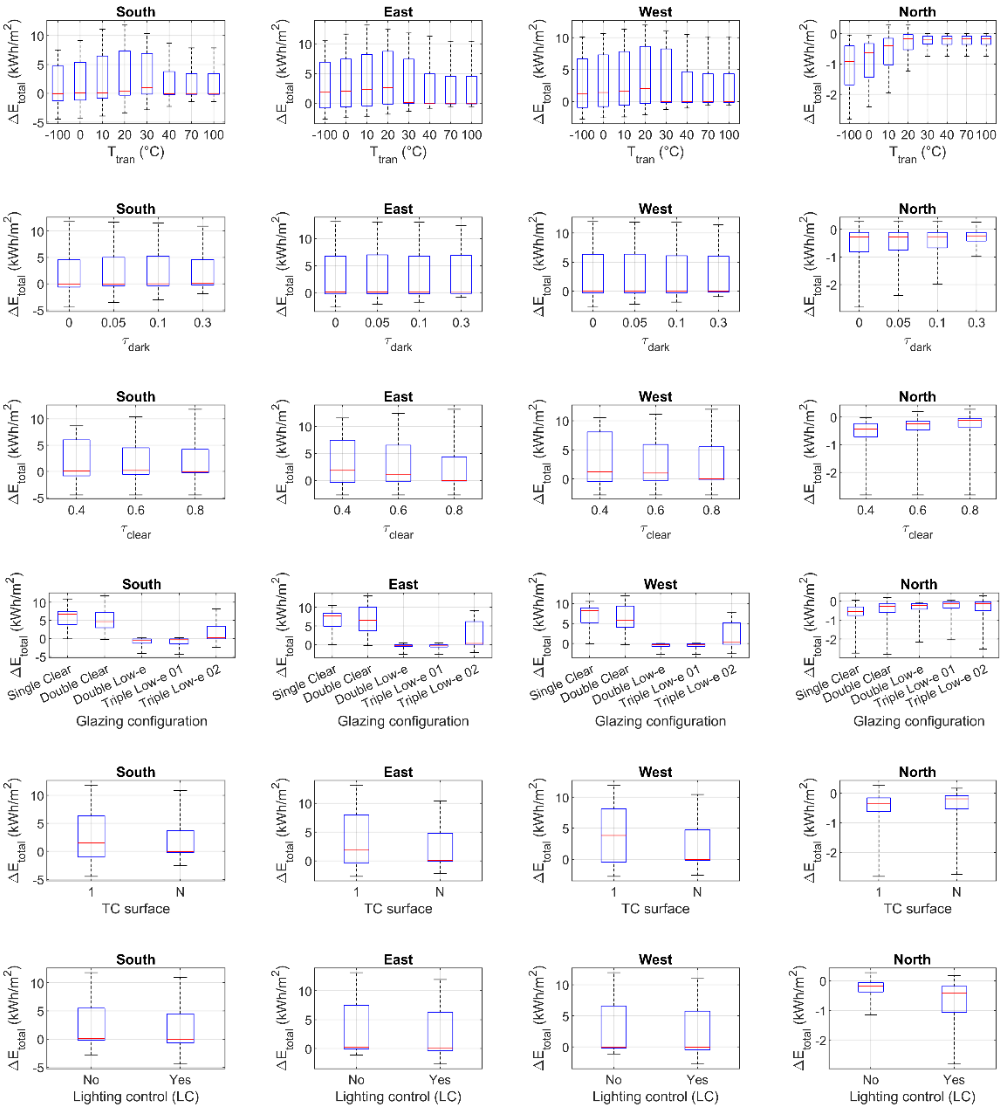

Supplementary Figure 16| Statistical analysis of the total site energy saving per conditioned floor area (ΔE_{total}) by TR windows with six variables (T_{tran}, τ_{dark}, τ_{clear}, glazing configuration, TR-applied surface, and lighting control) in four window orientations in Fairbanks, Alaska (climate classification: 8). In each box plot, the red central mark on each box indicates the median, and the bottom and top edges of the box indicate the 25th and 75th percentiles, respectively. The short black marks above and below each box indicate the maximum and minimum, respectively.

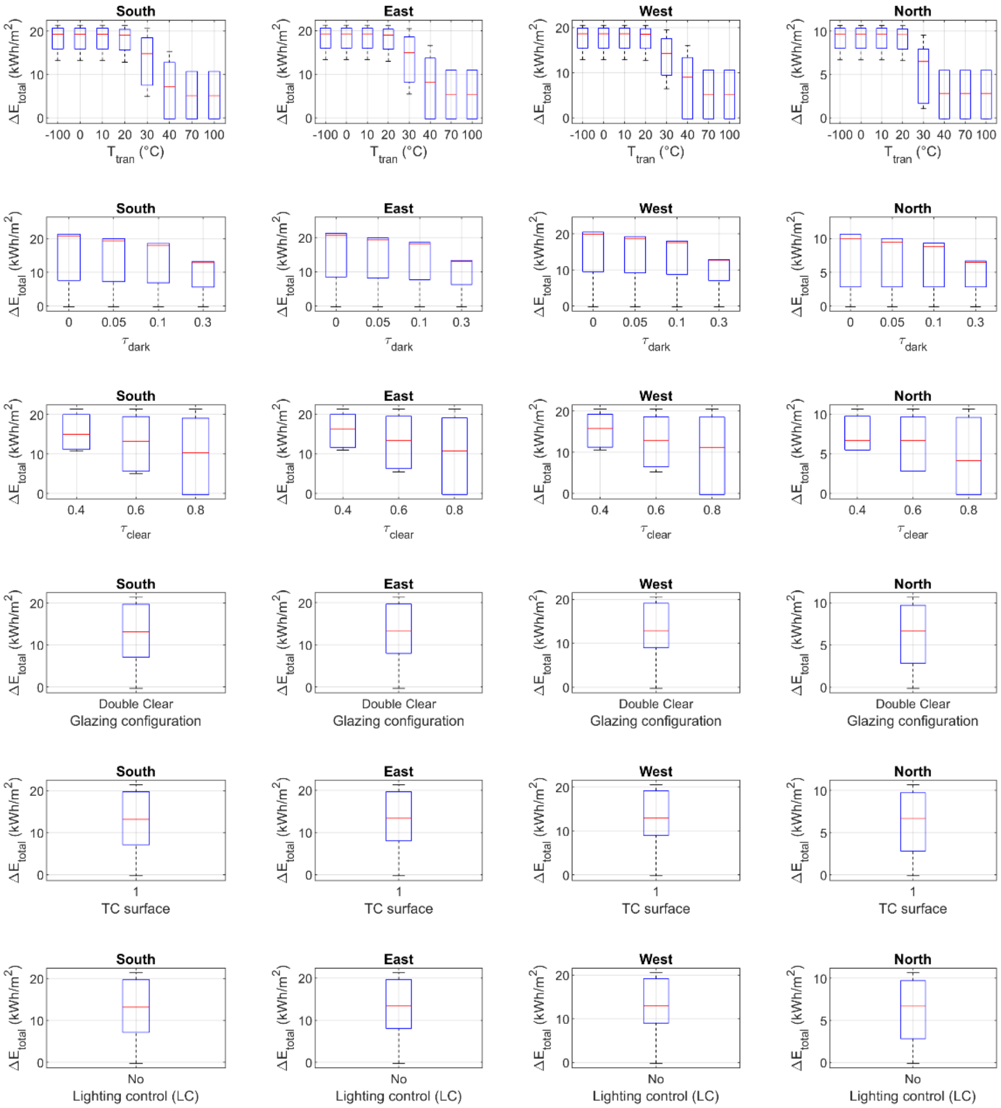

Supplementary Figure 17| Statistical analysis of the total site energy saving per conditioned floor area (ΔE_{total}) by TR windows with three variables (T_{tran}, τ_{dark}, and τ_{clear}) and three fixed parameters (glazing configuration (double clear), TR-applied surface (exterior), and **lighting control (No)**) in four window orientations in Miami, Florida (climate classification: 1A). In each box plot, the red central mark on each box indicates the median, and the bottom and top edges of the box indicate the 25th and 75th percentiles, respectively. The short black marks above and below each box indicate the maximum and minimum, respectively.

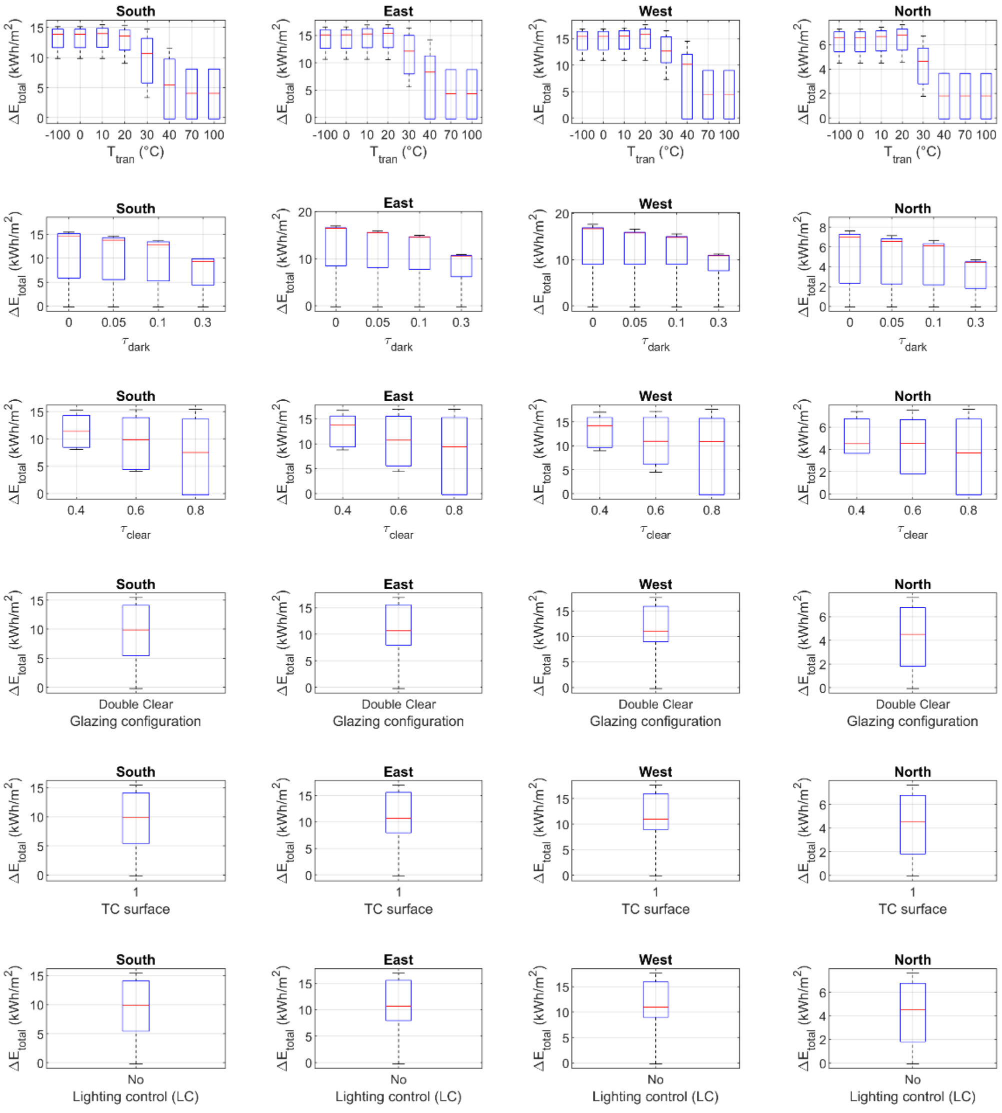

Supplementary Figure 18| Statistical analysis of the total site energy saving per conditioned floor area (ΔE_{total}) by TR windows with three variables (T_{tran}, τ_{dark}, and τ_{clear}) and three fixed parameters (glazing configuration (double clear), TR-applied surface (exterior), and lighting control (No)) in four window orientations in Houston, Texas (climate classification: 2A). In each box plot, the red central mark on each box indicates the median, and the bottom and top edges of the box indicate the 25th and 75th percentiles, respectively. The short black marks above and below each box indicate the maximum and minimum, respectively.

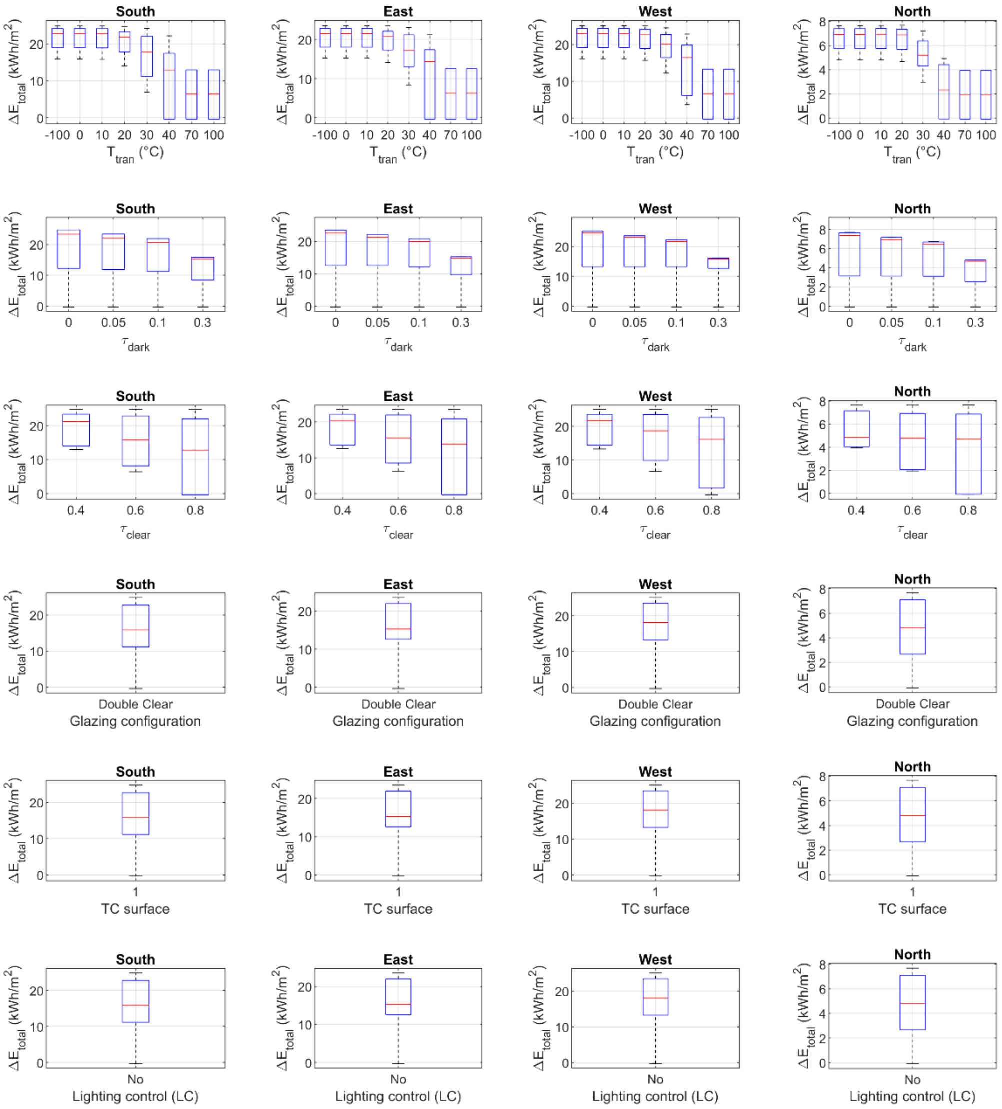

Supplementary Figure 19| Statistical analysis of the total site energy saving per conditioned floor area (ΔE_{total}) by TR windows with three variables (T_{tran}, τ_{dark}, and τ_{clear}) and three fixed parameters (glazing configuration (double clear), TR-applied surface (exterior), and lighting control (No)) in four window orientations in Phoenix, Arizona (climate classification: 2B). In each box plot, the red central mark on each box indicates the median, and the bottom and top edges of the box indicate the 25th and 75th percentiles, respectively. The short black marks above and below each box indicate the maximum and minimum, respectively.

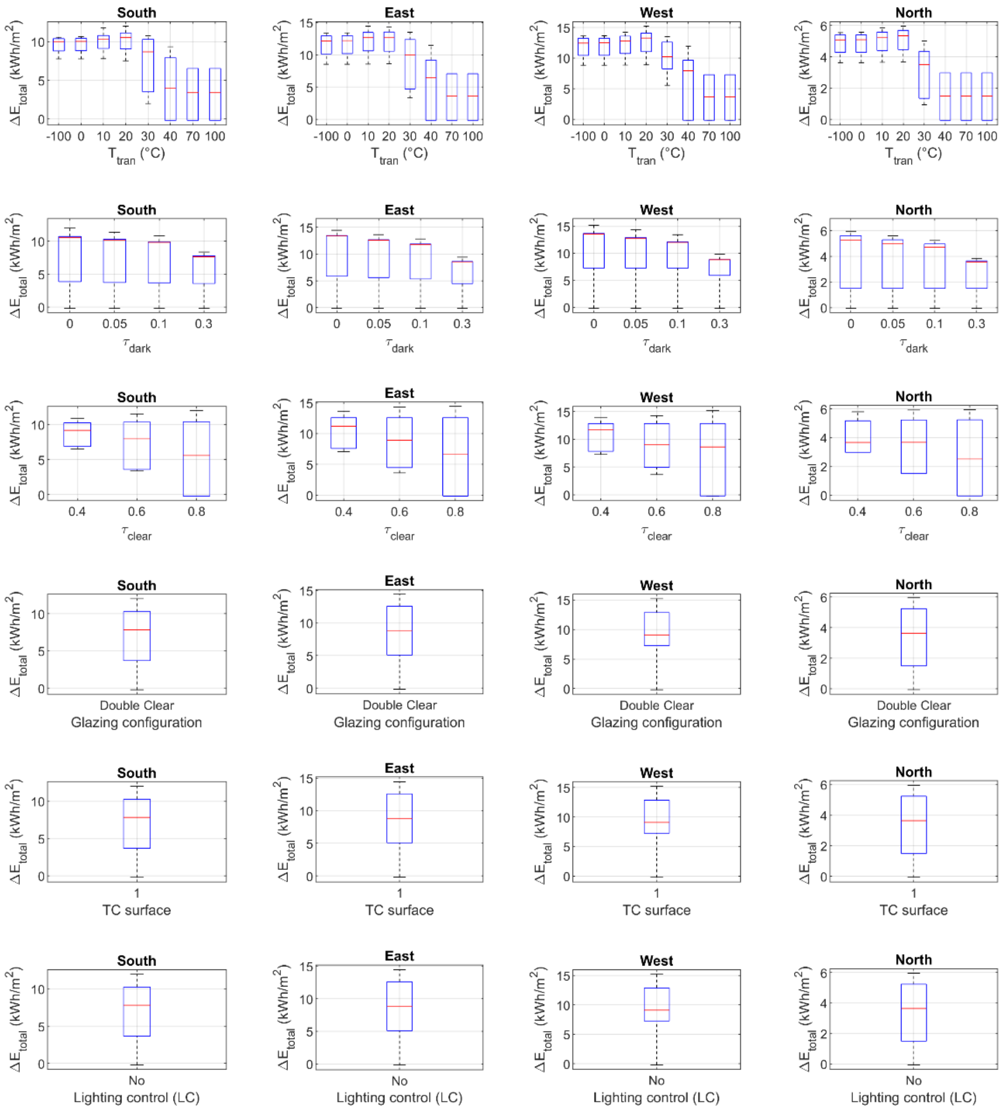

Supplementary Figure 20| Statistical analysis of the total site energy saving per conditioned floor area (ΔE_{total}) by TR windows with three variables (T_{tran}, τ_{dark}, and τ_{clear}) and three fixed parameters (glazing configuration (double clear), TR-applied surface (exterior), and lighting control (No)) in four window orientations in Atlanta, Georgia (climate classification: 3A). In each box plot, the red central mark on each box indicates the median, and the bottom and top edges of the box indicate the 25th and 75th percentiles, respectively. The short black marks above and below each box indicate the maximum and minimum, respectively.

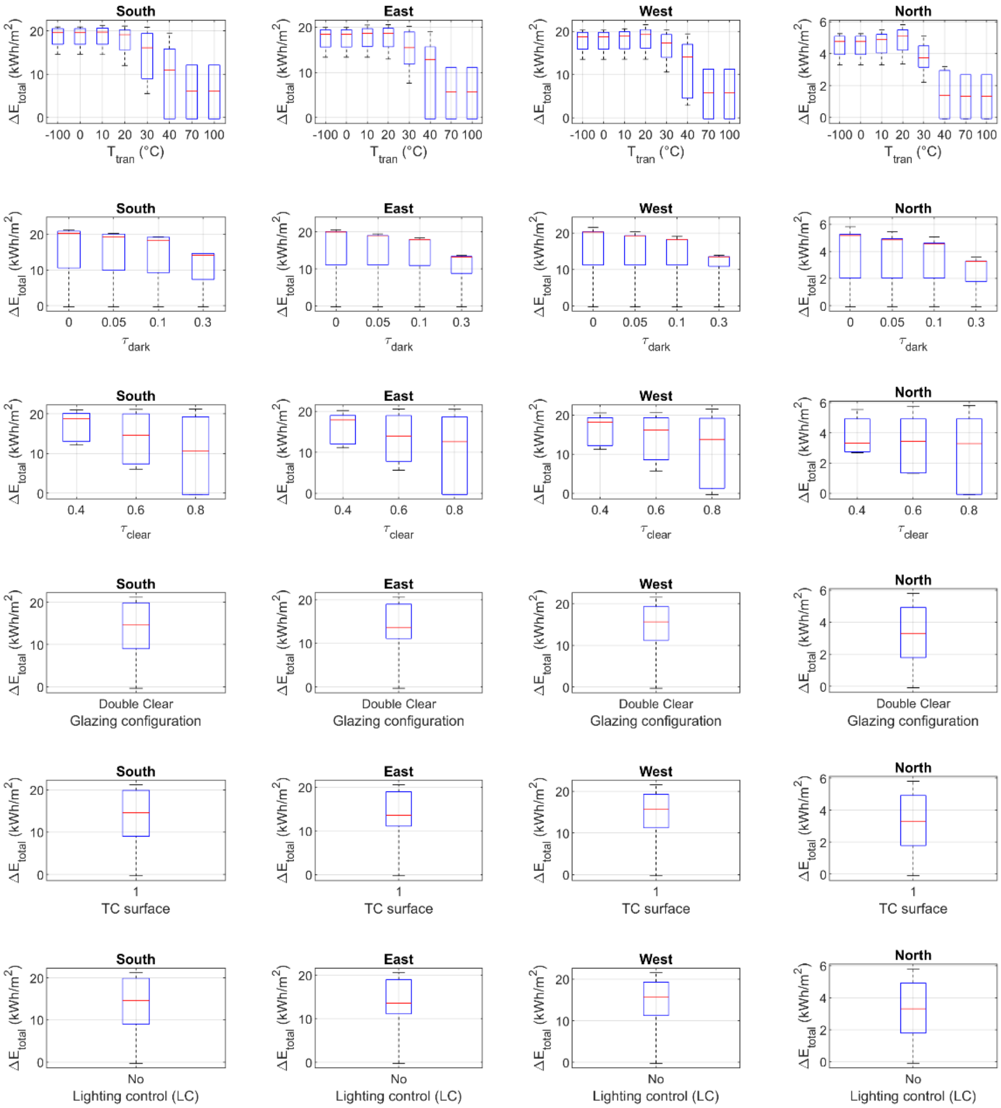

Supplementary Figure 21| Statistical analysis of the total site energy saving per conditioned floor area (ΔE_{total}) by TR windows with three variables (T_{tran}, τ_{dark}, and τ_{clear}) and three fixed parameters (glazing configuration (double clear), TR-applied surface (exterior), and lighting control (No)) in four window orientations in Las Vegas, Nevada (climate classification: 3B). In each box plot, the red central mark on each box indicates the median, and the bottom and top edges of the box indicate the 25th and 75th percentiles, respectively. The short black marks above and below each box indicate the maximum and minimum, respectively.

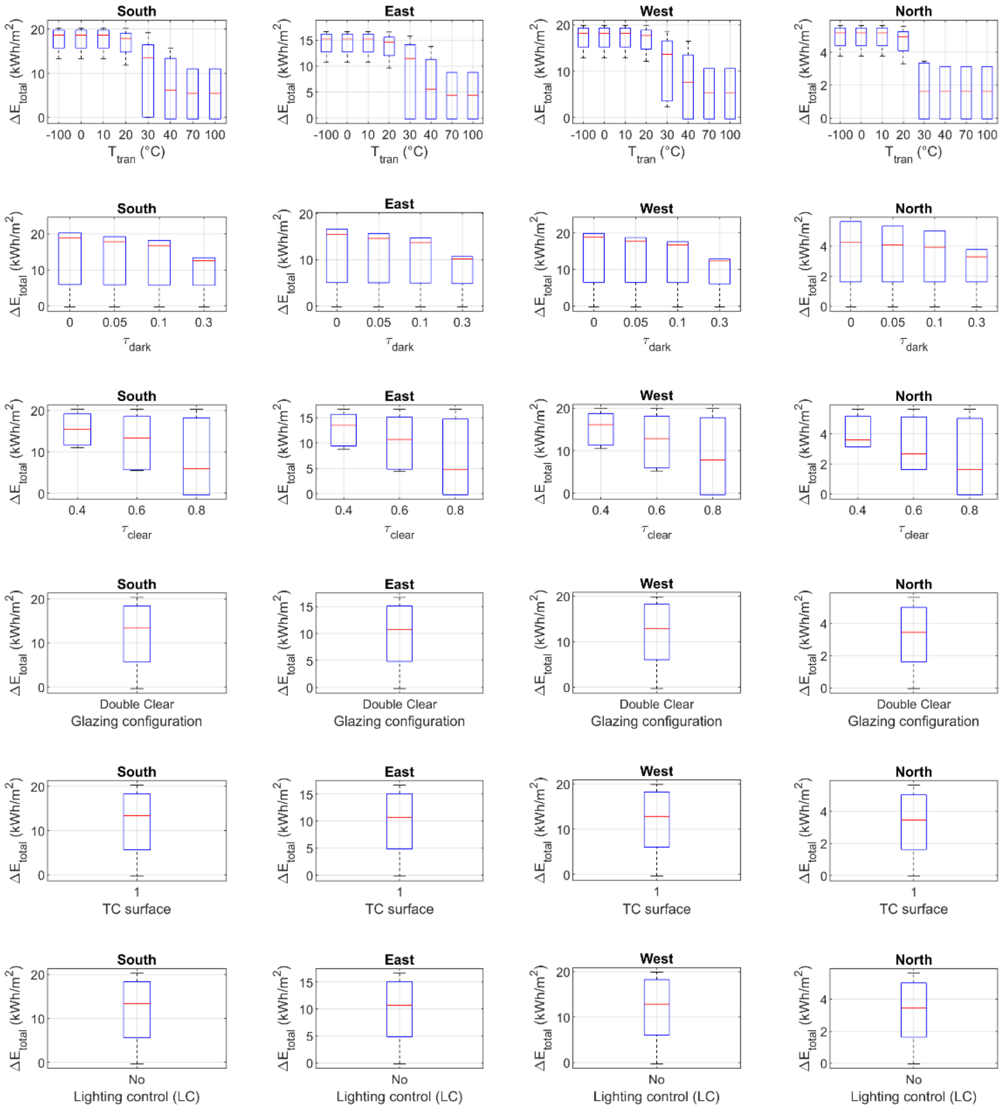

Supplementary Figure 22| Statistical analysis of the total site energy saving per conditioned floor area (ΔE_{total}) by TR windows with three variables (T_{tran}, τ_{dark}, and τ_{clear}) and three fixed parameters (glazing configuration (double clear), TR-applied surface (exterior), and lighting control (No)) in four window orientations in Los Angeles, California (climate classification: 3B). In each box plot, the red central mark on each box indicates the median, and the bottom and top edges of the box indicate the 25th and 75th percentiles, respectively. The short black marks above and below each box indicate the maximum and minimum, respectively.

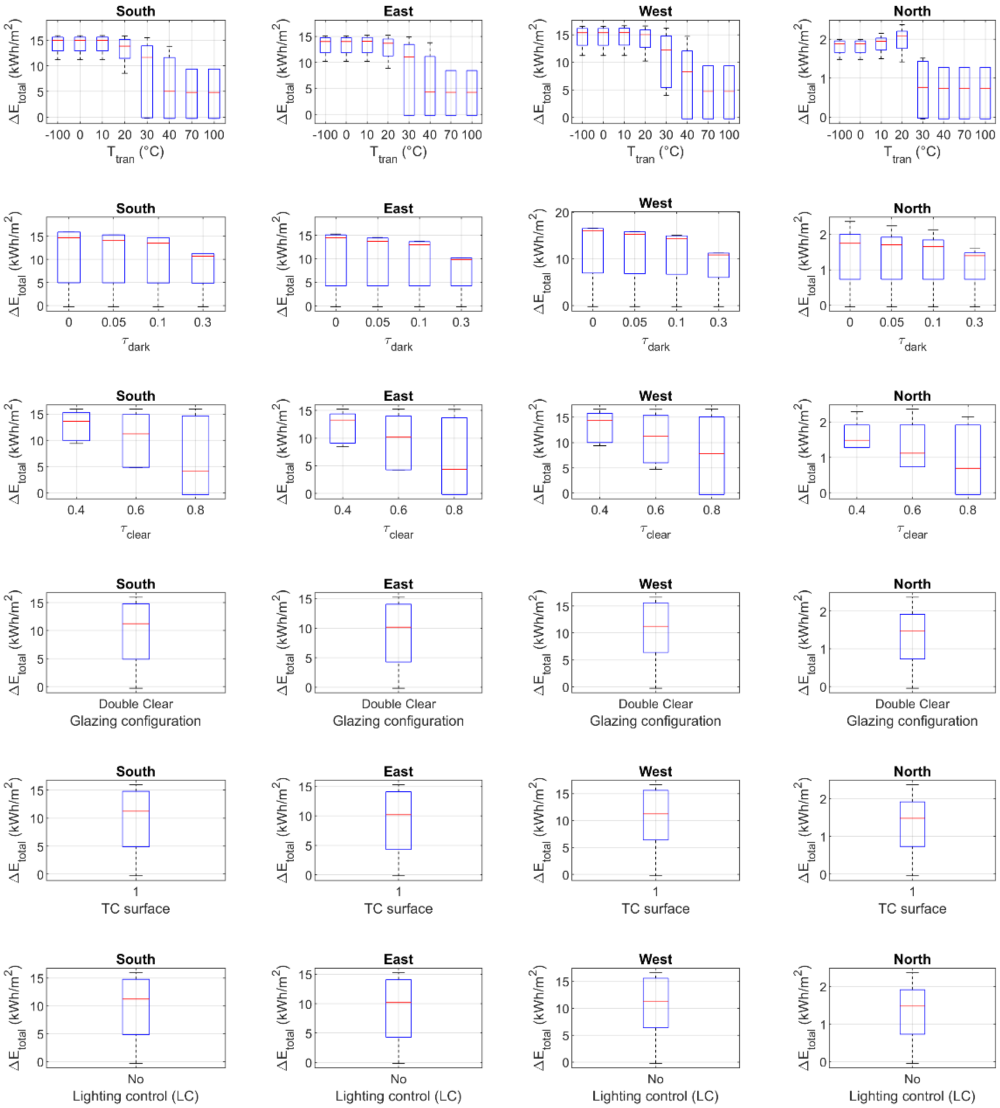

Supplementary Figure 23| Statistical analysis of the total site energy saving per conditioned floor area (ΔE_{total}) by TR windows with three variables (T_{tran}, τ_{dark}, and τ_{clear}) and three fixed parameters (glazing configuration (double clear), TR-applied surface (exterior), and lighting control (No)) in four window orientations in San Francisco, California (climate classification: 3C). In each box plot, the red central mark on each box indicates the median, and the bottom and top edges of the box indicate the 25th and 75th percentiles, respectively. The short black marks above and below each box indicate the maximum and minimum, respectively.

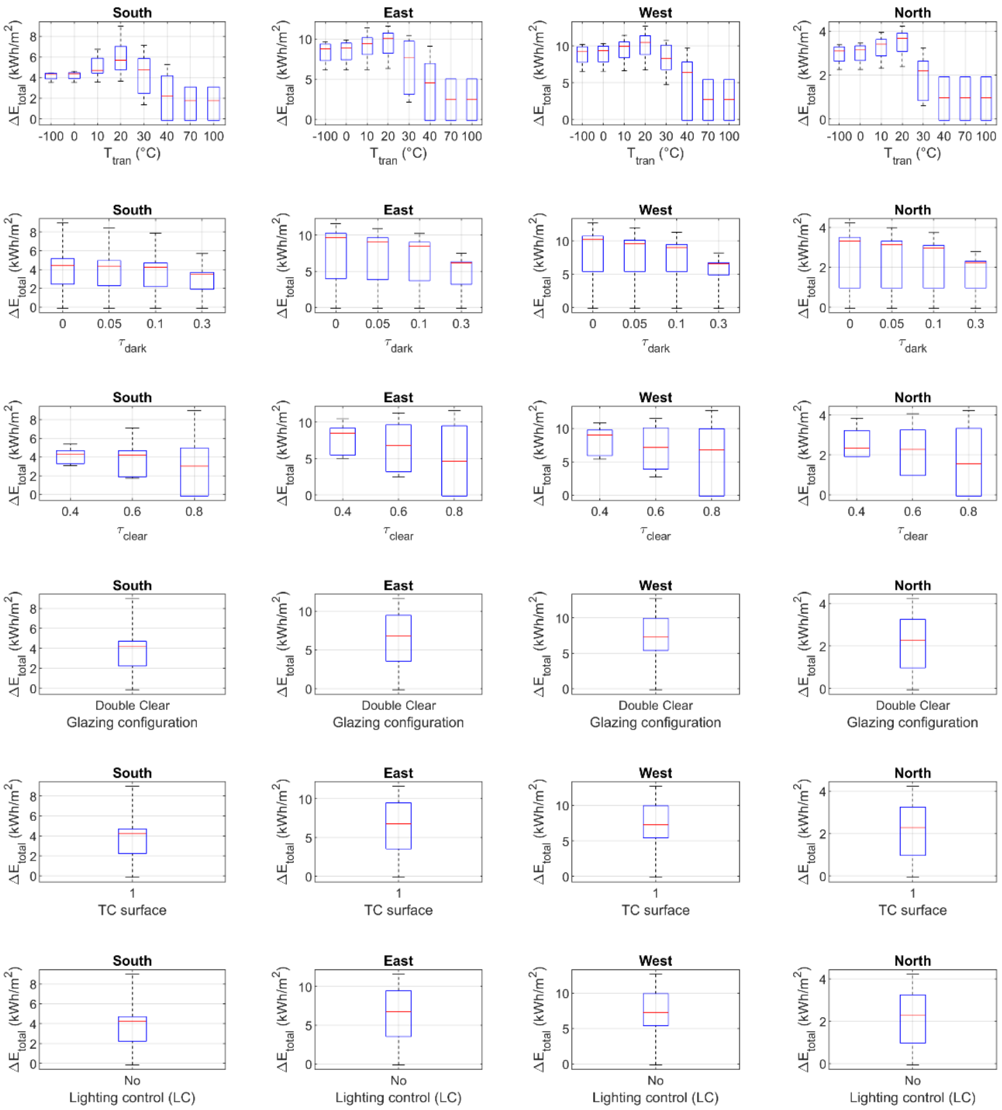

Supplementary Figure 24| Statistical analysis of the total site energy saving per conditioned floor area (ΔE_{total}) by TR windows with three variables (T_{tran}, τ_{dark}, and τ_{clear}) and three fixed parameters (glazing configuration (double clear), TR-applied surface (exterior), and lighting control (No)) in four window orientations in Baltimore, Maryland (climate classification: 4A). In each box plot, the red central mark on each box indicates the median, and the bottom and top edges of the box indicate the 25th and 75th percentiles, respectively. The short black marks above and below each box indicate the maximum and minimum, respectively.

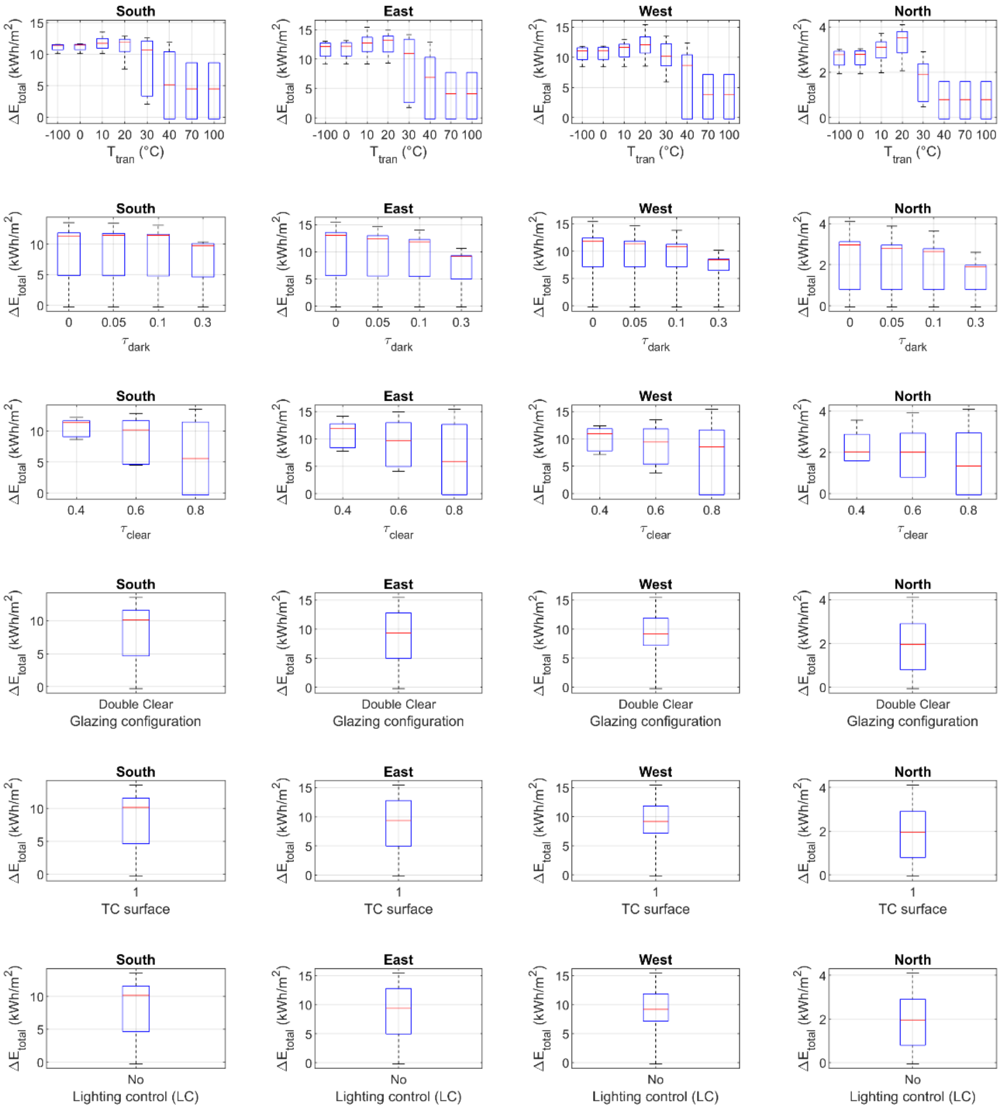

Supplementary Figure 25| Statistical analysis of the total site energy saving per conditioned floor area (ΔE_{total}) by TR windows with three variables (T_{tran}, τ_{dark}, and τ_{clear}) and three fixed parameters (glazing configuration (double clear), TR-applied surface (exterior), and lighting control (No)) in four window orientations in Albuquerque, New Mexico (climate classification: 4B). In each box plot, the red central mark on each box indicates the median, and the bottom and top edges of the box indicate the 25th and 75th percentiles, respectively. The short black marks above and below each box indicate the maximum and minimum, respectively.

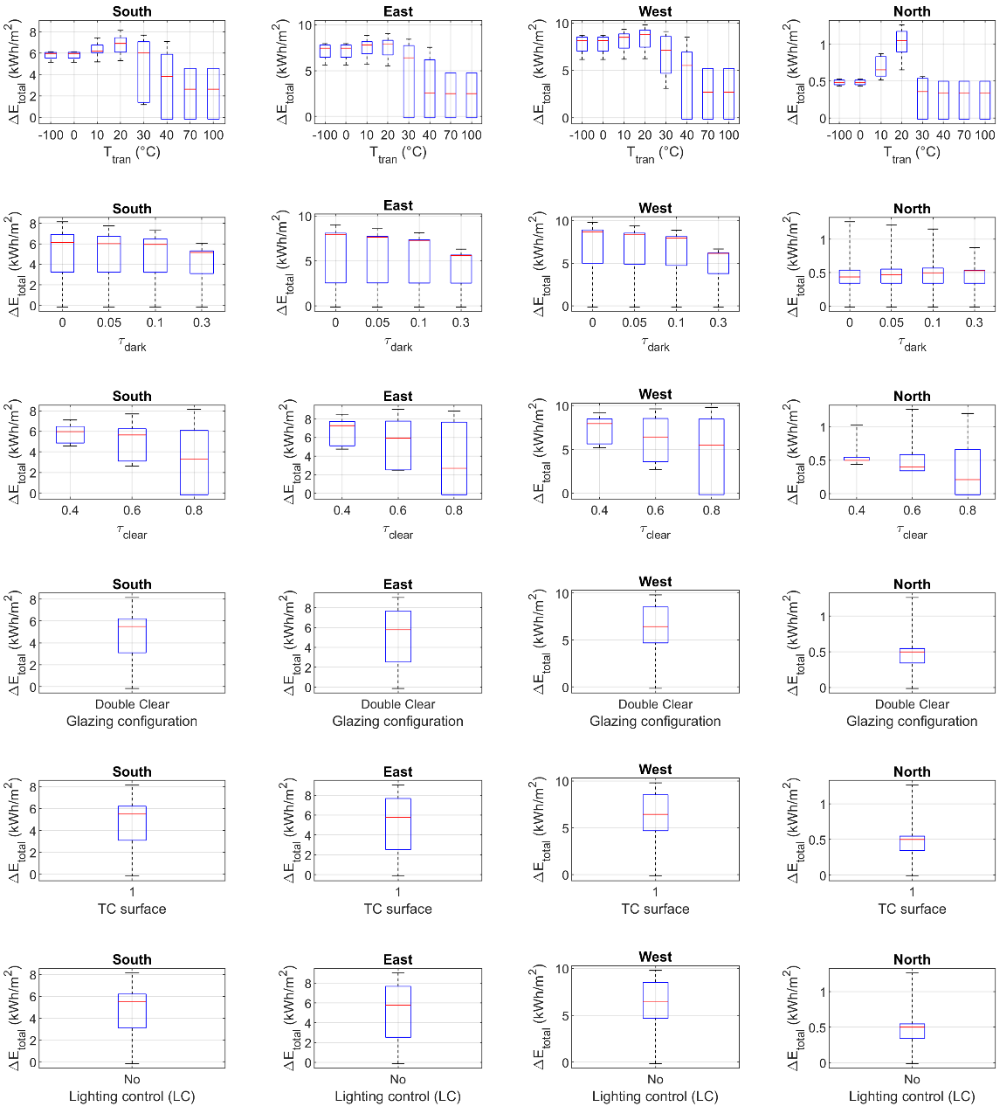

Supplementary Figure 26| Statistical analysis of the total site energy saving per conditioned floor area (ΔE_{total}) by TR windows with three variables (T_{tran}, τ_{dark}, and τ_{clear}) and three fixed parameters (glazing configuration (double clear), TR-applied surface (exterior), and lighting control (No)) in four window orientations in Seattle, Washington (climate classification: 4C). In each box plot, the red central mark on each box indicates the median, and the bottom and top edges of the box indicate the 25th and 75th percentiles, respectively. The short black marks above and below each box indicate the maximum and minimum, respectively.

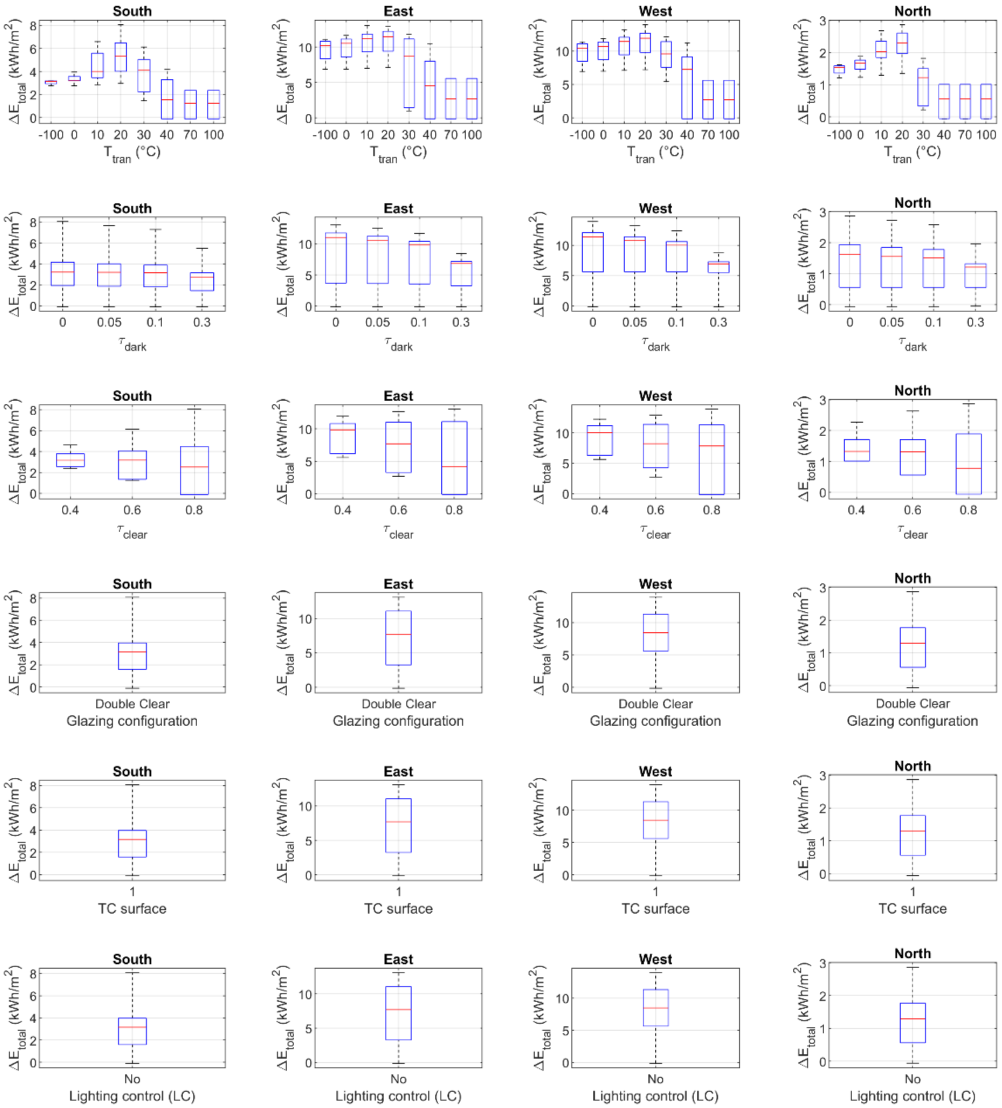

Supplementary Figure 27| Statistical analysis of the total site energy saving per conditioned floor area (ΔE_{total}) by TR windows with three variables (T_{tran}, τ_{dark}, and τ_{clear}) and three fixed parameters (glazing configuration (double clear), TR-applied surface (exterior), and lighting control (No)) in four window orientations in Chicago, Illinois (climate classification: 5A). In each box plot, the red central mark on each box indicates the median, and the bottom and top edges of the box indicate the 25th and 75th percentiles, respectively. The short black marks above and below each box indicate the maximum and minimum, respectively.

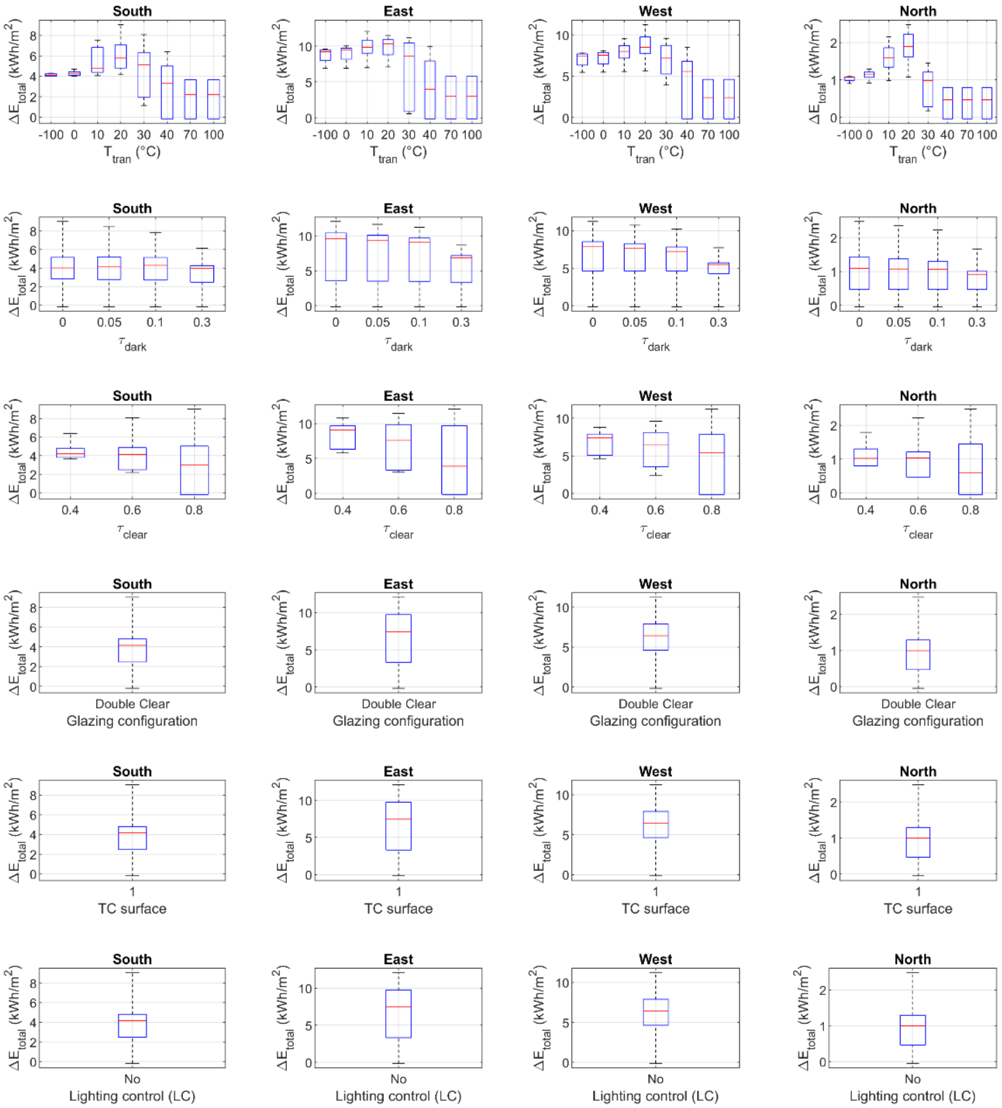

Supplementary Figure 28| Statistical analysis of the total site energy saving per conditioned floor area (ΔE_{total}) by TR windows with three variables (T_{tran}, τ_{dark}, and τ_{clear}) and three fixed parameters (glazing configuration (double clear), TR-applied surface (exterior), and lighting control (No)) in four window orientations in Boulder, Colorado (climate classification: 5B). In each box plot, the red central mark on each box indicates the median, and the bottom and top edges of the box indicate the 25th and 75th percentiles, respectively. The short black marks above and below each box indicate the maximum and minimum, respectively.

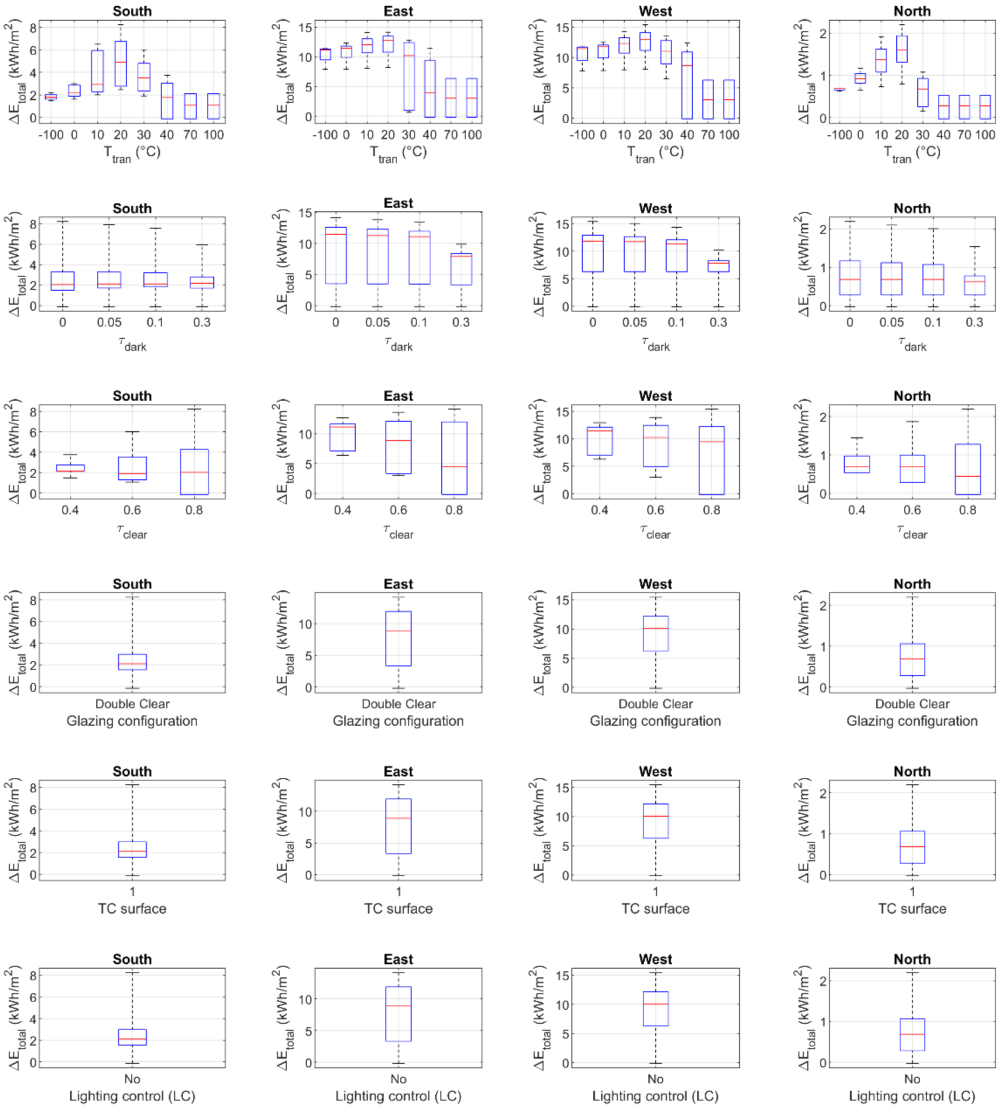

Supplementary Figure 29| Statistical analysis of the total site energy saving per conditioned floor area (ΔE_{total}) by TR windows with three variables (T_{tran}, τ_{dark}, and τ_{clear}) and three fixed parameters (glazing configuration (double clear), TR-applied surface (exterior), and lighting control (No)) in four window orientations in Minneapolis, Minnesota (climate classification: 6A). In each box plot, the red central mark on each box indicates the median, and the bottom and top edges of the box indicate the 25th and 75th percentiles, respectively. The short black marks above and below each box indicate the maximum and minimum, respectively.

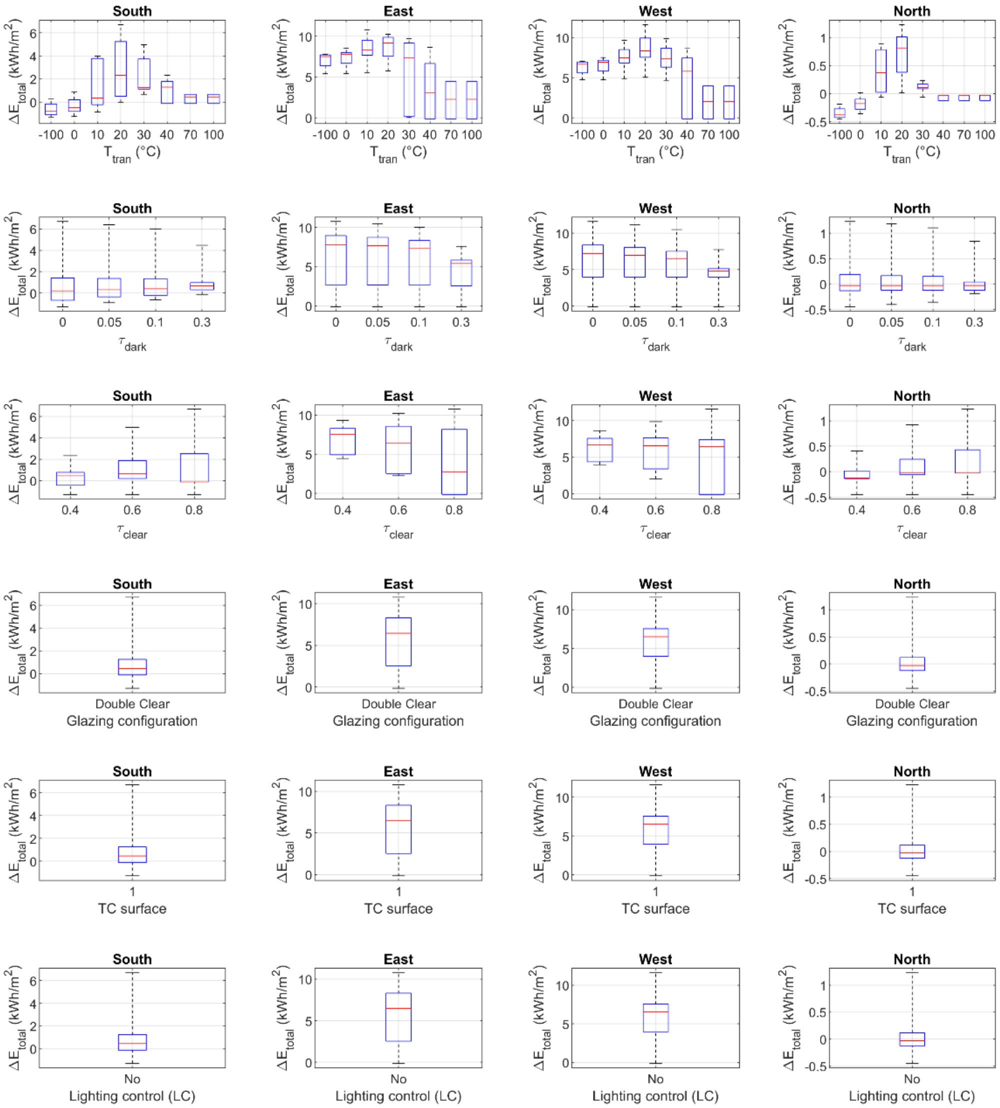

Supplementary Figure 30| Statistical analysis of the total site energy saving per conditioned floor area (ΔE_{total}) by TR windows with three variables (T_{tran}, τ_{dark}, and τ_{clear}) and three fixed parameters (glazing configuration (double clear), TR-applied surface (exterior), and lighting control (No)) in four window orientations in Helena, Montana (climate classification: 6B). In each box plot, the red central mark on each box indicates the median, and the bottom and top edges of the box indicate the 25th and 75th percentiles, respectively. The short black marks above and below each box indicate the maximum and minimum, respectively.

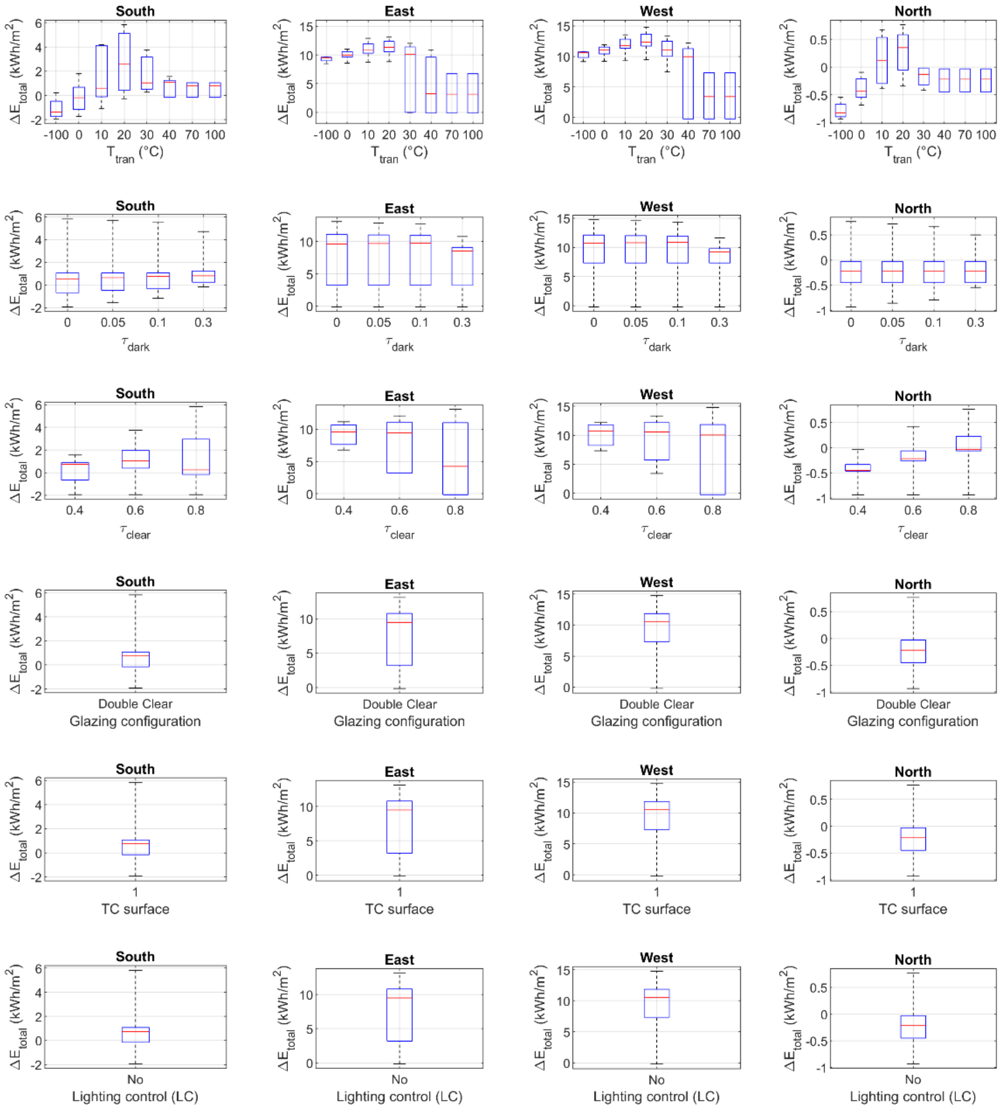

Supplementary Figure 31| Statistical analysis of the total site energy saving per conditioned floor area (ΔE_{total}) by TR windows with three variables (T_{tran}, τ_{dark}, and τ_{clear}) and three fixed parameters (glazing configuration (double clear), TR-applied surface (exterior), and lighting control (No)) in four window orientations in Duluth, Minnesota (climate classification: 7). In each box plot, the red central mark on each box indicates the median, and the bottom and top edges of the box indicate the 25th and 75th percentiles, respectively. The short black marks above and below each box indicate the maximum and minimum, respectively.

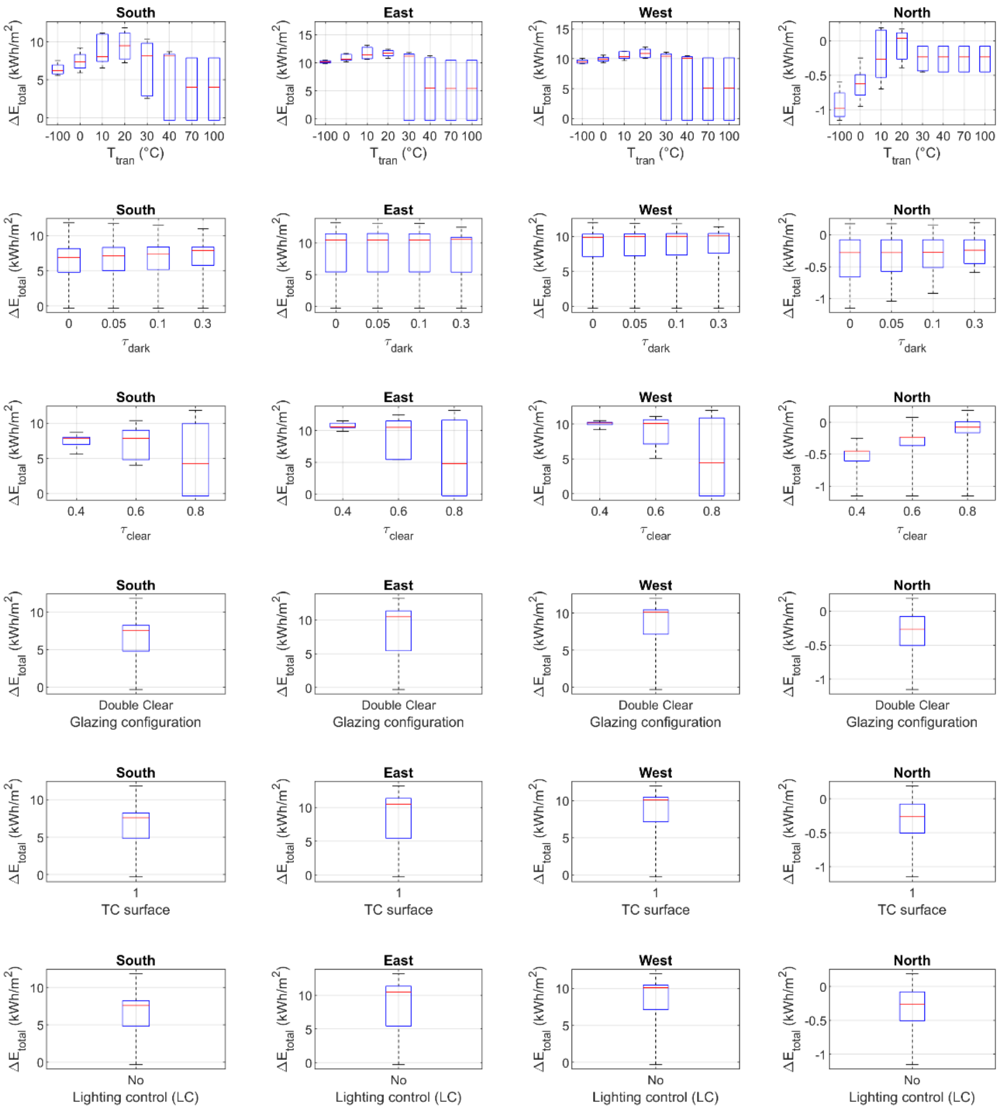

Supplementary Figure 32| Statistical analysis of the total site energy saving per conditioned floor area (ΔE_{total}) by TR windows with three variables (T_{tran}, τ_{dark}, and τ_{clear}) and three fixed parameters (glazing configuration (double clear), TR-applied surface (exterior), and lighting control (No)) in four window orientations in Fairbanks, Alaska (climate classification: 8). In each box plot, the red central mark on each box indicates the median, and the bottom and top edges of the box indicate the 25th and 75th percentiles, respectively. The short black marks above and below each box indicate the maximum and minimum, respectively.

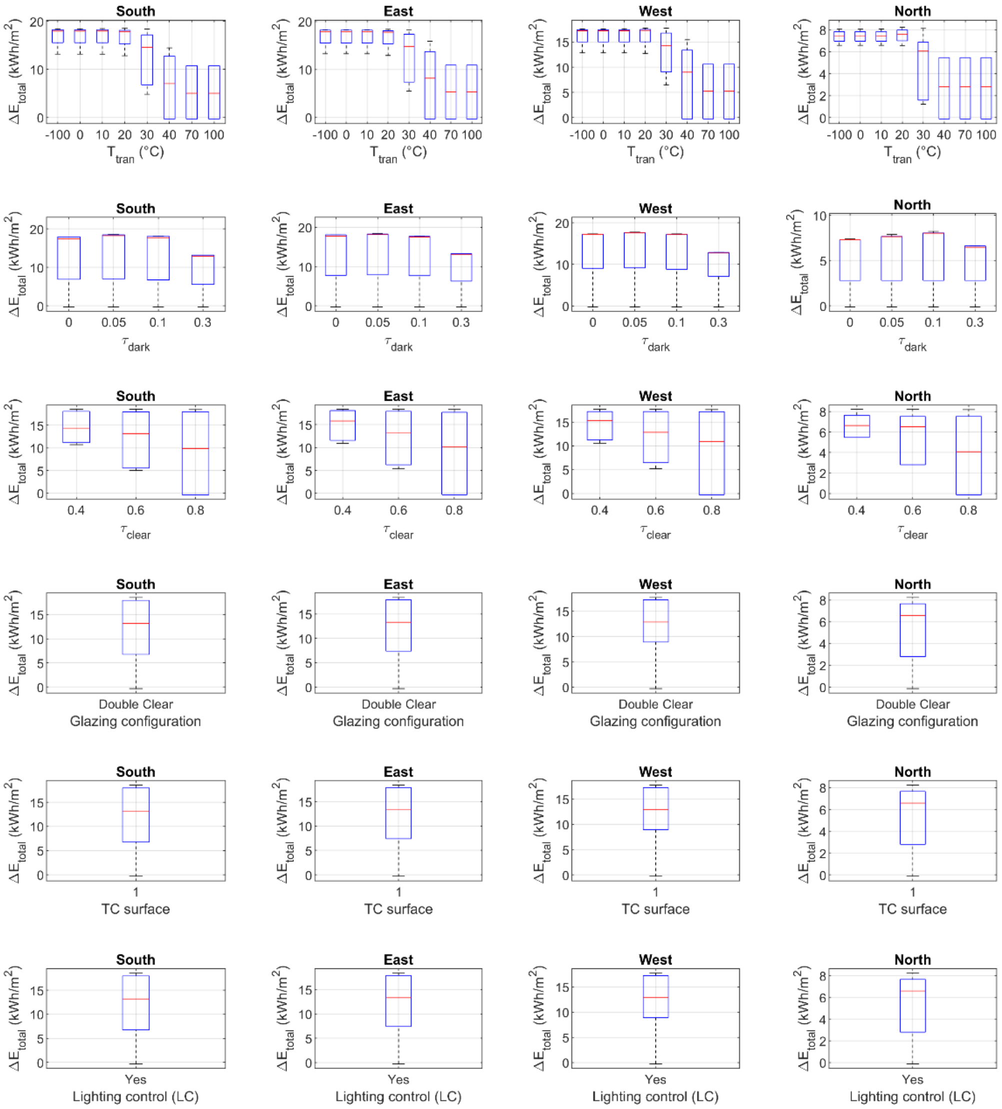

Supplementary Figure 33| Statistical analysis of the total site energy saving per conditioned floor area (ΔE_{total}) by TR windows with three variables (T_{tran}, τ_{dark}, and τ_{clear}) and three fixed parameters (glazing configuration (double clear), TR-applied surface (exterior), and lighting control (Yes)) in four window orientations in Miami, Florida (climate classification: 1A). In each box plot, the red central mark on each box indicates the median, and the bottom and top edges of the box indicate the 25th and 75th percentiles, respectively. The short black marks above and below each box indicate the maximum and minimum, respectively.

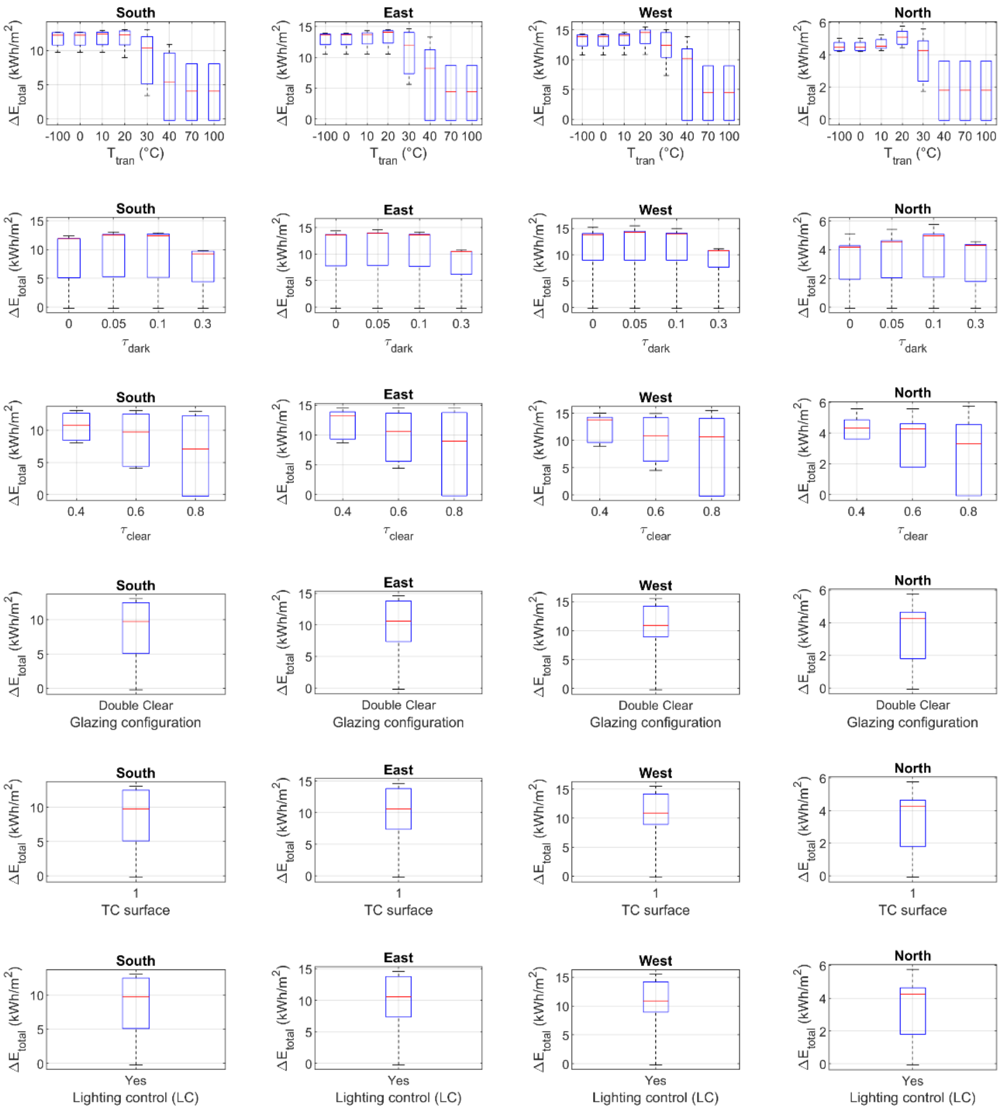

Supplementary Figure 34| Statistical analysis of the total site energy saving per conditioned floor area (ΔE_{total}) by TR windows with three variables (T_{tran}, τ_{dark}, and τ_{clear}) and three fixed parameters (glazing configuration (double clear), TR-applied surface (exterior), and lighting control (Yes)) in four window orientations in Houston, Texas (climate classification: 2A). In each box plot, the red central mark on each box indicates the median, and the bottom and top edges of the box indicate the 25th and 75th percentiles, respectively. The short black marks above and below each box indicate the maximum and minimum, respectively.

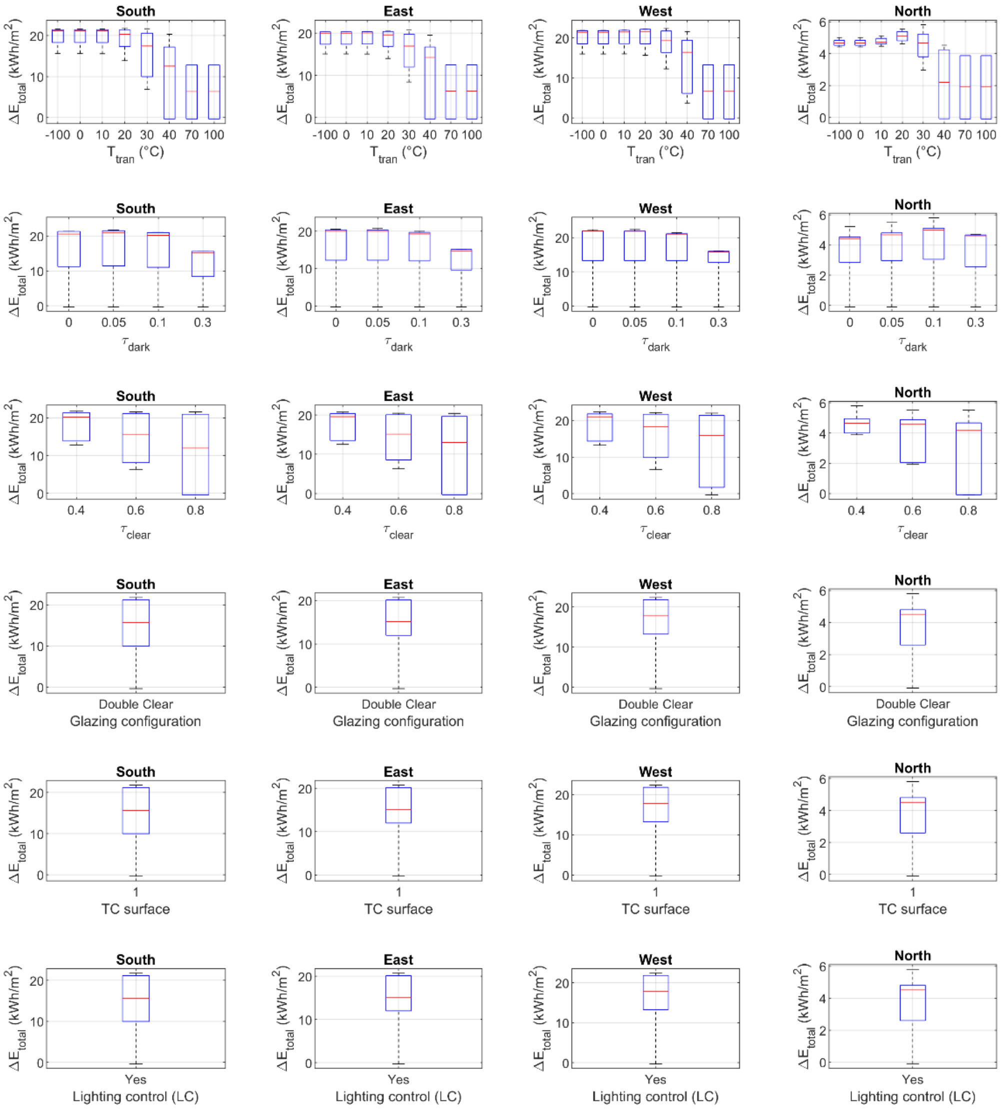

Supplementary Figure 35| Statistical analysis of the total site energy saving per conditioned floor area (ΔE_{total}) by TR windows with three variables (T_{tran}, τ_{dark}, and τ_{clear}) and three fixed parameters (glazing configuration (double clear), TR-applied surface (exterior), and lighting control (Yes)) in four window orientations in Phoenix, Arizona (climate classification: 2B). In each box plot, the red central mark on each box indicates the median, and the bottom and top edges of the box indicate the 25th and 75th percentiles, respectively. The short black marks above and below each box indicate the maximum and minimum, respectively.

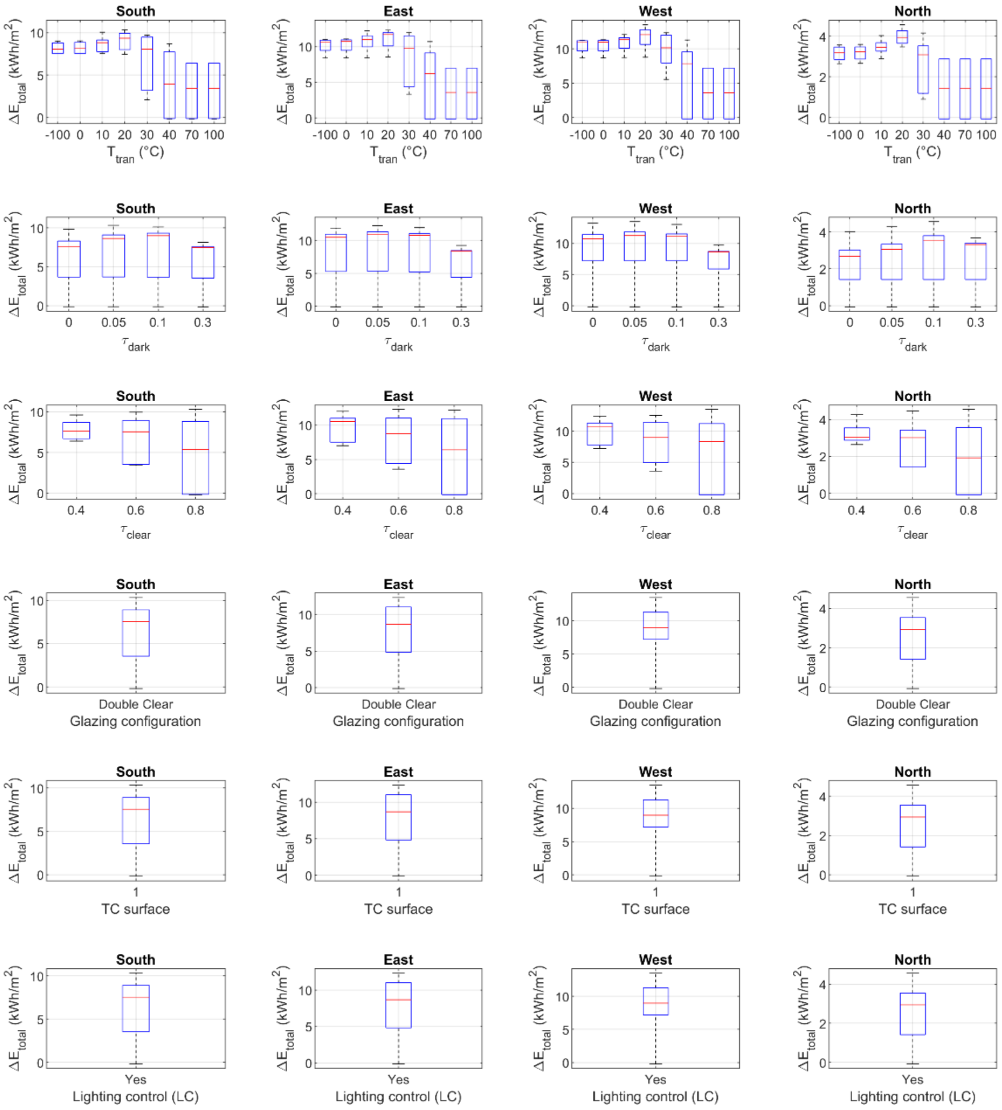

Supplementary Figure 36| Statistical analysis of the total site energy saving per conditioned floor area (ΔE_{total}) by TR windows with three variables (T_{tran}, τ_{dark}, and τ_{clear}) and three fixed parameters (glazing configuration (double clear), TR-applied surface (exterior), and lighting control (Yes)) in four window orientations in Atlanta, Georgia (climate classification: 3A). In each box plot, the red central mark on each box indicates the median, and the bottom and top edges of the box indicate the 25th and 75th percentiles, respectively. The short black marks above and below each box indicate the maximum and minimum, respectively.

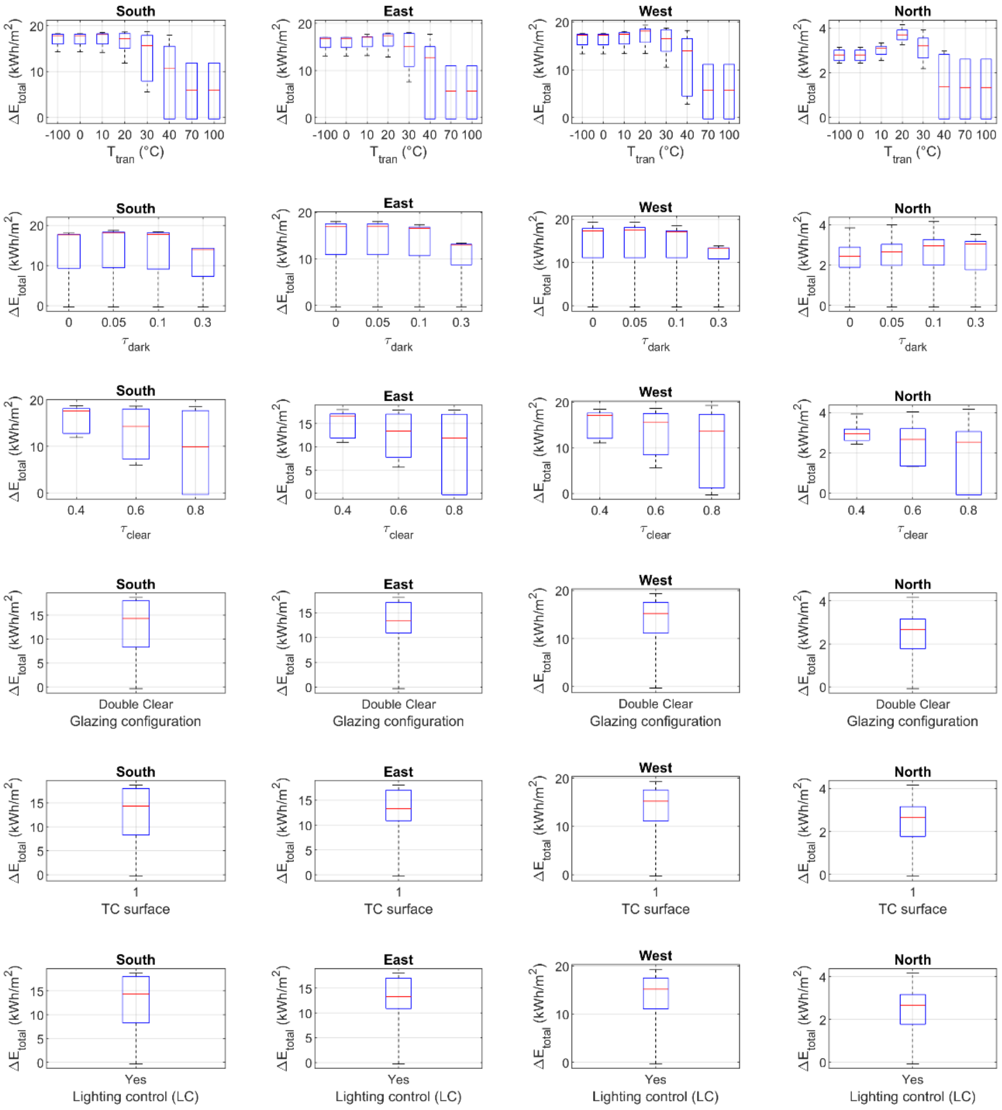

Supplementary Figure 37| Statistical analysis of the total site energy saving per conditioned floor area (ΔE_{total}) by TR windows with three variables (T_{tran}, τ_{dark}, and τ_{clear}) and three fixed parameters (glazing configuration (double clear), TR-applied surface (exterior), and lighting control (Yes)) in four window orientations in Las Vegas, Nevada (climate classification: 3B). In each box plot, the red central mark on each box indicates the median, and the bottom and top edges of the box indicate the 25th and 75th percentiles, respectively. The short black marks above and below each box indicate the maximum and minimum, respectively.

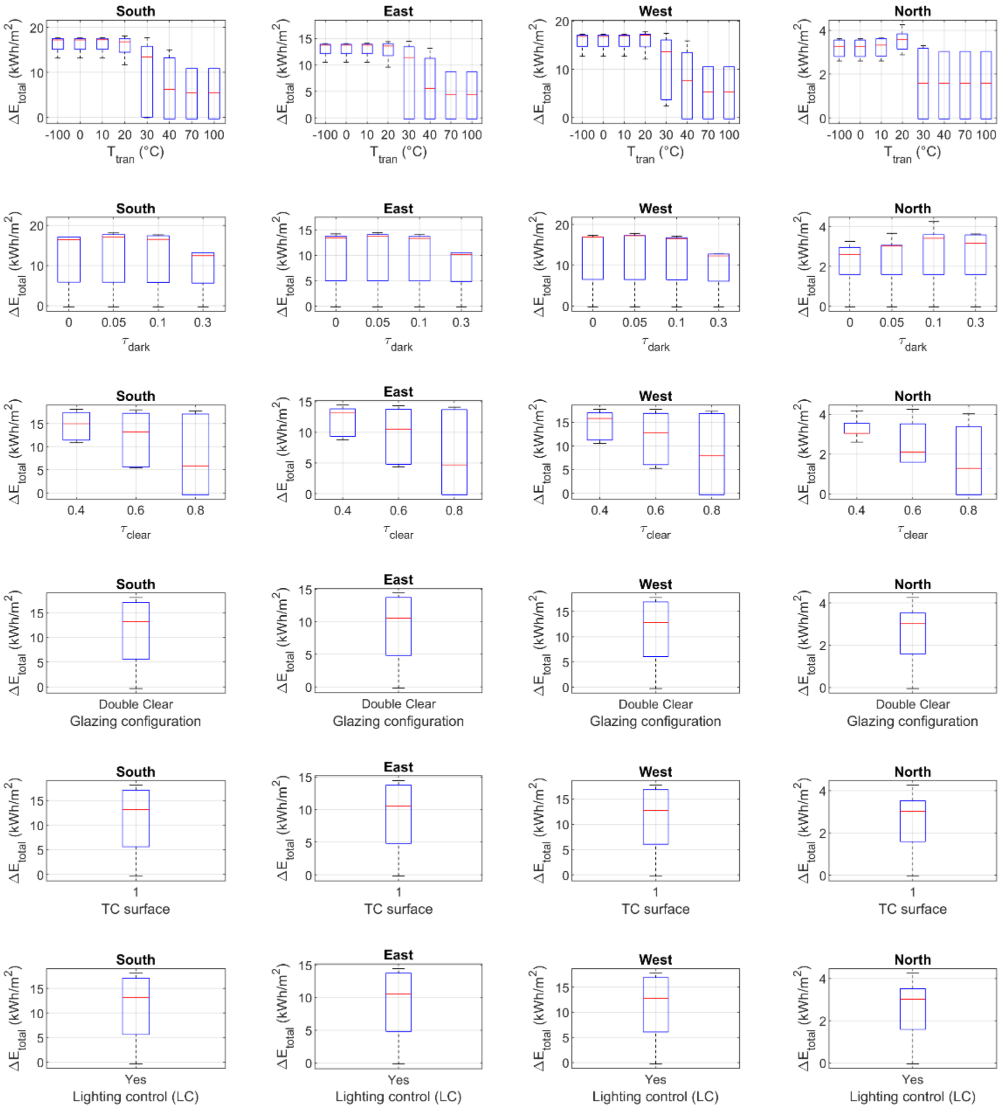

Supplementary Figure 38| Statistical analysis of the total site energy saving per conditioned floor area (ΔE_{total}) by TR windows with three variables (T_{tran}, τ_{dark}, and τ_{clear}) and three fixed parameters (glazing configuration (double clear), TR-applied surface (exterior), and lighting control (Yes)) in four window orientations in Los Angeles, California (climate classification: 3B). In each box plot, the red central mark on each box indicates the median, and the bottom and top edges of the box indicate the 25th and 75th percentiles, respectively. The short black marks above and below each box indicate the maximum and minimum, respectively.

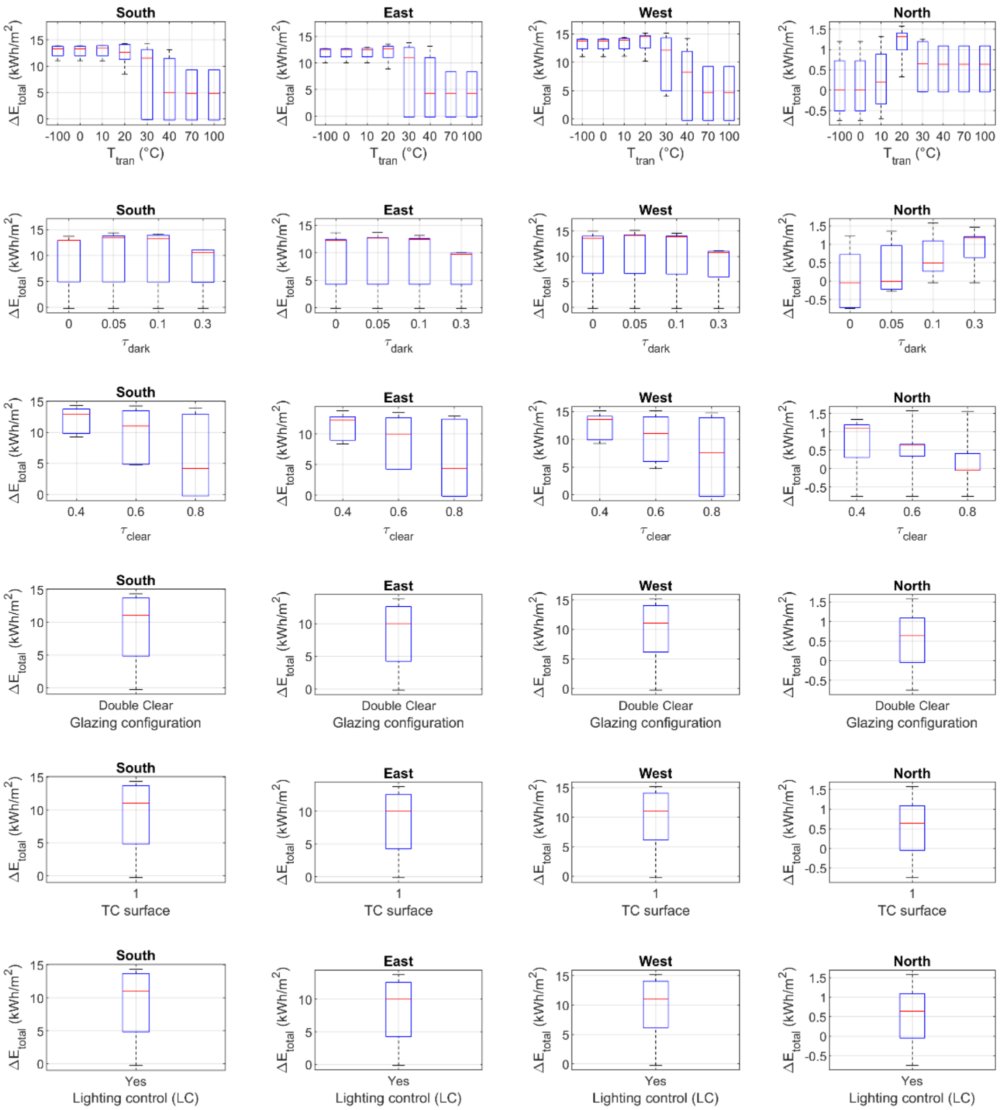

Supplementary Figure 39| Statistical analysis of the total site energy saving per conditioned floor area (ΔE_{total}) by TR windows with three variables (T_{tran}, τ_{dark}, and τ_{clear}) and three fixed parameters (glazing configuration (double clear), TR-applied surface (exterior), and lighting control (Yes)) in four window orientations in San Francisco, California (climate classification: 3C). In each box plot, the red central mark on each box indicates the median, and the bottom and top edges of the box indicate the 25th and 75th percentiles, respectively. The short black marks above and below each box indicate the maximum and minimum, respectively.

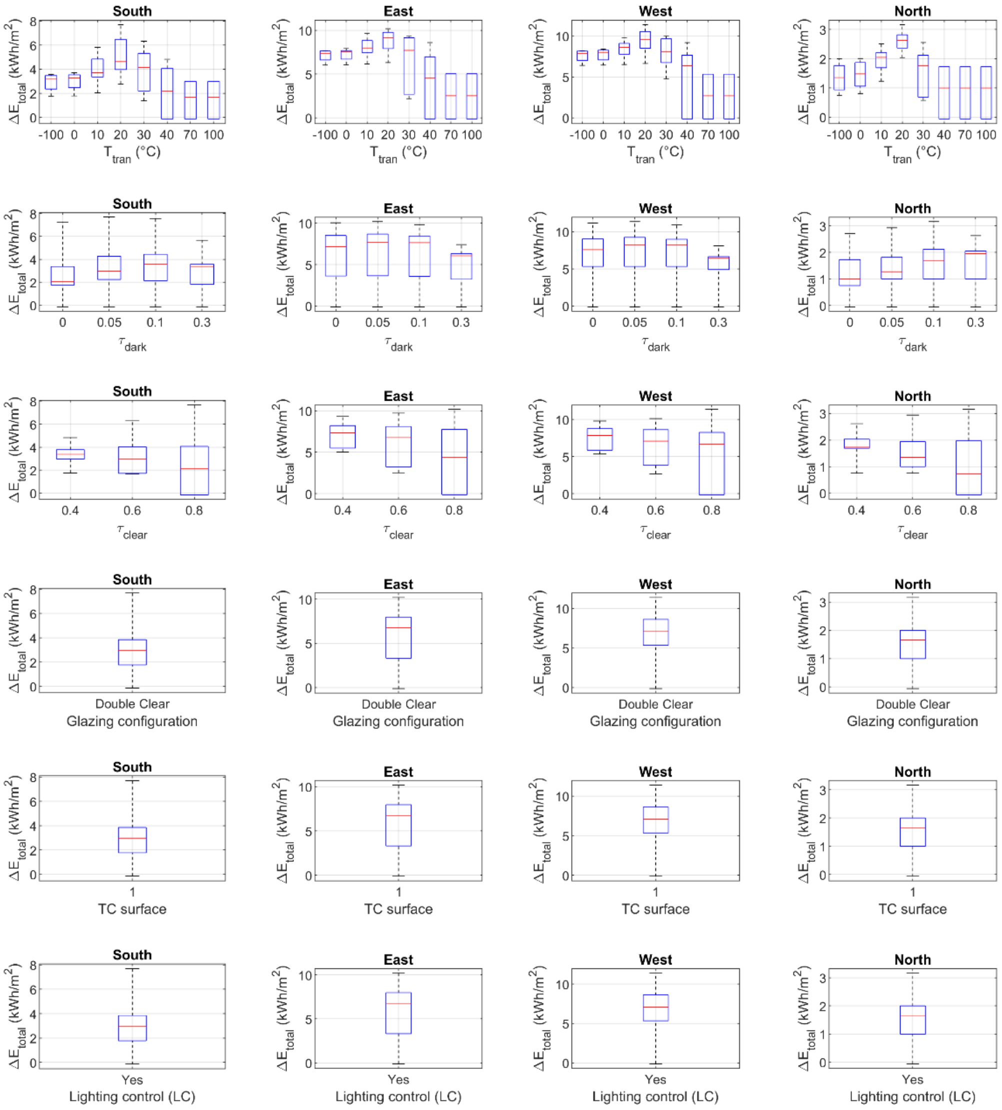

Supplementary Figure 40| Statistical analysis of the total site energy saving per conditioned floor area (ΔE_{total}) by TR windows with three variables (T_{tran}, τ_{dark}, and τ_{clear}) and three fixed parameters (glazing configuration (double clear), TR-applied surface (exterior), and lighting control (Yes)) in four window orientations in Baltimore, Maryland (climate classification: 4A). In each box plot, the red central mark on each box indicates the median, and the bottom and top edges of the box indicate the 25th and 75th percentiles, respectively. The short black marks above and below each box indicate the maximum and minimum, respectively.

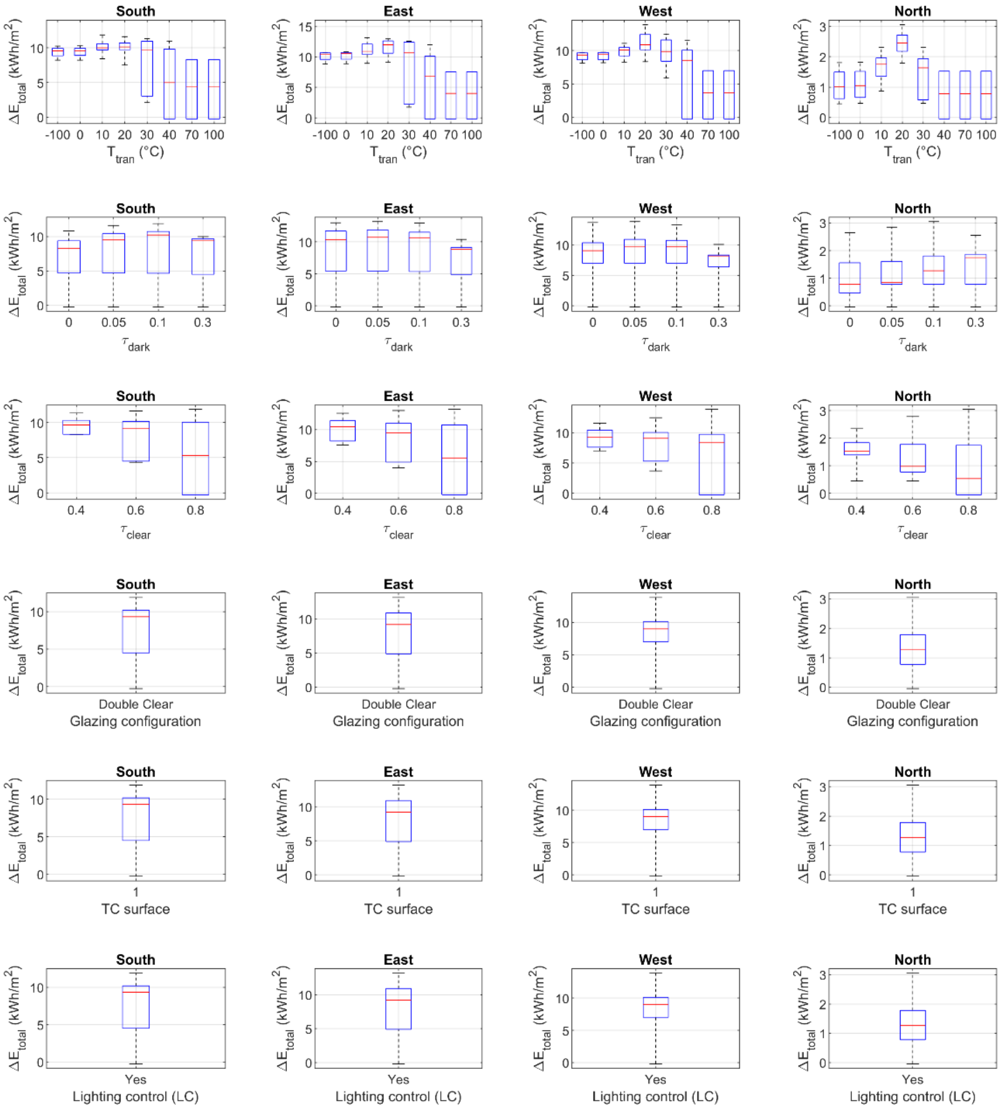

Supplementary Figure 41| Statistical analysis of the total site energy saving per conditioned floor area (ΔE_{total}) by TR windows with three variables (T_{tran}, τ_{dark}, and τ_{clear}) and three fixed parameters (glazing configuration (double clear), TR-applied surface (exterior), and lighting control (Yes)) in four window orientations in Albuquerque, New Mexico (climate classification: 4B). In each box plot, the red central mark on each box indicates the median, and the bottom and top edges of the box indicate the 25th and 75th percentiles, respectively. The short black marks above and below each box indicate the maximum and minimum, respectively.

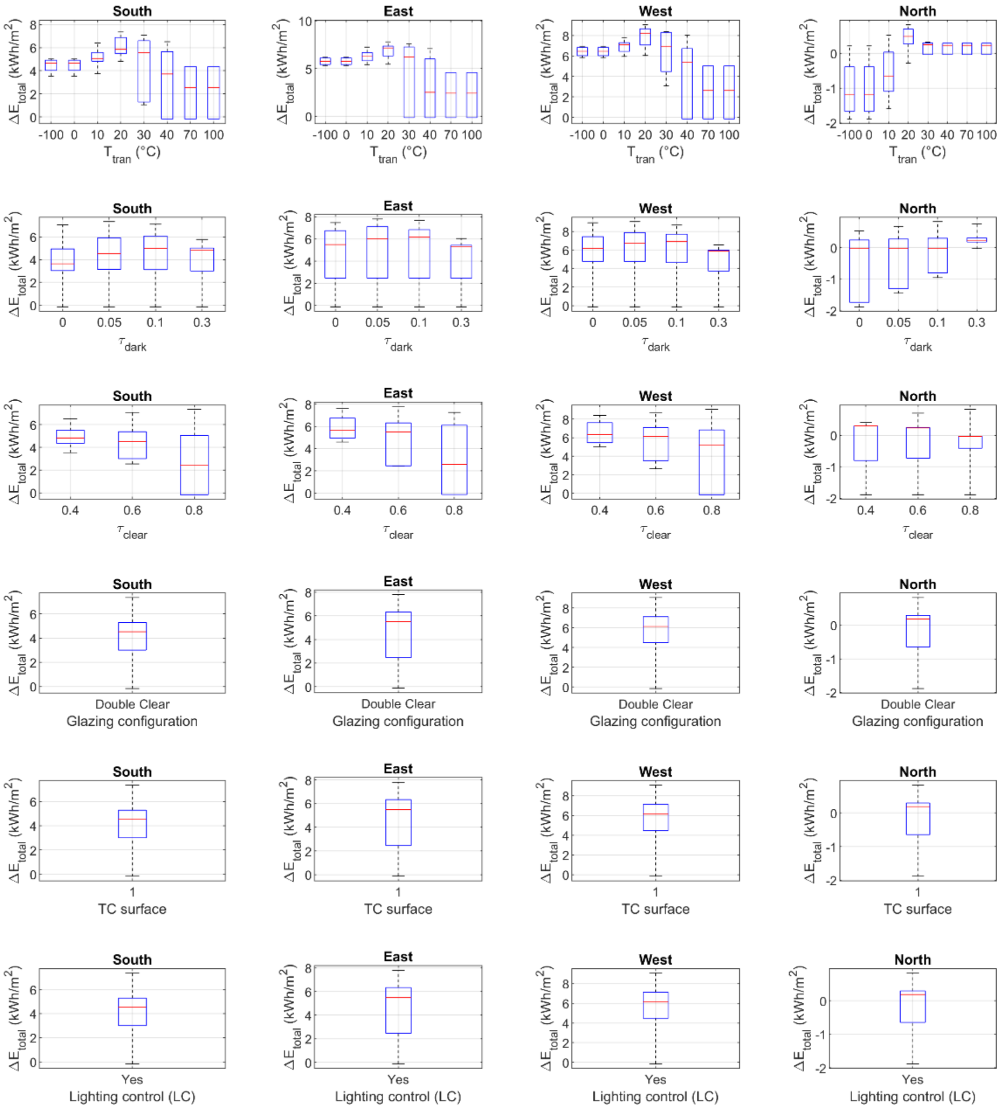

Supplementary Figure 42| Statistical analysis of the total site energy saving per conditioned floor area (ΔE_{total}) by TR windows with three variables (T_{tran}, τ_{dark}, and τ_{clear}) and three fixed parameters (glazing configuration (double clear), TR-applied surface (exterior), and lighting control (Yes)) in four window orientations in Seattle, Washington (climate classification: 4C). In each box plot, the red central mark on each box indicates the median, and the bottom and top edges of the box indicate the 25th and 75th percentiles, respectively. The short black marks above and below each box indicate the maximum and minimum, respectively.

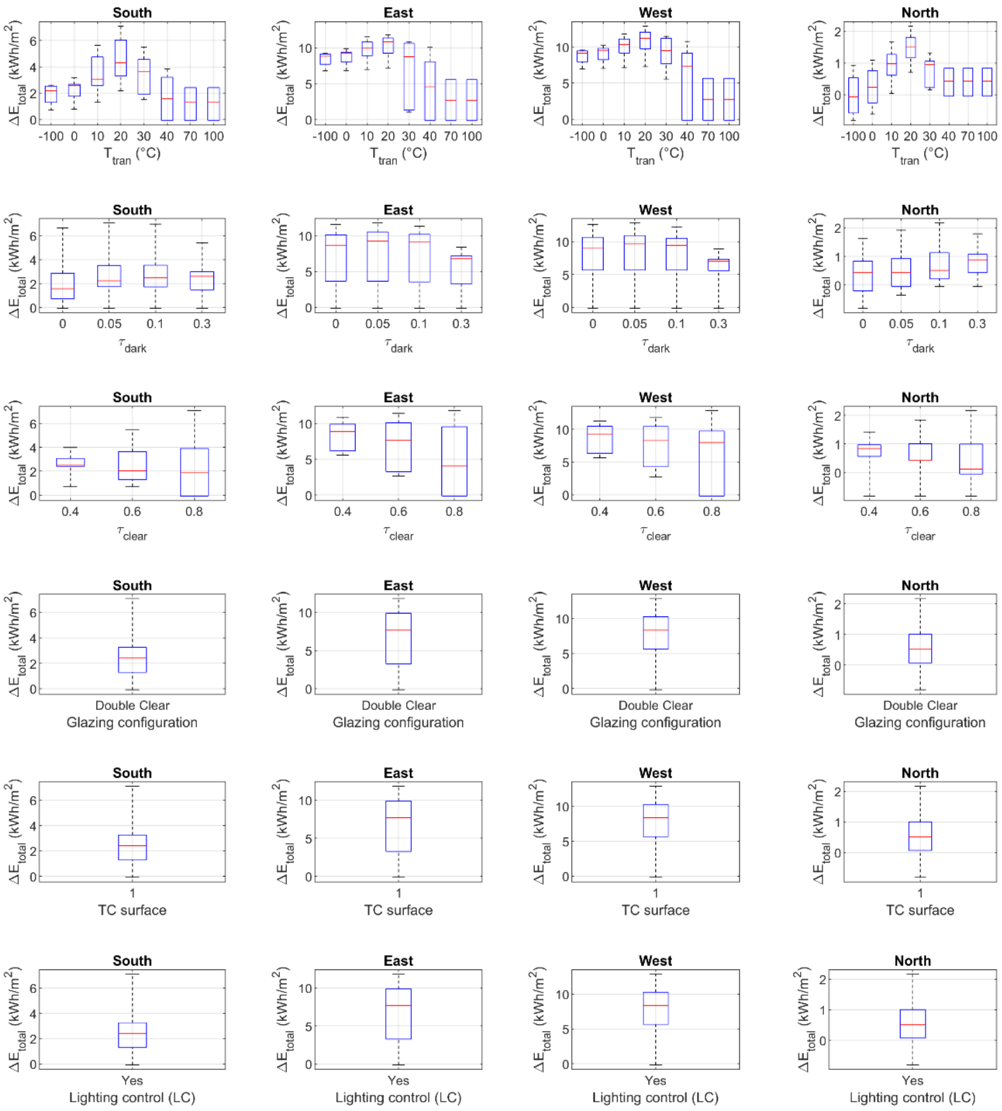

Supplementary Figure 43| Statistical analysis of the total site energy saving per conditioned floor area (ΔE_{total}) by TR windows with three variables (T_{tran}, τ_{dark}, and τ_{clear}) and three fixed parameters (glazing configuration (double clear), TR-applied surface (exterior), and lighting control (Yes)) in four window orientations in Chicago, Illinois (climate classification: 5A). In each box plot, the red central mark on each box indicates the median, and the bottom and top edges of the box indicate the 25th and 75th percentiles, respectively. The short black marks above and below each box indicate the maximum and minimum, respectively.

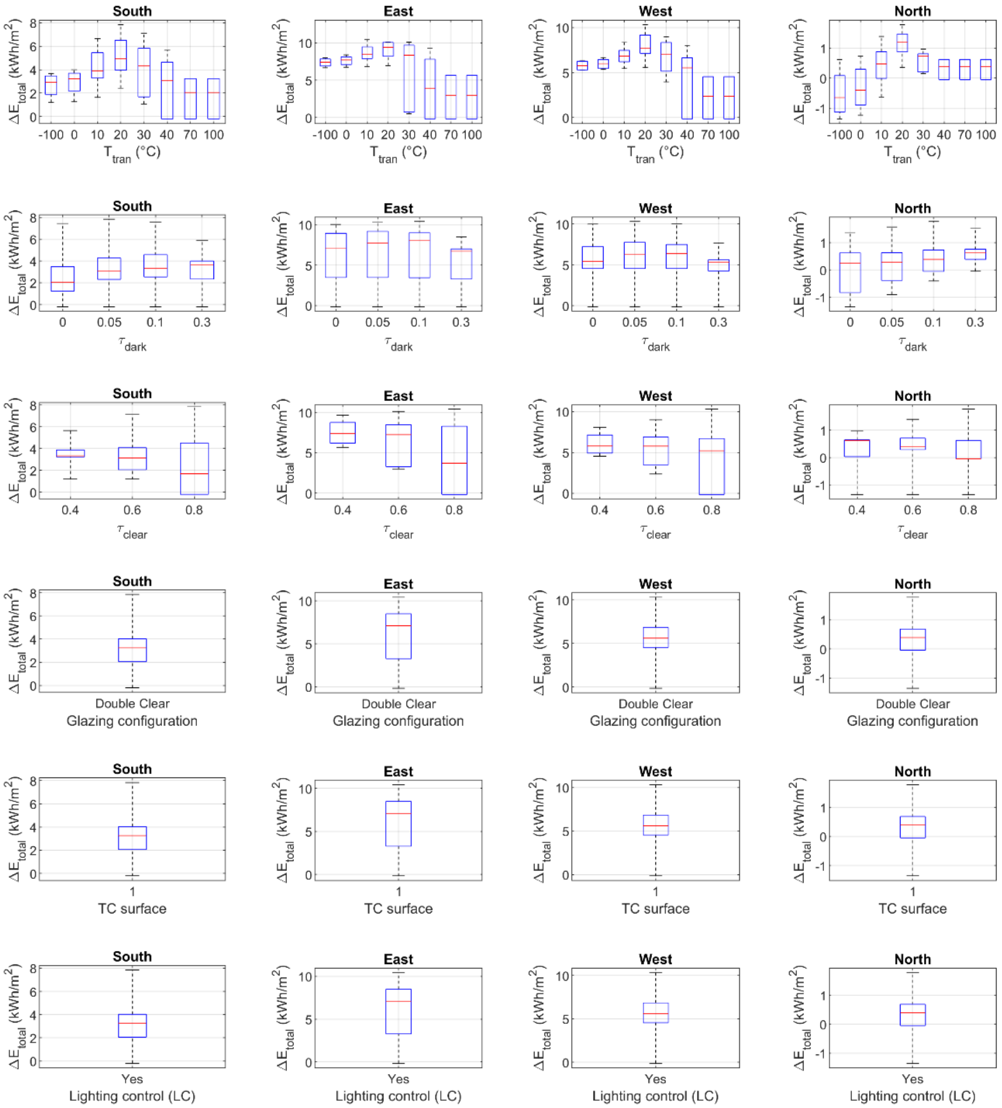

Supplementary Figure 44| Statistical analysis of the total site energy saving per conditioned floor area (ΔE_{total}) by TR windows with three variables (T_{tran}, τ_{dark}, and τ_{clear}) and three fixed parameters (glazing configuration (double clear), TR-applied surface (exterior), and lighting control (Yes)) in four window orientations in Boulder, Colorado (climate classification: 5B). In each box plot, the red central mark on each box indicates the median, and the bottom and top edges of the box indicate the 25th and 75th percentiles, respectively. The short black marks above and below each box indicate the maximum and minimum, respectively.

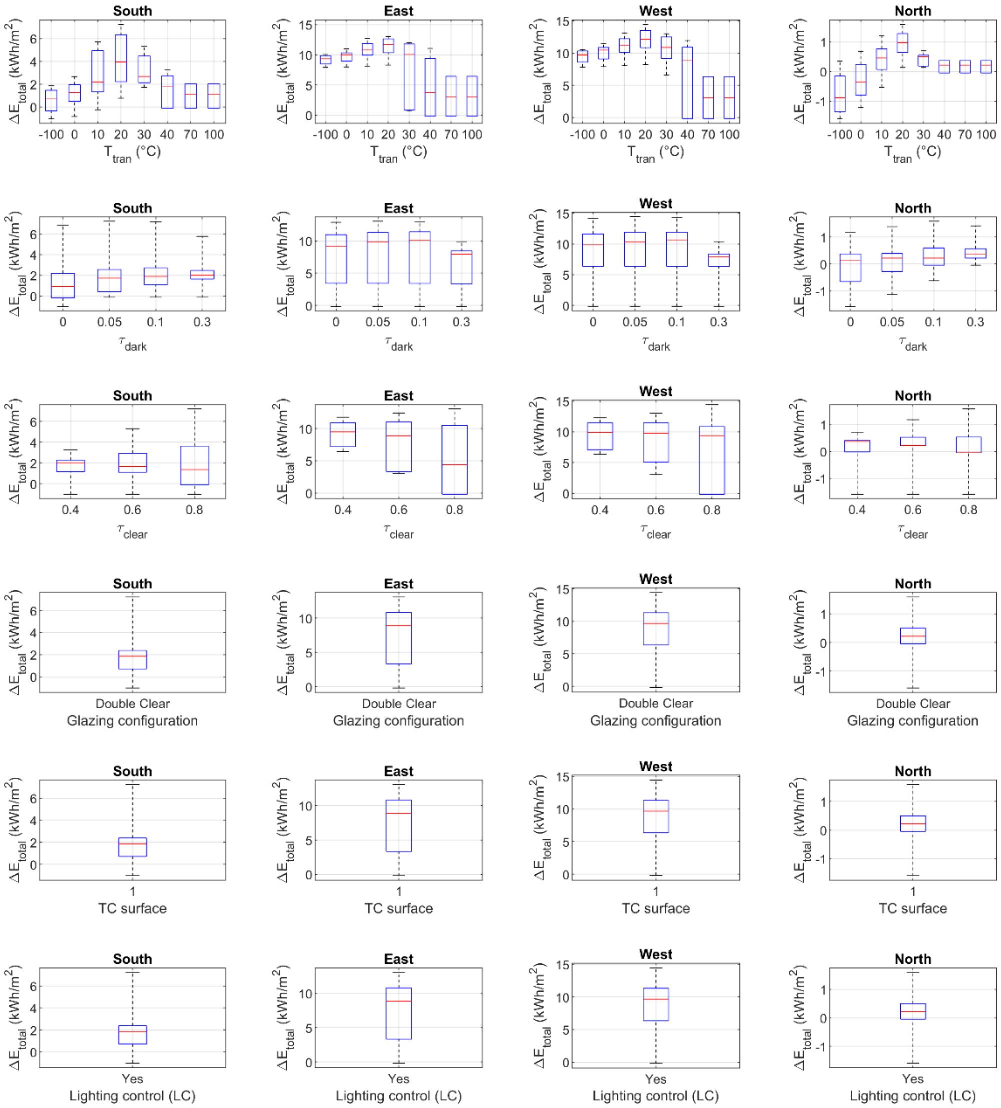

Supplementary Figure 45 | Statistical analysis of the total site energy saving per conditioned floor area (ΔE_{total}) by TR windows with three variables (T_{tran}, τ_{dark}, and τ_{clear}) and three fixed parameters (glazing configuration (double clear), TR-applied surface (exterior), and lighting control (Yes)) in four window orientations in Minneapolis, Minnesota (climate classification: 6A). In each box plot, the red central mark on each box indicates the median, and the bottom and top edges of the box indicate the 25th and 75th percentiles, respectively. The short black marks above and below each box indicate the maximum and minimum, respectively.

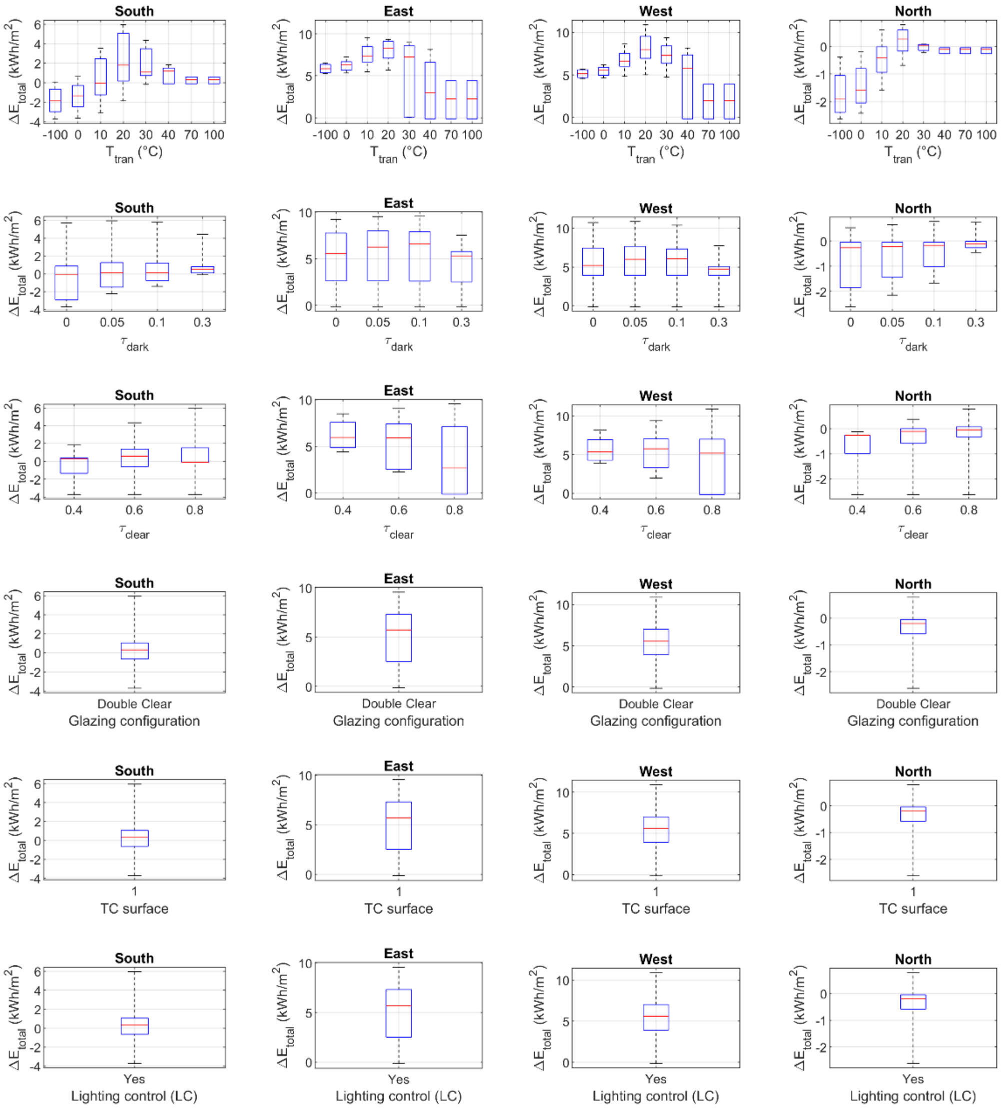

Supplementary Figure 46| Statistical analysis of the total site energy saving per conditioned floor area (ΔE_{total}) by TR windows with three variables (T_{tran}, τ_{dark}, and τ_{clear}) and three fixed parameters (glazing configuration (double clear), TR-applied surface (exterior), and lighting control (Yes)) in four window orientations in Helena, Montana (climate classification: 6B). In each box plot, the red central mark on each box indicates the median, and the bottom and top edges of the box indicate the 25th and 75th percentiles, respectively. The short black marks above and below each box indicate the maximum and minimum, respectively.

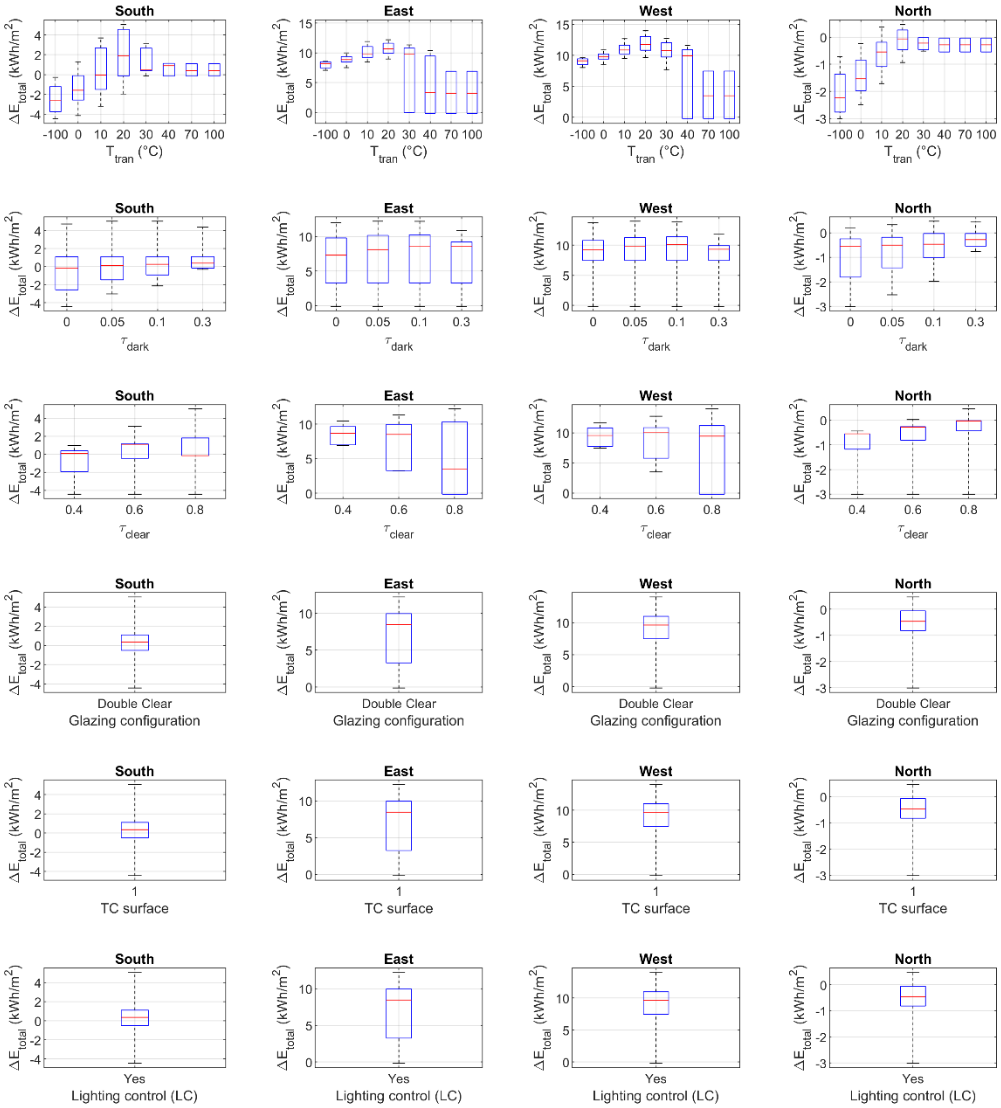

Supplementary Figure 47| Statistical analysis of the total site energy saving per conditioned floor area (ΔE_{total}) by TR windows with three variables (T_{tran}, τ_{dark}, and τ_{clear}) and three fixed parameters (glazing configuration (double clear), TR-applied surface (exterior), and lighting control (Yes)) in four window orientations in Duluth, Minnesota (climate classification: 7). In each box plot, the red central mark on each box indicates the median, and the bottom and top edges of the box indicate the 25th and 75th percentiles, respectively. The short black marks above and below each box indicate the maximum and minimum, respectively.

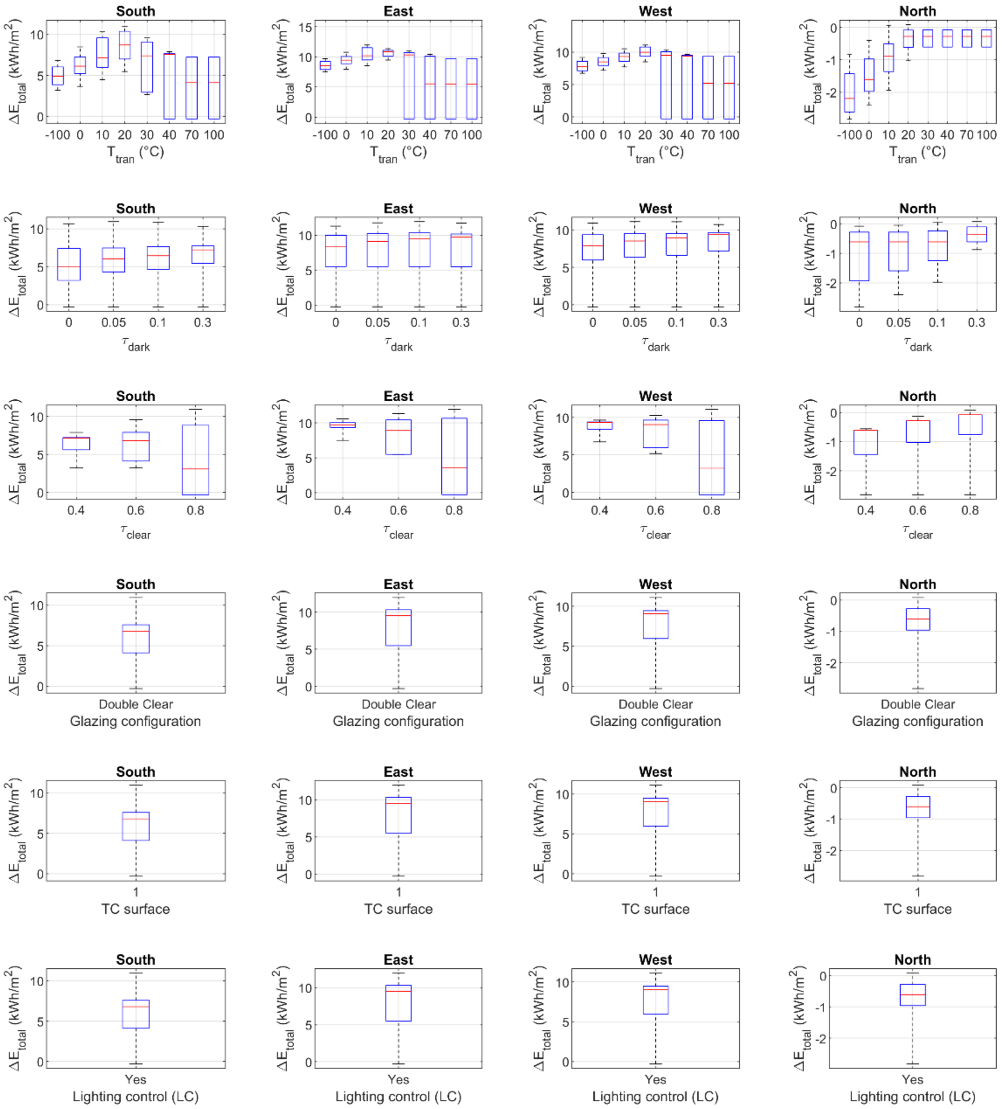

Supplementary Figure 48| Statistical analysis of the total site energy saving per conditioned floor area (ΔE_{total}) by TR windows with three variables (T_{tran}, τ_{dark}, and τ_{clear}) and three fixed parameters (glazing configuration (double clear), TR-applied surface (exterior), and lighting control (Yes)) in four window orientations in Fairbanks, Alaska (climate classification: 8). In each box plot, the red central mark on each box indicates the median, and the bottom and top edges of the box indicate the 25th and 75th percentiles, respectively. The short black marks above and below each box indicate the maximum and minimum, respectively.

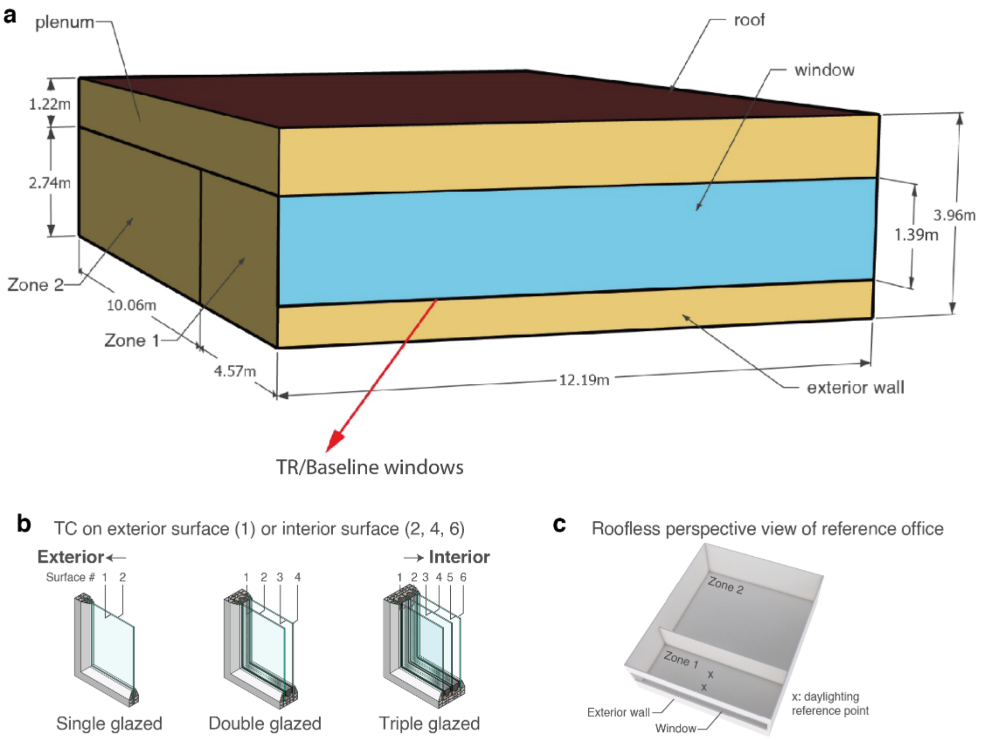

Supplementary Figure 49|Building models for EnergyPlus Simulations. a, Schematic diagram of reference commercial office room with a thermochromic/baseline window. This model is adapted from the U.S. Department of Energy (DOE) reference model for a medium-size office building[1]. The reference office room is divided into three zones, among which only one contains an exterior wall with fenestration, where the thermochromic or baseline window in this study is installed. The other two zones in the model are the interior part of the space and the ceiling plenum. The net conditioned building area is 178.4 m^2. This model has also been used in our previous study on phase change material (PCM) windows[2]. **b**, Window glazing configurations and TR-applied surface. **c**, Daylighting reference points in the model of commercial office space.

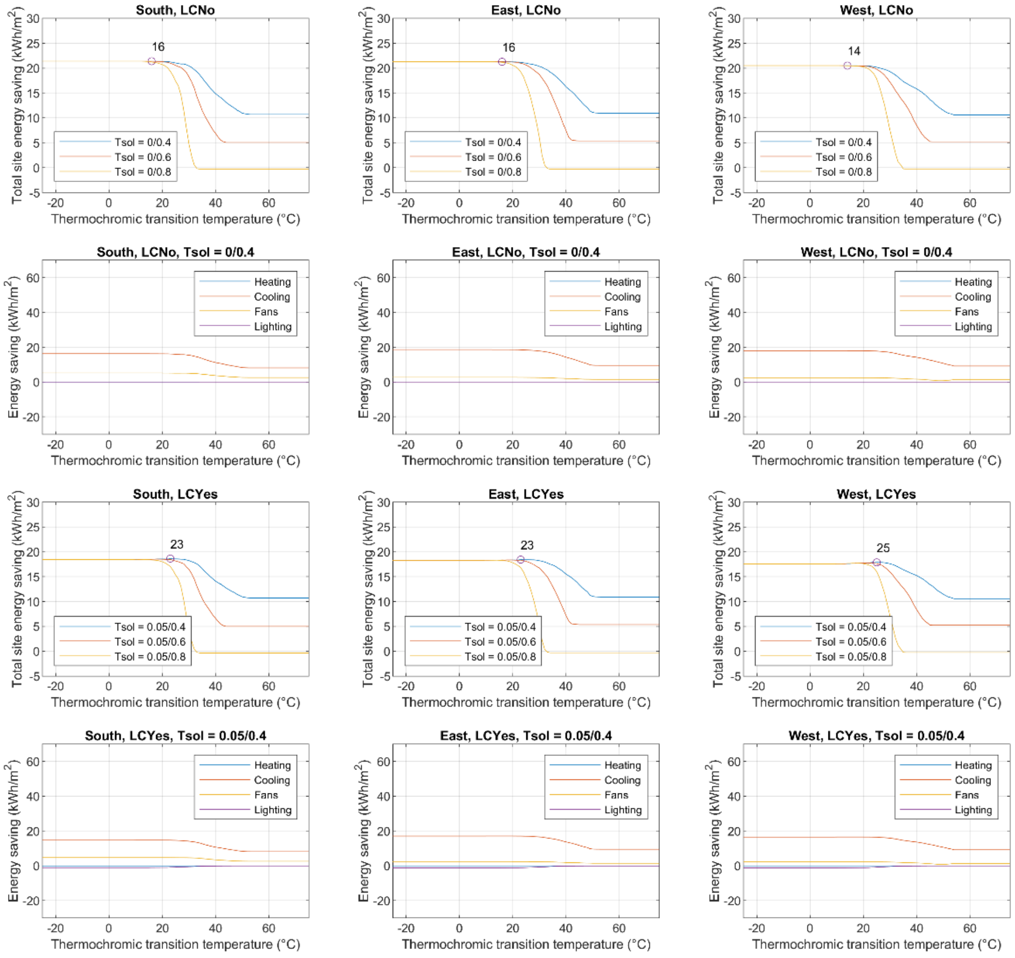

Supplementary Figure 50| Total site energy saving (first and third rows) and sectoral energy saving (second and fourth rows) per conditioned floor area by TR windows as functions of transition temperature in Miami, Florida (climate classification: 1A). One TR variable (τ_{clear}) and two building variables (lighting control (LC No for first and second rows, and LC Yes for third and fourth rows), and window orientations (south, east, and west for each column)) are considered. Other parameters (τ_{dark} (0 for LC No, and 0.05 for LC Yes), glazing configuration (double clear), and TR-applied surface (exterior)) are fixed. The maximum total site energy point is circled, where the optimal transition temperature is labeled.

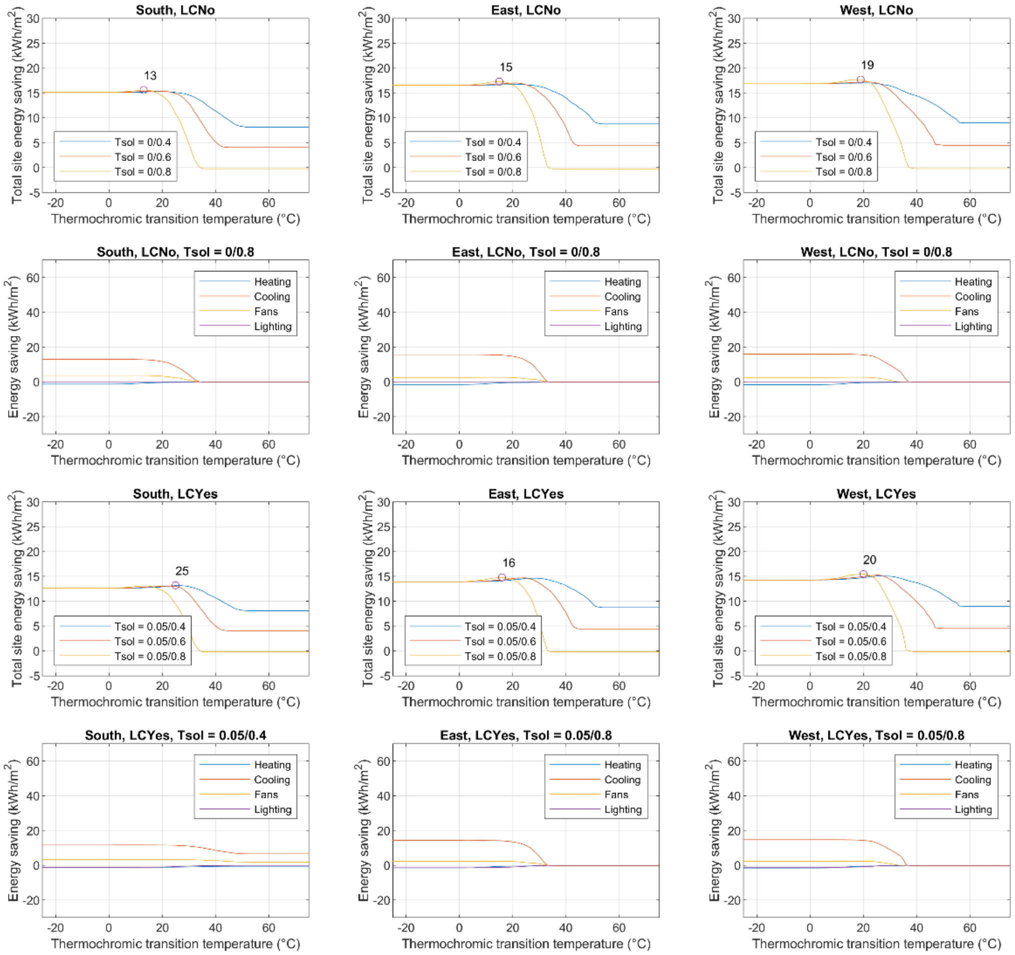

Supplementary Figure 51| Total site energy saving (first and third rows) and sectoral energy saving (second and fourth rows) per conditioned floor area by TR windows as functions of transition temperature in Houston, Texas (climate classification: 2A). One TR variable (τ_{clear}) and two building variables (lighting control (LC No for first and second rows, and LC Yes for third and fourth rows), and window orientations (south, east, and west for each column)) are considered. Other parameters (τ_{dark} (0 for LC No, and 0.05 for LC Yes), glazing configuration (double clear), and TR-applied surface (exterior)) are fixed. The maximum total site energy point is circled, where the optimal transition temperature is labeled.

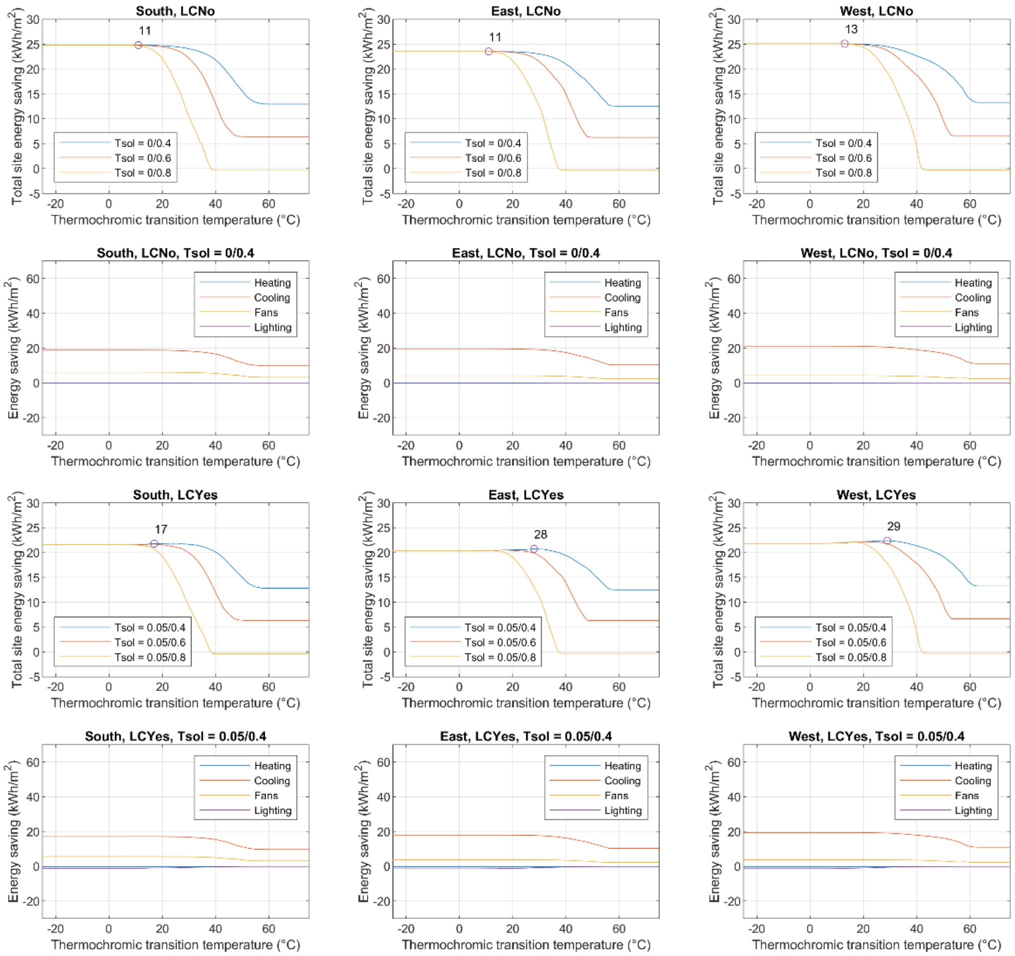

Supplementary Figure 52| Total site energy saving (first and third rows) and sectoral energy saving (second and fourth rows) per conditioned floor area by TR windows as functions of transition temperature in Phoenix, Arizona (climate classification: 2B). One TR variable (τ_{clear}) and two building variables (lighting control (LC No for first and second rows, and LC Yes for third and fourth rows), and window orientations (south, east, and west for each column)) are considered. Other parameters (τ_{dark} (0 for LC No, and 0.05 for LC Yes), glazing configuration (double clear), and TR-applied surface (exterior)) are fixed. The maximum total site energy point is circled, where the optimal transition temperature is labeled.

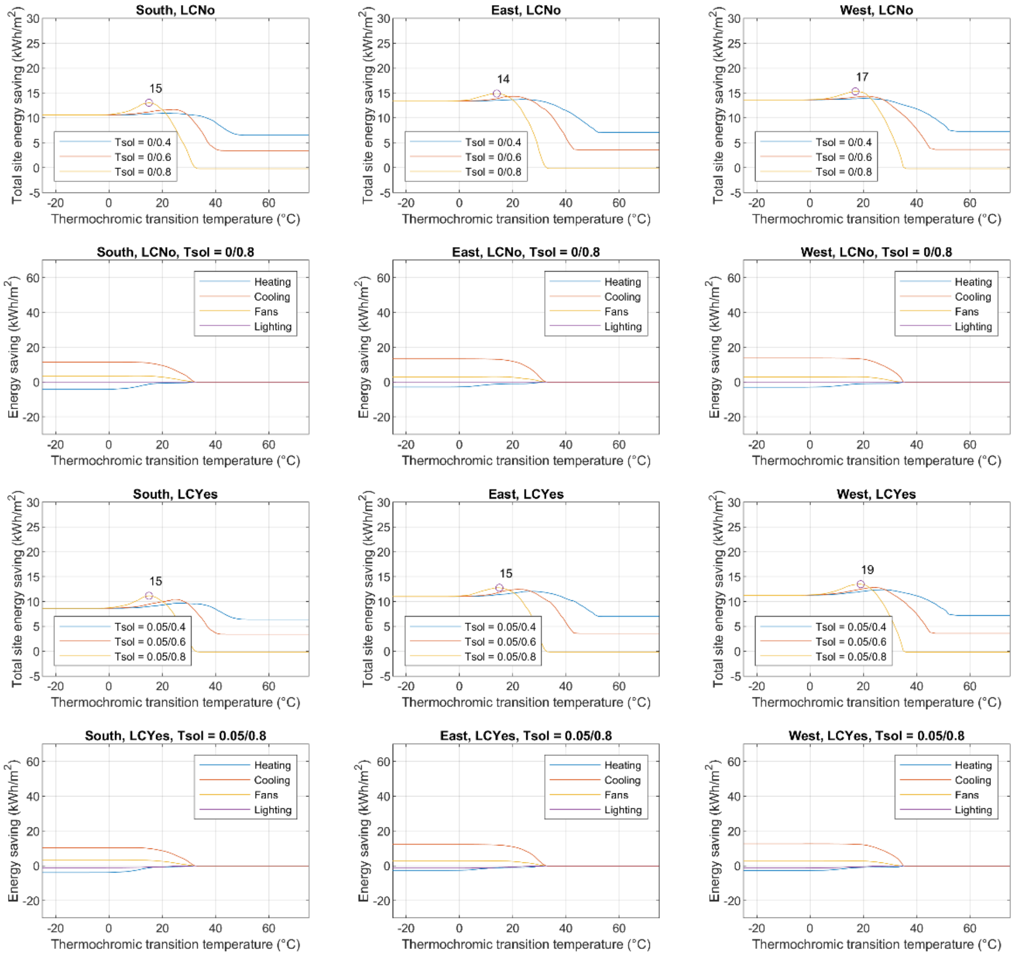

Supplementary Figure 53| Total site energy saving (first and third rows) and sectoral energy saving (second and fourth rows) per conditioned floor area by TR windows as functions of transition temperature in Atlanta, Georgia (climate classification: 3A). One TR variable (τ_{clear}) and two building variables (lighting control (LC No for first and second rows, and LC Yes for third and fourth rows), and window orientations (south, east, and west for each column)) are considered. Other parameters (τ_{dark} (0 for LC No, and 0.05 for LC Yes), glazing configuration (double clear), and TR-applied surface (exterior)) are fixed. The maximum total site energy point is circled, where the optimal transition temperature is labeled.

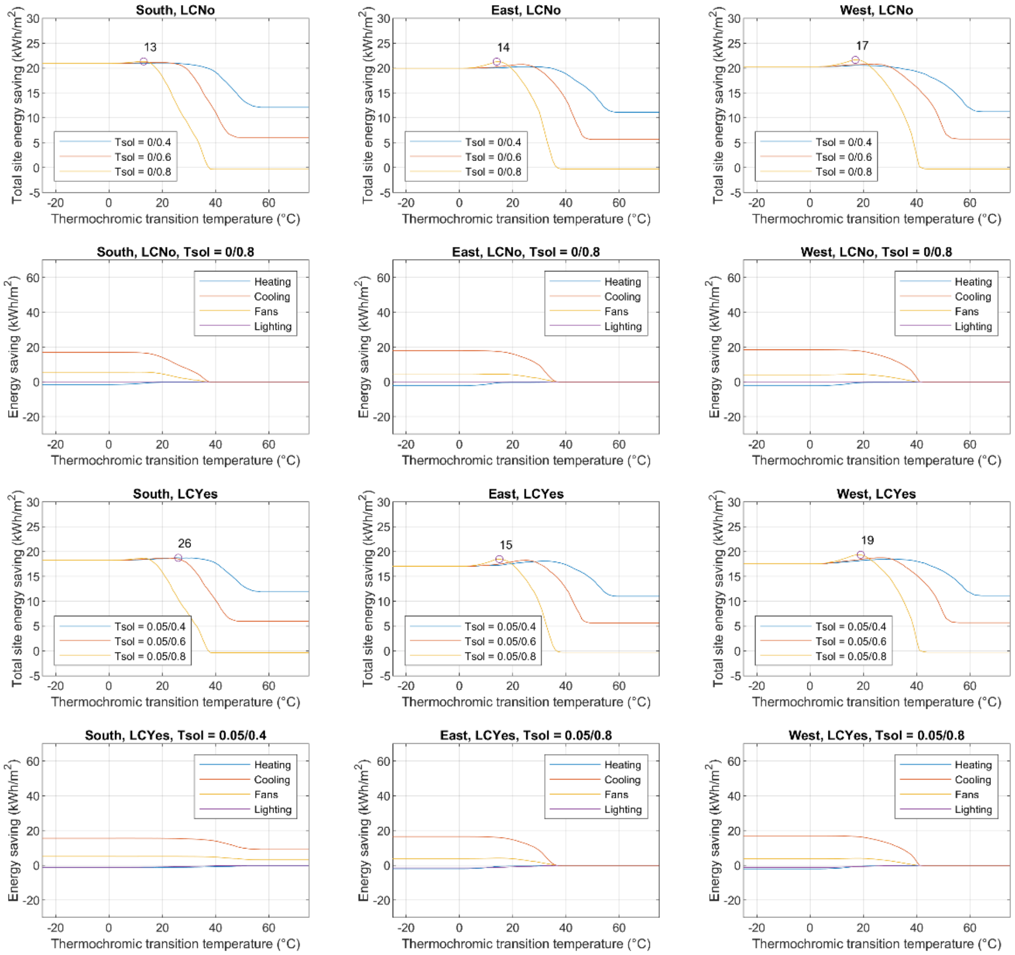

Supplementary Figure 54| Total site energy saving (first and third rows) and sectoral energy saving (second and fourth rows) per conditioned floor area by TR windows as functions of transition temperature in Las Vegas, Nevada (climate classification: 3B). One TR variable (τ_{clear}) and two building variables (lighting control (LC No for first and second rows, and LC Yes for third and fourth rows), and window orientations (south, east, and west for each column)) are considered. Other parameters (τ_{dark} (0 for LC No, and 0.05 for LC Yes), glazing configuration (double clear), and TR-applied surface (exterior)) are fixed. The maximum total site energy point is circled, where the optimal transition temperature is labeled.

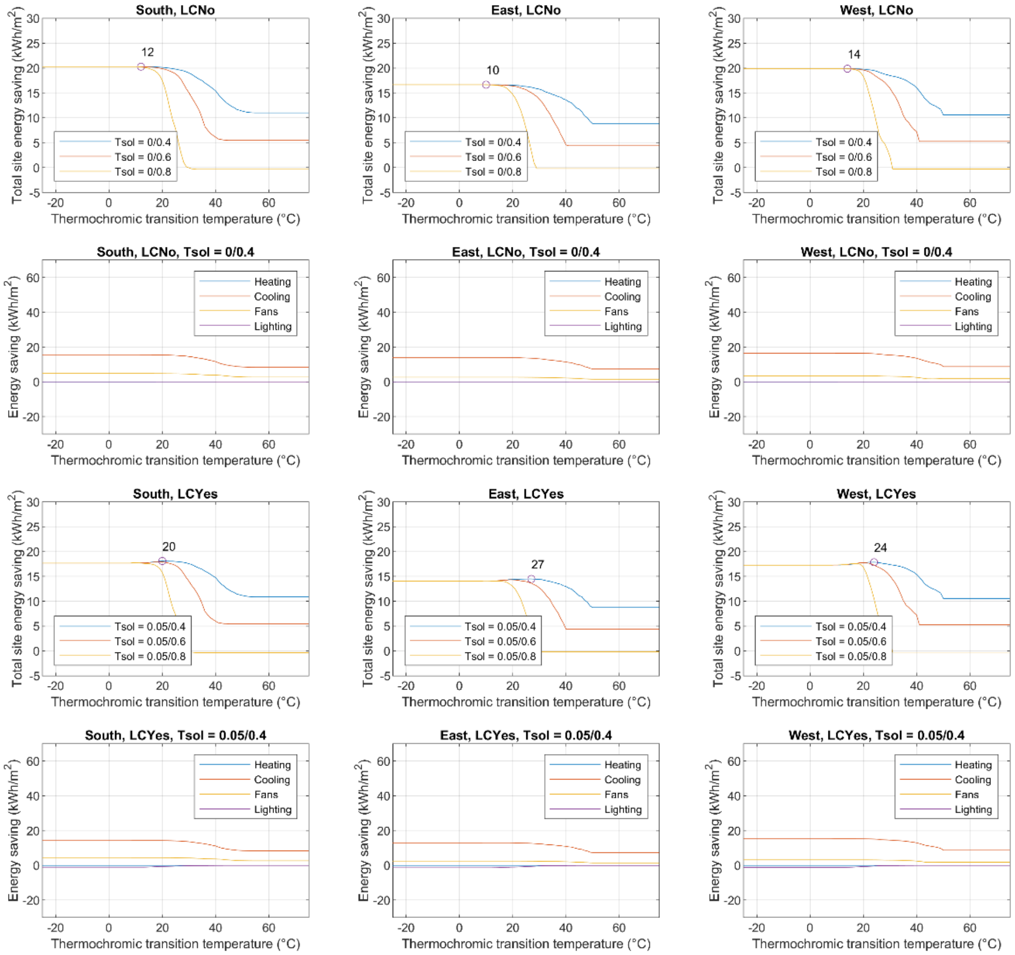

Supplementary Figure 55| Total site energy saving (first and third rows) and sectoral energy saving (second and fourth rows) per conditioned floor area by TR windows as functions of transition temperature in Los Angeles, California (climate classification: 3B). One TR variable (τ_{clear}) and two building variables (lighting control (LC No for first and second rows, and LC Yes for third and fourth rows), and window orientations (south, east, and west for each column)) are considered. Other parameters (τ_{dark} (0 for LC No, and 0.05 for LC Yes), glazing configuration (double clear), and TR-applied surface (exterior)) are fixed. The maximum total site energy point is circled, where the optimal transition temperature is labeled.

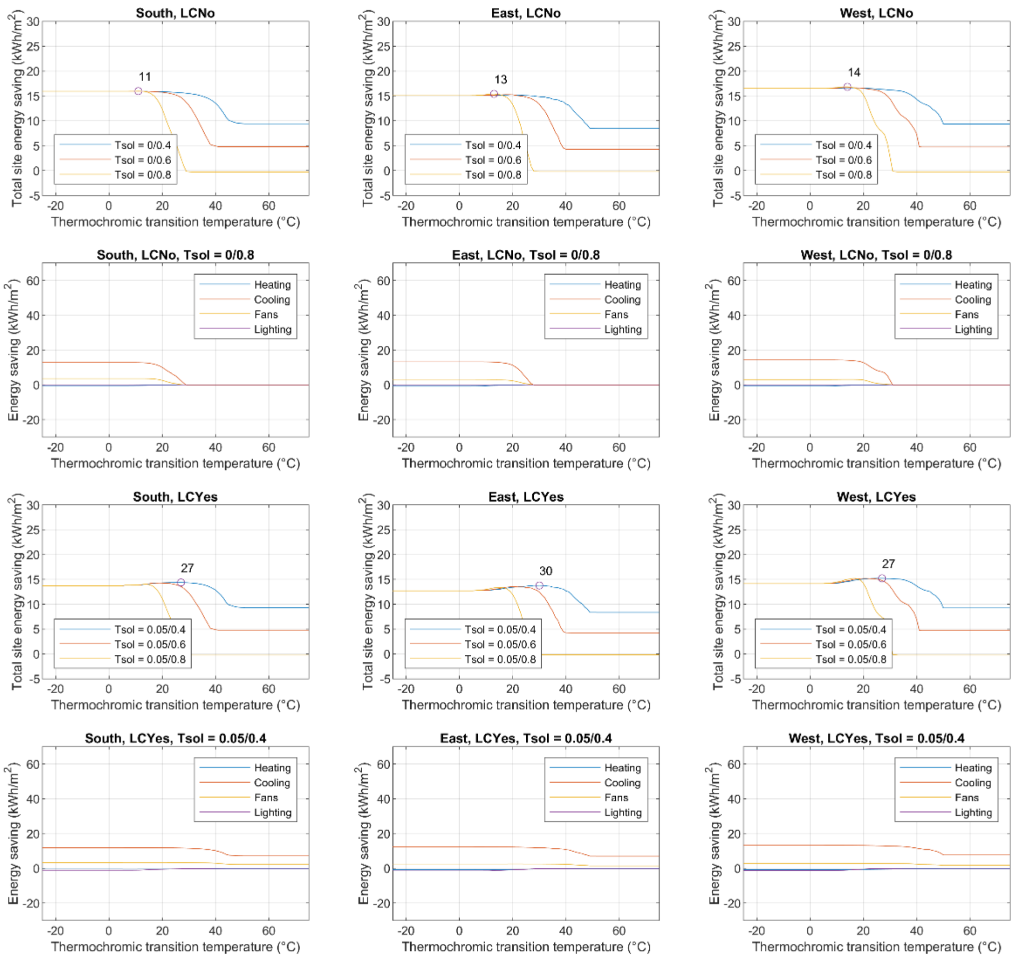

Supplementary Figure 56| Total site energy saving (first and third rows) and sectoral energy saving (second and fourth rows) per conditioned floor area by TR windows as functions of transition temperature in San Francisco, California (climate classification: 3C). One TR variable (τ_{clear}) and two building variables (lighting control (LC No for first and second rows, and LC Yes for third and fourth rows), and window orientations (south, east, and west for each column)) are considered. Other parameters (τ_{dark} (0 for LC No, and 0.05 for LC Yes), glazing configuration (double clear), and TR-applied surface (exterior)) are fixed. The maximum total site energy point is circled, where the optimal transition temperature is labeled.

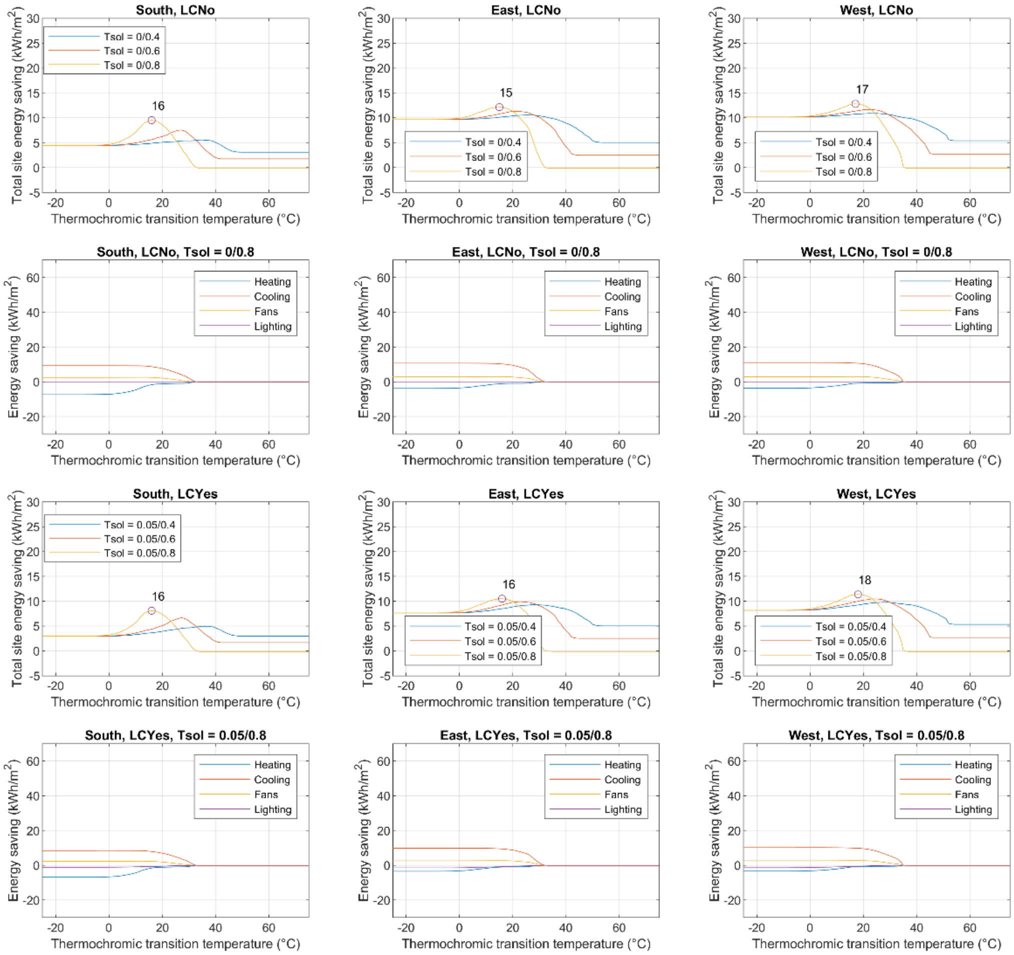

Supplementary Figure 57| Total site energy saving (first and third rows) and sectoral energy saving (second and fourth rows) per conditioned floor area by TR windows as functions of transition temperature in Baltimore, Maryland (climate classification: 4A). One TR variable (τ_{clear}) and two building variables (lighting control (LC No for first and second rows, and LC Yes for third and fourth rows), and window orientations (south, east, and west for each column)) are considered. Other parameters (τ_{dark} (0 for LC No, and 0.05 for LC Yes), glazing configuration (double clear), and TR-applied surface (exterior)) are fixed. The maximum total site energy point is circled, where the optimal transition temperature is labeled.

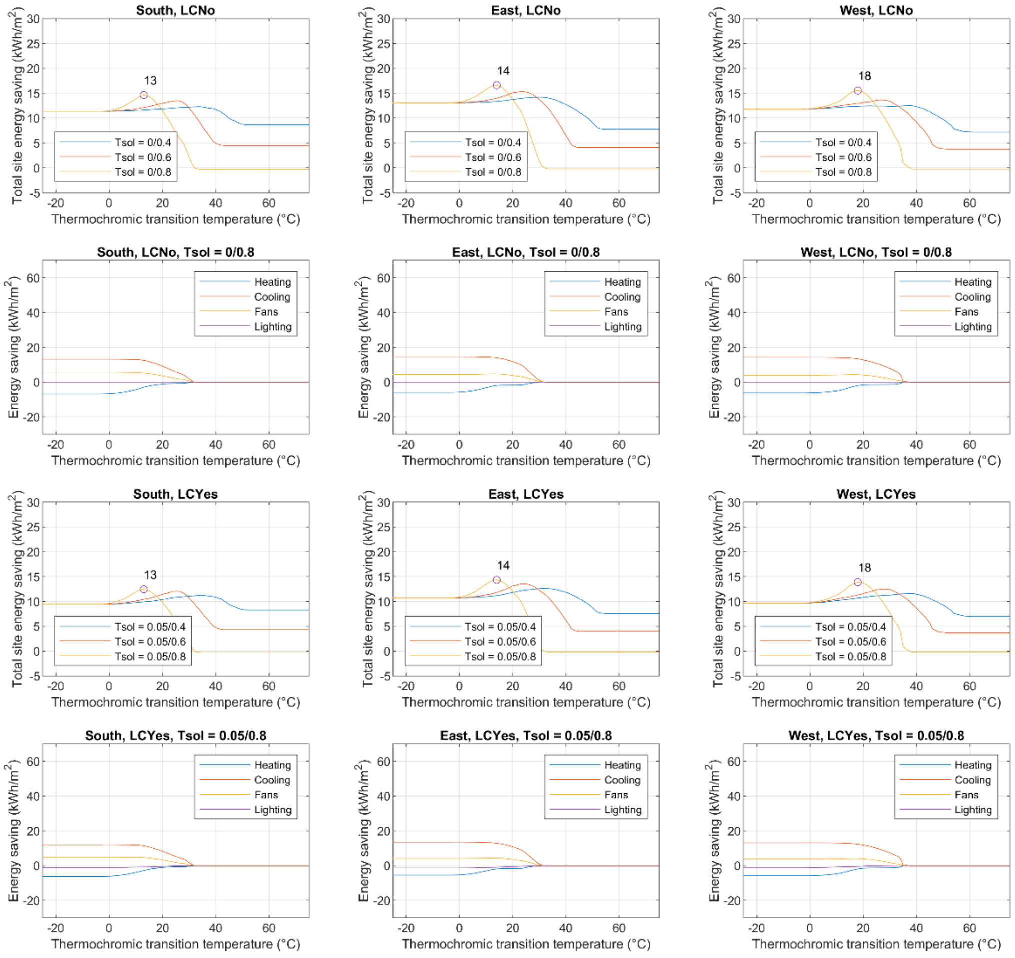

Supplementary Figure 58| Total site energy saving (first and third rows) and sectoral energy saving (second and fourth rows) per conditioned floor area by TR windows as functions of transition temperature in Albuquerque, New Mexico (climate classification: 4B). One TR variable (τ_{clear}) and two building variables (lighting control (LC No for first and second rows, and LC Yes for third and fourth rows), and window orientations (south, east, and west for each column)) are considered. Other parameters (τ_{dark} (0 for LC No, and 0.05 for LC Yes), glazing configuration (double clear), and TR-applied surface (exterior)) are fixed. The maximum total site energy point is circled, where the optimal transition temperature is labeled.

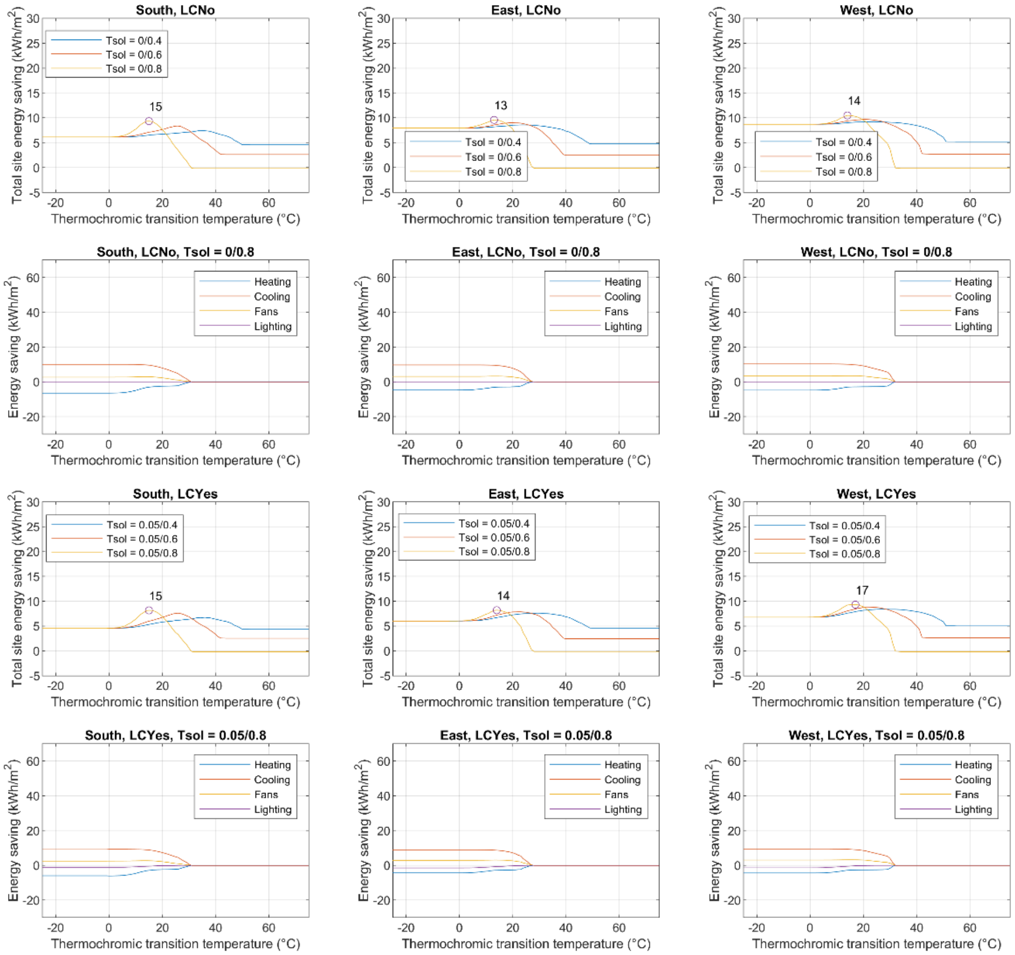

Supplementary Figure 59| Total site energy saving (first and third rows) and sectoral energy saving (second and fourth rows) per conditioned floor area by TR windows as functions of transition temperature in Seattle, Washington (climate classification: 4C). One TR variable (τ_{clear}) and two building variables (lighting control (LC No for first and second rows, and LC Yes for third and fourth rows), and window orientations (south, east, and west for each column)) are considered. Other parameters (τ_{dark} (0 for LC No, and 0.05 for LC Yes), glazing configuration (double clear), and TR-applied surface (exterior)) are fixed. The maximum total site energy point is circled, where the optimal transition temperature is labeled.

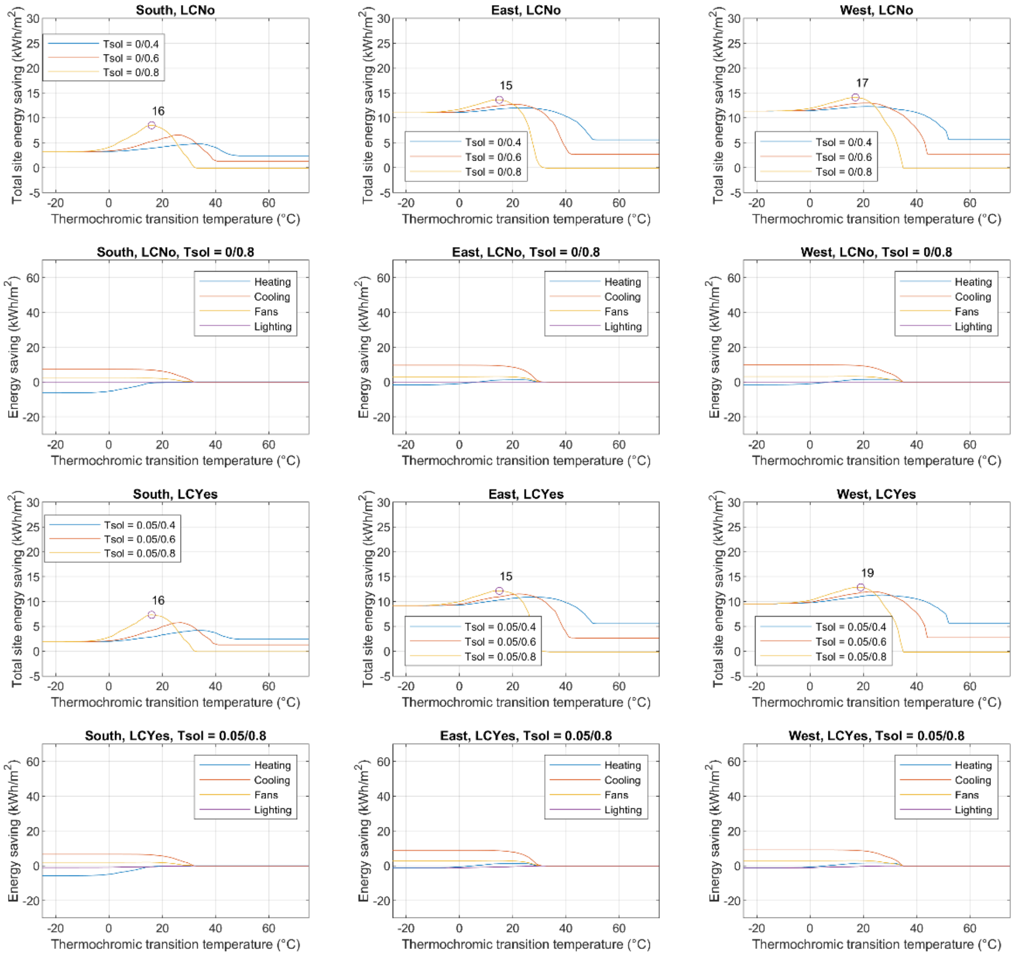

Supplementary Figure 60| Total site energy saving (first and third rows) and sectoral energy saving (second and fourth rows) per conditioned floor area by TR windows as functions of transition temperature in Chicago, Illinois (climate classification: 5A). One TR variable (τ_{clear}) and two building variables (lighting control (LC No for first and second rows, and LC Yes for third and fourth rows), and window orientations (south, east, and west for each column)) are considered. Other parameters (τ_{dark} (0 for LC No, and 0.05 for LC Yes), glazing configuration (double clear), and TR-applied surface (exterior)) are fixed. The maximum total site energy point is circled, where the optimal transition temperature is labeled.

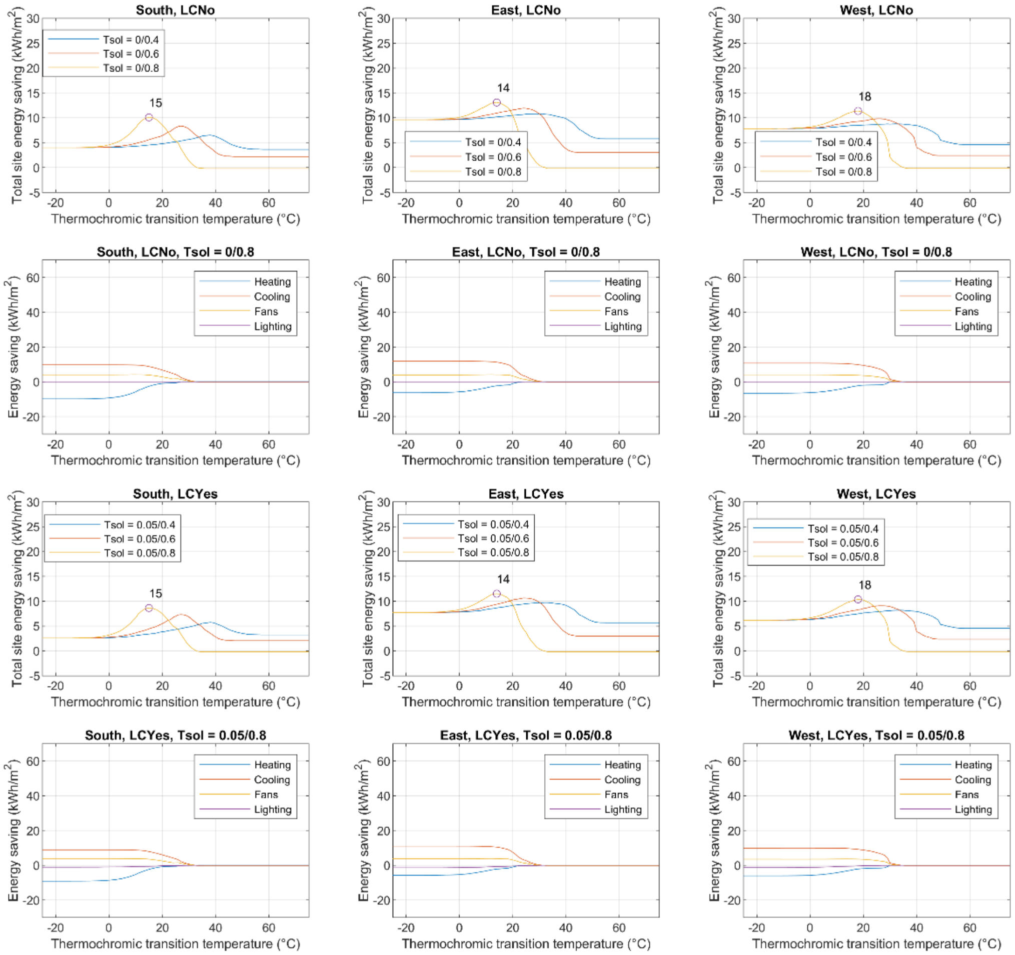

Supplementary Figure 61| Total site energy saving (first and third rows) and sectoral energy saving (second and fourth rows) per conditioned floor area by TR windows as functions of transition temperature in Boulder, Colorado (climate classification: 5B). One TR variable (τ_{clear}) and two building variables (lighting control (LC No for first and second rows, and LC Yes for third and fourth rows), and window orientations (south, east, and west for each column)) are considered. Other parameters (τ_{dark} (0 for LC No, and 0.05 for LC Yes), glazing configuration (double clear), and TR-applied surface (exterior)) are fixed. The maximum total site energy point is circled, where the optimal transition temperature is labeled.

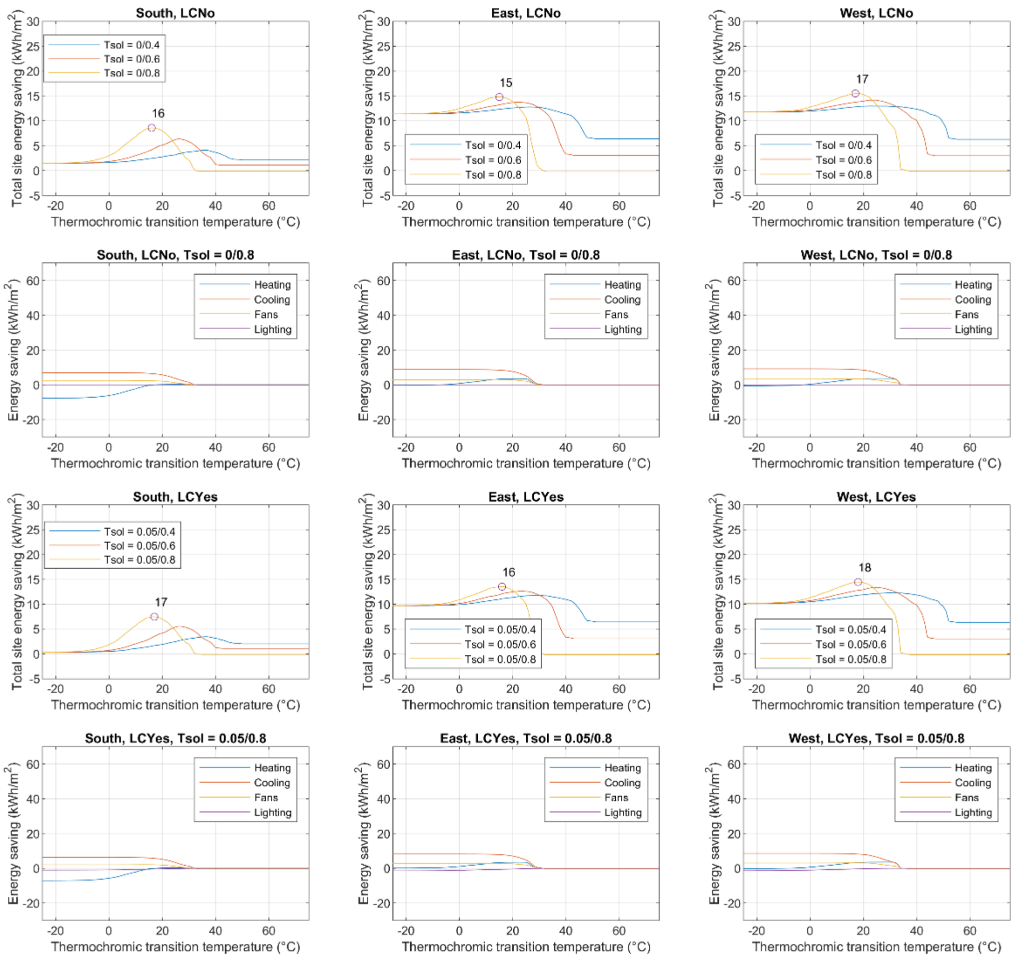

Supplementary Figure 62|Total site energy saving (first and third rows) and sectoral energy saving (second and fourth rows) per conditioned floor area by TR windows as functions of transition temperature in Minneapolis, Minnesota (climate classification: 6A). One TR variable (τ_{clear}) and two building variables (lighting control (LC No for first and second rows, and LC Yes for third and fourth rows), and window orientations (south, east, and west for each column)) are considered. Other parameters (τ_{dark} (0 for LC No, and 0.05 for LC Yes), glazing configuration (double clear), and TR-applied surface (exterior)) are fixed. The maximum total site energy point is circled, where the optimal transition temperature is labeled.

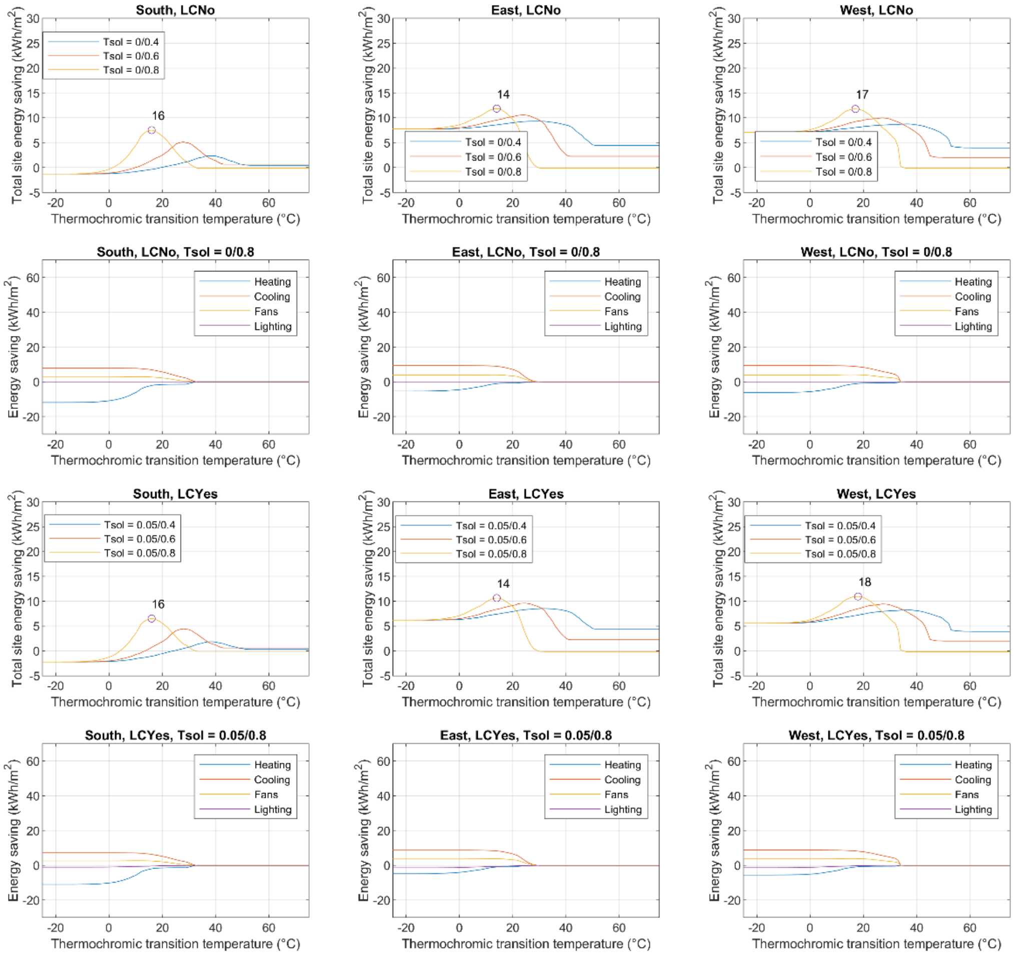

Supplementary Figure 63| Total site energy saving (first and third rows) and sectoral energy saving (second and fourth rows) per conditioned floor area by TR windows as functions of transition temperature in Helena, Montana (climate classification: 6B). One TR variable (τ_{clear}) and two building variables (lighting control (LC No for first and second rows, and LC Yes for third and fourth rows), and window orientations (south, east, and west for each column)) are considered. Other parameters (τ_{dark} (0 for LC No, and 0.05 for LC Yes), glazing configuration (double clear), and TR-applied surface (exterior)) are fixed. The maximum total site energy point is circled, where the optimal transition temperature is labeled.

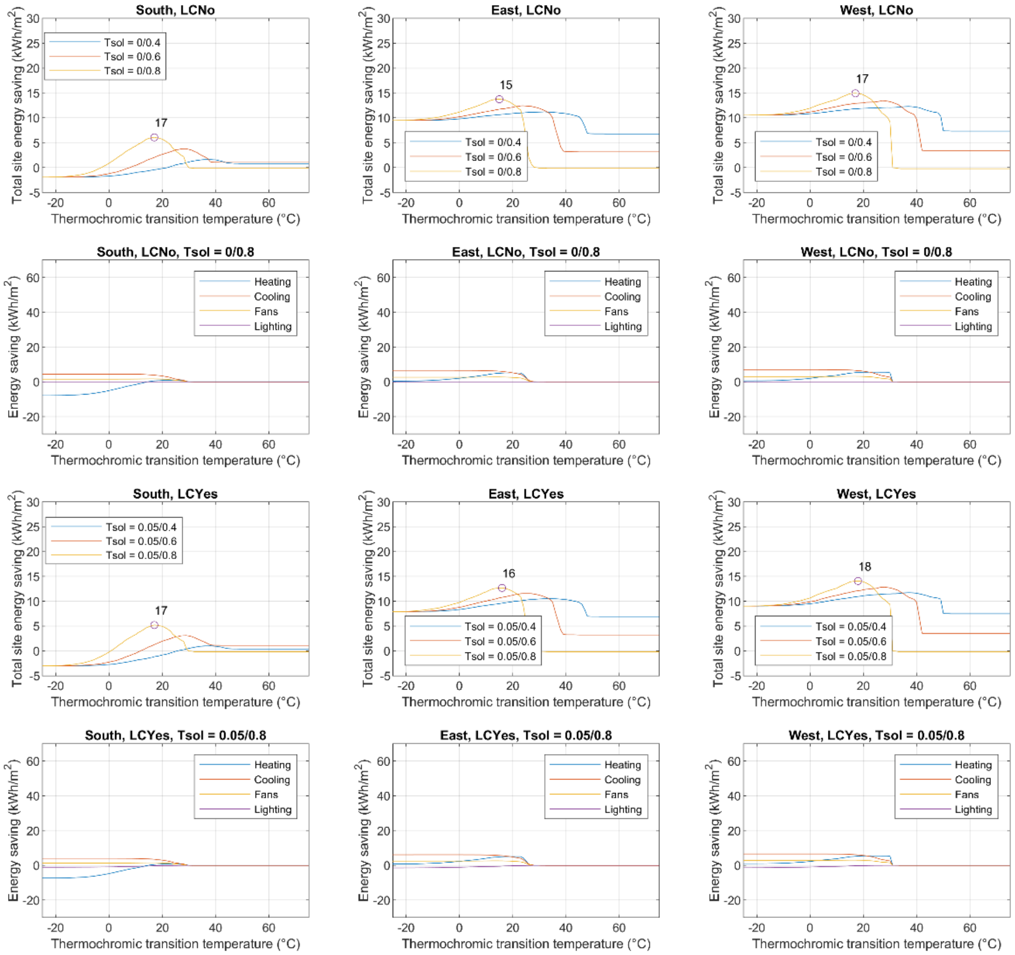

Supplementary Figure 64| Total site energy saving (first and third rows) and sectoral energy saving (second and fourth rows) per conditioned floor area by TR windows as functions of transition temperature in Duluth, Minnesota (climate classification: 7). One TR variable (τ_{clear}) and two building variables (lighting control (LC No for first and second rows, and LC Yes for third and fourth rows), and window orientations (south, east, and west for each column)) are considered. Other parameters (τ_{dark} (0 for LC No, and 0.05 for LC Yes), glazing configuration (double clear), and TR-applied surface (exterior)) are fixed. The maximum total site energy point is circled, where the optimal transition temperature is labeled.

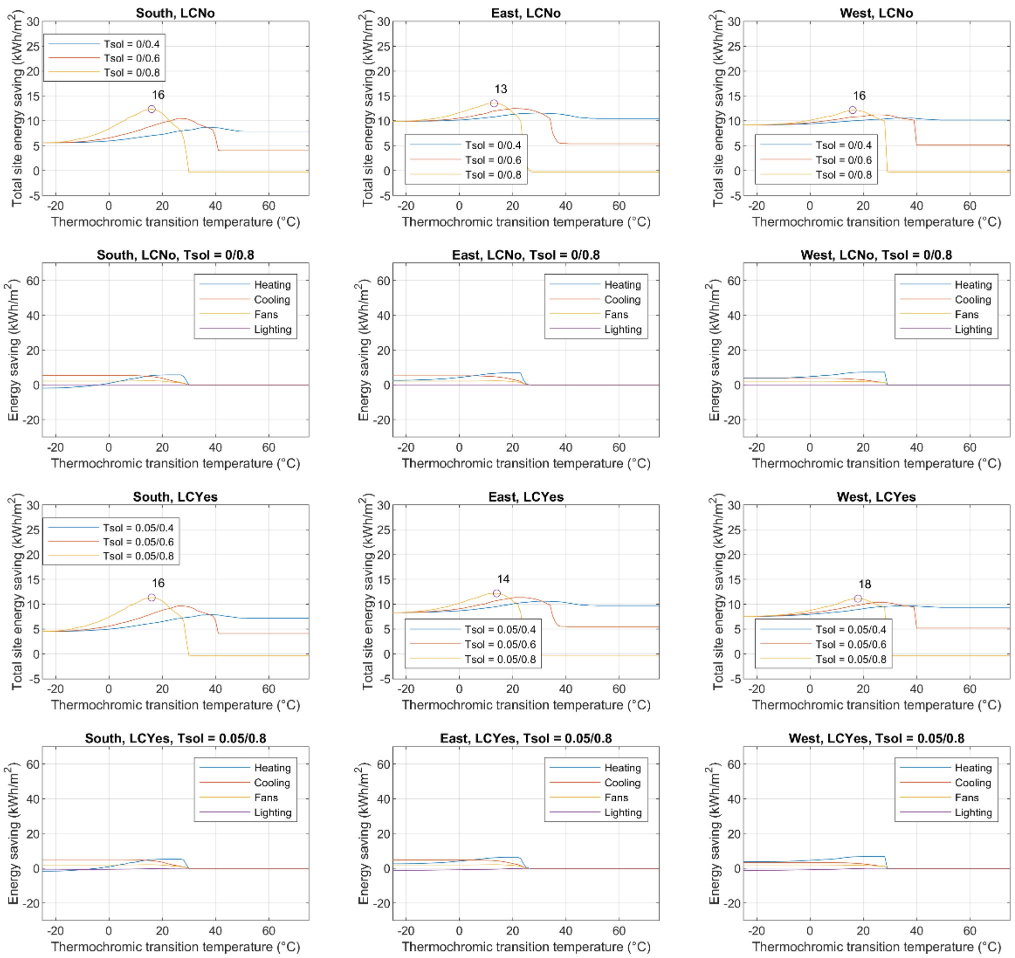

Supplementary Figure 65| Total site energy saving (first and third rows) and sectoral energy saving (second and fourth rows) per conditioned floor area by TR windows as functions of transition temperature in Fairbanks, Alaska (climate classification: 8). One TR variable (τ_{clear}) and two building variables (lighting control (LC No for first and second rows, and LC Yes for third and fourth rows), and window orientations (south, east, and west for each column)) are considered. Other parameters (τ_{dark} (0 for LC No, and 0.05 for LC Yes), glazing configuration (double clear), and TR-applied surface (exterior)) are fixed. The maximum total site energy point is circled, where the optimal transition temperature is labeled.

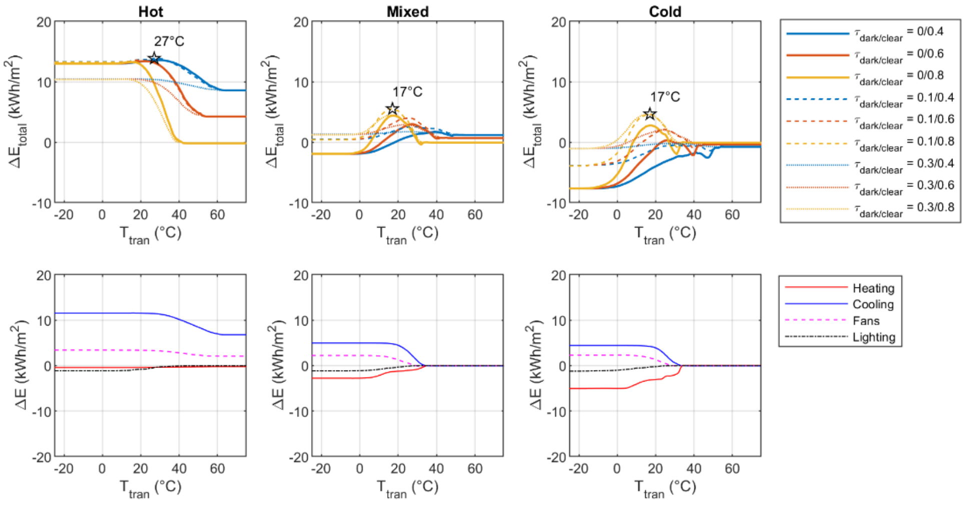

Supplementary Figure 66| Total site energy saving (ΔE_{total}) and sectoral energy saving (ΔE) per conditioned floor area in a medium office building (DOE prototype building model, three-floor, with lighting control) by TR windows (installed in all the four orientations) as functions of transition temperature (T_{tran}). The optimal τ_{dark} for energy saving is 0.1, which is higher than that in the model of office space with lighting control (0.05)[1].

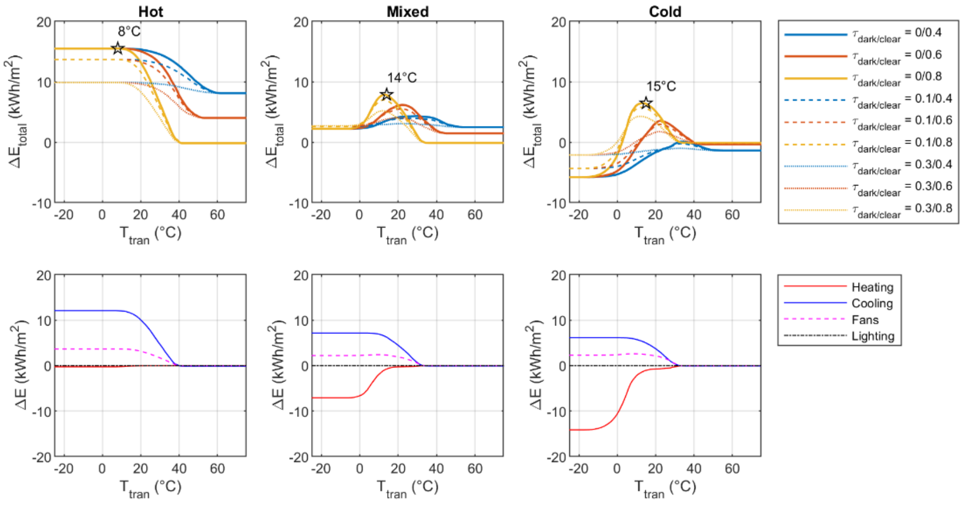

Supplementary Figure 67| Total site energy saving (ΔE_{total}) and sectoral energy saving (ΔE) per conditioned floor area in a mid-rise apartment (DOE prototype building model, four-floor, without lighting control) by TR windows (installed in all the four orientations) as functions of transition temperature (T_{tran}). The optimal τ_{dark} for energy saving is 0, which is the same as that in the model of office space with lighting control.

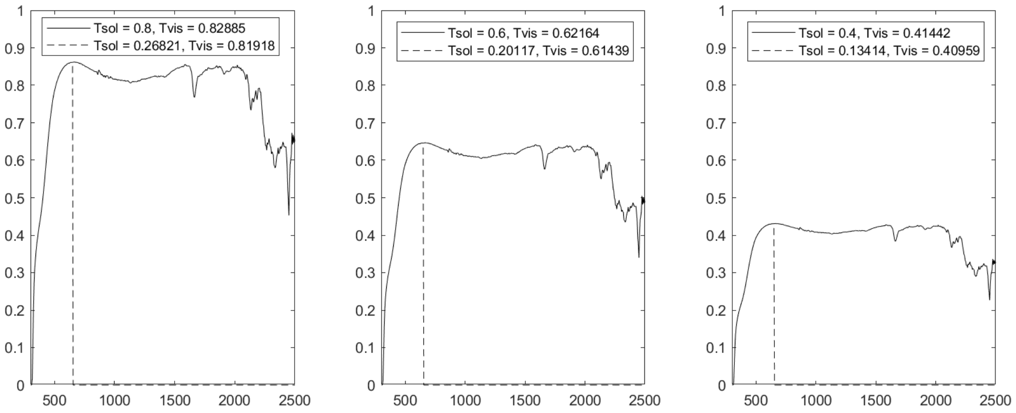

Supplementary Figure 68|Parameterization of spectral solar transmittance of NIR-response TR materials (solid lines indicate clear state, and dash lines indicate dark state). Defining NIR at 655 nm allows for an extra 15% of solar energy to be modulated with only a 1% impact on visible transmittance.

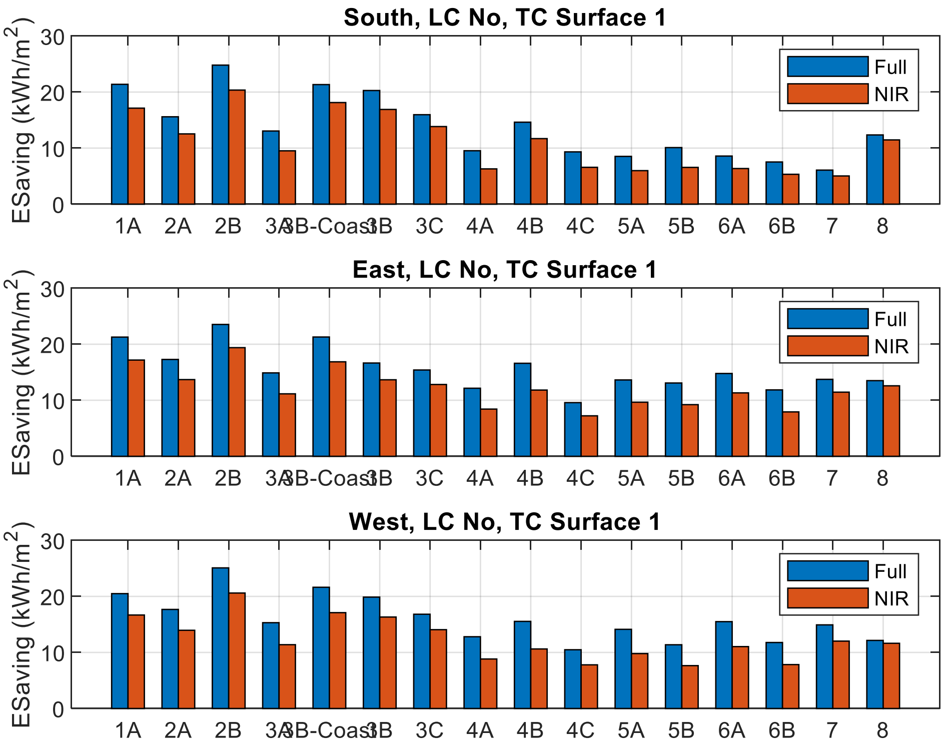

Supplementary Figure 69|Comparison of energy saving by TR windows with full-spectral response (blue) and NIR response (ref) in the buildings without light control (LC No) in three window orientations under 16 U.S. representitive cities for climate classifications[3].

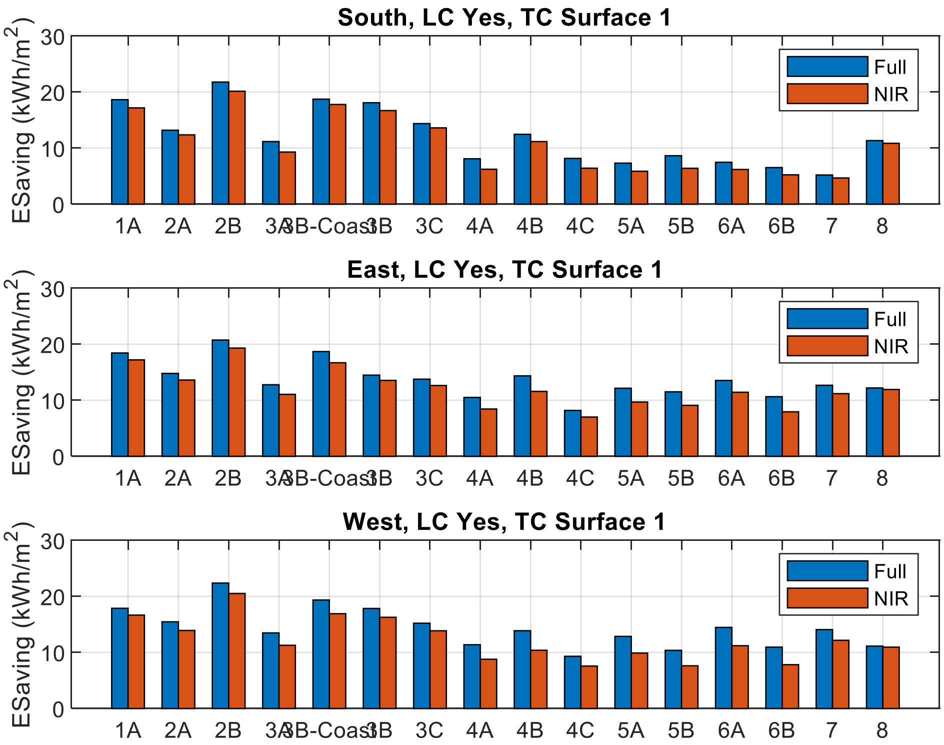

Supplementary Figure 70|Comparison of energy saving by TR windows with full-spectral response (blue) and NIR response (ref) in the buildings with light control (LC Yes) in three window orientations under 16 U.S. representitive cities for climate classifications[3].

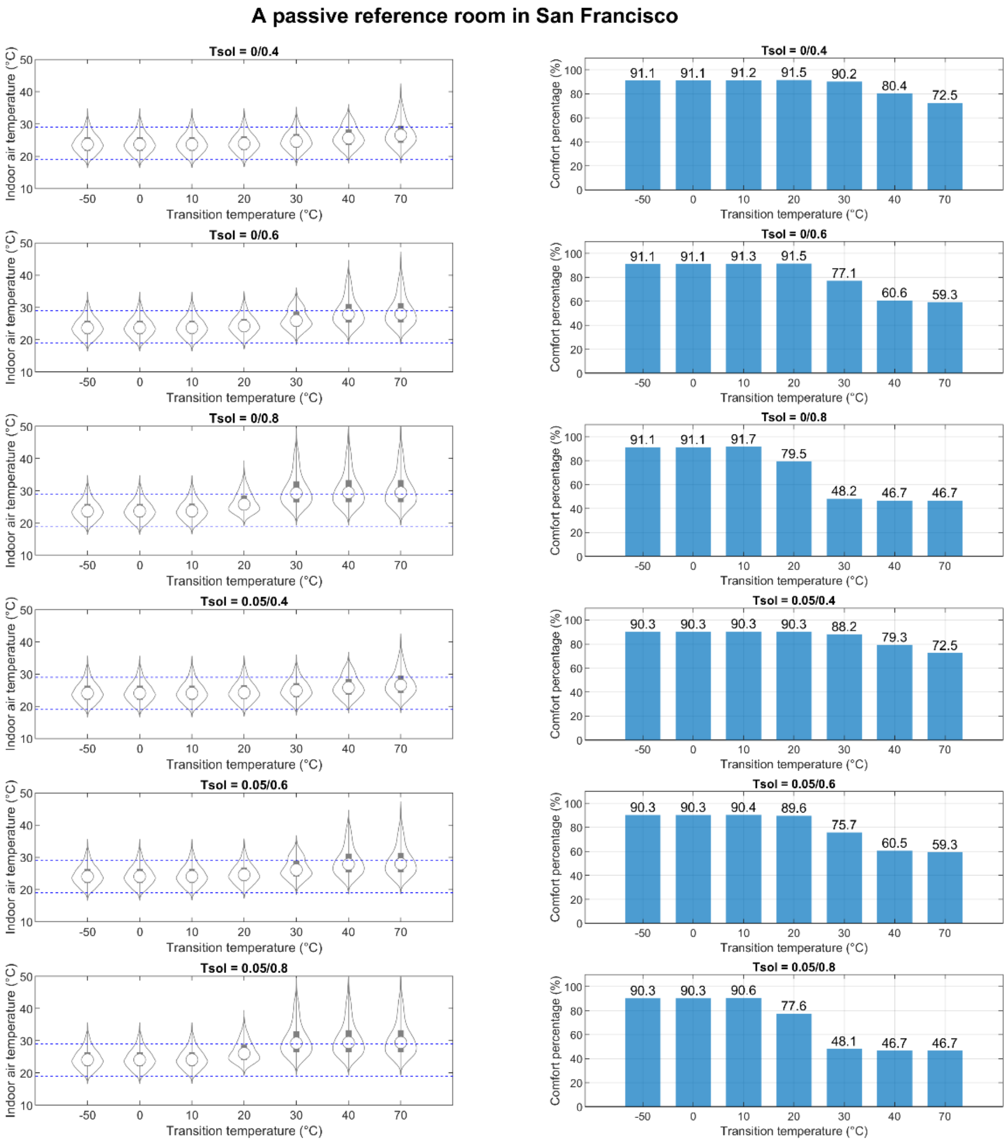

Supplementary Figure 71|Indoor air temperature and comfort percentage as the function of transition temperature of TR windows in a passive reference room in San Francisco.

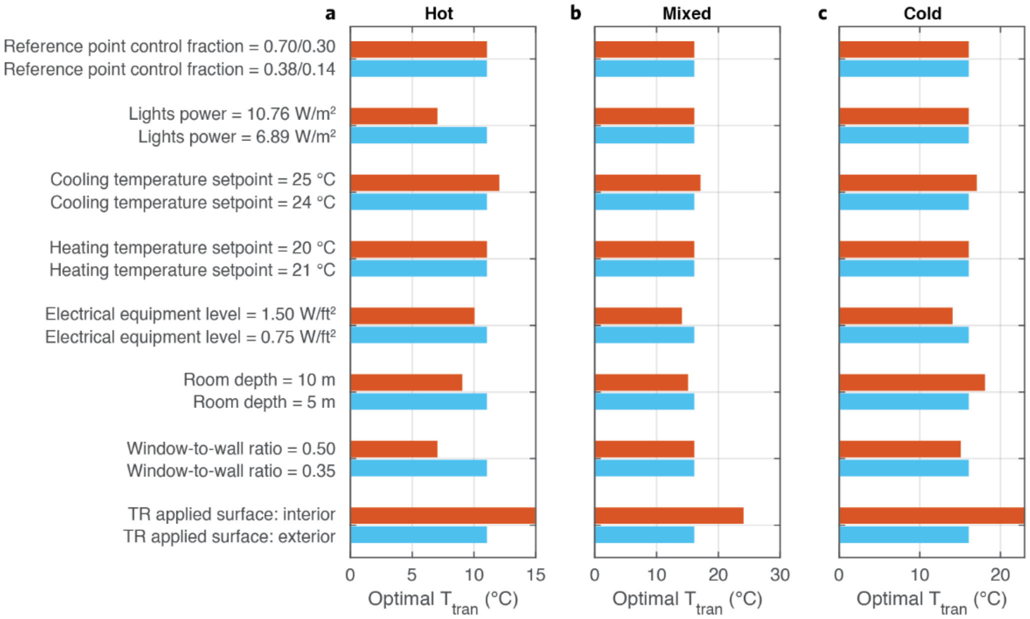

Supplementary Figure 72|Sensitivity analysis of optimal transition temperature of TR windows in hot (a), mixed (b), and cold (c) climate.

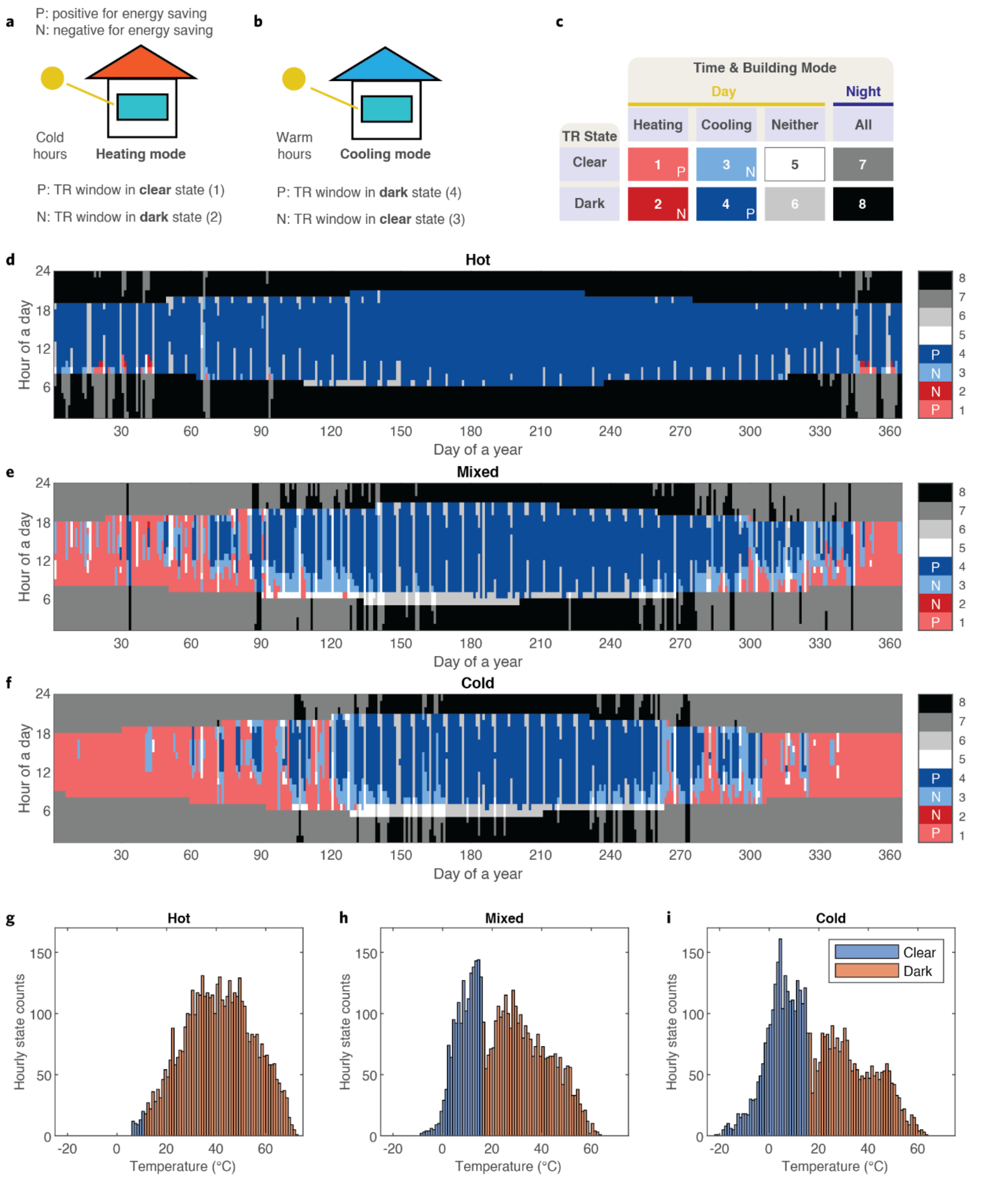

Supplementary Figure 73| Definition of TR window operation states and annual evaluation in hourly resolution. a,b, Definition of beneficial (State 1 & 4, positive for energy saving), detrimental (State 2 & 3, negative for energy saving) states for TR windows in building heating (**a**) and cooling (**b**) modes. **c**, Definition of eight operation states for TR windows. Light and dark color pairs are used to distinguish the clear and dark states in different time and building modes. **d**-**f**, Annual analysis of hourly operation states for TR windows in hot (**d**), mixed (**e**), and cold (**f**) climates, respectively. It indicates a good energy-saving performance for TR windows when there are more beneficial (positive) hours and fewer detrimental (negative) hours during a year. **g**-i, Hourly counts of clear and dark states for TR windows during daytime in hot (**g**), mixed (**h**), and cold (**i**) climates, respectively.

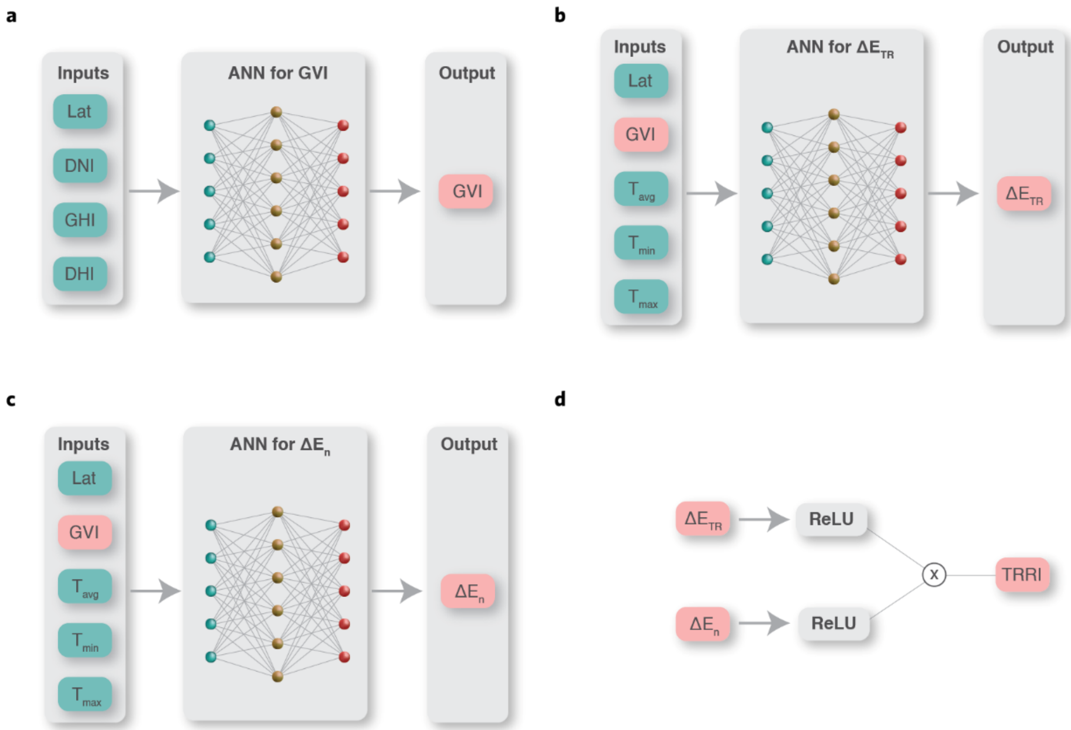

Supplementary Figure 74 | Structures of artificial neural networks (ANNs) for GVI, optimal ΔE_{TR}, and ΔE_n, and the calculation of TRRI. Note here the network structures depicted in the figure do not precisely resemble the actual hidden layers.

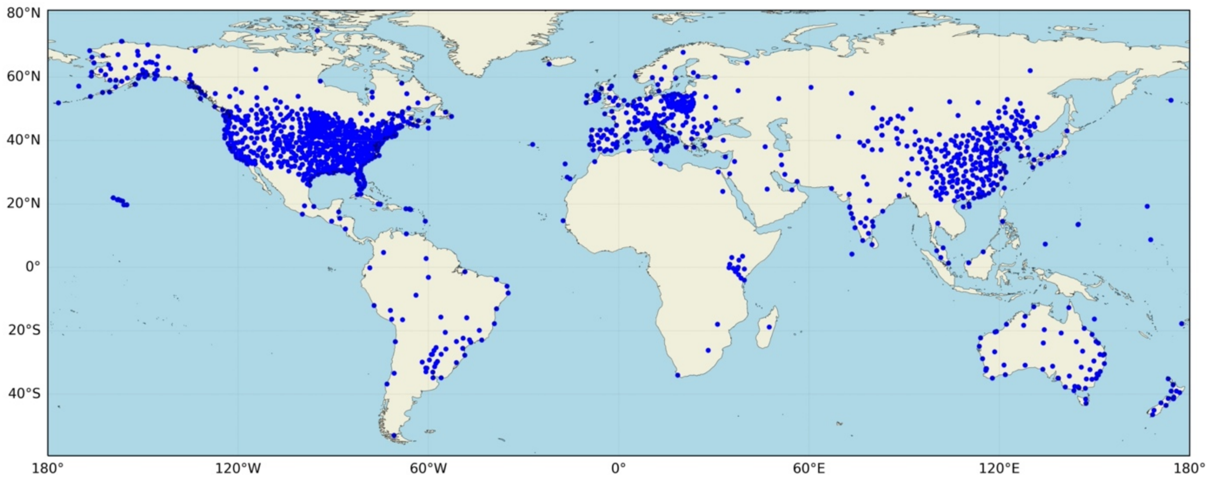

Supplementary Figure 75|Distribution of weather data from over two thousand stations used in this study.

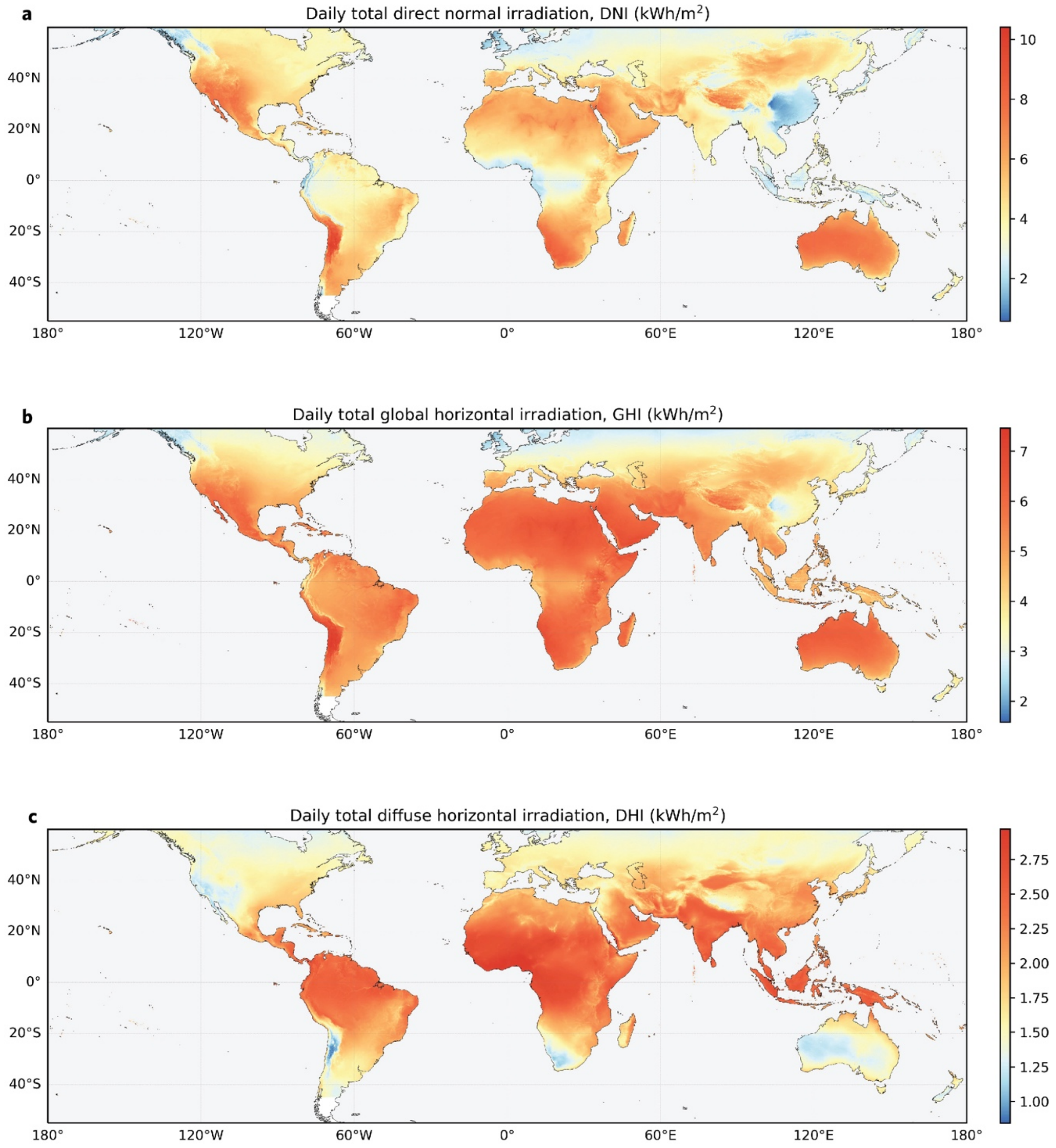

Supplementary Figure 76|World heatmaps of global solar irradiation for ANN inputs[4].

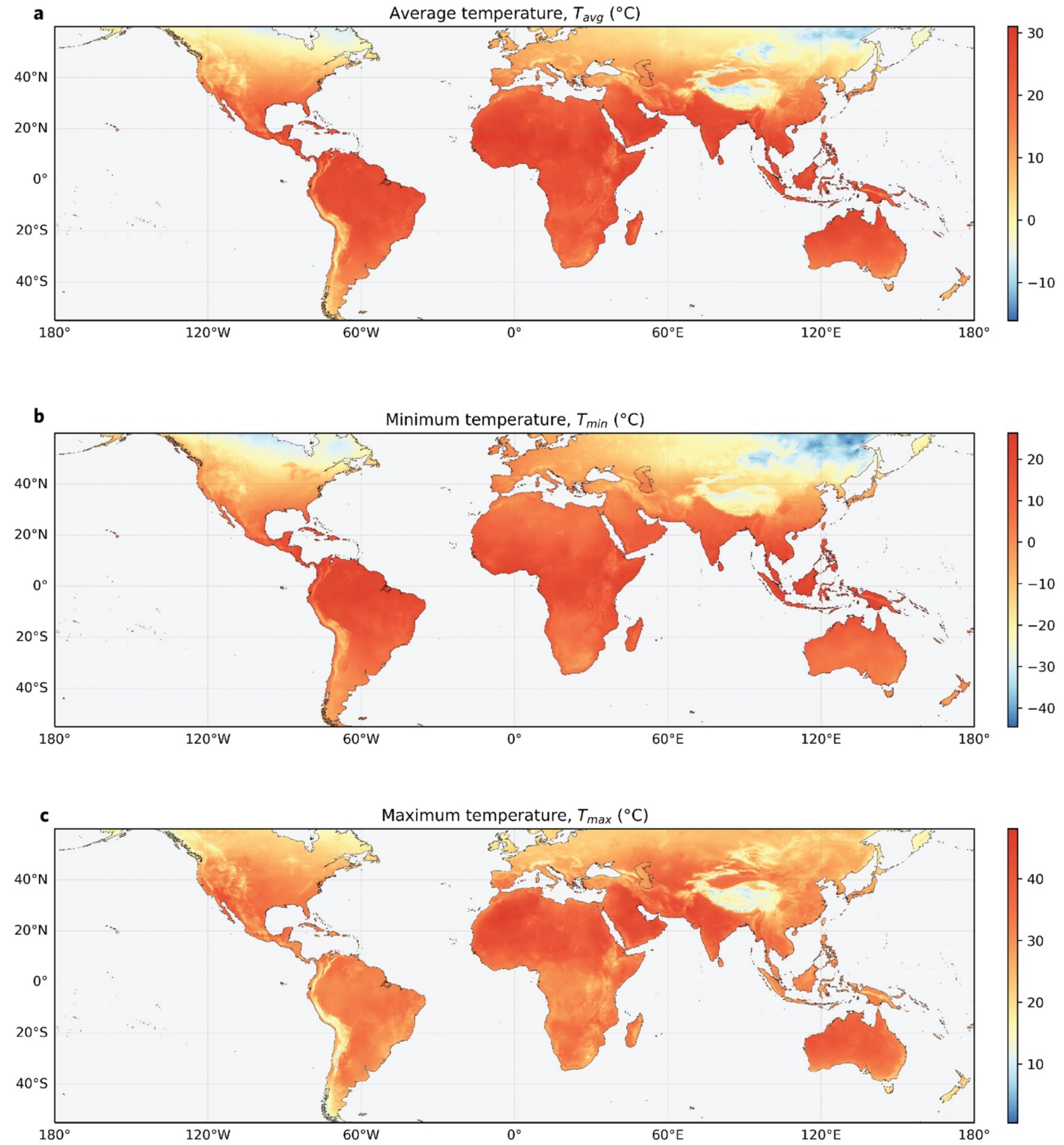

Supplementary Figure 77|World heatmaps of global temperature distribution for ANN inputs[5].

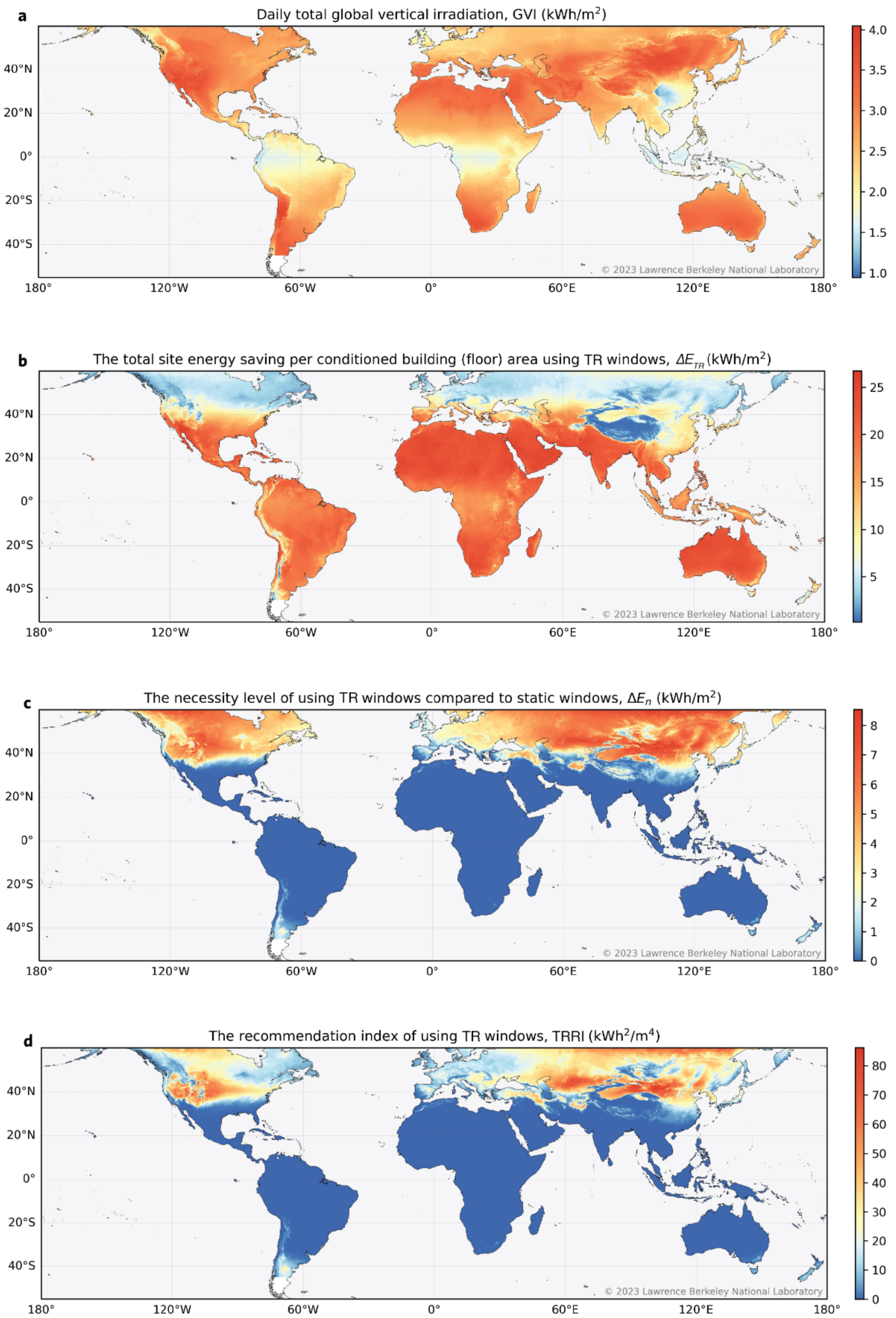

Supplementary Figure 78| World heatmaps of GVI, optimal ΔE_{TR}, ΔE_n, and TRRI generated by artificial neural networks (ANNs).

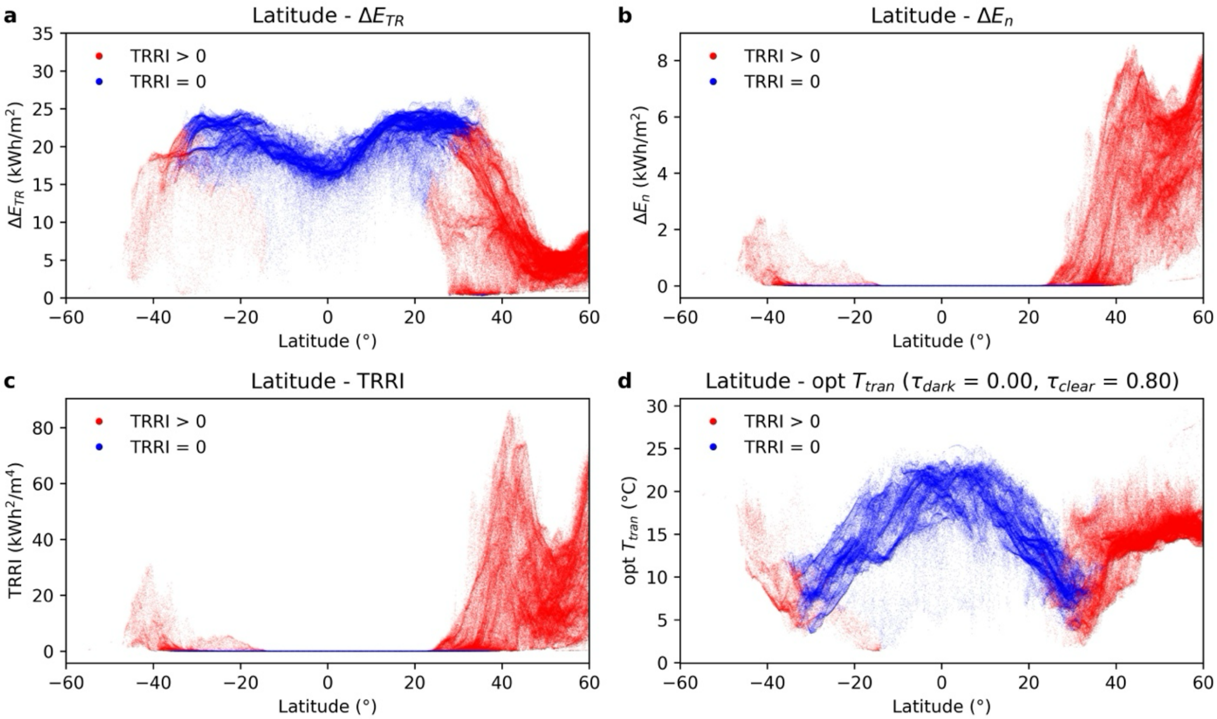

Supplementary Figure 79| Latitude charts of pixel counts in world maps of ΔE_{TR}, ΔE_n, TRRI, and optimal T_{tran} ($\tau_{clear} = 0.8$, and $\tau_{dark} = 0$) when TRRI (or ΔE_n) > 0 (red) and TRRI (or ΔE_n) $= 0$ (blue).

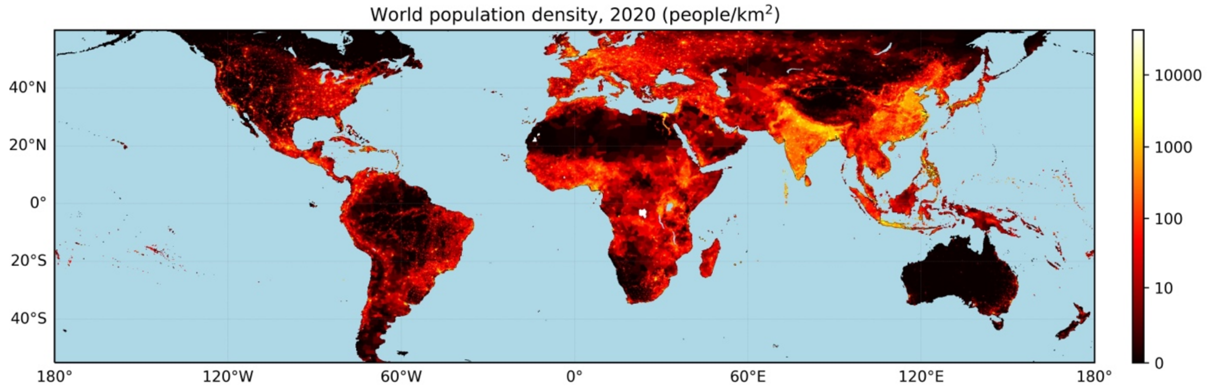

Supplementary Figure 80|World heatmap of populatoin density (year of 2020)[6]**.** Grey areas indicate unavailable data**.**

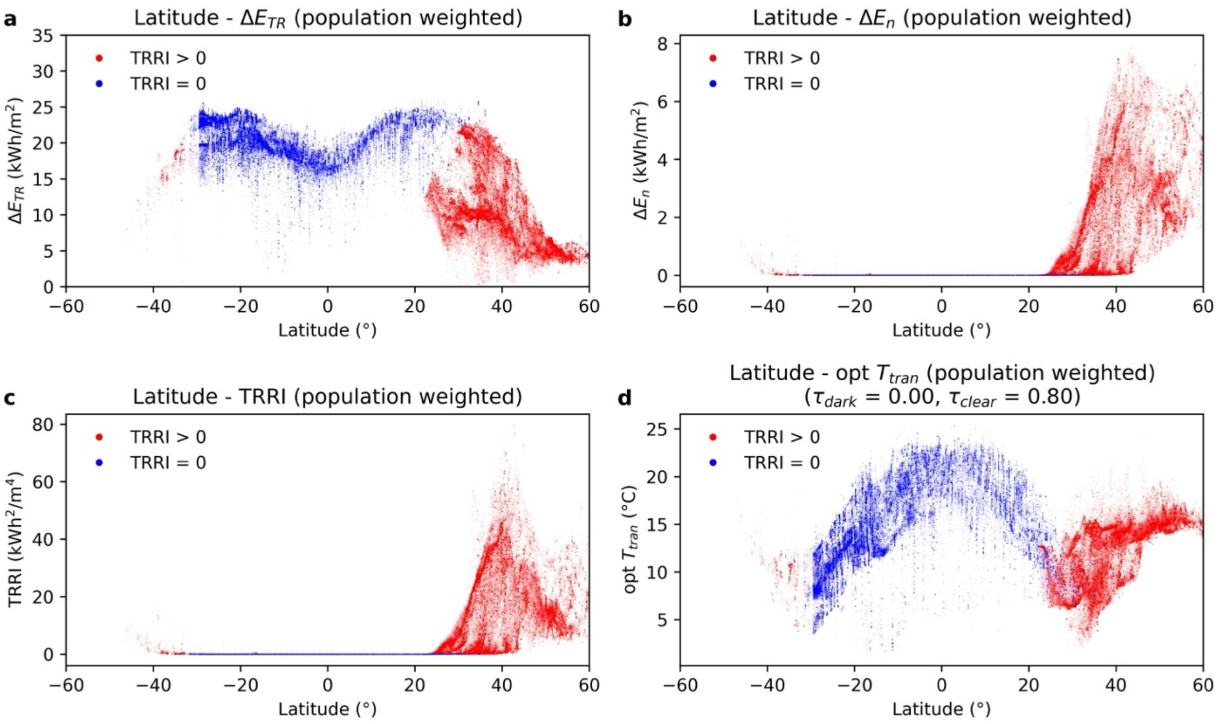

Supplementary Figure 81| Latitude charts of population density-weighted pixel counts in world maps of ΔE_{TR}, ΔE_n, TRRI, and optimal T_{tran} ($\tau_{clear} = 0.8$, and $\tau_{dark} = 0$) when TRRI (or ΔE_n) > 0 (red) and TRRI (or ΔE_n) $= 0$ (blue).

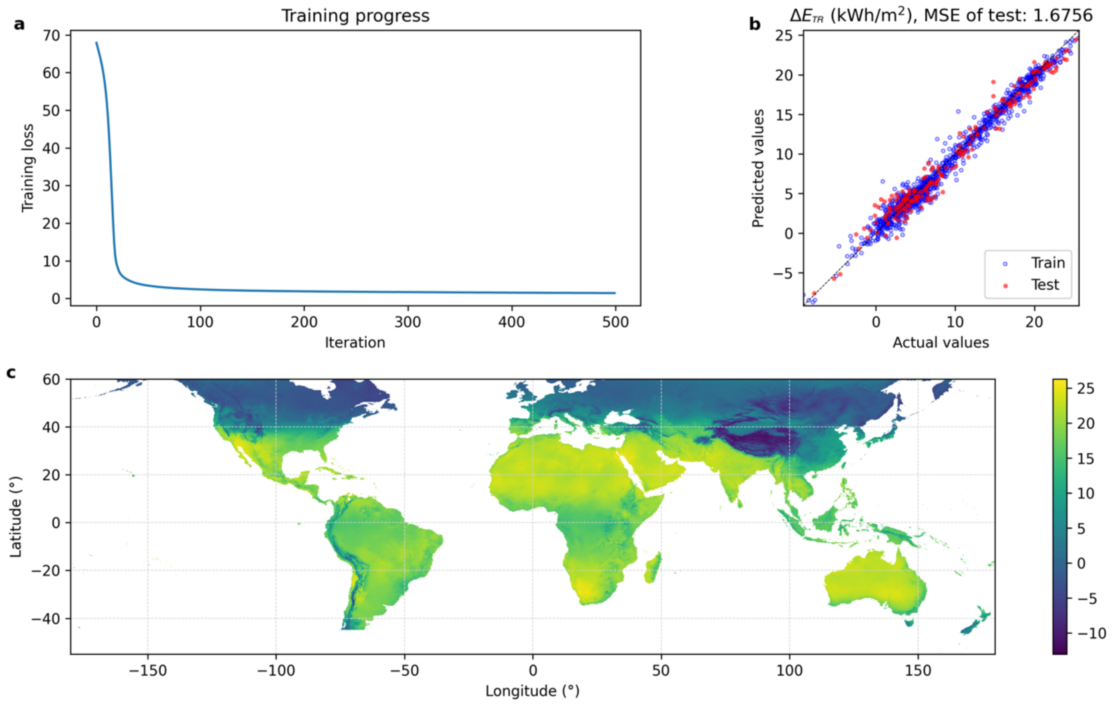

Supplementary Figure 82| ANN training progress, training and testing results, and world heatmap of ΔE_{TR} when $\tau_{clear} = 0.4$, and $\tau_{dark} = 0$. Note that the world map is not a geographically accurate representation with a proper projection, but rather a visual plot of array data.

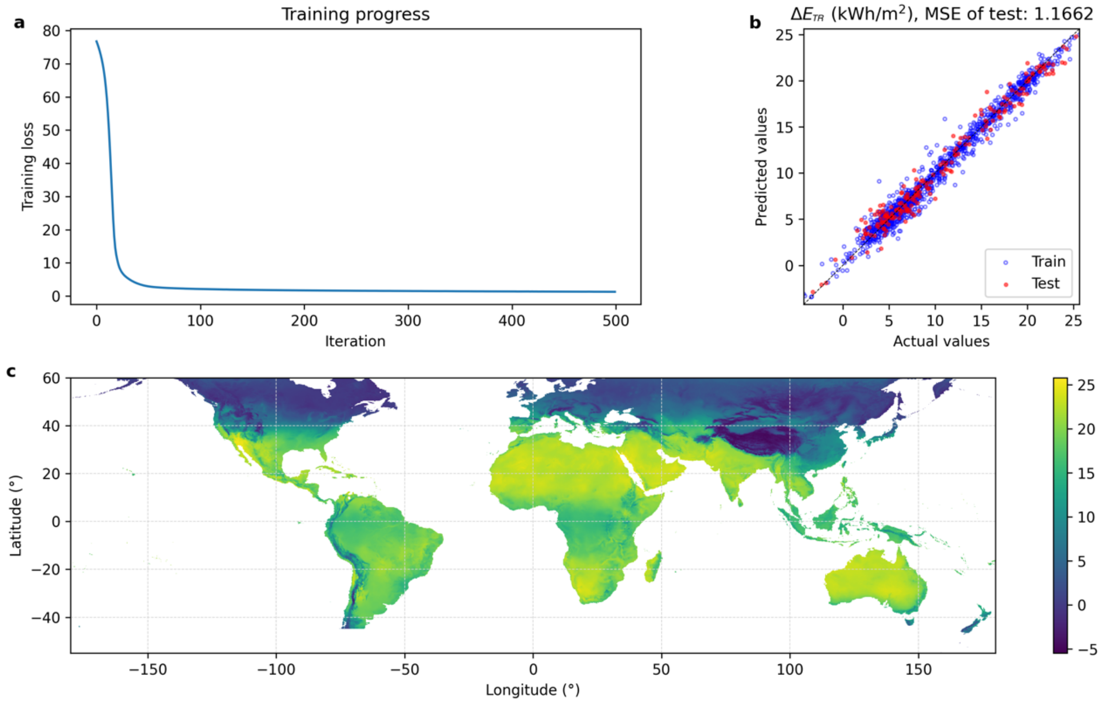

Supplementary Figure 83| ANN training progress, training and testing results, and world heatmap of ΔE_{TR} when $\tau_{clear} = 0.6$, and $\tau_{dark} = 0$. Note that the world map is not a geographically accurate representation with a proper projection, but rather a visual plot of array data.

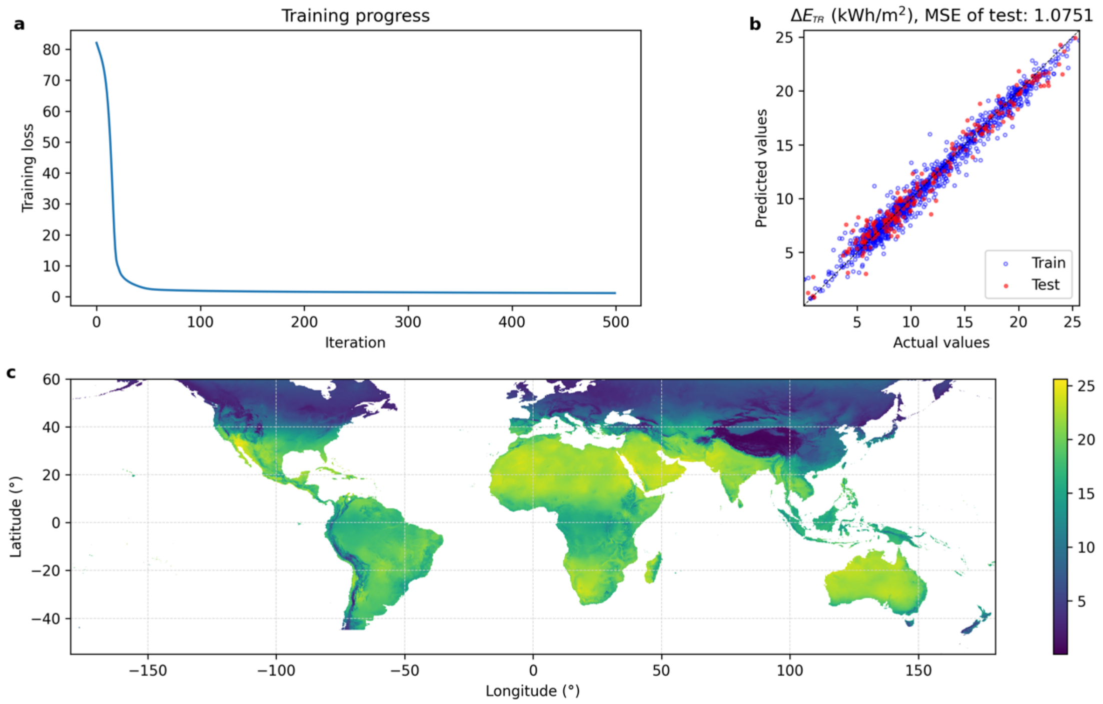

Supplementary Figure 84| ANN training progress, training and testing results, and world heatmap of ΔE_{TR} when $\tau_{clear} = 0.8$, and $\tau_{dark} = 0$. Note that the world map is not a geographically accurate representation with a proper projection, but rather a visual plot of array data.

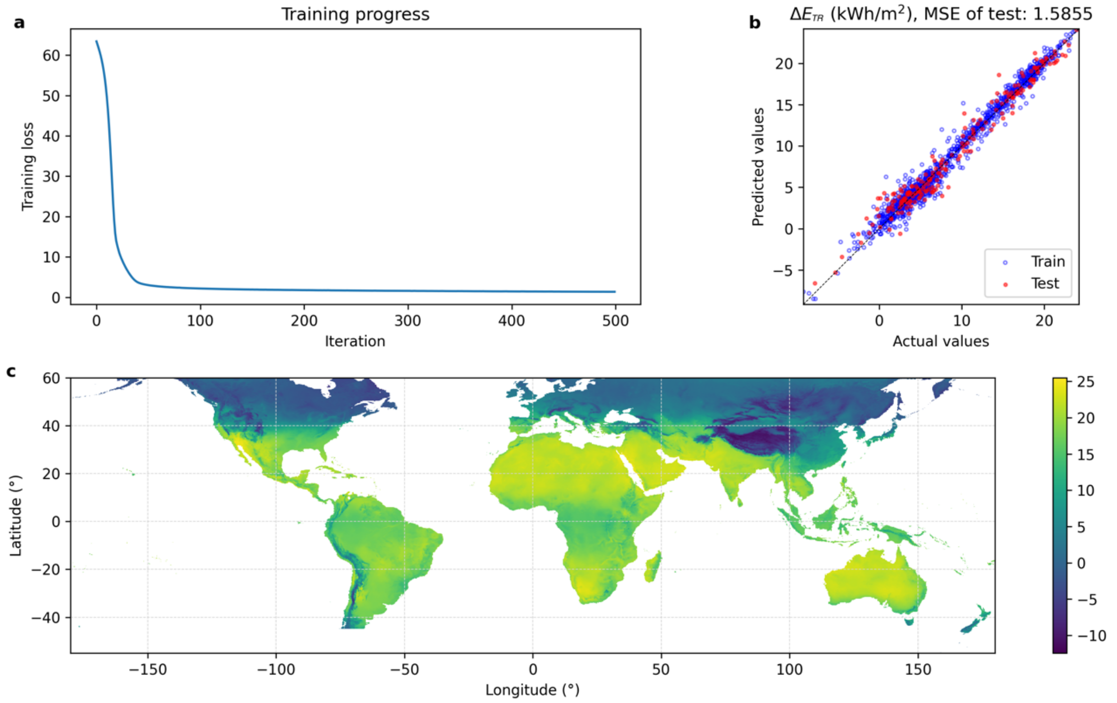

Supplementary Figure 85| ANN training progress, training and testing results, and world heatmap of ΔE_{TR} when $\tau_{clear} = 0.4$, and $\tau_{dark} = 0.05$. Note that the world map is not a geographically accurate representation with a proper projection, but rather a visual plot of array data.

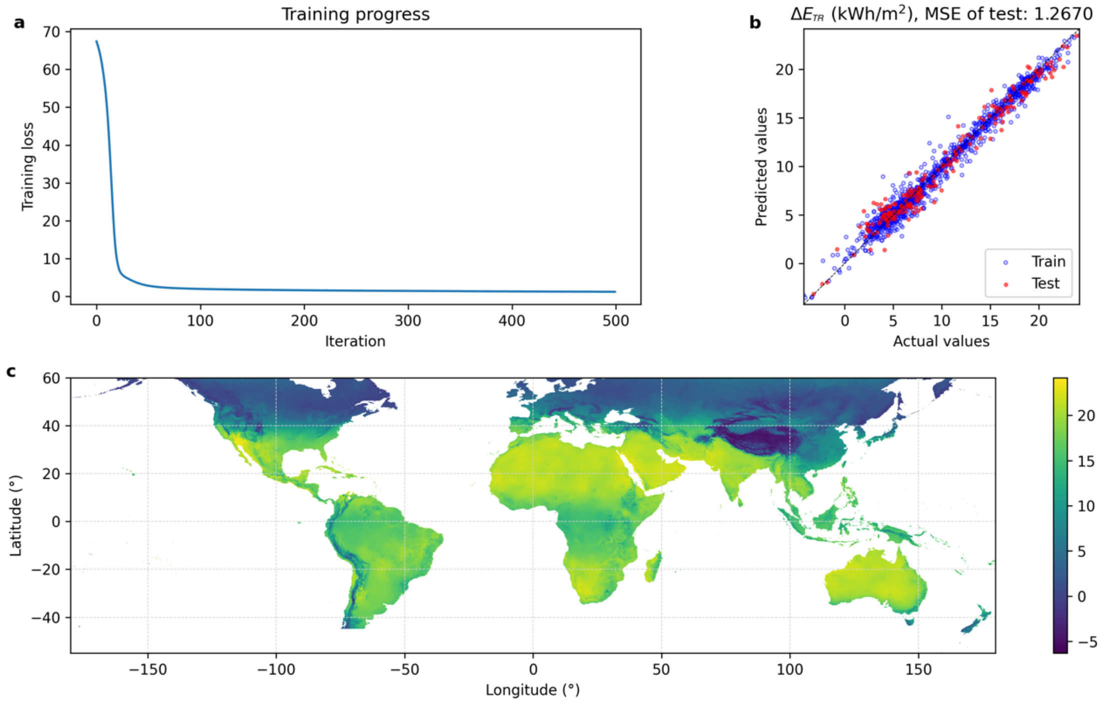

Supplementary Figure 86| ANN training progress, training and testing results, and world heatmap of ΔE_{TR} when $\tau_{clear} = 0.6$, and $\tau_{dark} = 0.05$. Note that the world map is not a geographically accurate representation with a proper projection, but rather a visual plot of array data.

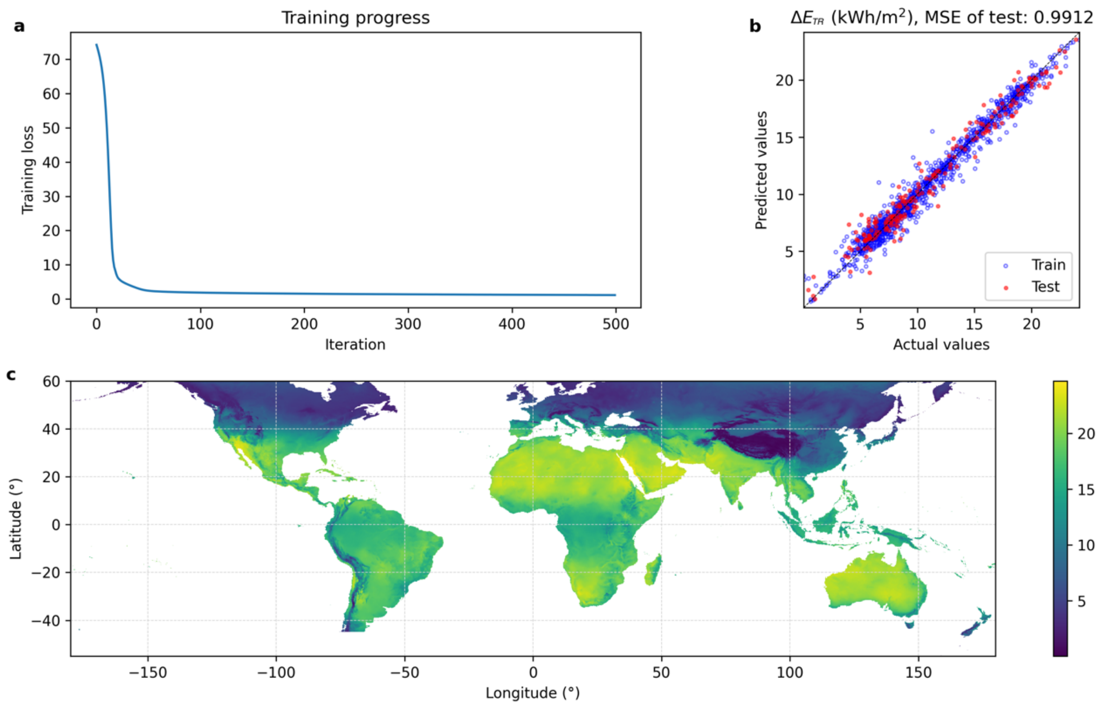

Supplementary Figure 87| ANN training progress, training and testing results, and world heatmap of ΔE_{TR} when $\tau_{clear} = 0.8$, and $\tau_{dark} = 0.05$. Note that the world map is not a geographically accurate representation with a proper projection, but rather a visual plot of array data.

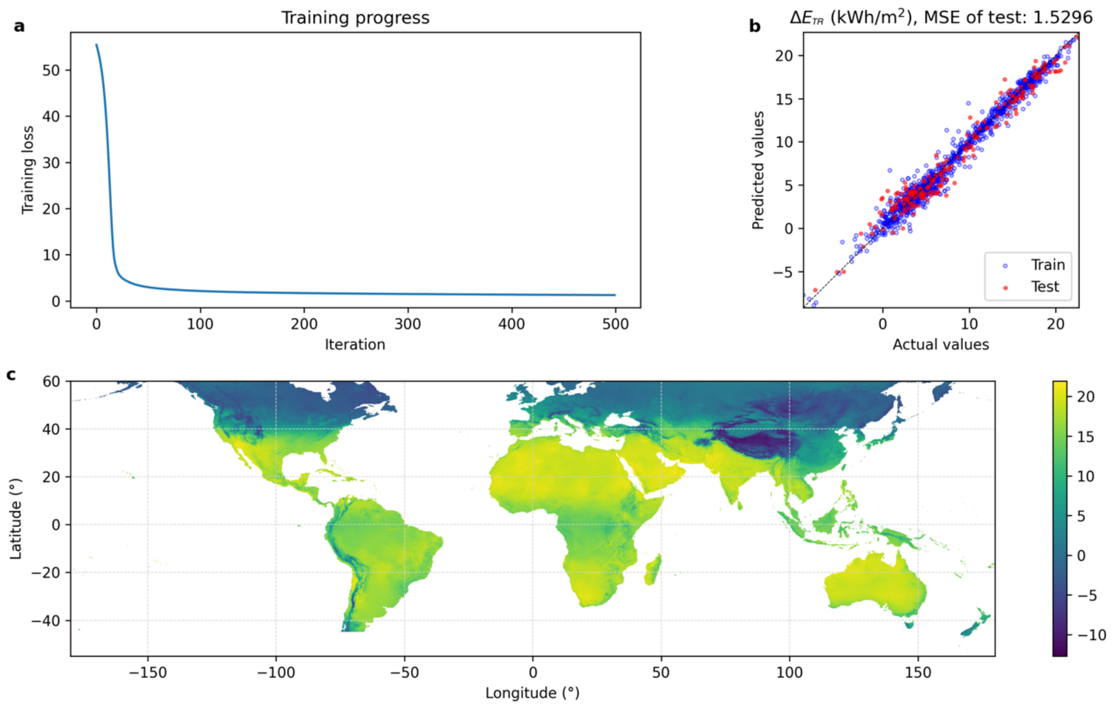

Supplementary Figure 88| ANN training progress, training and testing results, and world heatmap of ΔE_{TR} when $\tau_{clear} = 0.4$, and $\tau_{dark} = 0.1$. Note that the world map is not a geographically accurate representation with a proper projection, but rather a visual plot of array data.

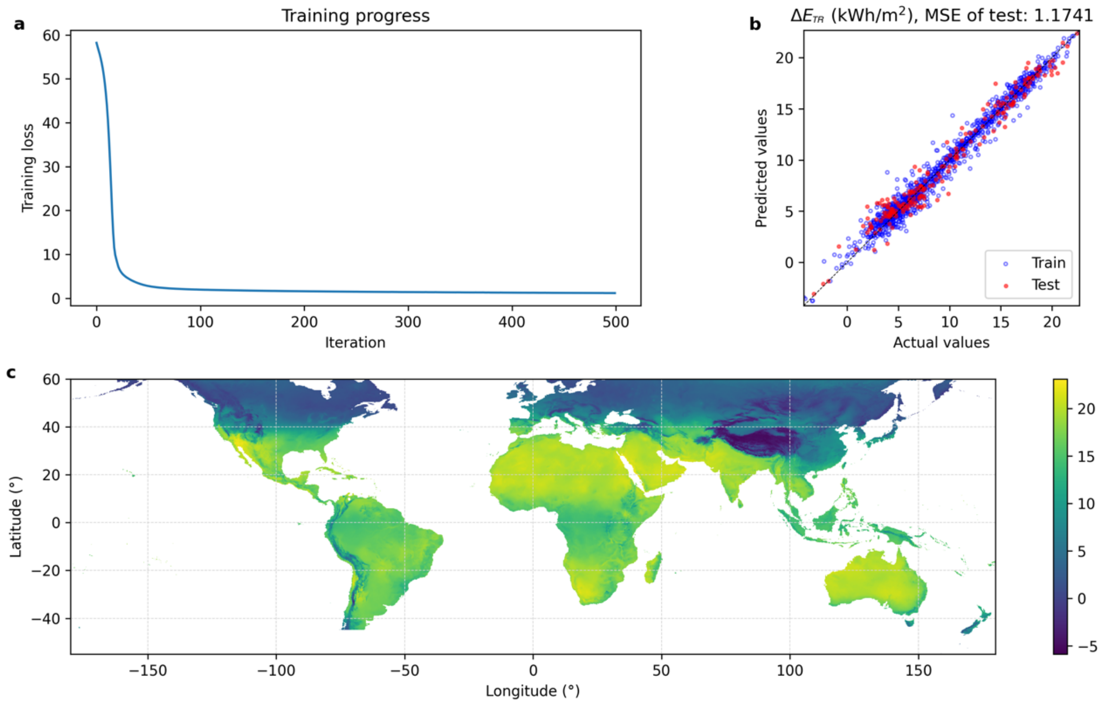

Supplementary Figure 89|ANN training progress, training and testing results, and world heatmap of ΔE_{TR} when $\tau_{clear} = 0.6$, and $\tau_{dark} = 0.1$. Note that the world map is not a geographically accurate representation with a proper projection, but rather a visual plot of array data.

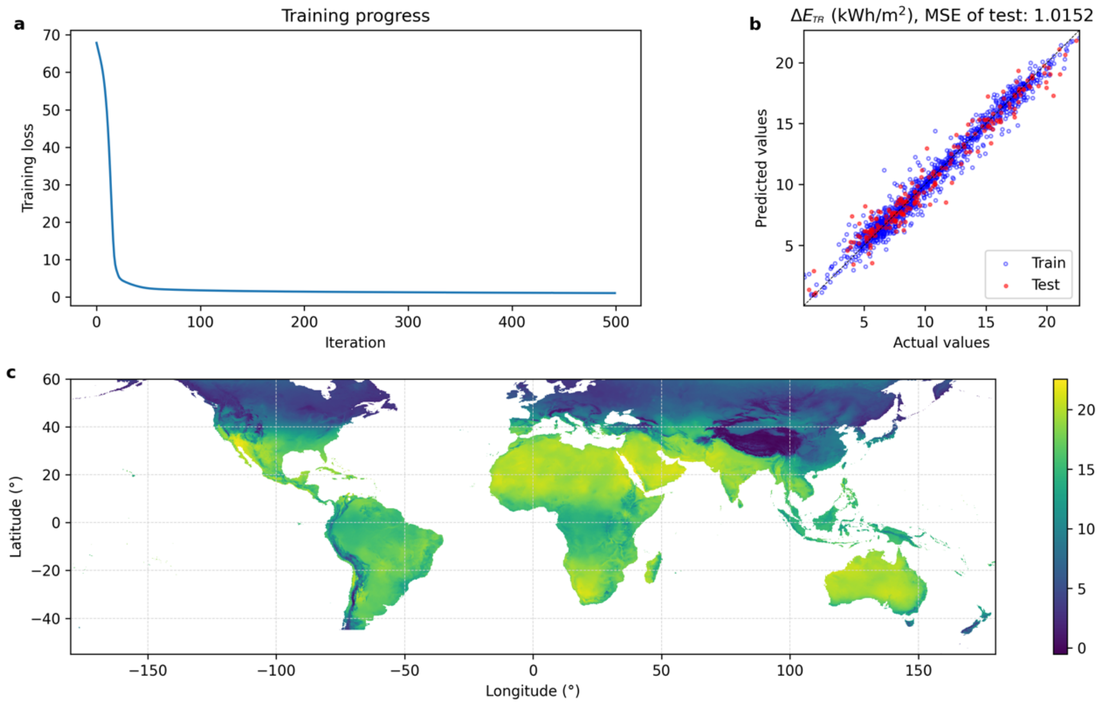

Supplementary Figure 90| ANN training progress, training and testing results, and world heatmap of ΔE_{TR} when $\tau_{clear} = 0.8$, and $\tau_{dark} = 0.1$. Note that the world map is not a geographically accurate representation with a proper projection, but rather a visual plot of array data.

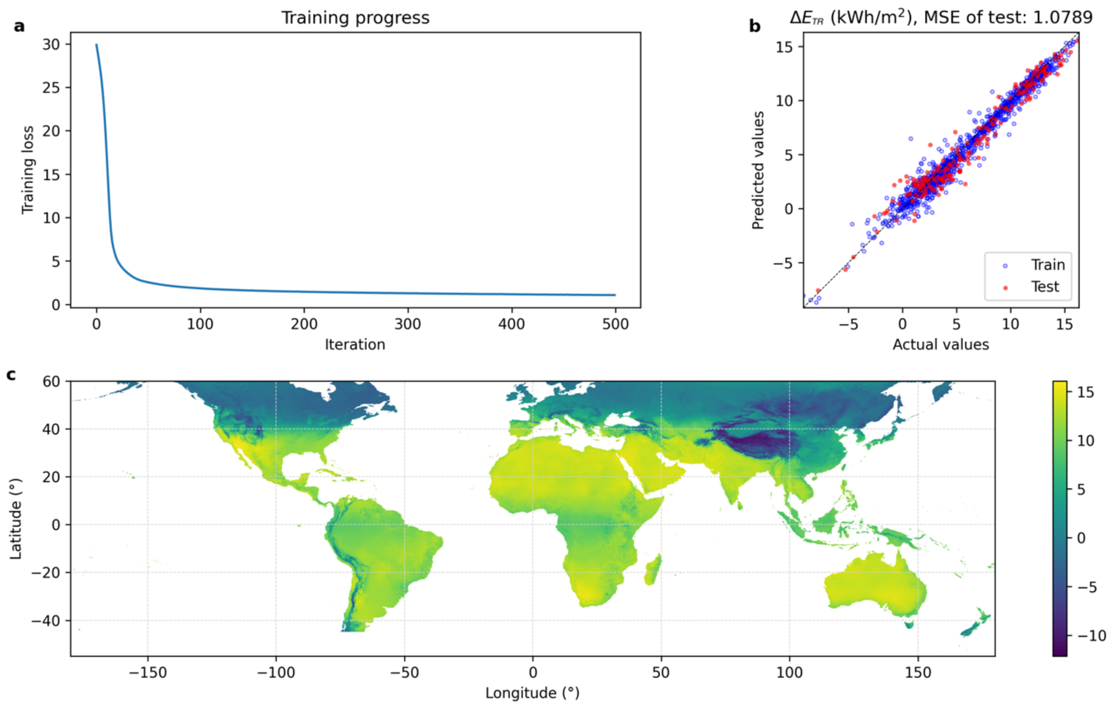

Supplementary Figure 91| ANN training progress, training and testing results, and world heatmap of ΔE_{TR} when $\tau_{clear} = 0.4$, and $\tau_{dark} = 0.3$. Note that the world map is not a geographically accurate representation with a proper projection, but rather a visual plot of array data.

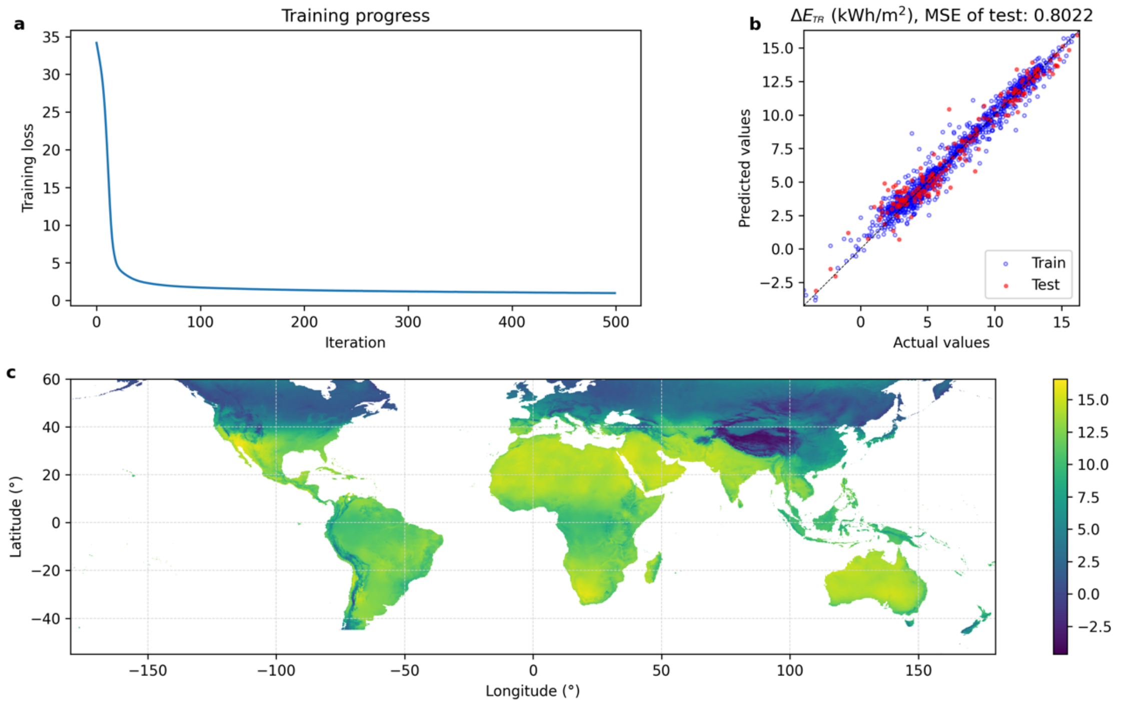

Supplementary Figure 92| ANN training progress, training and testing results, and world heatmap of ΔE_{TR} when $\tau_{clear} = 0.6$, and $\tau_{dark} = 0.3$. Note that the world map is not a geographically accurate representation with a proper projection, but rather a visual plot of array data.

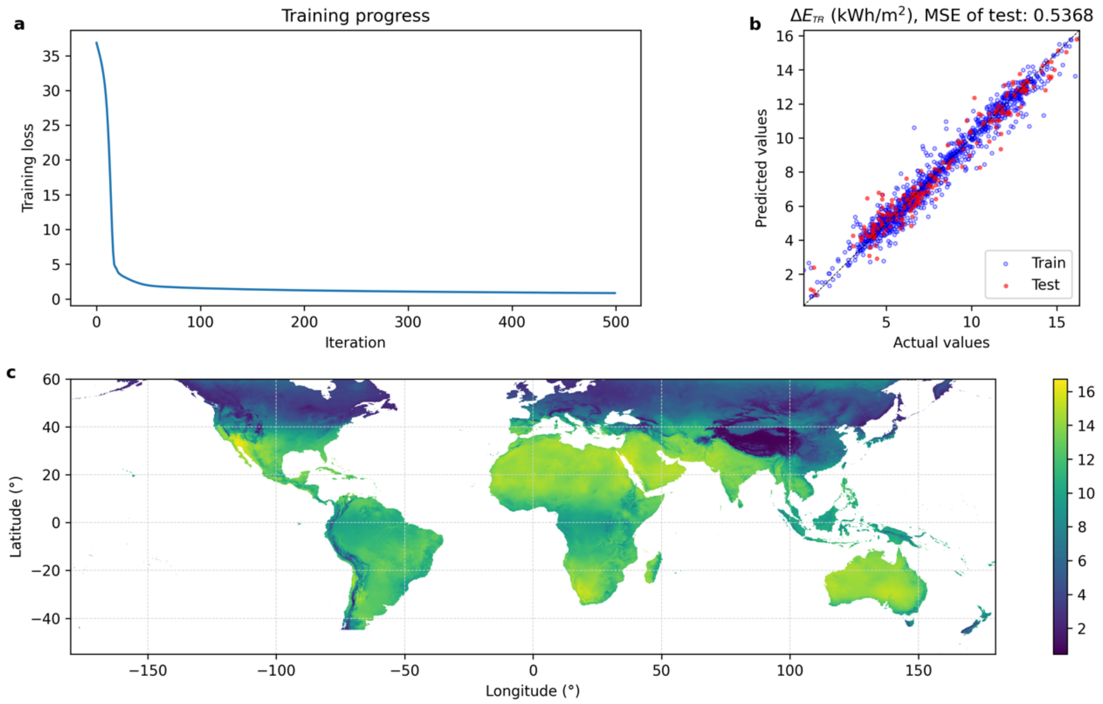

Supplementary Figure 93| ANN training progress, training and testing results, and world heatmap of ΔE_{TR} when $\tau_{clear} = 0.8$, and $\tau_{dark} = 0.3$. Note that the world map is not a geographically accurate representation with a proper projection, but rather a visual plot of array data.

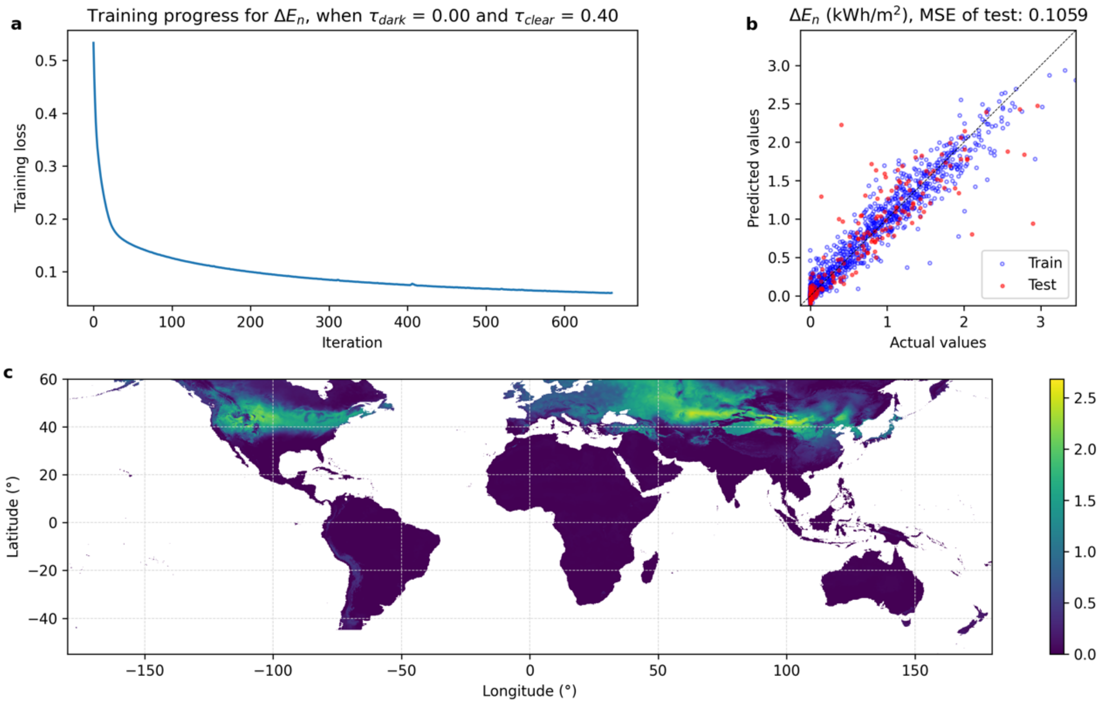

Supplementary Figure 94| ANN training progress, training and testing results, and world heatmap of ΔE_n when $\tau_{clear} = 0.4$, and $\tau_{dark} = 0$. Note that the world map is not a geographically accurate representation with a proper projection, but rather a visual plot of array data.

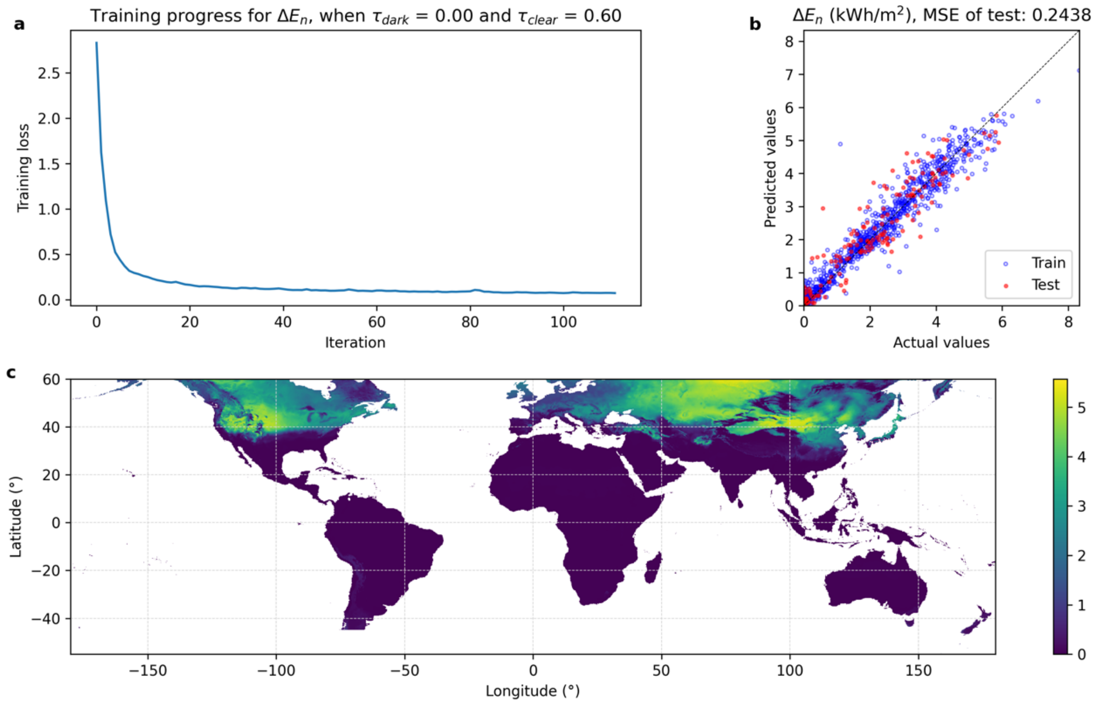

Supplementary Figure 95| ANN training progress, training and testing results, and world heatmap of ΔE_n when $\tau_{clear} = 0.6$, and $\tau_{dark} = 0$. Note that the world map is not a geographically accurate representation with a proper projection, but rather a visual plot of array data.

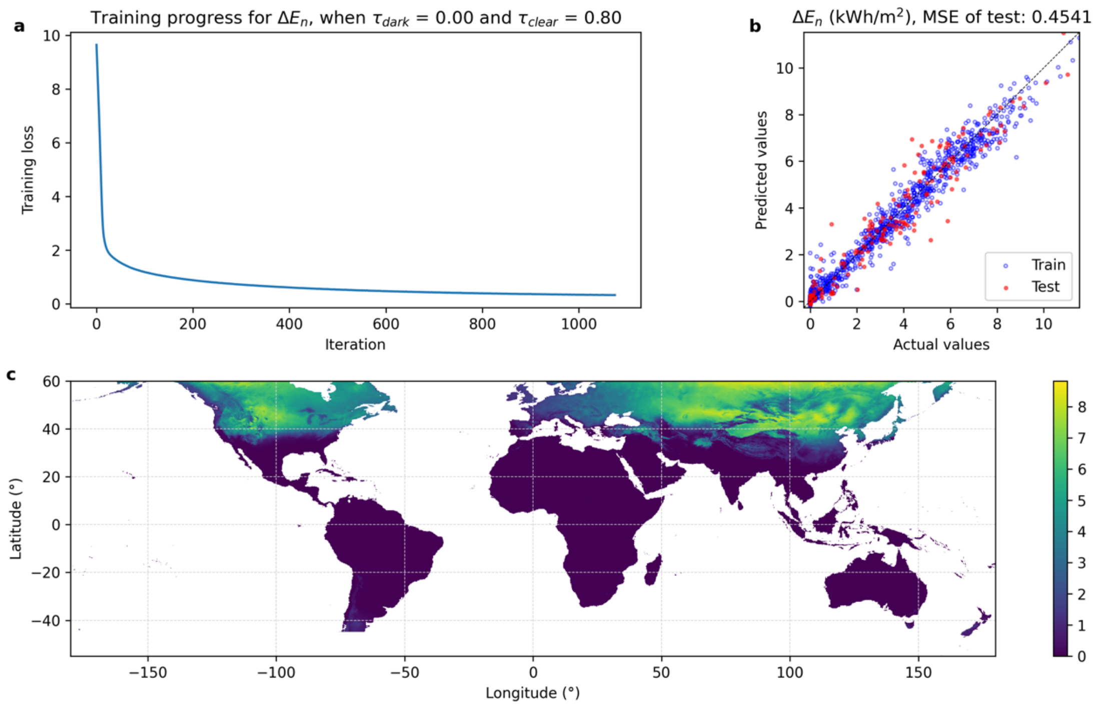

Supplementary Figure 96| ANN training progress, training and testing results, and world heatmap of ΔE_n when $\tau_{clear} = 0.8$, and $\tau_{dark} = 0$. Note that the world map is not a geographically accurate representation with a proper projection, but rather a visual plot of array data.

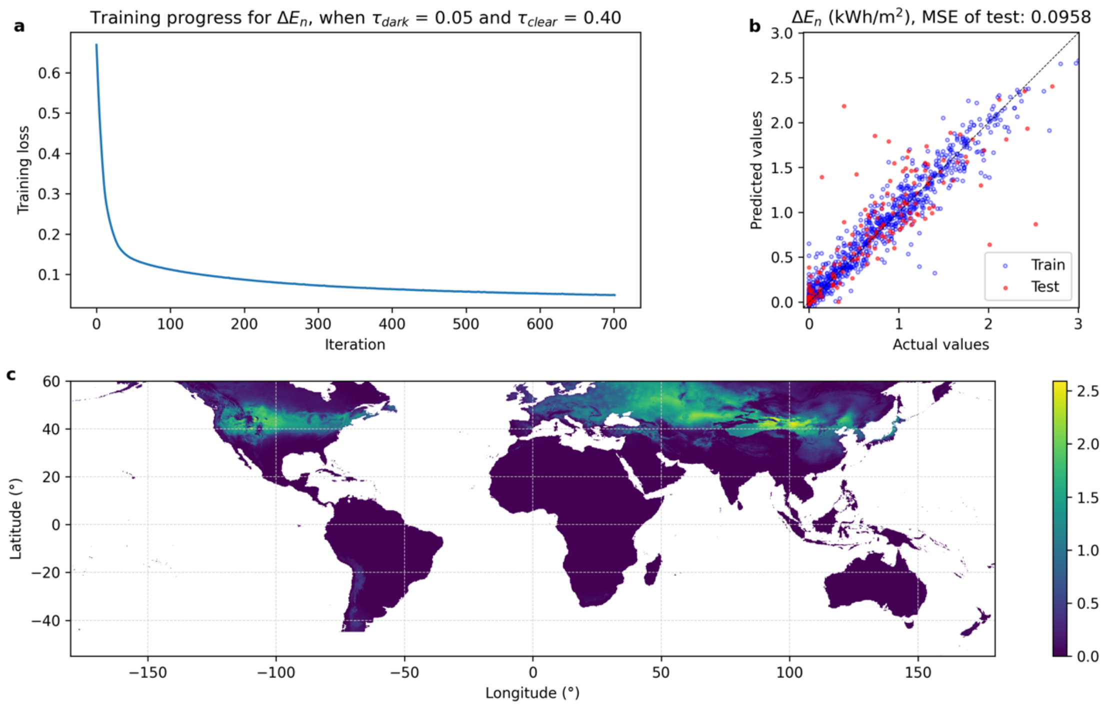

Supplementary Figure 97| ANN training progress, training and testing results, and world heatmap of ΔE_n when $\tau_{clear} = 0.4$, and $\tau_{dark} = 0.05$. Note that the world map is not a geographically accurate representation with a proper projection, but rather a visual plot of array data.

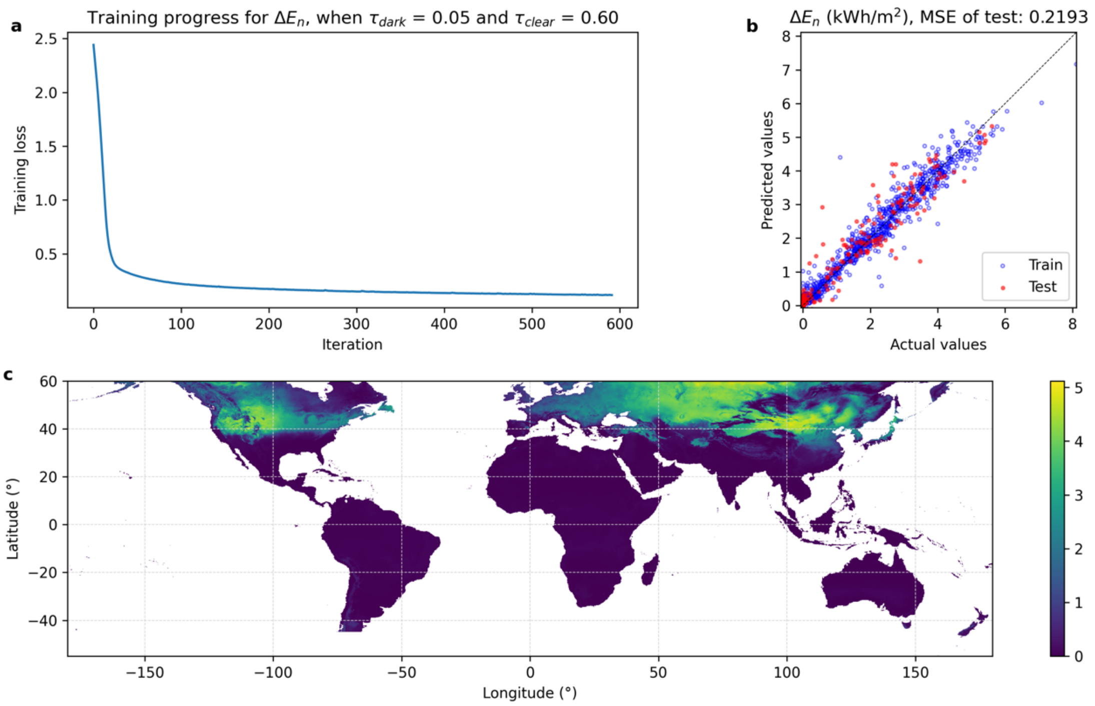

Supplementary Figure 98| ANN training progress, training and testing results, and world heatmap of ΔE_n when $\tau_{clear} = 0.6$, and $\tau_{dark} = 0.05$. Note that the world map is not a geographically accurate representation with a proper projection, but rather a visual plot of array data.

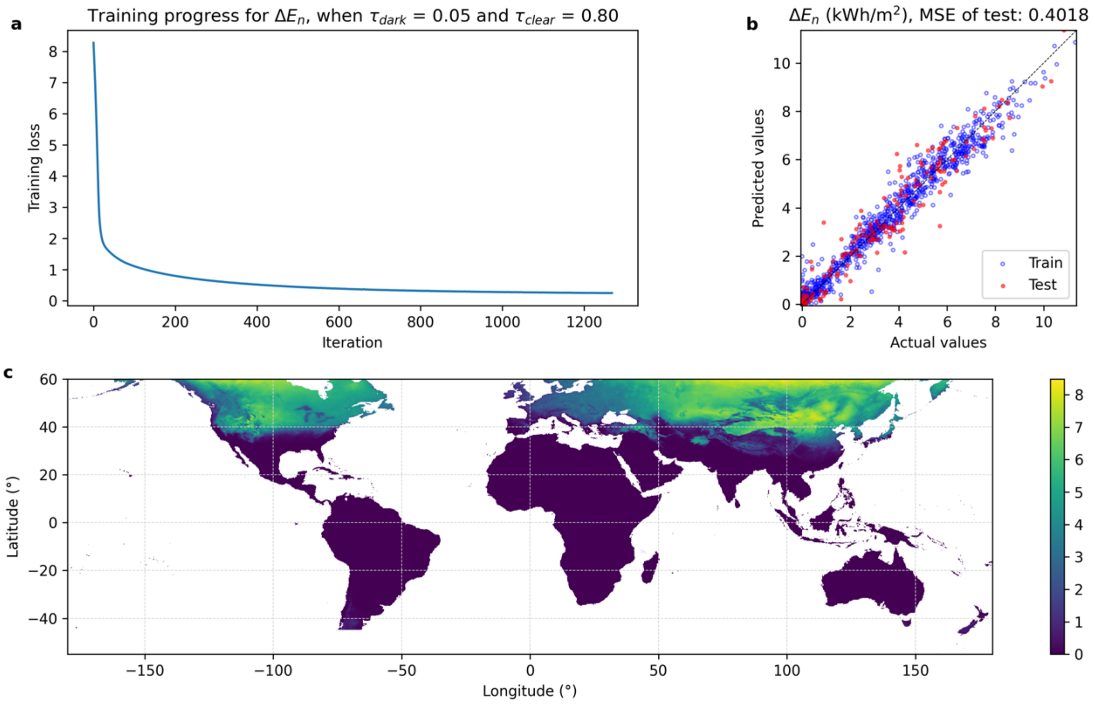

Supplementary Figure 99| ANN training progress, training and testing results, and world heatmap of ΔE_n when $\tau_{clear} = 0.8$, and $\tau_{dark} = 0.05$. Note that the world map is not a geographically accurate representation with a proper projection, but rather a visual plot of array data.

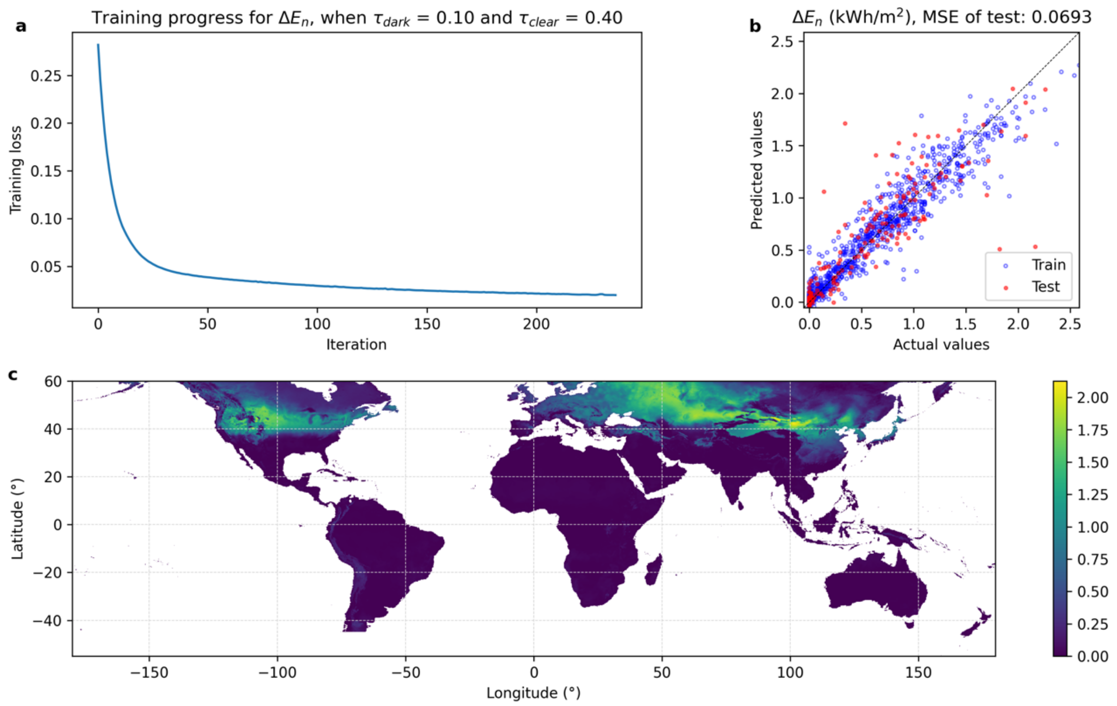

Supplementary Figure 100| ANN training progress, training and testing results, and world heatmap of ΔE_n when $\tau_{clear} = 0.4$, and $\tau_{dark} = 0.1$. Note that the world map is not a geographically accurate representation with a proper projection, but rather a visual plot of array data.

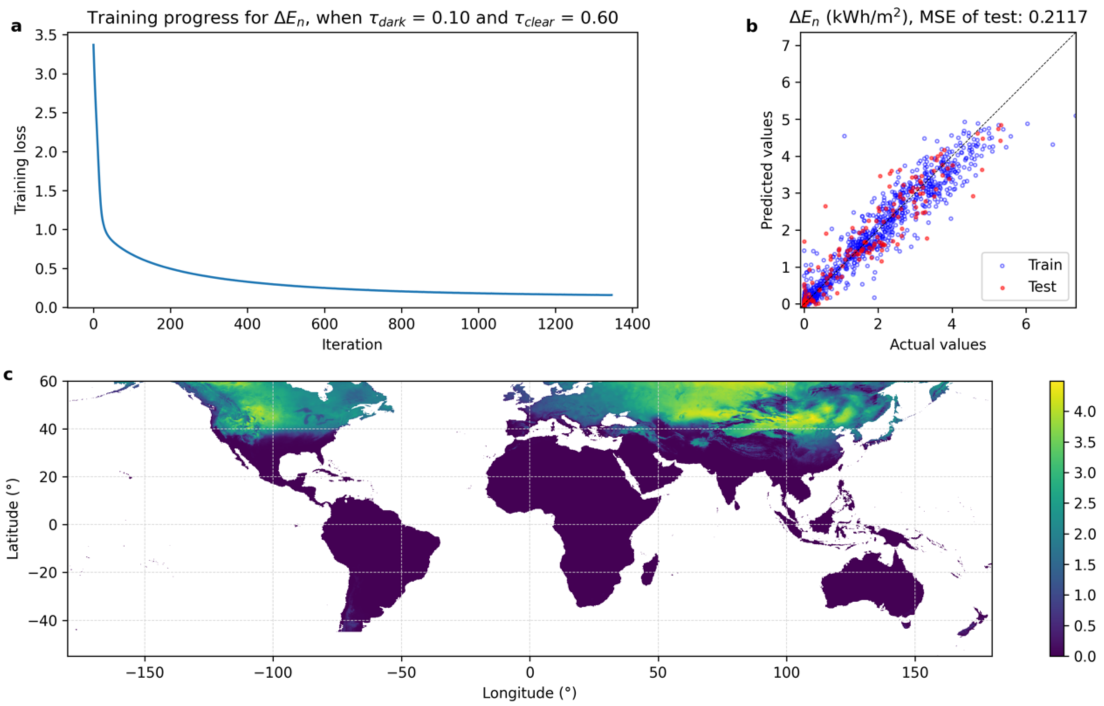

Supplementary Figure 101| ANN training progress, training and testing results, and world heatmap of ΔE_n when $\tau_{clear} = 0.6$, and $\tau_{dark} = 0.1$. Note that the world map is not a geographically accurate representation with a proper projection, but rather a visual plot of array data.

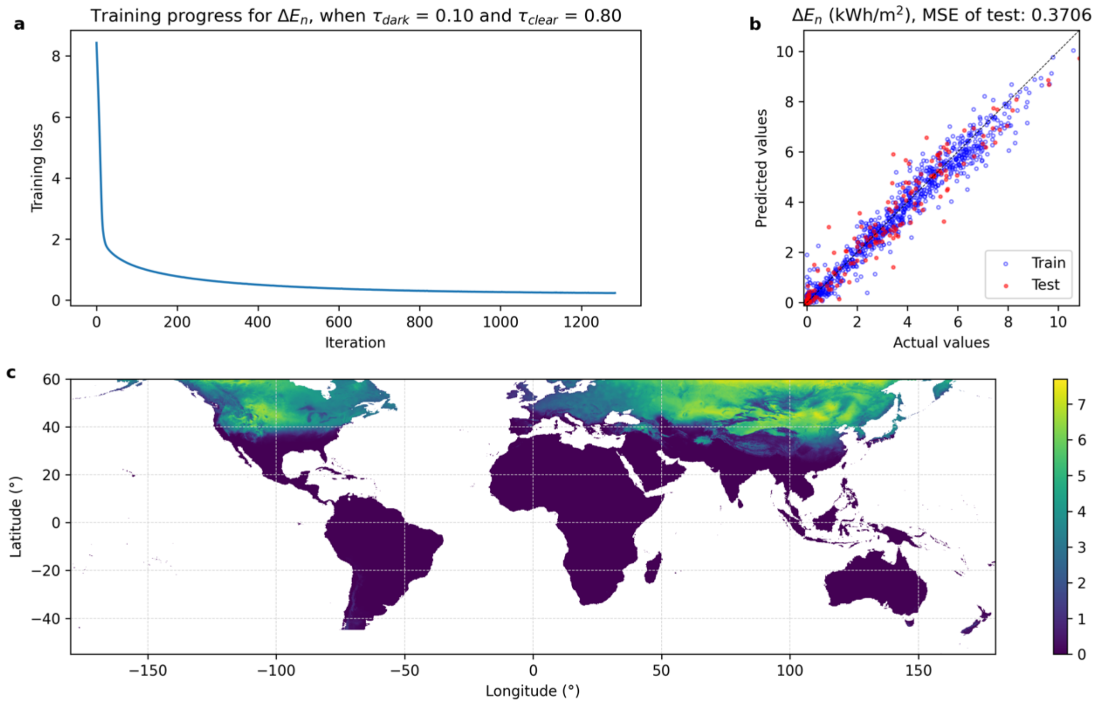

Supplementary Figure 102| ANN training progress, training and testing results, and world heatmap of ΔE_n when $\tau_{clear} = 0.8$, and $\tau_{dark} = 0.1$. Note that the world map is not a geographically accurate representation with a proper projection, but rather a visual plot of array data.

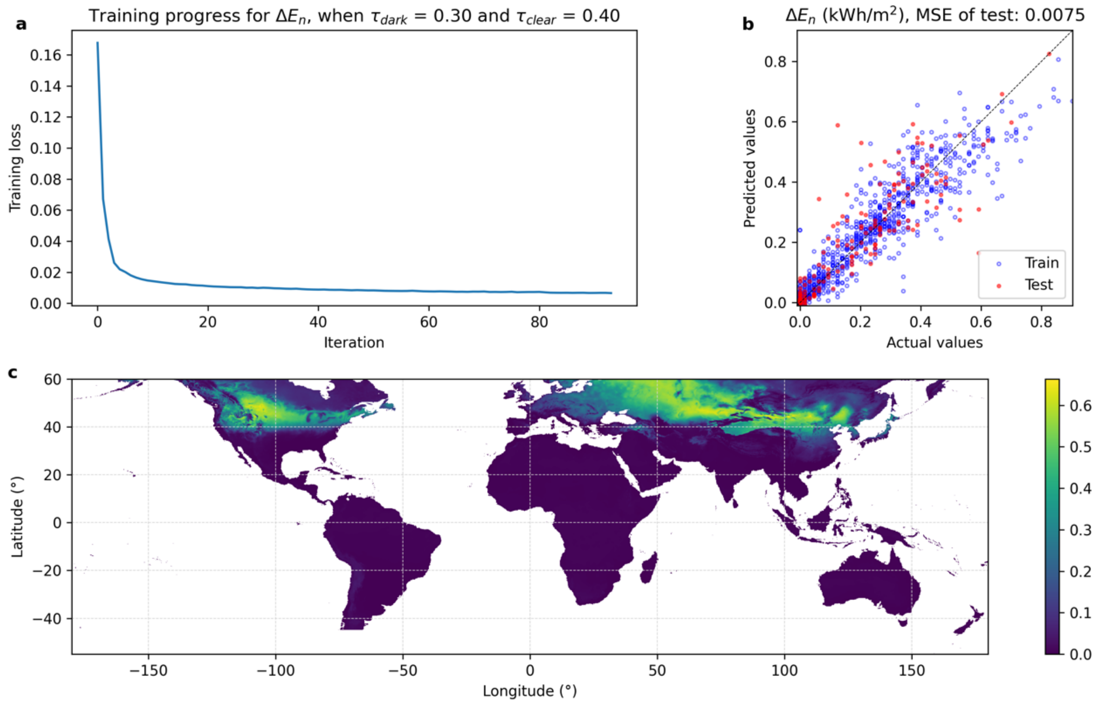

Supplementary Figure 103| ANN training progress, training and testing results, and world heatmap of ΔE_n when $\tau_{clear} = 0.4$, and $\tau_{dark} = 0.3$. Note that the world map is not a geographically accurate representation with a proper projection, but rather a visual plot of array data.

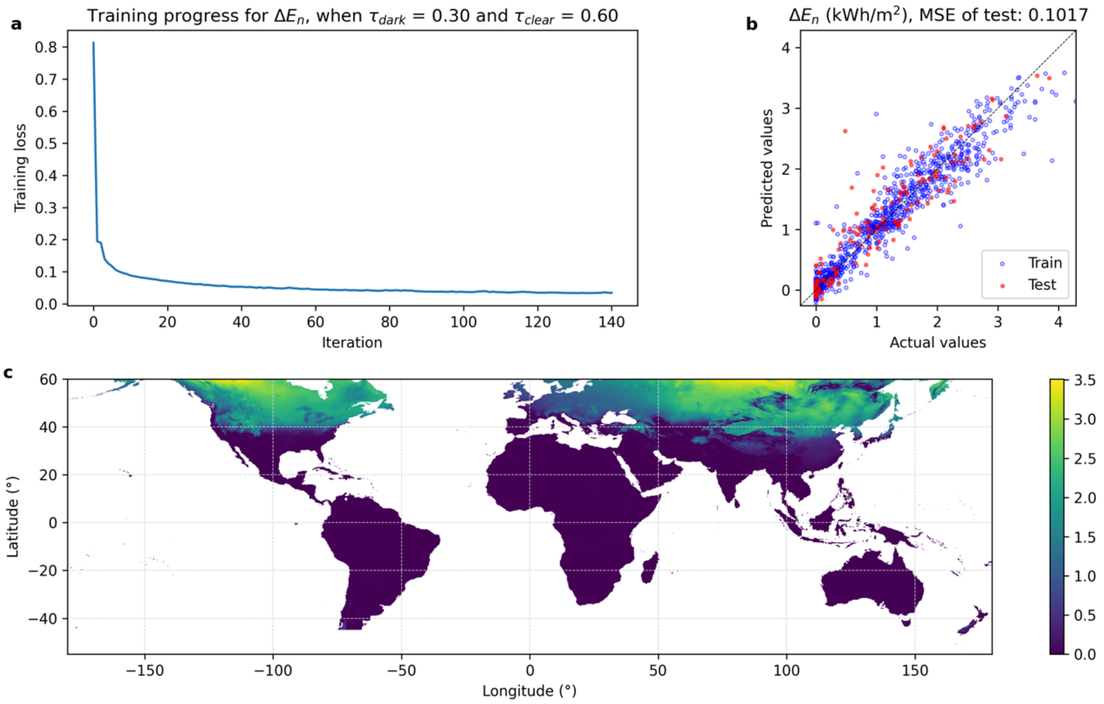

Supplementary Figure 104| ANN training progress, training and testing results, and world heatmap of ΔE_n when $\tau_{clear} = 0.6$, and $\tau_{dark} = 0.3$. Note that the world map is not a geographically accurate representation with a proper projection, but rather a visual plot of array data.

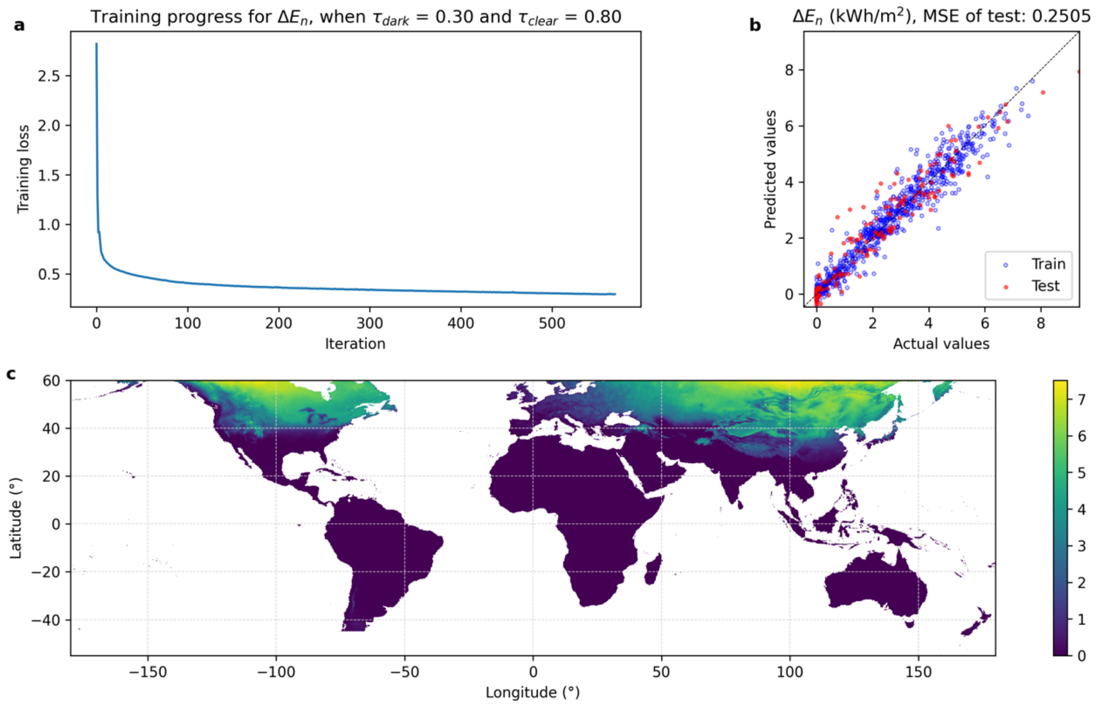

Supplementary Figure 105| ANN training progress, training and testing results, and world heatmap of ΔE_n when $\tau_{clear} = 0.8$, and $\tau_{dark} = 0.3$. Note that the world map is not a geographically accurate representation with a proper projection, but rather a visual plot of array data.

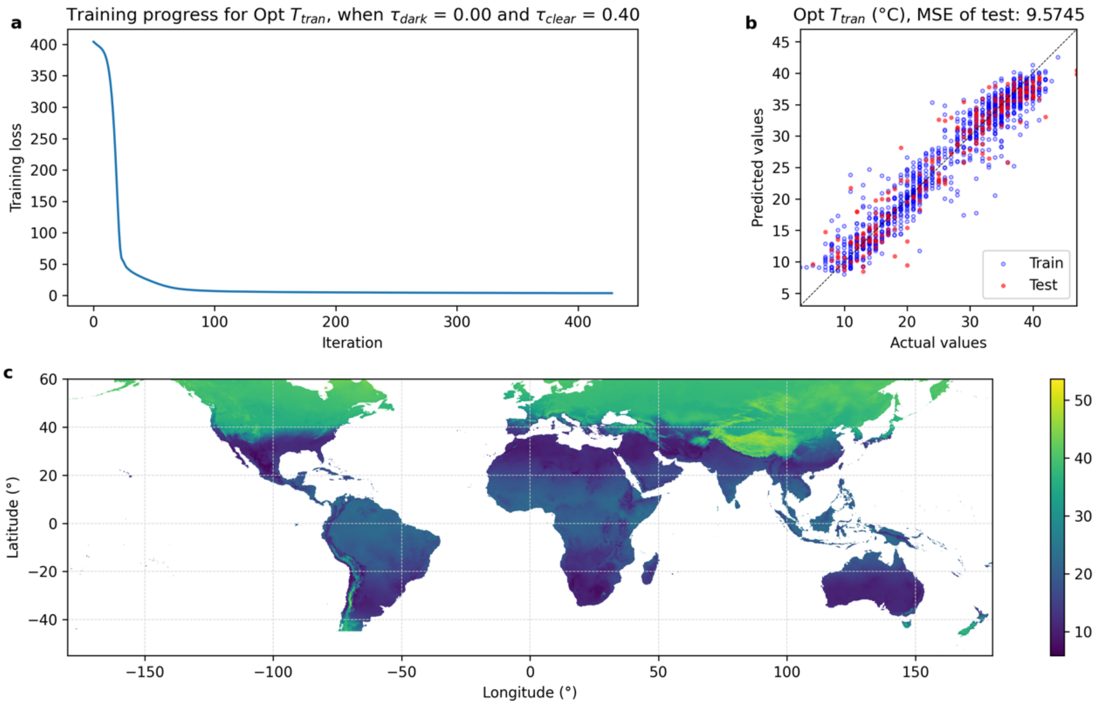

Supplementary Figure 106| ANN training progress, training and testing results, and world heatmap of optimal T_{tran} when $\tau_{clear} = 0.4$, and $\tau_{dark} = 0$. Note that the world map is not a geographically accurate representation with a proper projection, but rather a visual plot of array data.

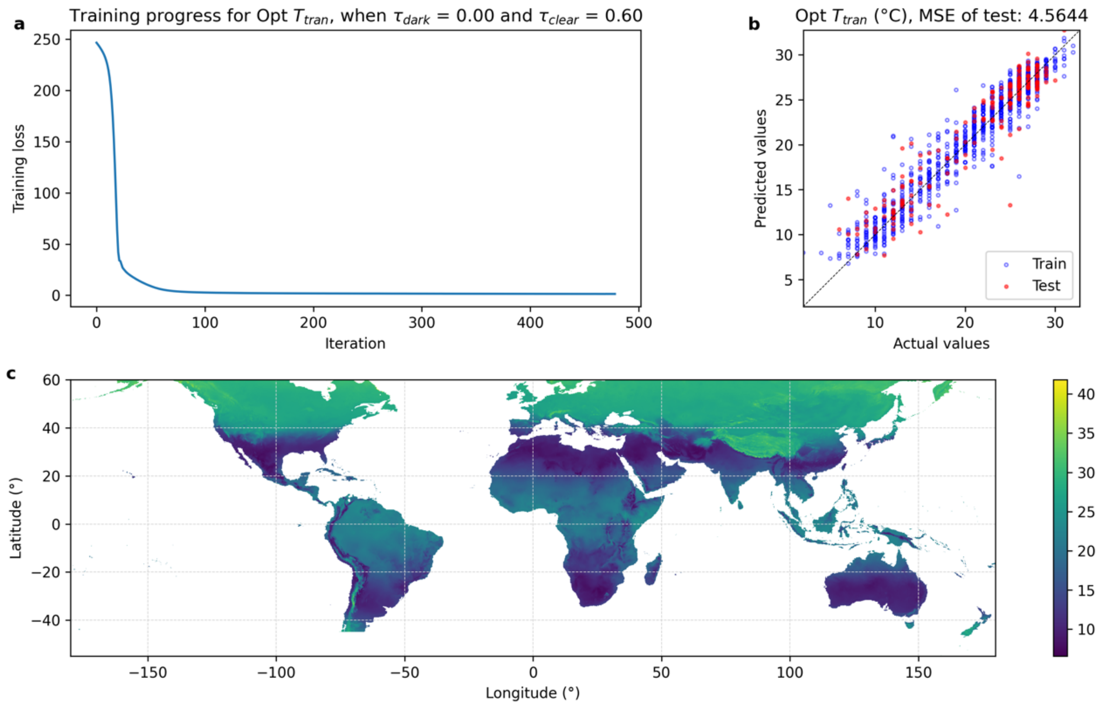

Supplementary Figure 107| ANN training progress, training and testing results, and world heatmap of optimal T_{tran} when $\tau_{clear} = 0.6$, and $\tau_{dark} = 0$. Note that the world map is not a geographically accurate representation with a proper projection, but rather a visual plot of array data.

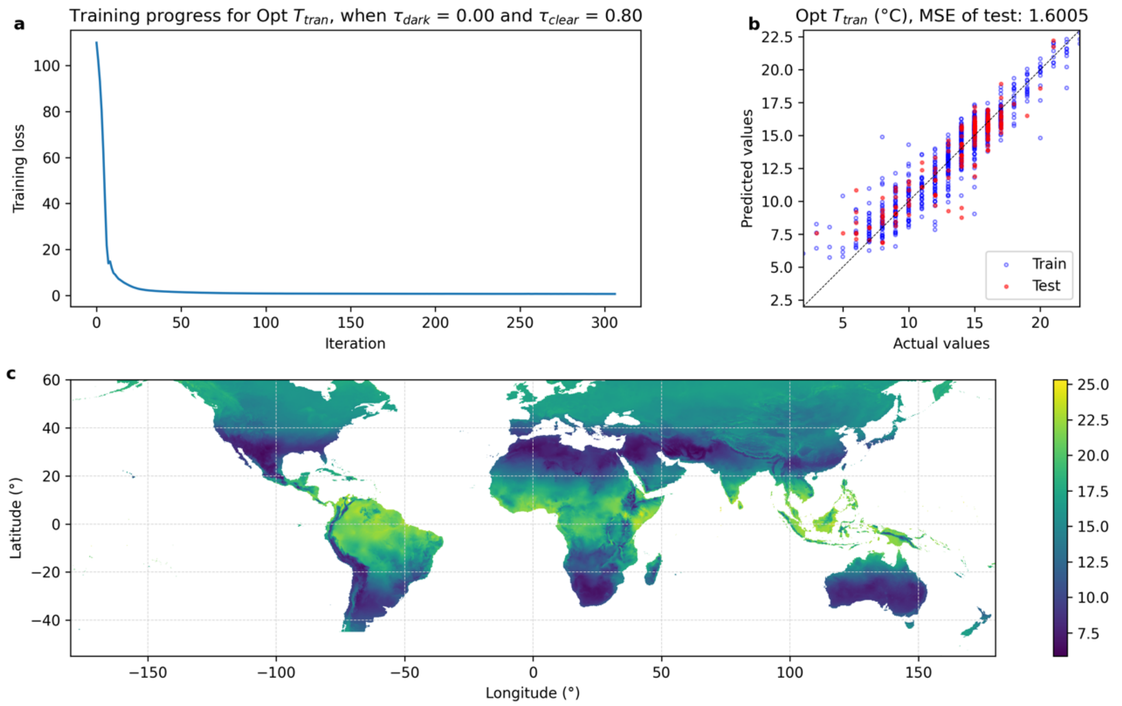

Supplementary Figure 108| ANN training progress, training and testing results, and world heatmap of optimal T_{tran} when $\tau_{clear} = 0.8$, and $\tau_{dark} = 0$. Note that the world map is not a geographically accurate representation with a proper projection, but rather a visual plot of array data.

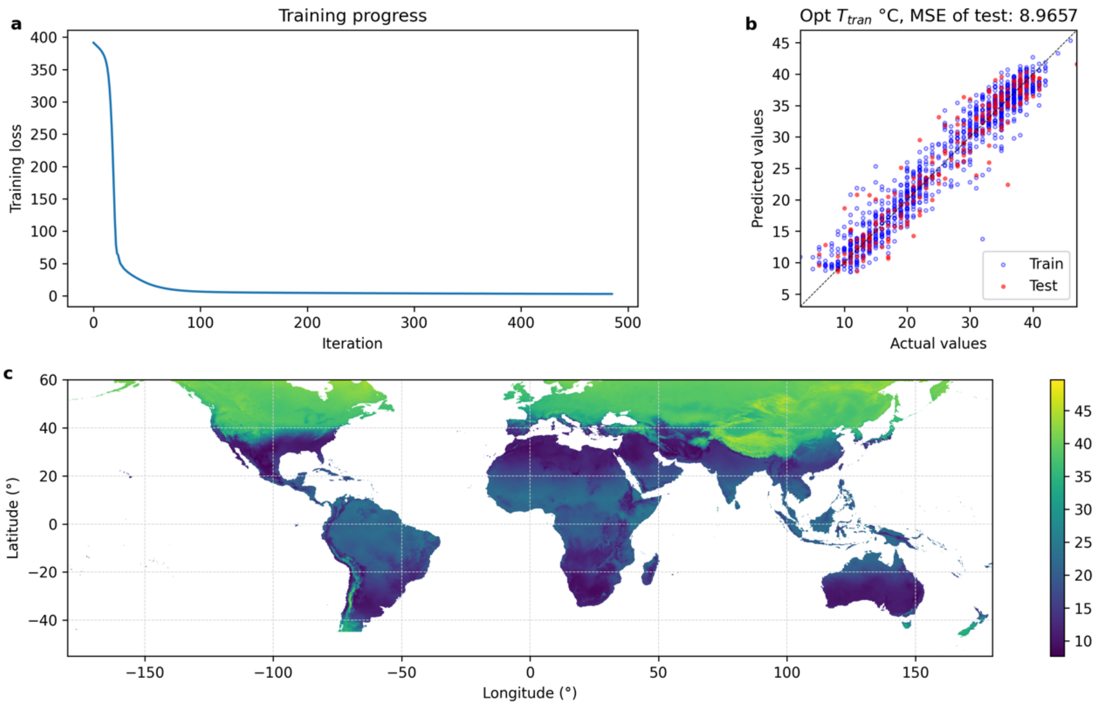

Supplementary Figure 109| ANN training progress, training and testing results, and world heatmap of optimal T_{tran} when $\tau_{clear} = 0.4$, and $\tau_{dark} = 0.05$. Note that the world map is not a geographically accurate representation with a proper projection, but rather a visual plot of array data.

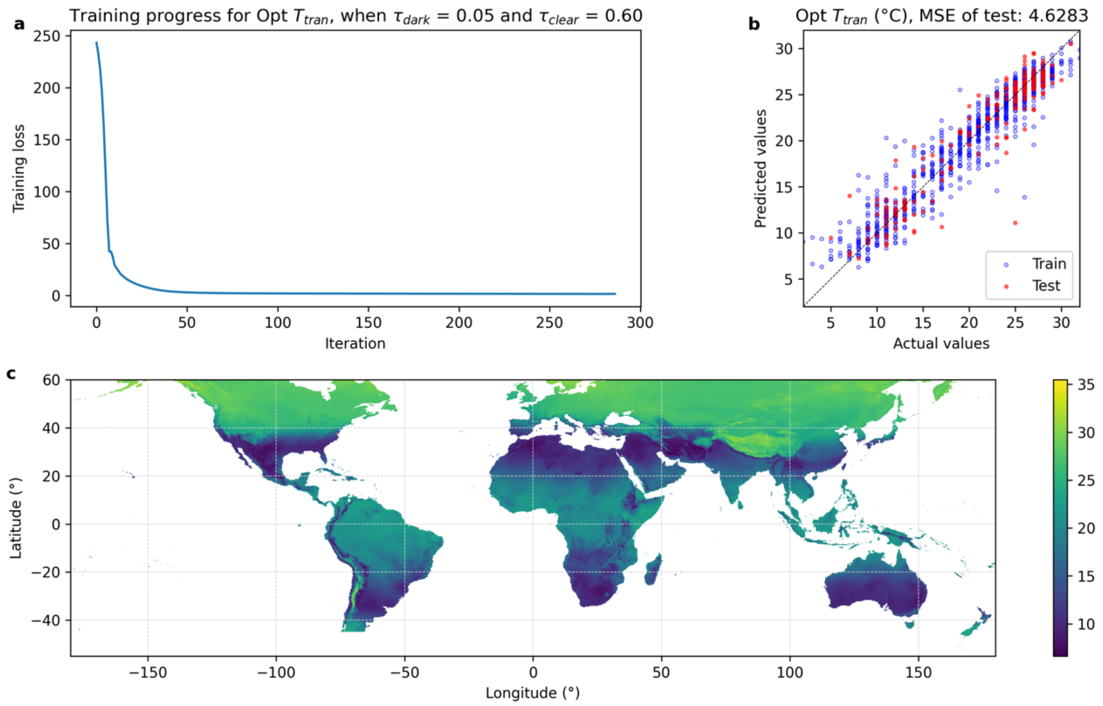

Supplementary Figure 110| ANN training progress, training and testing results, and world heatmap of optimal T_{tran} when $\tau_{clear} = 0.6$, and $\tau_{dark} = 0.05$. Note that the world map is not a geographically accurate representation with a proper projection, but rather a visual plot of array data.

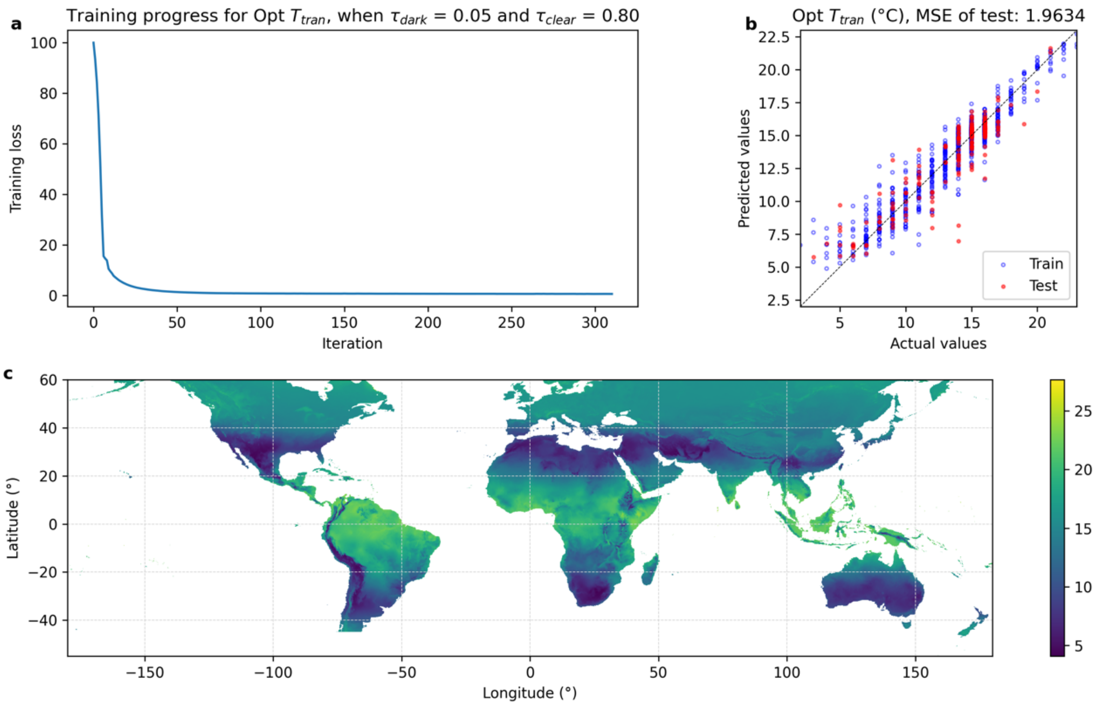

Supplementary Figure 111| ANN training progress, training and testing results, and world heatmap of optimal T_{tran} when $\tau_{clear} = 0.8$, and $\tau_{dark} = 0.05$. Note that the world map is not a geographically accurate representation with a proper projection, but rather a visual plot of array data.

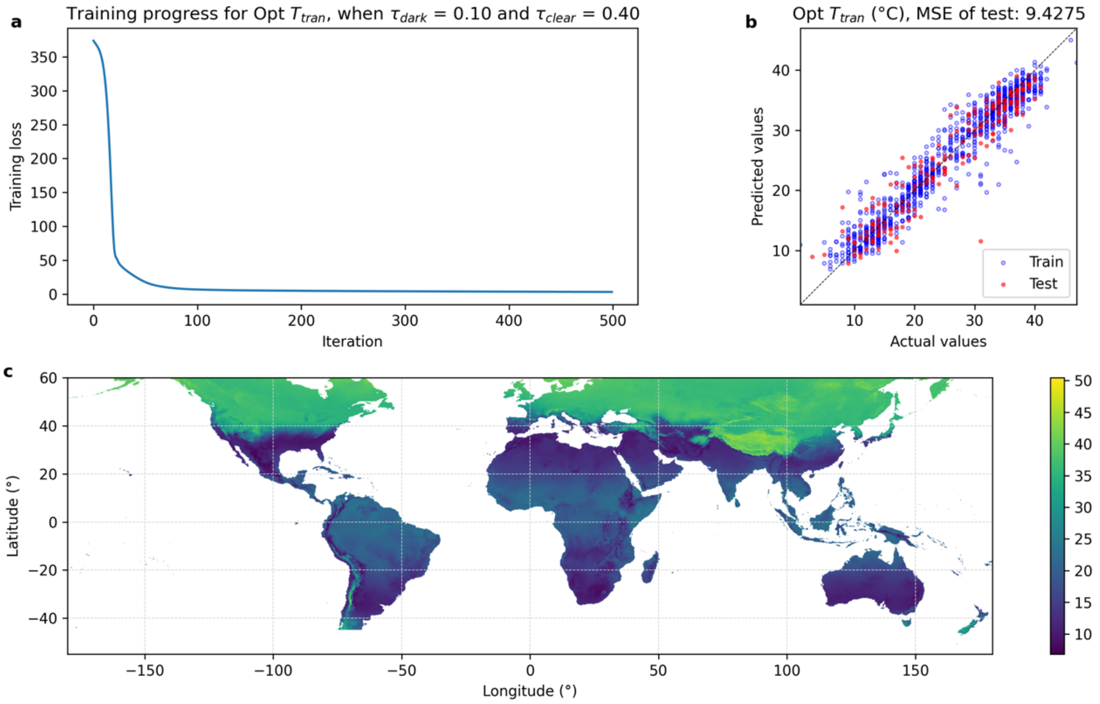

Supplementary Figure 112| ANN training progress, training and testing results, and world heatmap of optimal T_{tran} when $\tau_{clear} = 0.4$, and $\tau_{dark} = 0.1$. Note that the world map is not a geographically accurate representation with a proper projection, but rather a visual plot of array data.

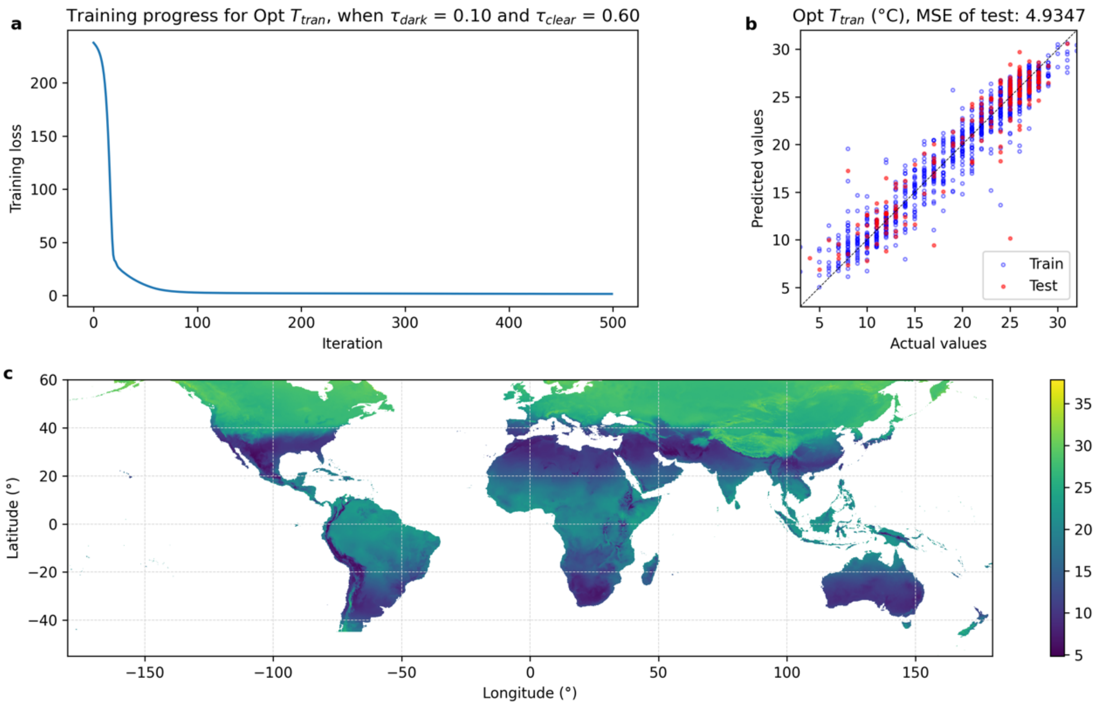

Supplementary Figure 113| ANN training progress, training and testing results, and world heatmap of optimal T_{tran} when $\tau_{clear} = 0.6$, and $\tau_{dark} = 0.1$. Note that the world map is not a geographically accurate representation with a proper projection, but rather a visual plot of array data.

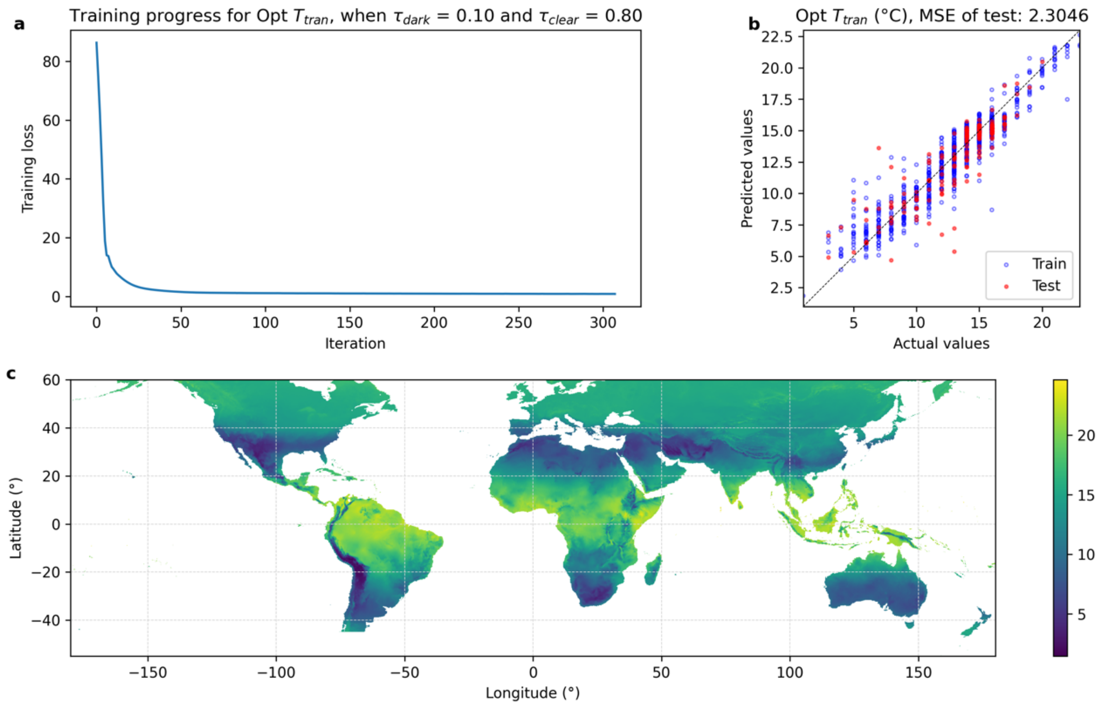

Supplementary Figure 114| ANN training progress, training and testing results, and world heatmap of optimal T_{tran} when $\tau_{clear} = 0.8$, and $\tau_{dark} = 0.1$. Note that the world map is not a geographically accurate representation with a proper projection, but rather a visual plot of array data.

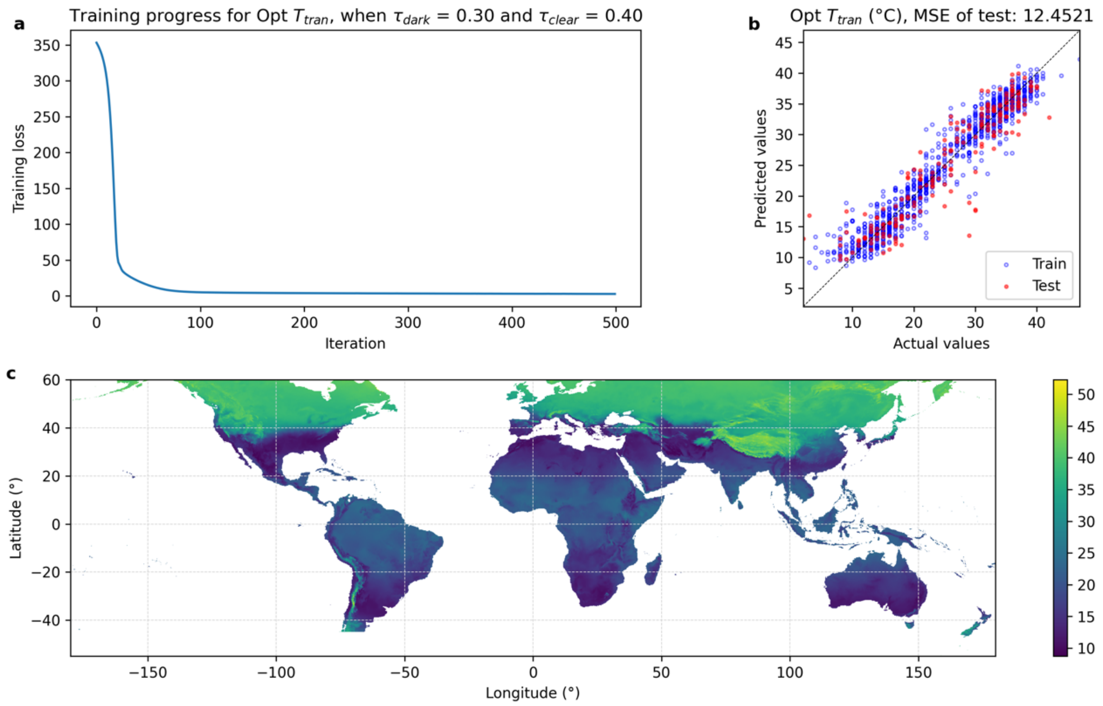

Supplementary Figure 115| ANN training progress, training and testing results, and world heatmap of optimal T_{tran} when $\tau_{clear} = 0.4$, and $\tau_{dark} = 0.3$. Note that the world map is not a geographically accurate representation with a proper projection, but rather a visual plot of array data.

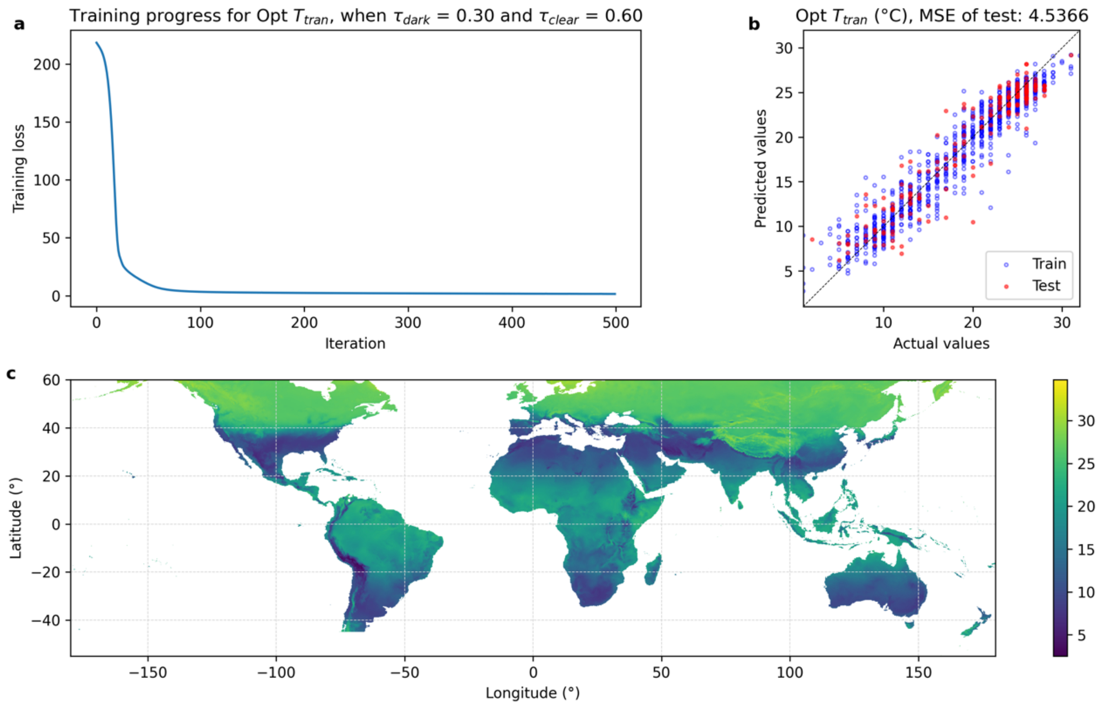

Supplementary Figure 116| ANN training progress, training and testing results, and world heatmap of optimal T_{tran} when $\tau_{clear} = 0.6$, and $\tau_{dark} = 0.3$. Note that the world map is not a geographically accurate representation with a proper projection, but rather a visual plot of array data.

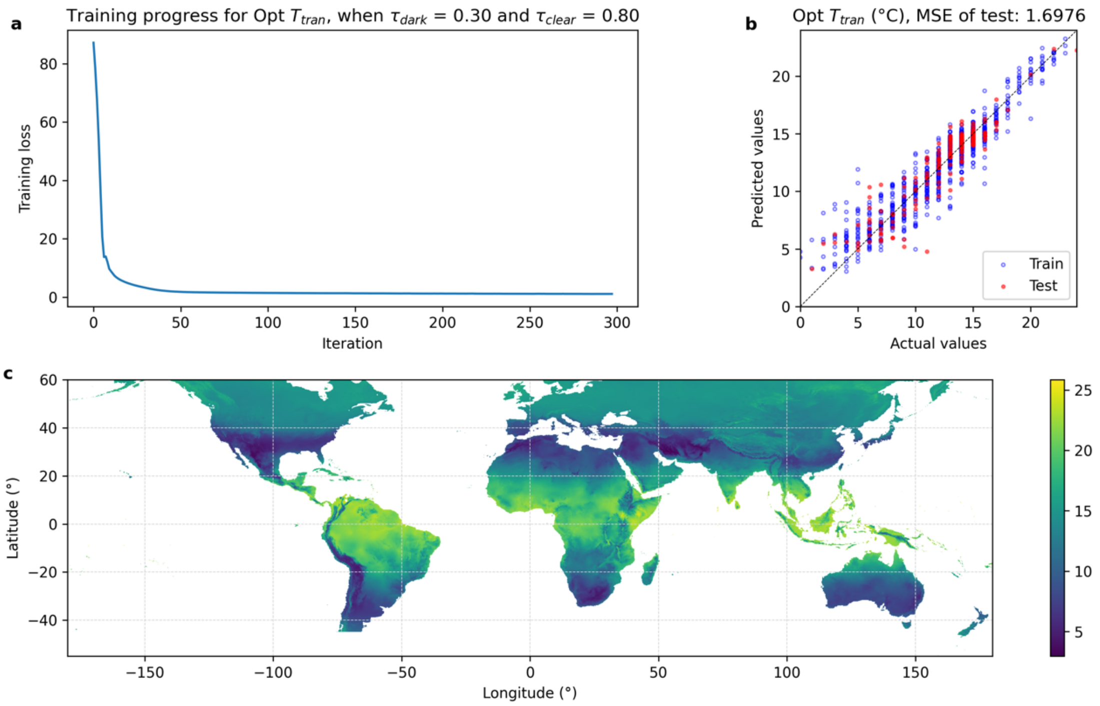

Supplementary Figure 117| ANN training progress, training and testing results, and world heatmap of optimal T_{tran} when $\tau_{clear} = 0.8$, and $\tau_{dark} = 0.3$. Note that the world map is not a geographically accurate representation with a proper projection, but rather a visual plot of array data.

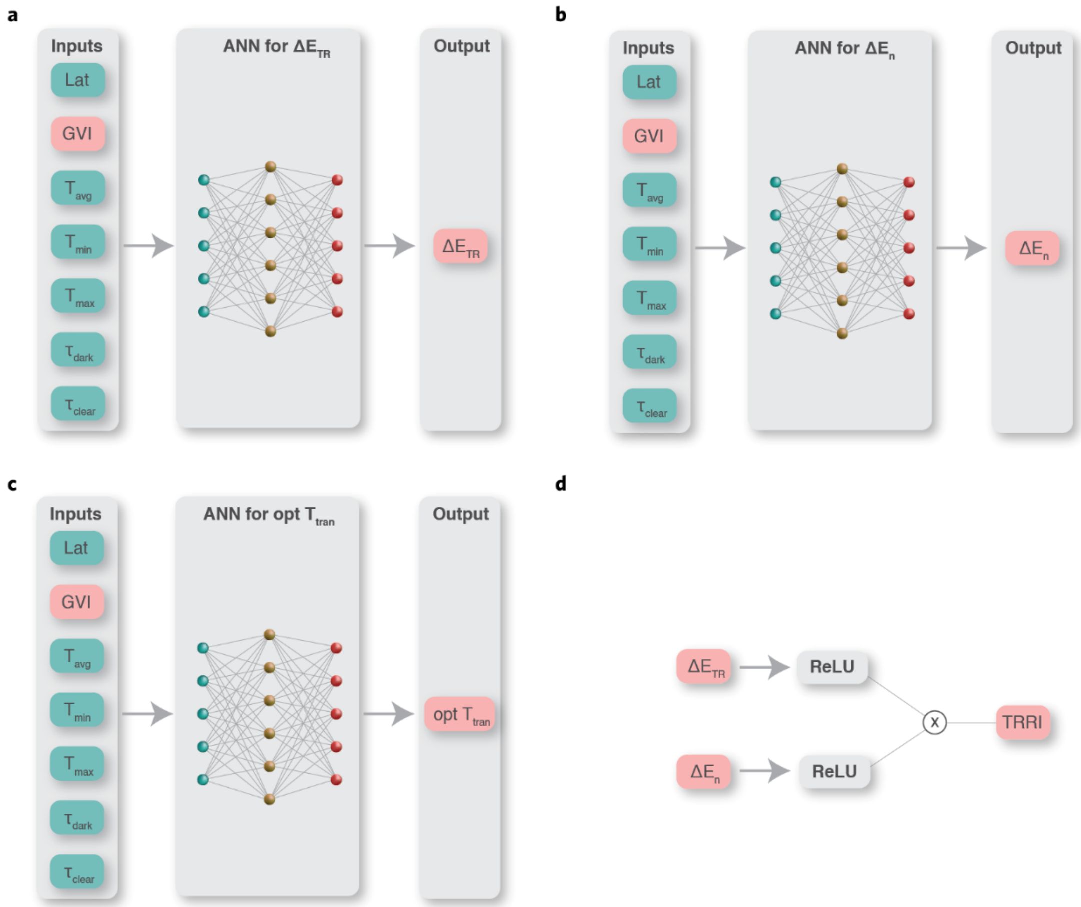

Supplementary Figure 118| Structures of artificial neural networks (ANNs) for ΔE_{TR}, ΔE_n, and optimal T_{tran} , and the calculation of TRRI with certain τ_{clear} and τ_{dark}. Note here the network structures depicted in the figure do not precisely resemble the actual hidden layers.

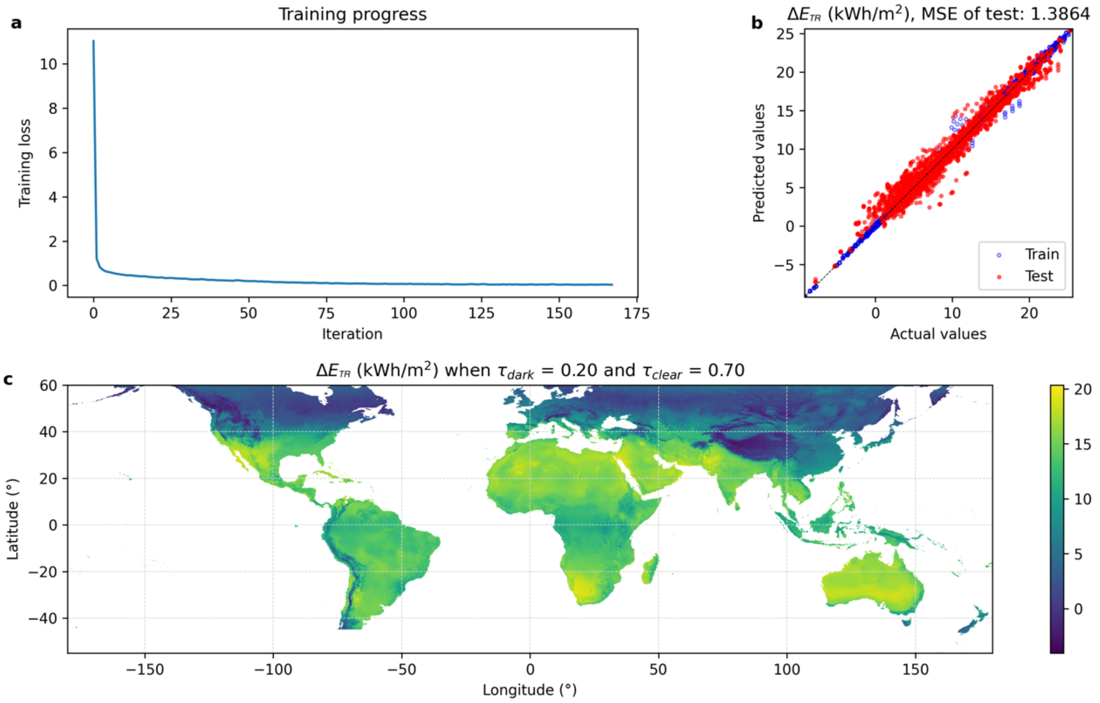

Supplementary Figure 119| ANN training progress, training and testing results, and world heatmap of ΔE_{TR} as inputting $\tau_{clear} = 0.7$, and $\tau_{dark} = 0.2$. The optimal hidden layer size is (181, 158, 187, 172, 141). Note that the world map is not a geographically accurate representation with a proper projection, but rather a visual plot of array data.

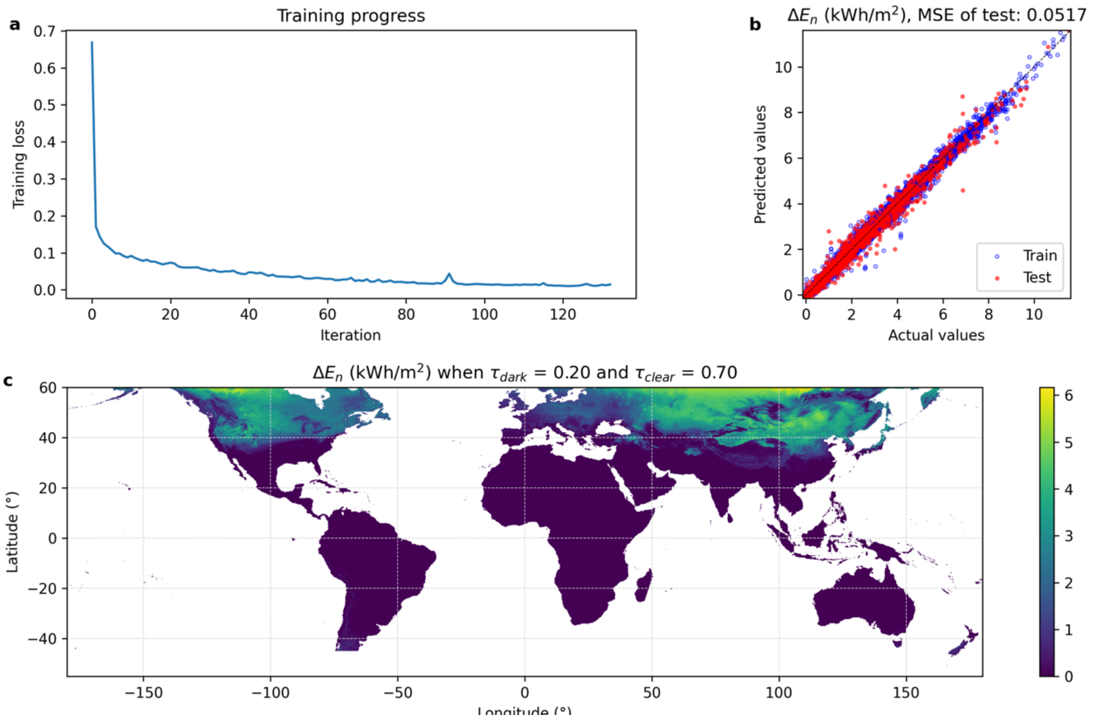

Supplementary Figure 120| ANN training progress, training and testing results, and world heatmap of ΔE_n as inputting $\tau_{clear} = 0.7$, and $\tau_{dark} = 0.2$. The optimal hidden layer size is (175, 179, 177, 140, 81). Note that the world map is not a geographically accurate representation with a proper projection, but rather a visual plot of array data.

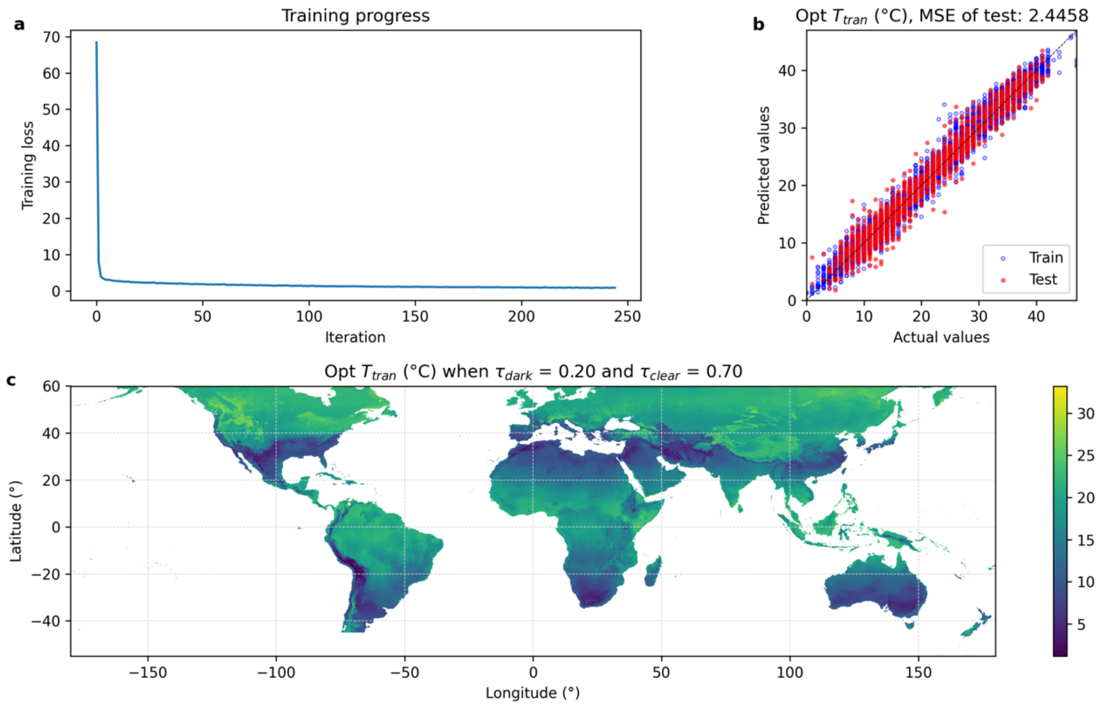

Supplementary Figure 121| ANN training progress, training and testing results, and world heatmap of optimal T_{tran} as inputting $\tau_{clear} = 0.7$, and $\tau_{dark} = 0.2$. The optimal hidden layer size is (182, 114, 124, 59, 107, 115, 171). Note that the world map is not a geographically accurate representation with a proper projection, but rather a visual plot of array data.

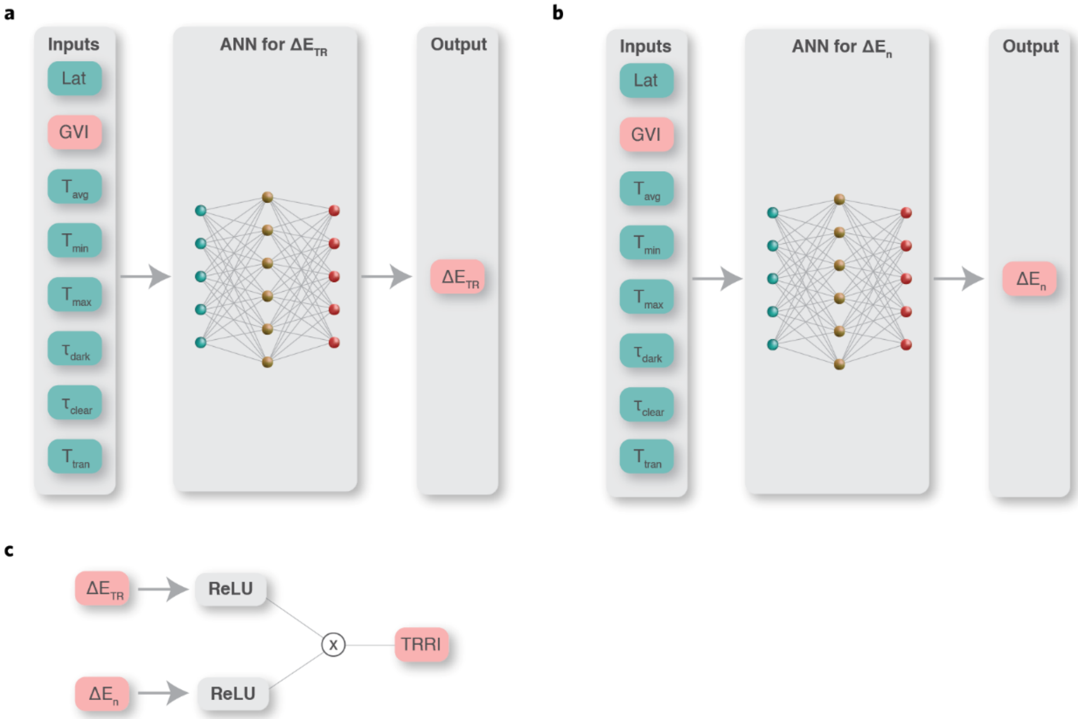

Supplementary Figure 122 | Structures of artificial neural networks (ANNs) for ΔE_{TR}, and ΔE_n, and the calculation of TRRI with certain τ_{clear}, τ_{dark}, and T_{tran}. Note here the network structures depicted in the figure do not precisely resemble the actual hidden layers.

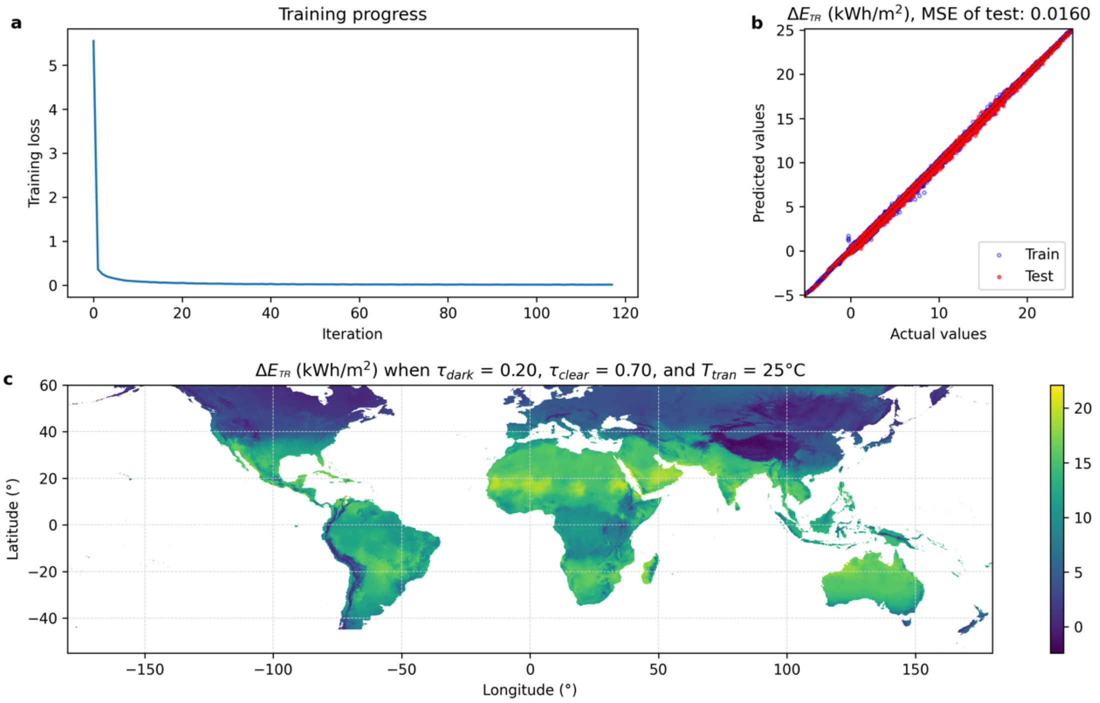

Supplementary Figure 123| ANN training progress, training and testing results, and world heatmap of ΔE_{TR} as inputting $\tau_{clear} = 0.7$, $\tau_{dark} = 0.2$, and $T_{tran} = 25$°C. The optimal hidden layer size is (60, 173, 72, 141, 51). Note that the world map is not a geographically accurate representation with a proper projection, but rather a visual plot of array data.

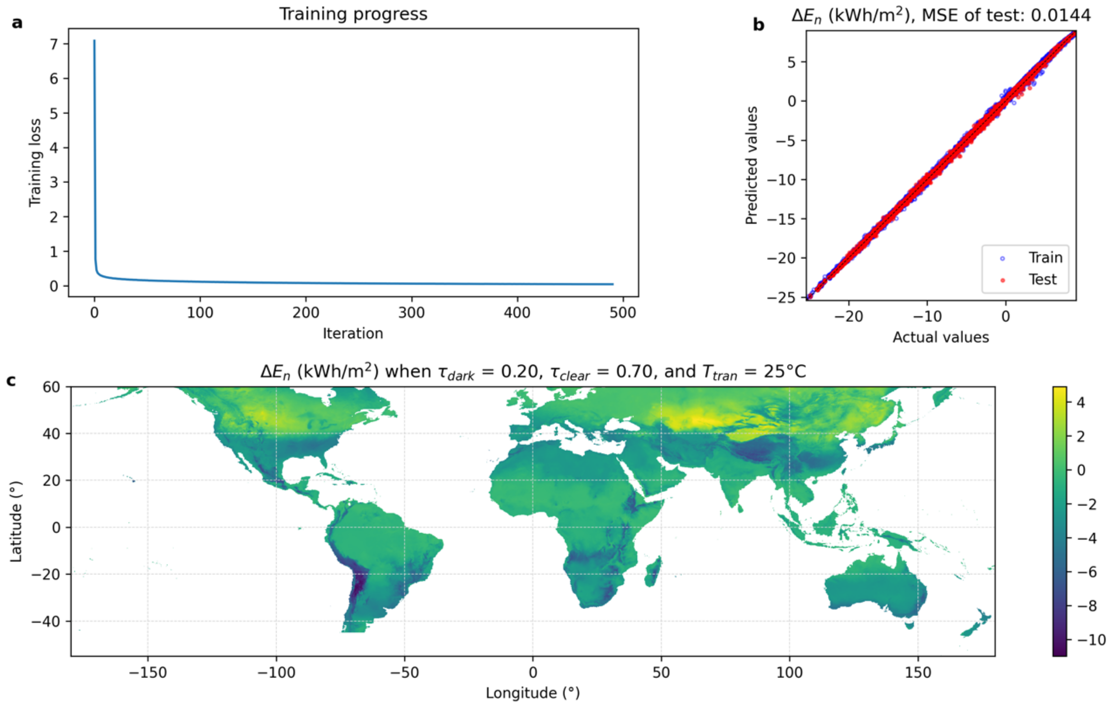

Supplementary Figure 124| ANN training progress, training and testing results, and world heatmap of ΔE_{n} as inputting $\tau_{\mathrm{clear}} = 0.7$, $\tau_{dark} = 0.2$, and $T_{tran} = 25$°C. The optimal hidden layer size is (146, 113, 140, 138, 199, 79, 171, 50). Note that the world map is not a geographically accurate representation with a proper projection, but rather a visual plot of array data.

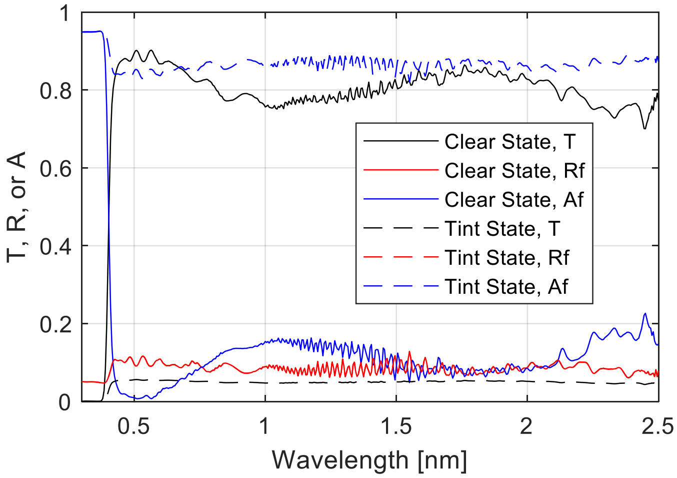

Supplementary Figure 125 | Modified spectral transmittance (T), reflectance (R), and absorptance (A) of thermochromic material based on measured results. The modified example has a dark-state transmittance of 0.05, and a clear-state transmittance of 0.8.

Supplementary Table 1|Summary of key parameters of TC and EC materials and devices

Material category	τ_{dark}	τ_{clear}	$\Delta\tau_{sol}$	T_{tran} (°C)	Year of publication	Reference number
VO2			0.126	34	2021	7
VO2	0.455	0.525	0.07	67.8	2021	8
VO2	0.464	0.559	0.095	63.9	2021	8
VO2	0.479	0.582	0.103	67.9	2021	8
VO2	0.482	0.57	0.088	56	2021	8
VO2	0.466	0.539	0.073	64.5	2021	8
VO2	0.449	0.513	0.064	37.5	2021	8
VO2			0.139	62.95	2021	9
VO2	0.352	0.52	0.168	37	2019	10
VO2			0.187	57	2021	11
VO2			0.114	50	2021	12
VO2				38.9	2021	13
VO2			0.107	39	2022	14
VO2	0.422	0.53	0.108	36.9	2021	15
VO2	0.496	0.602	0.106	46.9	2021	16
VO2	0.5129	0.6646	0.1517	60	2021	17
VO2	0.3	0.396	0.096	22	2022	18
VO2			0.183	42	2022	19
VO2	0.384	0.453	0.069	24.8	2021	20
VO2	0.356	0.445	0.089	49.7	2021	20
VO2			0.141	62.4	2019	21
VO2	0.497	0.681	0.184	64	2022	22
VO2	0.143	0.329	0.186	68	2011	23
VO2			0.088	68	2018	24
VO2			0.021	42.5	2018	25
VO2	0.269	0.477	0.208	68	2019	26
VO2			0.164	68	2019	27
VO2			0.142	66	2021	28
VO2			0.145	68	2019	29
VO2			0.184	58.5	2019	30
VO2			0.189	68	2018	31
VO2	0.431	0.543	0.112	68	2019	32
VO2	0.324	0.431	0.107	22	2021	33
VO2	0.372	0.529	0.157	34.2	2014	34
VO2	0.1355	0.2883	0.1529	68	2016	35
VO2	0.363	0.524	0.161	68	2017	36
Hydrogel			0.463	32.5	2019	37

Hydrogel	0.316	0.81	0.496	32	2014	[38]
Hydrogel	0.019	0.817	0.798	30	2021	[39]
Hydrogel	0.0028	0.9543	0.9515	27.2	2021	[40]
Hydrogel	0.028	0.841	0.813	31	2019	[41]
Hydrogel			0.343	32	2019	[42]
Hydrogel	0.0162	0.7882	0.772	30	2021	[43]
Hydrogel	0.508	0.765	0.257	38	2016	[44]
Hydrogel	0.381	0.782	0.401	35	2016	[44]
Hydrogel	0.233	0.669	0.436	30	2016	[44]
Hydrogel	0.442	0.789	0.347	32	2015	[45]
Hydrogel			0.475	26.5	2021	[46]
Hydrogel	0.016	0.697	0.681	32.5	2020	[47]
Hydrogel	0.175	0.872	0.697	42.7	2021	[48]
Hydrogel	0.156	0.873	0.717	41.7	2021	[48]
Hydrogel	0.15	0.878	0.728	40.5	2021	[48]
Hydrogel	0.115	0.863	0.748	34.1	2021	[48]
Hydrogel	0.096	0.858	0.762	30.7	2021	[48]
Hydrogel	0.404	0.719	0.315	32	2019	[49]
Hydrogel			0.8884	40.7	2023	[50]
Hydrogel	0.2096	0.7744	0.5648	30	2022	[51]
Hydrogel			0.608	32	2023	[52]
Hydrogel	0.004	0.806	0.802	30	2021	[53]
Hydrogel	0.0052	0.8762	0.871	35	2021	[54]
Hydrogel	0.4642	0.7489	0.2847	25	2021	[55]
Hydrogel	0.0405	0.6329	0.5924	32.9	2020	[56]
Hydrogel	0.001	0.8162	0.8152	32	2023	[57]
Hydrogel			0.572	34	2023	[58]
Hydrogel			0.655	30	2022	[59]
Hydrogel			0.737	23	2022	[60]
Hydrogel			0.737	42	2022	[60]
Hydrogel			0.686	32	2023	[61]
Hydrogel	0	0.5762	0.5762	30	2021	[62]
Hydrogel	0.0015	0.6867	0.6852	30	2021	[62]
Hydrogel	0.0123	0.7433	0.731	30	2021	[62]
Hydrogel	0.0162	0.7882	0.772	30	2021	[62]
Hydrogel	0.2008	0.8119	0.6111	30	2021	[62]
Hydrogel	0.3304	0.8185	0.4881	30	2021	[62]
Hydrogel	0.278	0.871	0.593	30	2023	[63]
Hydrogel	0.216	0.852	0.636	30	2023	[63]

Hydrogel	0.189	0.885	0.696	30	2023	[63]
Hydrogel	0.263	0.863	0.6	30	2023	[63]
Hydrogel	0.193	0.87	0.677	30	2023	[63]
Hydrogel	0.161	0.879	0.718	30	2023	[63]
Hydrogel	0.423	0.854	0.431	30	2023	[63]
Hydrogel	0.257	0.802	0.545	30	2023	[63]
Hydrogel	0	0.95	0.95	32.5	2021	[64]
Hydrogel	0.0774	0.4554	0.378	24	2021	[65]
Hydrogel	0.0774	0.4554	0.378	37	2021	[65]
Hydrogel	0.064	0.4206	0.3566	24	2021	[65]
Hydrogel	0.064	0.4206	0.3566	37	2021	[65]
Hydrogel			0.629	33	2022	[66]
Hydrogel			0.5991	19	2023	[67]
Hydrogel			0.5991	41	2023	[67]
Hydrogel			0.6089	20	2023	[68]
Hydrogel			0.6089	48	2023	[68]
Hydrogel	0.1493	0.8063	0.6571	29	2023	[69]
Hydrogel	0	0.872	0.872	36.3	2023	[70]
Hydrogel			0.858	26.7	2023	[71]
Hydrogel	0.021	0.671	0.65	30	2018	[72]
Hydrogel	0.041	0.69	0.649	30	2018	[72]
Hydrogel	0.075	0.623	0.548	30	2018	[72]
Hydrogel	0.097	0.724	0.627	30	2018	[72]
Hydrogel	0.048	0.693	0.645	29	2023	[73]
Hydrogel	0.008	0.826	0.818	32	2023	[74]
Liquid Crystals	0.696	0.891	0.195	42	2021	[75]
Liquid Crystals				42	2017	[76]
Liquid Crystals	0.171	0.64	0.469	29	2018	[77]
RME	0.001	0.7	0.699	NaN	2023	[78]
RME	0.001	0.7	0.699	NaN	2022	[79]
RME	0.06	0.75	0.69	NaN	2019	[80]
RME	0.05	0.6	0.55	NaN	2018	[81]
RME	0.001	0.6	0.599	NaN	2021	[82]
RME	0.01	0.7	0.69	NaN	2022	[83]
WO3 nanoarrays	0.08	0.9	0.82	NaN	2023	[84]
PDLC	0.42	0.62	0.2	NaN	2020	[85]
PDLC	0.23	0.41	0.18	NaN	2018	[86]

Note: blank cells indicate unavailable data in the reference paper.

Supplementary Table 2|Variable range and resolution for three simulation batches

Parameter name	Baseline windows	Batch #1 for U.S.	Batch #2 for U.S.	Batch #3 for world
τ_{dark}	-	0, 0.05, 0.1, 0.3	0, 0.05, 0.1, 0.3	0, 0.05, 0.1, 0.3
τ_{clear}	-	0.4, 0.6, 0.8	0.4, 0.6, 0.8	0.4, 0.6, 0.8
T_{tran} (°C)	-	-100, 0, 10, 20, 30, 40, 70, 100	0 to 40 with 1 interval, -100, 100 (-25 to 75 for double clear)	0 to 40 with 1 interval, -100, 100
Breadth (°C)	-	1	1	1
Hysteresis	-	0	0	0
TR-applied surface	-	Surface # 1, n (n = 2, 4, and 6 for single, double and triple glazing)	Surface # 1	Surface # 1
Locations	16 U.S. representative cities[3]	16 U.S. representative cities	16 U.S. representative cities	2226 cities in the world
Window orientations	S, E, W, N	S, E, W, N	S, E, W	S (or N)
Glazing configuration	Single clear, double clear, double low-e, triple low-e #1, triple low-e #2	Single clear, double clear, double low-e, triple low-e #1, triple low-e #2	Single clear, double clear, double low-e, triple low-e #1, triple low-e #2	Double clear
Lighting control (LC)	No, Yes	No, Yes	No, Yes	No
Combinations	640	122880	1572480	1148616

Supplementary Table 3|U.S. Climate zone classification and representative cities[3]

Climate zone**	Representative city	Köppen-Geiger classification	Heating Degree Days (HDD)*	Cooling Degree Days (CDD)*
1A	Miami, Florida	Am	72	2477
2A	Houston, Texas	Cfa	786	1667
2B	Phoenix, Arizona	BWh	523	2532
3A	Atlanta, Georgia	Cfa	1497	1023
3B-CA	Los Angeles, California	BSh and Csa/Csb	713	343
3B-other	Las Vegas, Nevada	BWh	1169	1860
3C	San Francisco, California	Csb	1504	79
4A	Baltimore, Maryland	Cfa	2537	682
4B	Albuquerque, New Mexico	BSk	2261	749
4C	Seattle, Washington	Csb	2627	98
5A	Chicago, Illinois	Dfa	3506	468
5B	Denver, Colorado	BSk	3301	432
6A	Minneapolis, Minnesota	Dfa	4203	417
6B	Helena, Montana	BSk	4266	208
7	Duluth, Minnesota	Dfb	5236	116
8	Fairbanks, Alaska	Dfc	7516	39

*****Annual (standard) heating/cooling degree-days (18.3°C (or 65°F) baseline)

**See the map in Supplementary Reference 3 to understand the climate zone classification in the U.S.[3] The numbers 1 – 8 indicate very hot, hot, warm, mixed, cool, cold, very cold, and subarctic, respectively.

Supplementary Note 1

In most material research, it is highly challenging to fabricate full-sized TR windows and conduct experiments in actual buildings, especially for labs that can only produce small-size samples. Instead, most studies experimented their samples in small-scale chambers (see Table 2 in *Cell Reports Physical Science* 4.5 (2023)) or evaluating the energy performance of materials in buildings through simulations. Only a few studies have employed full-sized TR windows in real buildings for field experiments (summarized in Supplementary Table 4).

A publication in 2013 introduced an experimental work on large-area polymer thermochromic (TC) laminated windows in a full-scale testbed office in our lab [*Solar Energy Materials and Solar Cells* 116 (2013): 14-26]. Several measured parameters, such as transmitted solar radiation, incident vertical irradiance, and outdoor dry-bulb temperature, were compared against the predicted values. EnergyPlus was used to calculate the annual energy performance for Chicago and Houston. However, the thermochromic module in EnergyPlus was not validated in this study. Both empirical and simulation data were utilized to demonstrate that the ideal critical switching temperature for TC windows should be determined by the zone heat balance rather than the ambient air temperature. Another demonstration work was also reported in 2013 by our lab regarding the energy performance of electrochromic and thermochromic windows in a federal building in Denver [GSA report (2013)]. The study on thermochromic windows involved several key measurements to evaluate their performance, including glazing temperature, vertical irradiance and illuminance. Environmental factors like incident solar radiation, outdoor air temperature, wind, and indoor air temperature were also monitored to understand their influence on the window's switching behavior. EnergyPlus simulation was conducted for annual energy calculation and was not validated by measured data. In 2013 and 2015, two publications reported the demonstration and simulation of VO_2 single glazing [*Solar energy materials and solar cells* 117 (2013): 168-173] and double glazing [*Solar Energy* 120 (2015): 55-64] in a full-scale room in Hefei, China, respectively by the same research team. The simulated cooling load was validated by the measured data. However, the simulation tool is BuildingEnergy software, which is more user-friendly, but less customizable compared with EnergyPlus. They concluded that the TC windows were suitable for hot climates rather than cold climates. VO_2 double window consumes approximately 11.1% less cooling energy than that with an ordinary double window. Another paper published in 2015 reported testing thermotropic glazing and a triple glazing unit in a full-scale outdoor test cell to evaluate their thermal and optical performance [*Proceedings of building simulation* (2015)]. Measurements were taken over several days under varying solar radiation and temperature conditions, focusing on internal glass surface temperature, transmitted solar radiation, and heat flux. The experimental data were then compared with simulation results from EnergyPlus to assess the accuracy of the built-in thermochromic model. Results showed that the errors of simulated internal glazing surface temperature, transmitted solar radiation, and heat flux stayed in acceptable range, proving the accuracy of the built-in thermochromic model in EnergyPlus. This study will be introduced in detail later in the Credibility Analysis Section (B).

Supplementary Table 4|Summary of experimental study on full-size TC windows in real building environment

Publishing year	TC material	Building	Location	Simulation software	Validation	Reference

2013	Polymer	Full-scale, south-facing, conditioned testbed office	Berkeley, California, USA	EnergyPlus	No	*Solar Energy Materials and Solar Cells* 116 (2013): 14-26
2013	Not mentioned	9,500-ft^2 perimeter zone of a Federal office building	Denver Federal Center, Colorado, USA	EnergyPlus	No	GSA report (2013)
2013	VO_2	2.9 x 1.8 x 1.8 m^3	Hefei, China	BuildingEnergy	Yes	*Solar energy materials and solar cells* 117 (2013): 168-173
2015	VO_2	2.9 x 1.8 x 1.8 m^3	Hefei, China	BuildingEnergy	Yes	*Solar Energy* 120 (2015): 55-64
2015	Technology based on a coreshell particle suspension	1.6 x 3.6 x 2.5 m^3 TWINS<br>outdoor test cell	Torino, Italy	EnergyPlus	Yes	*Proceedings of building simulation* (2015)

It will be extremely challenging to directly validate the results of this study. Due to the extensive range of TR window parameters and global climate conditions covered in this research, it is only feasible to validate one or a few of these variables with real-world experiments. Moreover, the simulations in this study utilize reference buildings from the U.S. Department of Energy's (DOE) prototype building models, chosen for their representativeness. The window data in this study were obtained from measurements of small-size samples. In practice, constructing buildings identical to the standard model and manufacturing windows identical to the sample on a large scale are both time-consuming and costly. Although direct validation is difficult, the reliability of this work can still be indirectly verified by analyzing the credibility of each component of this work.

Credibility Analysis

(A) EnergyPlus

This research primarily utilized EnergyPlus, a whole building energy simulation program. Its development is funded by the U.S. DOE Building Technologies Office (BTO). EnergyPlus has undergone extensive validation and verification processes [Testing and Validation, EnergyPlus]. It has been tested against empirical data from real buildings and benchmarked against other established simulation tools [IEA SHC Task 34/Annex 43]. It has been widely adopted in the industry and academia, with numerous peer-reviewed studies attesting to its accuracy and robustness [research articles related to EnergyPlus].

A previous study validated EnergyPlus by comparing simulation results against measured data from real-world buildings and controlled test environments [*Building Simulation 2019*. Vol. 16. IBPSA, 2019]. It is an empirical validation project conducted by multiple national laboratories, including Oak Ridge National

Laboratory (ORNL). The validation utilized ORNL's Flexible Research Platform, a small office building with detailed monitoring systems. The project generated extensive empirical data sets, including cooling energy consumption, fan energy consumption, and zone temperatures, under various test conditions. The accuracy of EnergyPlus was quantified using metrics such as Normalized Mean Bias Error (NMBE) and Coefficient of Variation of the Root Mean Square Error (CV(RMSE)),

$$\mathrm{NMBE} = \frac{1}{\bar{M}} \frac{\sum_{i=1}^{n}(M_i - S_i)}{n} \times 100\%$$

$$\mathrm{CV(RMSE)} = \frac{1}{\bar{M}} \sqrt{\frac{\sum_{i=1}^{n}(M_i - S_i)^2}{n}} \times 100\%$$

where *M*, *S*, and *n* represent the measurement, simulation, and the number of data, respectively. Upper bar refers to the average. The hourly NMBE and CV(RMSE) were less than 2.6% and 5.9%, respectively, indicating that simulation and experimental energy consumption are well matched.

(B) Built-in thermochromic module

Furthermore, the thermochromic module has also been verified by comparing with experimental data in a previous study [*Proceedings of building simulation* (2015)]. In this study, simulation results using EnergyPlus and the built-in thermochromic module were compared with the experimental results measured in a full-scale outdoor test facility (1.6 m x 3.6 m x 2.5 m) in Torino, Italy. The experimental data was collected using the side-by-side test cell facility equipped with a triple glazing unit (TGU) and a triple glazing unit with thermotropic (TT) glazing on the external side (TT+TGU). The data for comparison was taken from April 12th to April 15th, 2013, which included medium to high vertical solar radiation and temperature variations. The results of the reference TGU show good agreement between simulation and experimental data in general. Simulated and measured parameters include internal surface temperature of the glazing ($T_{\mathrm{glass,in}}$), transmitted solar radiation (G_{in}), and heat flux (radiative longwave and convective) on the internal surface of the glazing (HF_{lw}). A two-hour delay was observed in measured temperatures compared to simulation due to EnergyPlus not accounting for the thermal mass of the glazing. This issue could be solved by using equivalent models as demonstrated in our previous work [*Applied Energy* 301 (2021): 117467]. Please note that here our parametric study on TC windows doesn't take the thermal mass of the glazing into account. A peak difference in the heat flux in the afternoon was also observed, likely due to the heat flow meter sensor overheating from direct solar radiation [*Proceedings of building simulation* (2015)].

To quantitively evaluate the simulation performance, the errors between simulation and experimental data were defined as below:

Mean Bias Error (MBE):

$$\mathrm{MBE} = \frac{1}{n}\sum_{i=1}^{n}\left(X_{\mathrm{mod}} - X_{\mathrm{exp}}\right)$$

Root Mean Square Error (RMSE):

$$\mathrm{RMSE} = \sqrt{\frac{1}{n}\sum_{i=1}^{n}\left(X_{\mathrm{mod}} - X_{\mathrm{exp}}\right)^2}$$

Percentage Root Mean Square Error (PRMSE):

$$\mathrm{PRMSE} = \sqrt{\frac{1}{n}\sum_{i=1}^{n}\left(\frac{X_{\mathrm{mod}} - X_{\mathrm{exp}}}{X_{\mathrm{exp}}}\right)^2}$$

where n is the number of measurements.

The results are listed in Supplementary Table 5. The errors remain within an acceptable range, demonstrating that the simulation results for the reference room using EnergyPlus are reliable.

Supplementary Table 5 | Simulation errors compared with measured results in the cell with TGU [Proceedings of building simulation (2015)]

	MBE	RMSE	PRMSE
$T_{\mathrm{glass,in}}$ (°C)	-0.5	1.5	5.1%
G_{in} (W m^{-2})	-0.6	11.5	-
HF_{lw} (W m^{-2})	-6.3	13.7	-

The TT+TGU simulation showed good alignment with measured solar radiation data but exhibited a 2-hour delay and peak temperature differences of 3-4°C during peak solar radiation hours. Additionally, heat flux discrepancies of 10-15 W/m² were noted. These discrepancies, primarily due to increased solar reflectance of the TT glazing in experimental conditions, needed a model calibration to better match the experimental data. The calibration involved increasing the solar and luminous reflectance by a constant factor, which significantly improved model accuracy. After calibration, the MBE for $T_{\mathrm{glass,in}}$ was reduced to -0.01°C, with a RMSE of 1.38°C and a PRMSE of 5%. Similarly, for the HF_{lw}, the MBE was reduced to -0.84 W/m², and the RMSE to 10.31 W/m². These values indicate that the calibrated built-in TC model in EnergyPlus is reliable for predicting the performance of TC glazing technologies.

(C) ANNs

Our study reported multiple different artificial neural networks (ANNs), each trained and tested using different inputs and outputs. Here we select several representative ANNs and their performance, which are displayed in Supplementary Figure 128. The training of each neural network was based on the mean squared error (MSE), defined as

$$\mathrm{MSE} = \frac{1}{n}\sum_{i=1}^{n}(S_i - M_i)^2,$$

where S and M represent the predicted and actual values, respectively. Based on MSE values, we calculated other error metrics, which are listed in Supplementary Table 6. Different ANN structures exhibit various performances as shown in Supplementary Figure 126. Generally, the more effective data can be used to train the ANN, the better its performance becomes. It is noteworthy that due to the inherent randomness in neural network training, the same ANN structure may yield different MSE values across different trainings. In this study, we selected the best-performing results from multiple trainings. Although errors are inevitable, the linear regression between the actual and predicted values of the ANNs is obvious, enabling the ANNs to provide valuable guidance for the design of the TR window. From the error metrics, we can conclude that the errors of the ANNs in this study stay within an acceptable range (Supplementary Table 6).

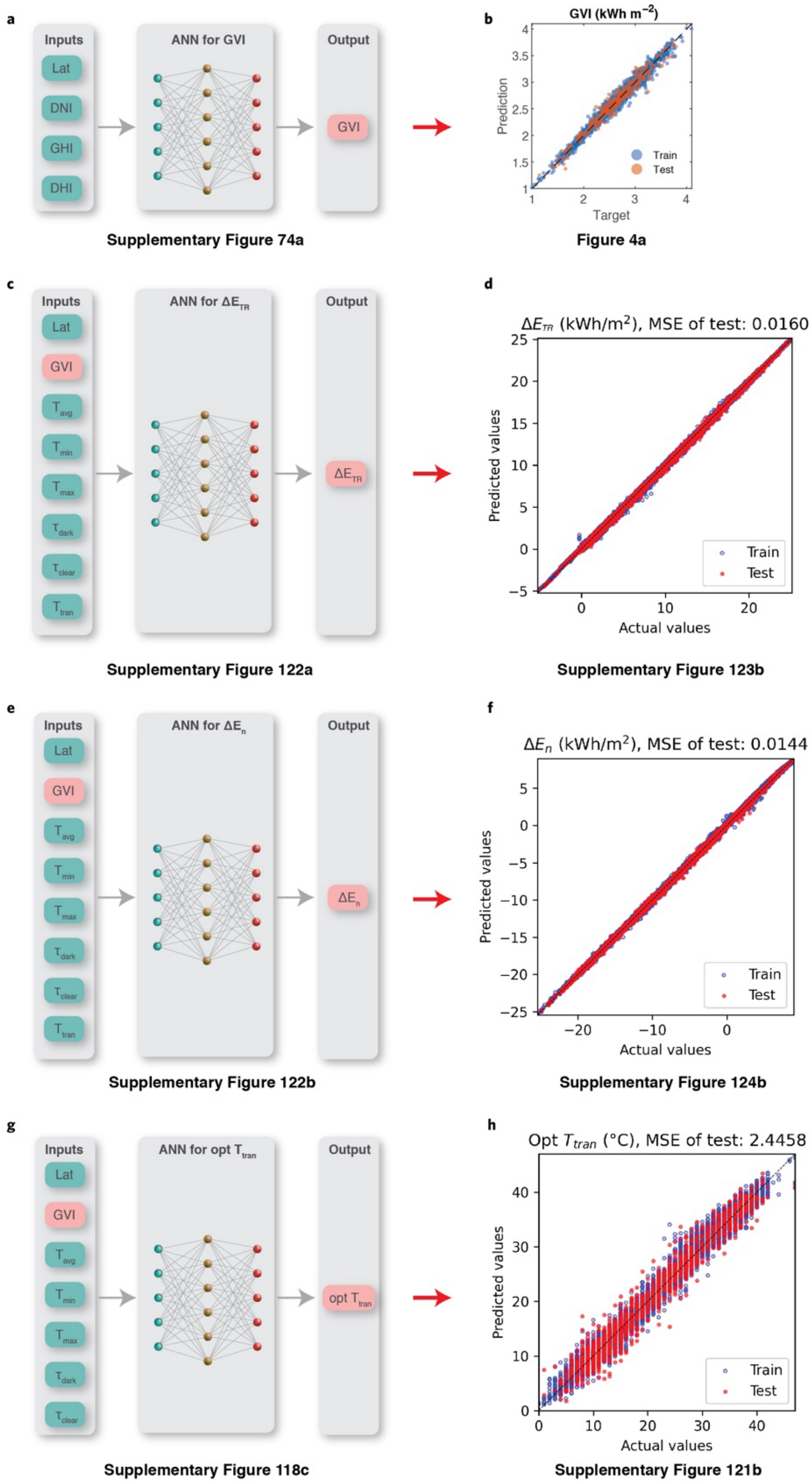

Supplementary Figure 126|Four selected ANNs and the performance of training and testing. Figures are adapted from manuscript and SI.

Supplementary Table 6|Testing performance of four selected ANNs in this study

Output of ANN	MSE	RMSE	CV(RMSE)
GVI	0.0080	0.0894	3.4%
ΔE_{TR} (trained by all conditions)	0.0160	0.1265	1.3%
ΔE_{n} (trained by all conditions)	0.0144	0.1200	3.8%
Optimal T_{tran} (trained by the optimal conditions)	2.4458	1.5639	7.9%

(D) Input data of solar radiation and air temperature

Solar radiation

Solar radiation inputs of this study include direct normal irradiation (DNI), global horizontal irradiation (GHI), and diffuse horizontal irradiation (DHI), which are obtained from the Solargis solar radiation model [Global Solar Atlas]. In a validation report, the accuracy of solar radiation data was calculated through the comparison with ground-data from the reference stations [Global Solar Atlas 2.0 : Validation Report]. In this report, the solar radiation data has been validated at 228 public sites worldwide. The mean bias of GHI and DNI for all sites are 0.3% and 2.2%, respectively. Besides the validation in this report, a list of independent validation studies can also be found in Section 6 of this report. The Solargis solar radiation model has been proved reliable and the solar radiation data used in this study falls within an acceptable error range.

Air temperature

Global air temperature input of this study include minimum, mean, and maximum temperature, which are obtained from WorldClim 2 database [*International journal of climatology* **37**, 4302–4315 (2017)]. The accuracy of the dataset was demonstrated by comparing with station data. All temperature variables exhibited a global correlation coefficient of 0.99 or higher between estimated and observed values, and an average RMSE ranging from 1.1 to 1.4°C. The accuracy is deemed sufficient to predict the trend of building energy performance in a global scale.

In summary, the accuracy of the EnergyPlus software, the built-in thermochromic model, ANNs and weather inputs are analyzed here. The performance of each component in our proposed models are summarized in Supplementary Figure 127. We have successfully demonstrated the accuracy of each component of our model. Strictly speaking, the accuracy of each individual model does not fully validate the accuracy of the overall model. However, we believe this is currently the most convincing evidence we can provide. We have explained previously why it is very difficult to validate the whole model by conducting field experiments. In the future, if conditions allow, we hope to conduct field experiments under specific climate conditions to further test the whole model's accuracy.

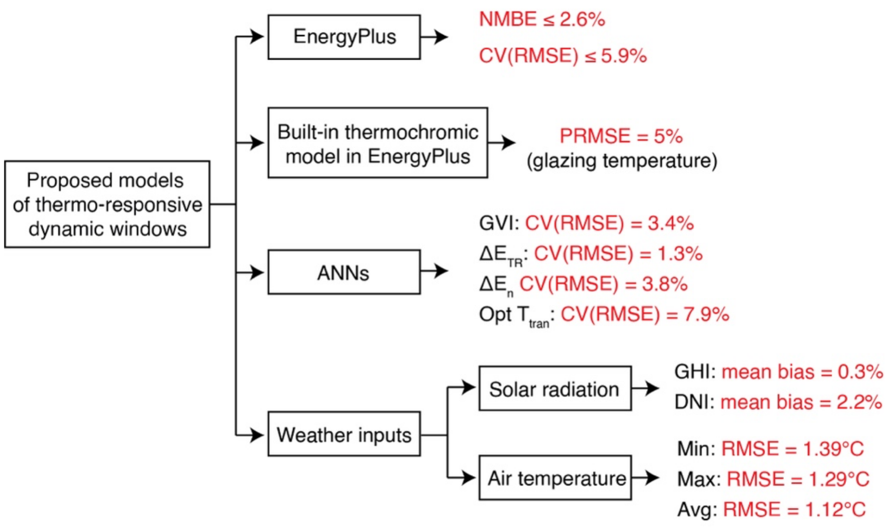

Supplementary Figure 127|Summary of accuracy performance of each component in the proposed models of thermo-responsive dynamic windows.

Supplementary References

1. Deru, M. *et al.* US Department of Energy commercial reference building models of the national building stock. (2011).

2. Gao, Y. *et al.* Parametric study of solid-solid translucent phase change materials in building windows. *Appl Energy* **301**, 117467 (2021).

3. Briggs, R. S., Lucas, R. G. & Taylor, Z. T. Climate Classification for Building Energy Codes and Standards: Part 2 - Zone Definitions, Maps, and Comparisons. *ASHRAE Winter Meetings CD, Technical and Symposium Papers* **2003**, 125–133 (2003).

4. Global solar radiation data. https://globalsolaratlas.info/download/world.

5. Fick, S. E. & Hijmans, R. J. WorldClim 2: new 1-km spatial resolution climate surfaces for global land areas. *International journal of climatology* **37**, 4302–4315 (2017).

6. Global population density map. https://neo.gsfc.nasa.gov/view.php?datasetId=SEDAC_POP.

7. Liang, J., Wang, S., Lei, D., Wang, Z. & Li, X. Enhanced visible and tunable infrared transmittance of W-doped VO2/SiO2/PVP composite films for smart windows. *Opt Mater (Amst)* **121**, 111485 (2021).

8. Guo, H., Wang, Y. G., Fu, H. R., Jain, A. & Chen, F. G. Influence of dopant valence on the thermochromic properties of VO2 nanoparticles. *Ceram Int* **47**, 21873–21881 (2021).

9. Zou, Z. *et al.* Phase transition mechanism and application of silicon-doped VO2 thin films to smart windows. *Journal of Materials Science: Materials in Electronics* **32**, 23825–23833 (2021).

10. Chang, T. *et al.* Mitigating deterioration of vanadium dioxide thermochromic films by interfacial encapsulation. *Matter* **1**, 734–744 (2019).

11. Zhou, X. *et al.* A new strategy of nanocompositing vanadium dioxide with excellent durability. *J Mater Chem A Mater* **9**, 15618–15628 (2021).

12. Shen, N. *et al.* Phase transition hysteresis of tungsten doped VO2 synergistically boosts the function of smart windows in ambient conditions. *ACS Appl Electron Mater* **3**, 3648–3656 (2021).

13. Wang, X. *et al.* Enhancing visible-light transmittance while reducing phase transition temperature of VO2 by Hf–W co-doping. *Appl Phys Lett* **118**, (2021).

14. Zhou, D. *et al.* Symmetrical SnO2/W-doped VO2/SnO2 sandwich structures with high luminous transmittance, excellent solar modulation ability and low phase transition temperature. *Infrared Phys Technol* **123**, 104198 (2022).

15. Xu, Q. *et al.* Anisotropic localized surface plasmon resonance of vanadium dioxide rods in flexible thermochromic film towards multifunctionality. *Solar Energy Materials and Solar Cells* **230**, 111163 (2021).

16. Guo, H., Wang, Y. G., Jain, A., Fu, H. R. & Chen, F. G. Preparation of W/Zr co-doped VO2 with improved microstructural and thermochromic properties. *J Alloys Compd* **878**, 160352 (2021).

17. Kang, J., Liu, J., Shi, F., Dong, Y. & Jiang, S. The thermochromic characteristics of Zn-doped VO2 that were prepared by the hydrothermal and post-annealing process and their polyurethane composite films. *Ceram Int* **47**, 15631–15638 (2021).

18. Rezek, J. *et al.* Transfer of the sputter technique for deposition of strongly thermochromic VO2-based coatings on ultrathin flexible glass to large-scale roll-to-roll device. *Surf Coat Technol* **442**, 128273 (2022).

19. Zhu, Z. *et al.* Preparation and durability evaluation of vanadium dioxide intelligent thermal insulation films. *Colloid Interface Sci Commun* **48**, 100619 (2022).

20. Li, B. *et al.* Thermochromic Ta doped VO2 films: enhanced luminous transmittance, significantly depressed phase transition temperature and hysteresis width. *Appl Surf Sci* **568**, 150959 (2021).

21. Long, S. *et al.* Self-template synthesis of nanoporous VO2-based films: localized surface plasmon resonance and enhanced optical performance for solar glazing application. *ACS Appl Mater Interfaces* **11**, 22692–22702 (2019).

22. Kang, J. *et al.* Facile fabrication of VO2/SiO2 aerogel composite films with excellent thermochromic properties for smart windows. *Appl Surf Sci* **573**, 151507 (2022).

23. Kang, L. *et al.* Nanoporous thermochromic VO2 films with low optical constants, enhanced luminous transmittance and thermochromic properties. *ACS Appl Mater Interfaces* **3**, 135–138 (2011).

24. Wang, N., Peh, Y. K., Magdassi, S. & Long, Y. Surface engineering on continuous VO2 thin films to improve thermochromic properties: top-down acid etching and bottom-up self-patterning. *J Colloid Interface Sci* **512**, 529–535 (2018).

25. Zhou, L. *et al.* Modified color for VO 2/Au/VO 2 sandwich structure-based smart windows. *Applied Physics A* **124**, 1–6 (2018).

26. Xu, F. *et al.* Highly enhanced thermochromic performance of VO2 film using "movable" antireflective coatings. *ACS Appl Mater Interfaces* **11**, 4712–4718 (2019).

27. Yao, L. *et al.* Long-lived multilayer coatings for smart windows: integration of energy-saving, antifogging, and self-healing functions. *ACS Appl Energy Mater* **2**, 7467–7473 (2019).

28. Savorianakis, G. *et al.* VO2 nanostripe-based thin film with optimized color and solar characteristics for smart windows. *J Appl Phys* **129**, (2021).

29. Long, S. *et al.* Application-oriented VO2 thermochromic coatings with composite structures: Optimized optical performance and robust fatigue properties. *Solar Energy Materials and Solar Cells* **189**, 138–148 (2019).

30. Zhan, Y. *et al.* Tuning thermochromic performance of VOx-based multilayer films by controlling annealing pressure. *Ceram Int* **46**, 2079–2085 (2020).

31. Liu, C. *et al.* Index-tunable anti-reflection coatings: Maximizing solar modulation ability for vanadium dioxide-based smart thermochromic glazing. *J Alloys Compd* **731**, 1197–1207 (2018).

32. Kim, K.-S., Son, E.-W., Youn, J. W. & Kim, D. U. Intense pulsed light sintering of vanadium dioxide nanoparticle films and their optical properties for thermochromic smart window. *Mater Des* **176**, 107838 (2019).

33. Jiang, J. C., Bárta, T., Vlček, J., Houška, J. & Meletis, E. I. Microstructure of high-performance thermochromic ZrO2/V0. 984W0. 016O2/ZrO2 coating with a low transition temperature (22° C) prepared on flexible glass. *Surf Coat Technol* **424**, 127654 (2021).

34. Zhao, L. *et al.* Solution-processed VO2-SiO2 composite films with simultaneously enhanced luminous transmittance, solar modulation ability and anti-oxidation property. *Sci Rep* **4**, 7000 (2014).

35. Powell, M. J. *et al.* Intelligent multifunctional VO2/SiO2/TiO2 coatings for self-cleaning, energy-saving window panels. *Chemistry of Materials* **28**, 1369–1376 (2016).

36. Chang, T. *et al.* Optical design and stability study for ultrahigh-performance and long-lived vanadium dioxide-based thermochromic coatings. *Nano Energy* **44**, 256–264 (2018).

37. He, Q. *et al.* PAM-PNIPAM/W-doped VO2 thermochromic hydrogel film with high solar modulation capability for smart windows deployment. *Opt Mater (Amst)* **97**, 109367 (2019).

38. Zhou, Y., Cai, Y., Hu, X. & Long, Y. Temperature-responsive hydrogel with ultra-large solar modulation and high luminous transmission for "smart window" applications. *J Mater Chem A Mater* **2**, 13550–13555 (2014).

39. Tian, J. *et al.* Hybrid thermochromic microgels based on UCNPs/PNIPAm hydrogel for smart window with enhanced solar modulation. *J Alloys Compd* **858**, 157725 (2021).

40. Tian, J. *et al.* Durable, broadband-light-manageable thermochromic hydrogel with adjustable LCST for smart windows application. *Prog Org Coat* **157**, 106287 (2021).

41. Li, X.-H., Liu, C., Feng, S.-P. & Fang, N. X. Broadband light management with thermochromic hydrogel microparticles for smart windows. *Joule* **3**, 290–302 (2019).

42. Wang, Y. *et al.* Tungsten-doped VO2/starch derivative hybrid nanothermochromic hydrogel for smart window. *Nanomaterials* **9**, 970 (2019).

43. Zhang, R. *et al.* Energy-efficient smart window based on a thermochromic microgel with ultrahigh visible transparency and infrared transmittance modulation. *J Mater Chem A Mater* **9**, 17481–17491 (2021).

44. Yang, Y.-S., Zhou, Y., Chiang, F. B. Y. & Long, Y. Temperature-responsive hydroxypropylcellulose based thermochromic material and its smart window application. *RSC Adv* **6**, 61449–61453 (2016).

45. Zhou, Y., Cai, Y., Hu, X. & Long, Y. VO 2/hydrogel hybrid nanothermochromic material with ultra-high solar modulation and luminous transmission. *J Mater Chem A Mater* **3**, 1121–1126 (2015).

46. Zhang, L. *et al.* Energy-saving smart windows with HPC/PAA hybrid hydrogels as thermochromic materials. *ACS Appl Energy Mater* **4**, 9783–9791 (2021).

47. Zhou, Y. *et al.* Liquid thermo-responsive smart window derived from hydrogel. *Joule* **4**, 2458–2474 (2020).

48. Liu, X. & Wu, Y. Experimental characterisation of a smart glazing with tuneable transparency, light scattering ability and electricity generation function. *Appl Energy* **303**, 117521 (2021).

49. Zhu, H. & Wang, L. Smart window based on Cu7S4/hydrogel composites with fast photothermal response. *Solar Energy Materials and Solar Cells* **202**, 110109 (2019).

50. Chen, G. *et al.* Printable Thermochromic Hydrogel-Based Smart Window for All-Weather Building Temperature Regulation in Diverse Climates. *Advanced Materials* 2211716 (2023).

51. Liu, Y. *et al.* Molecularly engineered CMC-caged PNIPAM for broadband light management in energy-saving window. *Carbohydr Polym* **281**, 119056 (2022).

52. Li, G. *et al.* Physical crosslinked hydrogel-derived smart windows: anti-freezing and fast thermal responsive performance. *Mater Horiz* (2023).

53. Nakamura, A., Ogai, R. & Murakami, K. Development of smart window using an hydroxypropyl cellulose-acrylamide hydrogel and evaluation of weathering resistance and heat shielding effect. *Solar Energy Materials and Solar Cells* **232**, 111348 (2021).

54. Tian, J. *et al.* Sunlight-driven photo-thermochromic hybrid hydrogel with fast responsiveness and durability for energy efficient smart windows. *Compos Part A Appl Sci Manuf* **149**, 106538 (2021).

55. Wang, Y. *et al.* Mineralized supramolecular hydrogel as thermo-responsive smart window. *J Mater Sci* **56**, 6955–6965 (2021).

56. Wei, G., Yang, D., Zhang, T., Yue, X. & Qiu, F. Thermal-responsive PNIPAm-acrylic/Ag NRs hybrid hydrogel with atmospheric window full-wavelength thermal management for smart windows. *Solar Energy Materials and Solar Cells* **206**, 110336 (2020).

57. Wang, K. *et al.* Thermo-Responsive Poly (N-isopropylacrylamide)/Hydroxypropylmethyl Cellulose Hydrogel with High Luminous Transmittance and Solar Modulation for Smart Windows. *ACS Appl Mater Interfaces* **15**, 4385–4397 (2023).

58. Zhang, L., Du, Y., Xia, F. & Gao, Y. Two birds with one stone: A novel thermochromic cellulose hydrogel as electrolyte for fabricating electric-/thermal-dual-responsive smart windows. *Chemical Engineering Journal* **455**, 140849 (2023).

59. Lei, Q., Wang, L., Xie, H. & Yu, W. Active-passive dual-control smart window with thermochromic synergistic fluidic glass for building energy efficiency. *Build Environ* **222**, 109407 (2022).

60. Zhang, L., Du, Y., Xia, H., Yang, G. & Gao, Y. HPC-PAA hydrogel smart windows with and without Cs0. 32WO3: High solar modulation ability and luminous transmittance. *Ceram Int* **48**, 37122–37131 (2022).

61. Li, Y., Wang, Y., Lu, J., Wang, W. & Wang, D. Synergistically photothermal Au Nanoprisms@ MXene enable adaptive solar modulation of HA-PNIPAM hydrogels for smart window. *Chemical Engineering Journal* **457**, 141299 (2023).

62. Zhang, R. *et al.* Energy-Efficient Smart Window Based on Thermochromic Hydrogel with Ultrahigh Visible Transparency and Unprecedented Infrared Transmittance Modulation. (2021).

63. Nakamura, A., Yamane, N. & Murakami, K. Development of smart window with hydroxypropyl cellulose-acrylamide hydrogel: Effect of hydroxypropyl cellulose molecular weight on light scattering property. *Optik (Stuttg)* **294**, 171464 (2023).

64. Tan, Y. *et al.* Temperature-responsive 'cloud'with controllable self-assembled particle size for smart window application. *Appl Mater Today* **25**, 101248 (2021).

65. Sun, Z. *et al.* Chameleon-inspired energy-saving smart window responding to natural weather. *ACS Sustain Chem Eng* **9**, 12949–12959 (2021).

66. Ma, D. *et al.* Solar Light Management Enabled by Dual-Responsive Smart Window. *ACS Appl Mater Interfaces* **14**, 56065–56073 (2022).

67. Timusk, M. *et al.* Surface-active thermally responsive hydrogels by emulsion sedimentation for smart window applications. *ACS Appl Polym Mater* **5**, 5937–5950 (2023).

68. Liu, J. *et al.* Dual-function smart windows with dynamic and fast thermal response for building energy-saving/storage. *Solar Energy Materials and Solar Cells* **249**, 112048 (2023).

69. Feng, Y. *et al.* Phase-Changing Polymer Film for Smart Windows with Highly Adaptive Solar Modulation. *ACS Appl Mater Interfaces* **15**, 5836–5844 (2023).

70. Zheng, L. *et al.* Energy-saving thermochromic smart shield based on double network hydrogel with intelligent light management and robust mechanical property. *Composites Communications* **42**, 101684 (2023).

71. Li, J. *et al.* A Facile yet Versatile Strategy to Construct Liquid Hybrid Energy-Saving Windows for Strong Solar Modulation. *Advanced Science* **10**, 2206044 (2023).

72. Wang, Y. *et al.* VO2@ SiO2/Poly (N-isopropylacrylamide) hybrid nanothermochromic microgels for smart window. *Ind Eng Chem Res* **57**, 12801–12808 (2018).

73. Feng, Y. *et al.* Entanglement in Smart Hydrogels: Fast Response Time, Anti-Freezing and Anti-Drying. *Adv Funct Mater* **33**, 2211027 (2023).

74. Lei, Q. *et al.* Novel Photothermochromic Smart Window Based on PNIPAm-glass-MXene/PAM with High Shield, Fast Response, and Excellent Stability. *Solar RRL* **7**, 2200990 (2023).

75. Kakiuchida, H., Kabata, M., Matsuyama, T. & Ogiwara, A. Thermoresponsive reflective scattering of meso-scale phase separation structures of uniaxially orientation-ordered liquid crystals and reactive mesogens. *ACS Appl Mater Interfaces* **13**, 41066–41074 (2021).

76. Guo, S.-M. *et al.* Preparation of a thermally light-transmittance-controllable film from a coexistent system of polymer-dispersed and polymer-stabilized liquid crystals. *ACS Appl Mater Interfaces* **9**, 2942–2947 (2017).

77. Oh, S., Kim, S., Baek, J. & Yoon, T. Optical and thermal switching of liquid crystals for self-shading windows. *Adv Sustain Syst* **2**, 1700164 (2018).

78. McAndrews, G. R., Yeang, A. L., Cai, Y., Barile, C. J. & McGehee, M. D. Understanding and Improving Mechanical Stability in Electrodeposited Cu and Bi for Dynamic Windows Based on Reversible Metal Electrodeposition. *Adv Energy Mater* **13**, 2202843 (2023).

79. Yeang, A. L. *et al.* Transparent, High-Charge Capacity Metal Mesh Electrode for Reversible Metal Electrodeposition Dynamic Windows with Dark-State Transmission< 0.1%. *Adv Energy Mater* **12**, 2200854 (2022).

80. Islam, S. M., Hernandez, T. S., McGehee, M. D. & Barile, C. J. Hybrid dynamic windows using reversible metal electrodeposition and ion insertion. *Nat Energy* **4**, 223–229 (2019).

81. Strand, M. T. *et al.* Factors that determine the length scale for uniform tinting in dynamic windows based on reversible metal electrodeposition. *ACS Energy Lett* **3**, 2823–2828 (2018).

82. Madu, D. C., Islam, S. M., Pan, H. & Barile, C. J. Electrolytes for reversible zinc electrodeposition for dynamic windows. *J Mater Chem C Mater* **9**, 6297–6307 (2021).

83. Zhao, X. *et al.* Dynamic glazing with switchable solar reflectance for radiative cooling and solar heating. *Cell Rep Phys Sci* **3**, (2022).

84. Shi, Y. *et al.* Structure modulated amorphous/crystalline WO3 nanoporous arrays with superior electrochromic energy storage performance. *Solar Energy Materials and Solar Cells* **212**, 110579 (2020).

85. Hemaida, A., Ghosh, A., Sundaram, S. & Mallick, T. K. Evaluation of thermal performance for a smart switchable adaptive polymer dispersed liquid crystal (PDLC) glazing. *Solar Energy* **195**, 185–193 (2020).

86. Ghosh, A. & Mallick, T. K. Evaluation of optical properties and protection factors of a PDLC switchable glazing for low energy building integration. *Solar Energy Materials and Solar Cells* **176**, 391–396 (2018).
