# Supplementary material for: Global and regional perspectives on optimizing thermo-responsive dynamic windows for energy-efficient buildings
Source: Nat Commun. 2025 Jan 2;16:199. doi: 10.1038/s41467-024-54967-8 (PMC11695856; doi:10.1038/s41467-024-54967-8)
Supplement: Supplementary file 2 — Description of Additional Supplementary Files [file 41467_2024_54967_MOESM2_ESM.docx]

**Description of Additional Supplementary Files**

File Name: Supplementary Video 1
Description: A tutorial and demonstration of the open-source tool PyDynamicWindow for researchers and Python beginners.
